# Extracellular vesicles secreted by 3D tumor organoids are enriched for immune regulatory signaling biomolecules compared to conventional 2D glioblastoma cell systems

**DOI:** 10.3389/fimmu.2024.1388769

**Published:** 2024-04-25

**Authors:** Martina Schuster, Frank K. Braun, Dapi Meng-Lin Chiang, Christina Ludwig, Chen Meng, Christian Grätz, Benedikt Kirchner, Martin Proescholdt, Peter Hau, Ortrud K. Steinlein, Michael W. Pfaffl, Markus J. Riemenschneider, Marlene Reithmair

**Affiliations:** ^1^ Institute of Human Genetics, University Hospital, Ludwig-Maximilians-University Munich, Munich, Germany; ^2^ Department of Neuropathology, Regensburg University Hospital, Regensburg, Germany; ^3^ Division of Animal Physiology and Immunology, School of Life Sciences Weihenstephan, Technical University of Munich, Freising, Germany; ^4^ Bavarian Center for Biomolecular Mass Spectrometry (BayBioMS), School of Life Sciences Weihenstephan, Technical University of Munich, Freising, Germany; ^5^ Department of Neurosurgery, Regensburg University Hospital, Regensburg, Germany; ^6^ Department of Neurology and Wilhelm Sander Neuro-Oncology Unit, Regensburg University Hospital, Regensburg, Germany

**Keywords:** glioblastoma multiforme, brain cancer, 3D organoid model, extracellular vesicle, EV, microRNA, proteomics

## Abstract

**Background:**

Newer 3D culturing approaches are a promising way to better mimic the *in vivo* tumor microenvironment and to study the interactions between the heterogeneous cell populations of glioblastoma multiforme. Like many other tumors, glioblastoma uses extracellular vesicles as an intercellular communication system to prepare surrounding tissue for invasive tumor growth. However, little is known about the effects of 3D culture on extracellular vesicles. The aim of this study was to comprehensively characterize extracellular vesicles in 3D organoid models and compare them to conventional 2D cell culture systems.

**Methods:**

Primary glioblastoma cells were cultured as 2D and 3D organoid models. Extracellular vesicles were obtained by precipitation and immunoaffinity, with the latter allowing targeted isolation of the CD9/CD63/CD81 vesicle subpopulation. Comprehensive vesicle characterization was performed and miRNA expression profiles were generated by smallRNA-sequencing. *In silico* analysis of differentially regulated miRNAs was performed to identify mRNA targets and corresponding signaling pathways. The tumor cell media and extracellular vesicle proteome were analyzed by high-resolution mass spectrometry.

**Results:**

We observed an increased concentration of extracellular vesicles in 3D organoid cultures. Differential gene expression analysis further revealed the regulation of twelve miRNAs in 3D tumor organoid cultures (with nine miRNAs down and three miRNAs upregulated). MiR-23a-3p, known to be involved in glioblastoma invasion, was significantly increased in 3D. MiR-7-5p, which counteracts glioblastoma malignancy, was significantly decreased. Moreover, we identified four miRNAs (miR-323a-3p, miR-382-5p, miR-370-3p, miR-134-5p) located within the DLK1-DIO3 domain, a cancer-associated genomic region, suggesting a possible importance of this region in glioblastoma progression. Overrepresentation analysis identified alterations of extracellular vesicle cargo in 3D organoids, including representation of several miRNA targets and proteins primarily implicated in the immune response.

**Conclusion:**

Our results show that 3D glioblastoma organoid models secrete extracellular vesicles with an altered cargo compared to corresponding conventional 2D cultures. Extracellular vesicles from 3D cultures were found to contain signaling molecules associated with the immune regulatory signaling pathways and as such could potentially change the surrounding microenvironment towards tumor progression and immunosuppressive conditions. These findings suggest the use of 3D glioblastoma models for further clinical biomarker studies as well as investigation of new therapeutic options.

## Background

Isocitrate dehydrogenase (IDH) wild-type glioblastoma multiforme (GBM), (central nervous system tumor World Health Organization classified grade 4) is the most common malignant tumor of the adult brain, characterized by its aggressive and infiltrative growth and very poor clinical outcome (1). Tumor cell heterogeneity for GBM is frequently reported. Several transcriptional subtypes (i.e. proneural, mesenchymal and classic) are identified for GBM and used for subclassification (2–4). GBM forms a complex and heterogeneous tumor microenvironment (TME) composed of tumor cells and non-malignant stromal cells, including endothelial cells and brain resident glial cells (oligodendrocytes, astrocytes, ependymal cells and microglia), as well as infiltrating immune cells such as myeloid monocytes/macrophages and lymphocytes (5). Moreover, the TME consists of non-cellular components such as apocrine and paracrine signaling molecules like extracellular vesicles (EVs), extracellular matrix components and secreted enzymes that remodel the extracellular matrix (6). During tumor progression, malignant cells alter the organization of the surrounding stroma by continuously exchanging information transmitted through secretion of the effector molecules, cell-cell gap junctions, and tunneling nanotubes (7). The close interaction between the different components of the TME may further promote tumor invasiveness and resistance to therapy (8).

Currently, there are no reliable biomarkers for GBM that allow a timely diagnosis. Conventional 2D *in vitro* models grown in monolayers or as small spheres in suspension are still the basis for exploring new and promising therapeutic approaches. It is well recognized that *in vitro* culture conditions often lack the cellular complexity organization, secretion and cell signaling found in primary tumors. Particularly the lack of disease-specific TME hampers the development of new therapeutic strategies for GBM (9). One promising approach to ameliorate some aspects of *in vitro* (2D) culturing are 3D tumor organoid models. They are shown to maintain and regenerate a heterogeneous tumor cell population and thereby better represent the complex microenvironment found in GBM (10, 11).

Membrane-enveloped EVs represent one of the conceivable ways in which GBM maintains the large heterogeneity within the tumor. EVs can transport biomolecules such as proteins, lipids and nucleic acids, such as miRNAs, over short or long distances between cells, ensuring coordinated release for intercellular communication. EVs are secreted by all cells including cancer cells and can contain various biomolecules. In general, EVs can be classified into three major subtypes based on their size, biogenesis, and content. These include exosomes (30-150 nm in diameter), microvesicles (also called ectosomes or shedding vesicles) (100-1000 nm) and apoptotic bodies (50- 5000 nm) (12, 13). The clinical relevance of EVs, especially the exosomes and microvesicles, are underlined by their ability to transport biomarkers or therapeutic RNA. They are also regarded as a promising biomolecule/shuttle for the development of new therapeutic strategies in GBM, as they play an important role in tumor pathogenesis (14, 15). In GBM, EVs have been shown to be involved in several key processes, including tumor cell proliferation, survival, migration and invasion, as well as angiogenesis and immunomodulation (16). However, current knowledge about how different culture condition such as 3D tumor organoids alter the EVs secretion in quality and quality are still limited. Challenges in purifying/separating different subpopulations and study their cargo remain.

In this study, we addressed this knowledge gap by comprehensively analyzing EVs of primary GBM cultured in 3D culture organoid models compared to EVs from the conventional 2D culture from the same patient based on their EV properties, miRNA and protein profiles.

## Methods

### Patient sampling

GBM cell models were generated from freshly resected brain tumor tissue collected and processed in a collaboration with the Department of Neurosurgery, Department of Neuropathology at the University Hospital Regensburg. All tumors were classified according to the WHO 2021 diagnostic criteria (1). In the study: Six glioblastomas, IDH wild-type (CNS WHO grade 4) of which three were subclassified as proneural and three as mesenchymal. Of each GBM model conventional (2D) and tumor organoid (3D) cultures were investigated (for further details see **Table 1**).

**Table 1:** Clinical information for the six selected GBM primary cell lines.

Cell line	Sex	Age	Cancer type	subgroup	IDH status
BTIC10	male	48	GBM grade IV	mesenchymal	wild-type
BTIC13	male	44	GBM grade IV	mesenchymal	wild-type
BTIC131	female	65	GBM grade IV	mesenchymal	wild-type
BTIC18	male	50	GBM grade IV	proneural	wild-type
BTIC129	female	63	GBM grade IV	proneural	wild-type
BTIC155	female	65	GBM grade IV	proneural	wild-type

BTIC, brain tumor initiating cells; GBM, glioblastoma multiforme.

### Cell culturing

Six glioblastomas, IDH wild-type (CNS WHO grade 4) derived models (BTIC10, -13, -131, -18, -129, -155) were cultured in RHB-A (Takara Bio, Kusatsu, Japan) growth media supplemented with penicillin/streptomycin (Merck, Darmstadt, Germany), epidermal growth factor (20 ng/ml, ReliaTech, Wolfenbüttel, Germany), and basic fibroblast growth factor (20 ng/ml, ReliaTech, Wolfenbüttel, Germany). Cultures were kept at 37°C, with 5% CO2 and 95% humidity, growth media was changed once a week. Conventional cultures (2D) were maintained in standard cell culture flasks and passaged when 80% confluency was reached. Tumor organoid models (3D) were generated by mixing 20000 GBM cells in 80 µl Matrigel™ largely following a previously published protocol (11, 17). 3D cultures were further kept on an orbital shaker to prevent attachment, multiple 3D spheroids were kept in 5 ml petri dish. Supernatant of cultures was harvested, centrifuged at 200 g x 5 min, and stored at -80°C.

### EV isolation

EVs were isolated from each supernatant sample by two different isolation methods, precipitation (PP) (miRCURY exosome cell/urine/CSF kit, Qiagen, Hilden, Germany) and immunoaffinity (IA) (EV isolation kit pan, human, Miltenyi Biotec, Bergisch Gladbach, Germany) for EV characterization and in duplicates for smallRNA-Sequencing (smallRNA-Seq) according to the suppliers’ protocols. Briefly, for EV isolation by IA, supernatant was centrifuged at 10000 x g for 45 min. Supernatant was vortexed with 50 µl microbeads for 5s and incubated for 1h on an overhead rotor. Column was equilibrated and then washed three times before and four times with sample on a magnetic stand. EVs were eluted with 100 µl isolation buffer (provided in the kit). For EV isolation by PP, supernatant was centrifuged at 3200 x g for 5 min and supernatant was mixed with precipitation buffer (provided in the kit). After incubation for 1h at 4°C, sample was centrifuged at 3200 x g for 30 min and supernatant was discarded. The pellet was resuspended in 100 µl resuspension buffer (provided in the kit) by vortexing for 15s. EVs were stored at -80°C until further processing.

### EV characterization

For transmission electron microscopy (TEM) analysis, 5 µl EV sample was pipetted onto parafilm. A grid was laid onto the drop and incubated for 10 min. Next, the grid was laid onto a 100 µl uranyl acetate drop for 5 min for negative staining and dried at room temperature for 30 min.

For particle numbers and sizes, nanoparticle tracking analysis (NTA) was performed. A ZetaView PMX 110 device equipped with a 250 nm laser (Particle Metrix, Inning am Ammersee, Germany) was used. EV suspensions were appropriately diluted in particle-free PBS and analyzed in 2 imaging cycles at 11 positions each. 5 µg/ml of the membrane dye CellMask Orange™ (CMO) was used for particle fluorescence measurements. Settings for video capture in fluorescence mode were adjusted to 70°C for shutter, 95% for sensitivity and a frame rate of 30 per second. Data analysis was performed using the ZetaView software version 8.05.11 SP1 (Particle Metrix GmbH, Inning am Ammersee, Germany).

### EV flow cytometry

Carboxyfluorescein succinimidyl ester (CFSE) was used to distinguish between vesicle membranes and non-vesicles particles (18, 19). Anti-CD9, CD63, CD81 and TSG101 antibodies were used to identify human EVs (13) and Cytochrome c as non-EVs marker (20). CD44, C1Q and SOX2 were used as glioblastoma biomarkers (20–23).

### EV surface marker staining

2E8 PP EVs particles or 5E7 IA EVs particles were first diluted in 400 µl PBS (Sigma + Merck, Darmstadt, Germany). 100 µl of master antibody mix containing 4 µl of APC-CD9 antibody (HI9a clone: 1:125 dilution) (Biolegend, San Diego, CA, USA), 3 µl of PE/Cyanine7-CD63 antibody (H5C6 clone: 1:166 dilution) (Biolegend, San Diego, CA, USA), 5 µl of PE-CD81 antibody (5A6 clone: 1:100 dilution) (Biolegend, San Diego, CA, USA), 4 µl of Brilliant Violet 421™-CD44 antibody (BJ18 clone: 1:125 dilution) (Biolegend, San Diego, CA, USA), 1 µl of 5 mM CFSE stock solution (1:5000 dilution) (Biolegend, San Diego, CA, USA). To ensure specificity of our results, isotype controls (IgG) (Biolegend, San Diego, CA, USA) with non-CFSE staining were included for analysis as negative controls. 400 µl of EVs were mixed with 100 µl of master antibody mix and incubated for 30 min at 37°C. One ml of PBS was further added to the antibodies/CFSE stained EVs and subsequently prepared on the BD LSR Fortessa™ (Becton, Dickinson and Company, Franklin Lakes, NJ, USA). The data analysis including gating was performed by using FlowJo version 10.8 (Becton, Dickinson and Company, Franklin Lakes, NJ, USA). In the flow cytometry gating strategy, antibody-/CFSE-stained EVs were initially gated by FSC-H and SSC-H to identify vesicle populations. To enhance the accuracy of EV signal, the double positive population was gated based on CFSE+ and CD9+, CFSE+ and CD63+, CFSE+ and CD81+, and CFSE+ and CD44+ populations, as determined by comparison to the isotype control. Percentages of each sample were subsequently subtracted from their own IgG control to accurately determine the purity of EVs isolated by PP and IA methods and minimize potential distortions arising from nonspecific antibody binding.

### Bead-based intracellular marker staining

First, 250 μl of 40 μg/ml 1-ethyl-3-(3-dimethylaminopropyl) carbodiimide hydrochloride (EDC) (Thermo Fisher Scientific, Bremen, Germany), in 0.1 M 2-(N-Morpholino) ethanesulfonic acid (MES) (Sigma + Merck, Darmstadt, Germany), pH 5.0, to activate 10 mg of carboxyl group magnetic particles (2.13E+10 particles/ml, IKERLAT Polymers, Lasarte-Oria, Spain) at 25°C for a 15 min incubation was used. The activated magnetic particles were resuspended in 1 ml of PBS. 5E+7 PP EVs particles in 1ml PBS were incubated with 25 μl of activated magnetic particles at 37°C for 2h. Beads only were included as negative control. To saturate empty bead binding site, PP EV-bead complexes were further blocked with 1 ml of 10% EV-free BSA in PBS (100000 g, 4°C for overnight centrifugation) at 37°C for 30 min. PP EV-bead complexes and negative control bead only were fixed by 200 μl of 4% paraformaldehyde in PBS for 10 min at 25°C, penetrated by 200 μl of 0.1% Tween-20 in PBS for 10 min at 25°C and blocked by 200 μl of 10% EV-free BSA in PBS 10% EV-free BSA in PBS (100000 g, 4°C for overnight centrifugation) for 30 min at 25°C. Fixed/penetrated PP EV-bead complexes and EV-free beads only as negative control were further stained with 200 μl master antibody mix at 37°C for 1h incubation. Master antibody mix contained 5 μl of PE-TSG101 antibody (EPR7130(B) clone: 1:40 dilution) (Abcam, Cambridge, UK), 5 μl of Alexa Fluor^®^ 647-SOX2 antibody (14A6A34 clone: 1:40 dilution) (Biolegend, San Diego, CA, USA), 5 μl of Alexa Fluor^®^ 488-Cytochrome c antibody (6H2.B4 clone: Biolegend, San Diego, CA, USA) and 5 μl of PE-Cy7-C1Q antibody (orb871651 clone: 1:40 dilution) (Biorbyt, Cambridge, UK). Antibodies-stained PP EV-bead complexes were further washed twice with 1 ml 0.5% EV-free BSA in PBS (100000 g, 4°C for overnight centrifugation) on the magnetic rank and subsequently prepared on the BD LSR Fortessa™ (Becton, Dickinson and Company, Franklin Lakes, USA). The data analysis including gating was performed by using FlowJo version 10.8 (Becton, Dickinson and Company, Franklin Lakes, USA). To ensure specificity of our results, an IgG control was included in the analysis. In the flow cytometry gating strategy, the complexes of antibody-stained PP EV-beads and EV-free beads (negative control) were initially gated using FSC-A and SSC-A. The double-positive population, indicative of glioblastoma EVs, was further refined based on TSG101+ and SOX2+ or TSG101+ and C1Q+ expression, as determined through comparison with the isotype control and the EV-free beads (negative control).

To assess EV purity, EV-bead complexes were gated as cytochrome c positive or negative and TSG101 positive or negative, again referenced against the isotype control and the EV-free beads. Cytochrome c+ and TSG101- identified non-EV populations, cytochrome c- and TSG101+ represented EV populations, while cytochrome c+ and TSG101+ denoted mitochondrial EV populations. Percentages of each sample were subsequently adjusted based on their respective IgG controls to accurately determine EV purity and mitigate potential distortions resulting from nonspecific antibody binding.

### Library preparation and sequencing

EVRNA extraction was performed using the miRNeasy mini kit (Qiagen, Hilden, Germany) as per the supplier’s protocol starting with 700 µl QIAzol lysis reagent and applying optional step 10 and 13 according to the protocol. RNA eluates were concentrated to 6 µl by evaporation. SmallRNA library preparation was performed in duplicates using the NEBNext Multiplex SmallRNA Library Prep Set for Illumina, Ipswich, MA, USA) as described in (24). Size distribution of final libraries was assessed by capillary electrophoresis (DNA 1000 Assay, 2100 Bioanalyzer, Agilent Technologies, Santa Clara, CA, USA) prior to 50 sequencing cycles on a NovaSeq 6000 platform (Illumina, San Diego, CA, USA). All samples passed quality control.

### Bioinformatic analyses of sequencing data

Sequencing data was processed using an in-house pipeline described previously (24). After assessing technical sequencing quality using FastQC, adaptor sequences were clipped using Btrim. Reads without adaptors as well as reads shorter than 16 nucleotides were removed. Remaining reads were sequentially aligned to sequences from human non-coding RNAs (rRNA, tRNA, snRNA, snoRNA) from RNAcentral (release 22) (25). Next, reads that did not map to these RNA classes were aligned to miRBase (release 22) (26). All alignment steps were performed using Bowtie’s “best” algorithm and allowing one mismatch (27). Read counts for each RNA class were extracted from Bowtie output. Normalization, differential expression analysis (DGE) and false discovery rate (FDR) according to Benjamini-Hochberg correction (padj) were carried out using DESeq2 (28) and R (29).

### Protein preparation for proteomics

30 µg of cell culture media were first measured by BCA assay (Thermo Fisher Scientific Inc., Waltham, USA) and then mixed with 7.5 µl of 4x Laemmli Sample Buffer (Bio-Rad Laboratories, Hercules, USA) in a final volume 30 µl. 30 µl of cell culture media proteins with final 1x Laemmli Sample Buffer were boiled at 70°C for 10 min and sonicated at 4°C for 5 min. The sonicated protein extracts were further sent to the proteomics core facility (Bavarian Center for Biomolecular Mass Spectrometry).

25 µl EVs were first mixed with 25 µl 2x RIPA buffer (Abcam plc, Cambridge, UK; ab156034) and stored at -80°C for overnight incubation. Next, samples were boiled at 70°C for 10 min and sonicated at 4°C for 5 min. The sonicated protein extracts were centrifuged at 10,000 rcf at 4°C for 30 min. Protein concentration was measured by performing a BCA assay (Thermo Fisher Scientific Inc., Waltham, USA). 2 µg of EV proteins were further mixed with ddH_2_O and 5 µl of 4x Laemmli Sample Buffer (Bio-Rad Laboratories, Hercules, USA) with 2-mercaptoethanol (Merck KGaA, Darmstadt, Germany) in a final volume of 20 µl. 20 µl of EV proteins with 1x Laemmli Sample Buffer were sent to the proteomics core facility (Bavarian Center for Biomolecular Mass Spectrometry).

### Proteomic sample preparation

According to standard procedures, in-gel trypsin digestion of all plasma EV samples was performed (30). Briefly, the protein samples were loaded on a Nu-PAGE™ 4%–12% Bis‐Tris protein gel (Thermo Fisher Scientific, Germany) for about 1 cm. Subsequently, the accumulated and not size‐separated single protein band per sample was cut out, reduced (50 mM dithiothreitol, Carl Roth, Germany), alkylated (55 mM chloroacetamide, Merck, Germany), and digested overnight with trypsin (Trypsin Gold, mass spectrometry grade, Promega, USA). The dry peptide samples were resuspended in 25 µl buffer A (2% acetonitrile, 0.1% formic acid in HPLC grade water), of which 5 μl were injected per mass spectrometry (MS) measurement.

### Proteomic data acquisition

Liquid chromatography (LC)-MS/MS measurements were carried out on a Dionex Ultimate 3000 RSLCnano system coupled to a Q-Exactive HF-X mass spectrometer (Thermofisher Scientific, Germany). Injected peptides were delivered to a trap column (ReproSil-our C18-AQ, five μm, 20 mm × 75 μm, self-packed, Dr. Maisch, Germany) at a flow rate of 5 μL/min in 100% solvent A (0.1% formic acid in HPLC grade water). After 10 min of loading, peptides were transferred to an analytical column (ReproSil Gold C18-AQ, three μm, 450 mm × 75 μm, self-packed, Dr. Maisch, Germany) and separated using a 50 min gradient from 4% to 32% of solvent B (0.1% FA, 5% DMSO in acetonitrile) in solvent A (0.1% FA, 5% DMSO in HPLC grade water) at 300 nL/min flow rate. The Q-Exactive HF-X mass spectrometer was operated in data-dependent acquisition and positive ionization mode. MS1 spectra (360–1300 m/z) were recorded at a resolution of 60k using an automatic gain control target value of 3E6 and a maximum injection time of 45 msec. Up to 18 peptide precursors were selected for fragmentation. Only precursors with charge states 2 to 6 were selected, and dynamic exclusion of 25 sec was enabled. Peptide fragmentation was performed using higher-energy collision-induced dissociation and normalized collision energy of 26%. The precursor isolation window width was set to 1.3 m/z. MS2 Resolution was 15.000 with automatic gain control target value of 1E+5 and a maximum injection time of 25 msec (complete proteome).

### Proteomic data analysis

Peptide identification and quantification were performed using the software MaxQuant (version 1.6.3.4) (31) with its built‐in search engine Andromeda (32). MS2 spectra were searched against the human protein database from Uniprot (UP000005640, downloaded July 2020, 20353 protein entries) supplemented with common contaminants (built‐in option in MaxQuant). Trypsin/P was specified as a proteolytic enzyme, and carbamidomethylated cysteine was set as a fixed modification. Oxidation of methionine and acetylation at the protein N‐terminus were defined as variable modifications. Results were adjusted to a 1% FDR on peptide spectrum match level and protein level employing a target‐decoy approach using reversed protein sequences. Protein label-free quantification (LFQ) intensities were log10 transformed and further analyzed using Perseus version v2.0.9.0 (33). The built-in filter functions of Perseus were used to filter out “Reverse”, “Only identified by site” and “Potential contaminates”. Only proteins with a peptide count higher than 1 were considered. For the analysis of the cell culture medium data, we additionally removed all proteins that were detected in the pure medium control samples. For determination of differential proteins between 3D versus 2D EV samples, the log10-transformed datasets were filtered using the function “Filter rows based on valid values”, which was set to “3 in at least one group” in Perseus version v2.0.9.0 (33). Missing values were imputed by “normal distribution” (default Perseus setting: width = 0.3, down shift = 1.8). Imputed datasets were further tested by a paired t-tests and Benjamini-Hochberg correction (FDR: 0.05). The generation of volcano plots involved plotting the log10 protein fold-changes on the x-axis and the -log10 t-test p-values on the y-axis for protein pairwise comparisons.

### Normalization index of cell culture medium proteomic analysis

In order to compare 2D and 3D cultures, EV concentration in the samples was first determined using NTA. However, it was not possible to calculate the cell numbers in 3D cultures. We could not directly compare particle numbers between 2D and 3D cultures due to unknown cell numbers. For this reason, we assumed that EVs/proteins were cumulatively secreted from both the 2D and 3D cultures. We created a calibration index to calibrate the NTA results. BTIC18 was the most stable cell line throughout all 6-time points of culture and was therefore used as the model cell line for calculating the normalization index. Proteomic analysis using BTIC18 at six time points was performed to determine reliable and measurable protein parameters that could be used for normalization.

For the 2D and 3D index, log10-transformed LFQ intensities from six time points were filtered using the function ‘Filter rows based on valid values’ and set to ‘6 in total.’ The filtered results were identified as consistent proteins across the 2D or 3D cell culture medium at different time points (starting from time point 2). These consistent proteins were further analyzed by dividing them by the values at the first time point. The normalized results were then used to determine the 2D and 3D index.

### *In silico* analysis

Reactome pathway analysis was applied to identify pathways of our smallRNA-Seq miRNA expression data. For statistical significance, we only considered pathways with a FDR of <0.05. The miRNA targets identified in miRTarBase were entered into the Reactome database, and strong experimentally confirmed relationships between miRNA and target mRNAs by qPCR, Western blot, and reporter assay or experimentally verified associations by additional methods, including microarray and next generation sequencing (NGS), were considered for miRNA effects analysis.

### Statistics and applied software

The normality of data was determined before performing statistical analyses. Analysis were performed using GraphPad Prism 9.0 (GraphPad, San Diego, CA, USA) and expressed as mean ± SEM. Data were assessed by a paired Student’s t test for EV size determination and concentration analysis of two individual groups (2D and 3D) and by a multiple t-test (paired/nonparametric test: Wilcoxon matched-pairs signed rank test) for flow cytometry measurements. Enrichment bars of the Reactome overrepresentation were plotted using http://www.bioinformatics.com.cn/srplot, an online platform for data analysis and visualization.

## Results

### Characterization of GBM derived EVs shows differences in size distribution and comparable marker expression in 3D versus 2D

**Figure 1:**
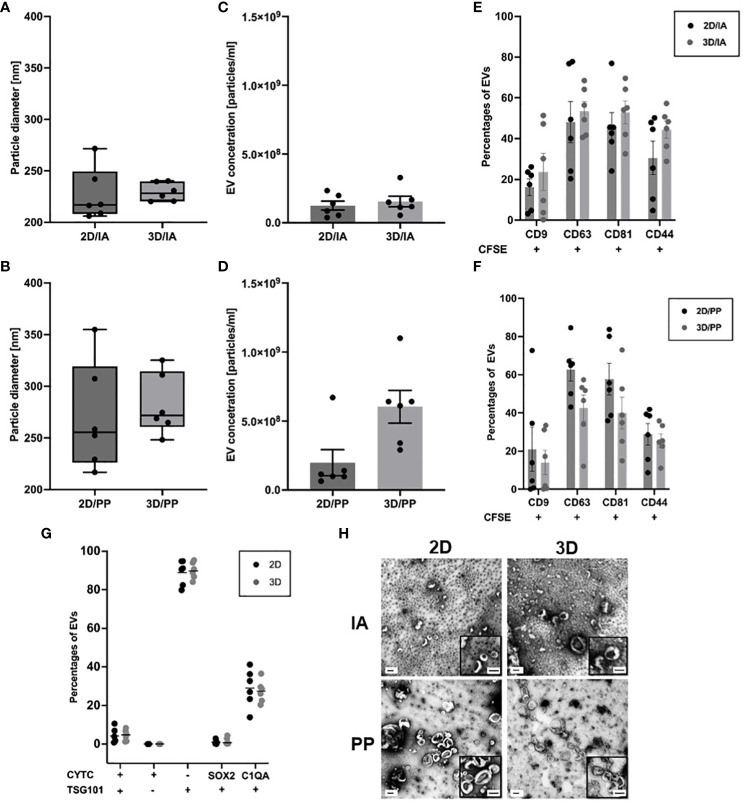
EV characterisation in 3D organoids and corresponding 2D models. **(A)** Size measurements of purified EVs after IA and **(B)** PP from 2D and 3D cultures. Box-Whisker Plots of the median (line) (n=6). Concentration measurements of purified EVs after **(C)** IA and **(D)** PP from 2D and 3D cultures and corrected for sample dilution. Data are shown as mean ± SEM (n=6). **(E)** Surface– and bead-based flow cytometry: Percentages of CD81+, CD63+, CD9+, CD44+ EVs isolated by IA and **(F)** PP (n=6, multiple t-test). **(G)** Percentages of CYTC+,TSG101+, SOX2+,C1QA+ EVs isolated by PP. Data are shown as mean ± SEM (n=6, multiple t-test); **(H)** TEM images of purified EVs obtained from 2D and 3D. Images show wide-field and close-up views (bottom right box). Scale bars show 100 nm.

For size determination and quantification, NTA was performed. NTA confirmed EV preparations with the narrowest particle diameter ranging from 221 to 240 nm and found by EVs derived from 3D culture and isolated by IA (**Figure 1A**). The widest size distribution, ranging from 217 to 355 nm, was observed for EVs from 3D culture and isolated by PP (**Figure 1B**). Overall, EV characterization revealed similar size distribution by NTA when comparing 2D with 3D. However, the results indicate that the IA procedure isolates an EV subpopulation that is smaller on average than that of the PP method.

In terms of concentration measurements, the lowest particle concentration of 1.25E+08 ± 2.97E+07 particles/ml was observed by EVs derived from 2D culture and by IA (**Figure 1C**). The highest particle concentration of 6.03E+08 ± 1.08E+08 particles/ml for EVs was obtained from 3D culture and isolated by PP method (**Figure 1D**). We determined a 2D index of 1.004 and a 3D index of 1.092. For analysis, we first identified the consistent proteins from all six time points, 2D (77 proteins) and 3D (3 proteins), respectively. The protein IDs are listed in the **Supplementary Data Sheet 1**. Dividing the BTIC18 EV concentration by the individual index and comparing 2D and 3D, we found that the EV yield in the supernatant of the 3D cell model was significantly higher than in the 2D model using the PP approach (**Figure 1D**).

We were also able to quantify intact EVs by the amount of CFSE^+^ events using flow cytometry. To further confirm the presence of EVs, we assessed the enrichment of the tetraspanins CD9, CD63, CD81 by surface marker analysis. EVs purified from 2D or 3D cells showed the expected enrichment of the EV marker proteins CD9, CD63, CD81. We found substantial amounts of CD81 and CD63 (about 60% on average) and lower amounts of CD9 (about 20% on average) in independent EV isolates of 2D and 3D cell models (**Figure 1E, F**; **Supplementary Data Sheet 2**). In addition, TSG101, as a typical intracellular EV marker, could be detected to a comparable proportion (about 90% on average) in EVs of 2D and 3D cells following PP isolation (**Figure 1G**; **Supplementary Data Sheet 2**). Moreover, our results showed the presence of GBM-related surface marker CD44 (about 30% on average) and GBM-related intracellular markers C1QA (about 30% on average) with no significant differences between 2D and 3D. SOX2 positive EVs were almost absent (**Figure 1G**; **Supplementary Data Sheet 2**). Besides exosomes and microvesicles, there are also apoptotic bodies, which can occur in increased numbers under certain conditions and are particularly conspicuous due to their variable sizes of 50- 5000 nm (34). To rule out the presence of apoptotic bodies, we have measured cytochrome c (CYTC) (20), which according to MISEV2018 is also a non-EV marker (13). We could rarely detect CYTC and can thus confirm the absence of apoptotic bodies (**Figure 1G**).

Investigating EV morphology by TEM, both cultivation methods and applied EV isolation methods exhibited the “cup-shape” morphologies typical of EVs, which can be attributed to drying during grid preparation (**Figure 1H**). The corresponding sizes in TEM are more than 100 nm smaller than the hydrodynamic sizes estimated in NTA, since the TEM size measurement was performed on dried EVs.

### Cell cultivation influences the EV-associated miRNA profile

**Figure 2:**
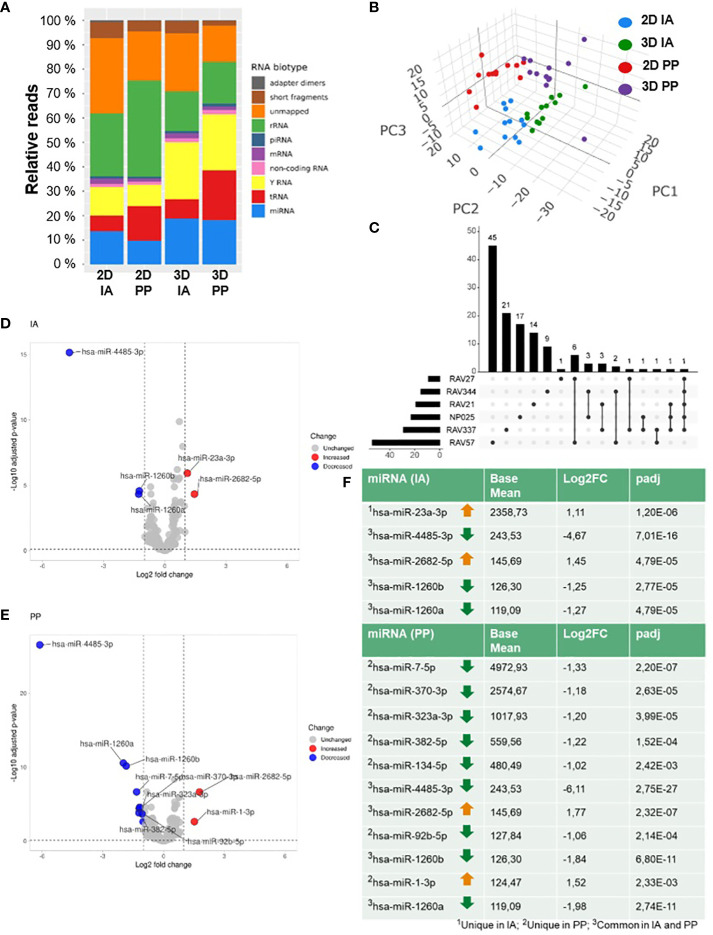
SmallRNA-Seq of 2D and 3D GBM models. **(A)** Graphical representation of the distribution of RNA reads from SmallRNA-Seq aligning to different EVRNAs from 2D or 3D culture and after IA and PP isolation; **(B)** PCA of total analyzed samples (n=48). **(C)** UpSet Plot shows the pairwise comparison of 2D and 3D with the number of unique and common differentially regulated miRNAs in six GBM cell lines. **(D)** Volcano plots of differentially expressed EVmiRNAs after IA and **(E)** PP isolation. **(F)** DGE analysis: Table lists the numbers of differentially expressed miRNAs after IA and PP isolation (Filter criteria: Base mean ≥ 100; log2FC ≥ 1 or log2FC ≤ -1; padj ≤ 0.1).

We analyzed the expression profiles of small RNAs in EVs from 2D and 3D cells by either IA or PP isolation with smallRNA-Seq. Differences were found when mapping reads to classes of small RNA. Increased frequencies of miRNAs were observed in 3D EVs, regardless of whether the IA or PP approach was used (**Figure 2A**). After mapping to miRBase, 1964 EVmiRNAs were detected, with at least one hit in each sample. Principal component analysis (PCA) of the differentially expressed miRNAs provided a clear separation between the two cultivation (2D/3D) and the two isolation methods (IA/PP) (**Figure 2B**). DESeq2 was applied to assess differential regulation of miRNA levels in EVs from 2D and 3D models.

We first examined GBM model-specific EV-miRNA profiles by a cell-line specific comparison (pairwise comparison) of 2D and 3D of each cell line to determine cell line-specific changes. To analyze significant miRNAs in 3D of the different cell lines, an Upset plot was constructed showing the number of unique and common differentially regulated miRNAs in six glioma models (**Figure 2C**). The number of all altered miRNAs changed with the cell model as follows: 54 miRNAs were differentially regulated in BTIC18, 28 in BTIC129, 22 in BTIC155, 19 in BTIC10, 15 in BTIC131, and 9 in BTIC13. There was not any altered miRNA found that was shared by all cell lines, but one miRNA, has-miR-4485-3p, was differentially regulated in five of six GBM models. Most shared miRNAs were found between BTIC18 and BTIC13. A detailed list of all significantly regulated miRNAs from the pairwise comparison and according to the following criteria (base mean ≥ 100, log2 fold change (log2FC) ≥ 1 or log2FC ≤ 1, adjusted p-value (padj) ≤ 0.01) can be found in the supplement (**Supplementary Data Sheet 3**).

To identify general 2D and 3D differences in miRNA expression, cell type specific bias was incorporated into the statistical model and an overall comparison including all samples was performed. Following the application of stringent filtering criteria (base mean ≥ 100, log2FC ≥ 1 or log2FC ≤ 1, padj ≤ 0.01), we found in total five (IA) and eleven (PP) miRNAs significantly regulated in EVs from primary GBM 3D models compared to 2D models. The different miRNA expression signatures of 2D and 3D EVs are shown in the volcano plot in **Figure 2D** for the IA method and **Figure 2E** for the PP method. Using the IA method, according to the DGE analysis, two miRNAs (miR-23a-3p, miR-2682-5p) were upregulated in 3D, while three miRNAs (miR-4485-3p, miR-1260b, miR-1260a) were downregulated. In EVs isolated by PP, two miRNAs were upregulated (miR-2682-5p, miR-1-3p) and nine miRNAs were downregulated (miR-7-5p, miR-370-3p, miR-323a-3p, miR-382-5p, miR-134-5p, miR-4485-3p, miR-92b-5p, miR-1260b, miR-1260a). Differentially regulated miRNAs in 3D with the corresponding base mean, log2FC and padj values are shown in **Figure 2F**. The number of differentially regulated miRNAs detected in DESeq2 analysis varied between isolation methods, but four of five miRNAs from the IA approach were also differentially regulated in the PP approach, resulting in a total number of twelve individual miRNAs (**Figure 2F**). Nine of twelve miRNAs were also significantly altered in the cell-line specific comparison and are reported in **Supplementary Data Sheet 3**. However, three miRNAs (miR-1-3p, miR-7-5p, and miR-92b-5p) were ruled out by the stringent filtering criteria, mainly related to the loss of statistical power by the pairwise comparison.

In addition, among the twelve, a group of four miRNAs (miR-370-3p, miR-323a-3p, miR-382-5p, miR-134-5p) located on chromosome 14 in the same genomic region (DLK1-DIO3 region) was identified (**Supplementary Data Sheet 4**).

### Overrepresentation analysis identifies biological pathways related to immune response, regulation of gene expression, and signal transduction

**Figure 3:**
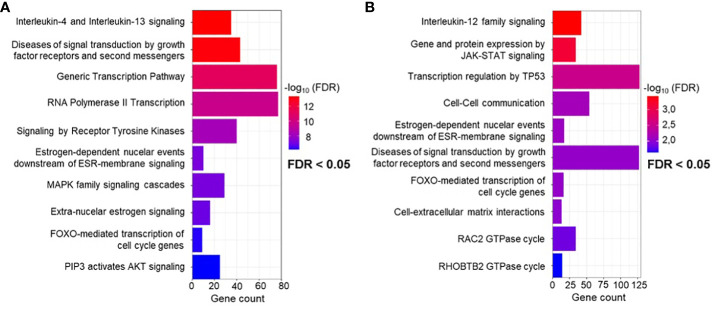
Reactome overrepresentation analysis. **(A)** Significant terms of pathways identified using miRNA-target interactions containing only experimentally validated miRNA-target interactions by qPCR, western blot and reporter assay, and **(B)** extended analysis by additional experimentally validated target interactions, including Microarray and NGS.

Based on our DGE analysis and overall comparison of miRNA expression, we decided to combine the differentially regulated miRNAs from both approaches (IA and PP) for further target and pathway analysis. Reactome pathway overrepresentation was performed for all twelve differentially regulated miRNAs in 3D and their targets, which were either identified with miRTarBase by strong experimental validation using qPCR, Western blot, and reporter assay or experimentally verified by additional methods, including microarray and NGS. Since a more rigorous miRNA target validation excludes four miRNAs (miR-2682-5p, miR-1260a, miR-92b-5p, miR-4485-3p) from our analysis as they have not yet been experimentally confirmed by qPCR, Western blot, or reporter assays, we also evaluated the second approach separately, with all twelve miRNAs included in the pathway analysis. The main canonical signaling pathways emerging from both approaches are related to the categories immune response, regulation of gene expression and signal transduction (**Figures 3A, B**). Among the most affected signaling pathways determined by the more stringent selection process of miRNA-target mRNAs, pathways regulating the immune response, such as interleukin-4 and 13 (IL-4, IL-13) signaling are particularly evident. In addition, many miRNA targets are affected, that indicate altered gene transcription in 3D models compared to 2D models (**Figure 3A**). Closer examination also reveals altered signal transduction mediated by receptor tyrosine kinases and estrogen-mediated signaling and their downstream elements such as MAPK and PIP3/AKT. In addition, FOXO-mediated transcription is altered, which is associated with the regulation of the expression of multiple cell cycle genes such as cyclin-dependent kinase (CDK) inhibitor CDKN1A (p21Cip1) (35, 36).

Overall, miRNA target determination enhanced by NGS and microarray validation shows very similar alteration of transcription and signal transduction by the same targeted pathways (**Figure 3B**). Similarly, key canonical pathways include immunoregulatory pathways, but this time focusing on interleukin-12 (IL-12) signaling. In addition, pathway analysis encompassing all twelve miRNAs predicts a cell stress response regulated by TP53. Finally, affected pathways related to cell-cell communication and interaction with the extracellular matrix are also shown. A detailed table of all significant terms of the pathway analysis with precise FDR value information can be found in the supplement **Supplementary Data Sheets 5, 6**.

### The proteome of the 3D cell culture media and EV cargo is associated with crucial tumor signaling pathways

**Figure 4:**
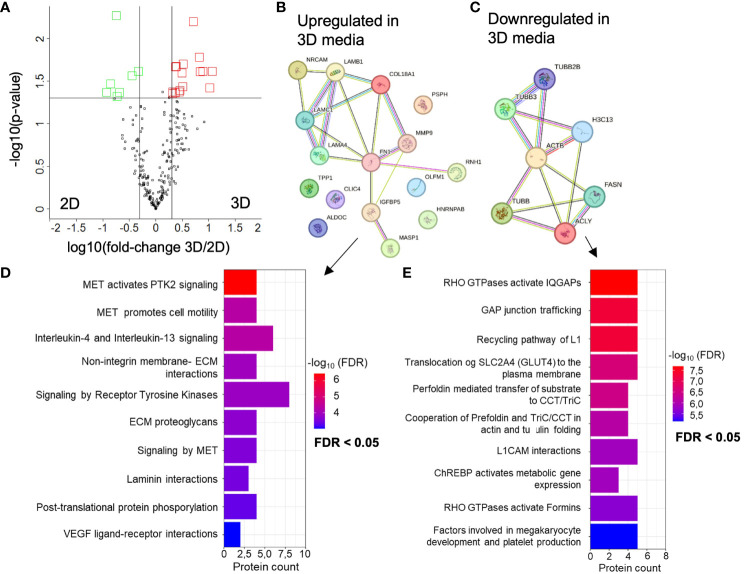
Altered proteomes of BTIC-18 3D versus 2D cell culture medium. **(A)** Volcano plot showing differentially abundant proteins from 3D versus 2D cell culture medium. Black line represents significant cut offs (t-test p-value multiple testing corrected < 0.05; -2 > fold change 3D/2D < 2). All experiments were conducted in 6 time points. Proteins highlighted in red were significantly higher concentrated in 3D cell culture medium. Proteins highlighted in green were significantly higher concentrated in 2D cell culture medium. **(B, C)** Interaction analysis of up- and downregulated proteins in 3D using STRING database (string-db.org); The line color indicates the type of interaction evidence, with purple and light blue lines denoting known, experimentally validated or database-derived interactions and other colors defining predicted interactions. The line thickness indicates the strength of the data support. **(D)** Significant terms of the Reactome overrepresentation analysis of increased and **(E)** decreased proteins in 3D media.

First, we analyzed the protein composition derived from 6 time points of 2D and 3D GBM cell media of BTIC18 using LC-MS/MS-based proteomics. We detected an average of 157 proteins ± 51 (standard deviations) in the 2D GBM cell media and 211 ± 186 proteins in the 3D GBM cell culture media (n=6). To identify significantly differentially expressed proteins in the cell culture media from 2D and 3D cultures, we conduct pairwise comparisons using paired t-tests and apply Benjamini-Hochberg correction (FDR: 0.05). Applying filter criteria of fold-changes > 2 and p-values < 0.05, the analysis identified 16 upregulated proteins in 3D culture medium and seven downregulated. The corresponding volcano plot of all quantified proteins is shown in **Figure 4A**. A network of the up- (**Figure 4B**) and downregulated (**Figure 4C**) proteins in 3D was generated using STRING-db software (37–39). Increased and decreased proteins in 3D were further analyzed individually using Reactome pathway analysis (40, 41). For the increased proteins in 3D media the top canonical pathways were related to MET (Mesenchymal Epithelial Transition/tyrosine kinase) -associated signaling promoting cell motility, IL-4 and IL-13 signaling, extracellular matrix interactions and organization, and VEGF ligand-receptor interactions (**Figure 4D**). The decreased proteins in 3D medium influence signaling pathways that primarily affect the cytoskeleton and thus the dynamics of the cytoskeleton, i.e. their mobility and transport within and outside the cell (**Figure 4E**). A detailed table of all significant terms of the pathway analysis from upregulated (**Supplementary Data Sheet 7**) and downregulated (**Supplementary Data Sheet 8**) proteins in 3D media with precise FDR value information can be found in the supplement.

**Figure 5:**
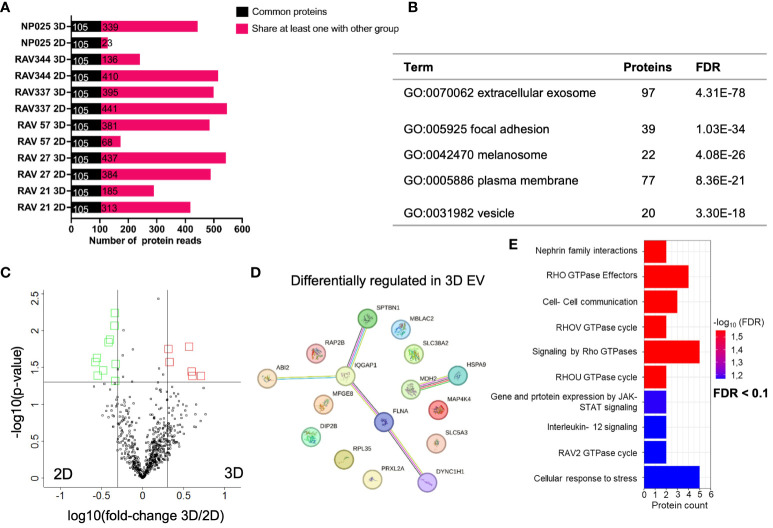
Comparative proteome analysis of immunoaffinity purified EVs from six 3D versus 2D samples. **(A)** Number of proteins detected in 2D and 3D EV preparations in cell lines derived from six different patients (n=6). Common proteins across 12 samples are displayed in black, while shared proteins present in at least one group are indicated in pink. **(B)** TOP 5 GO terms from DAVID cellular component analysis (https://david.ncifcrf.gov/tools.jsp) using all 105 common proteins from 2D and 3D EVs. **(C)** Volcano plot showing differentially abundant proteins from 3D versus 2D cell culture medium. Black line represents significant cut offs (t-test p-value multiple testing corrected < 0.05; -2 > fold change 3D/2D < 2). Proteins highlighted in red were significantly higher concentrated in 3D EVs. Proteins highlighted in green were significantly higher concentrated in 2D EVs **(D)** Interaction analysis of up- and downregulated proteins in 3D using STRING database (string-db.org); The line color indicates the type of interaction evidence, with purple and light blue lines denoting known, experimentally validated or database-derived interactions and other colors defining predicted interactions. The line thickness indicates the strength of the data support. **(E)** Significant terms of the Reactome overrepresentation analysis of all significantly regulated proteins in 3D EVs.

In a next experiment, we analyzed the EV proteome composition of six 2D and 3D GBM models by performing immunoaffinity (IA) purification of EVs followed by LC-MS/MS-based proteomics. We opted for EVs isolated by IA because of the known protein aggregate as co-precipitates present in PP isolates. We identified an average of 423 proteins ± 182 in 2D EVs and 462 proteins ± 123 in 3D EVs. 105 proteins (**Supplementary Data Sheet 9**) were identified as common proteins in all 2D and 3D EV samples, as shown in **Figure 5A**. Interestingly, GO analysis of cellular components showed that 97 of the 105 common proteins were associated with extracellular exosomes (GO:0070062) (**Figure 5B**), which strongly supports the use of an immunoaffinity enrichment targeting the EV marker proteins CD81, CD63, and CD9 for proteomic analysis. Applying the filter criteria of fold-changes > 2 and p-values < 0.05, the analysis identified 16 (6 up- and 10 down) differentially regulated proteins in 3D versus 2D EVs with the corresponding volcano plot shown in **Figure 5C**. A network of differentially regulated proteins was generated using STRING-db software (37–39) (**Figure 5D**). Moreover, Reactome pathway analysis (40, 41) overrepresentation analysis showed that signaling pathways were altered that mainly affect GTPases. In addition, signaling pathways related to cell-cell communication, immune system regulation (IL-12), and stress response were altered (**Figure 5E**). A detailed table of all significant terms of the pathway analysis in 3D EVs with precise FDR value information can be found in the supplement (**Supplementary Data Sheet 10**).

## Discussion

Conventional GBM *in vitro* cell cultures select towards a homogeneous phenotype and therefore do not adequately reflect the *in vivo* situation (9). However, the amount and composition of EVs secreted by tumor cells is largely determined by the interplay between different tumor cell types and the immediate and distant TME (7, 42). To our knowledge, many GBM studies investigating the role of EVs derive them usually from conventional cell cultures (2D). Here we compared EVs derived from different GBM culture conditions (2D versus 3D) and investigated the amount and the composition (miRNA and protein load) of these isolated EVs. The verification of EV purification under MISEV guidelines is an essential issue, so we used standard methods such as NTA, TEM, and flow cytometry to validate the characteristics of EVs. When performing NTA, we observed differences in particle size and concentrations depending on the chosen EV purification method. As EV yield could correlate with cell number we used a proteomic analysis approach to normalize cell numbers across the different BTIC18 culture conditions and found that 3D released a much higher amount of EVs compared to corresponding 2D cultures. These observations are consistent with previous studies showing that 3D culture systems of tumor cells yield a higher number of EVs compared to 2D monocultures (43–45), and that the number of cells used to produce EVs from 3D cultures is significantly lower than from 2D (43). Thus, 3D culture systems may represent more useful models for mimicking the physiological environment *in vivo*, as they more closely resemble the environment generated by patient tumors, consistent with recent reports (43, 45, 46).

In a next step, we investigated the composition of the isolated EVs from 2D and 3D cultures. With a smallRNA-Seq approach we identified interesting differences between the miRNA cargo secreted 2D and 3D cultured cells. EVs from 3D cells were characterized by high loads of different miRNAs. This is due to either more EVs being secreted from the 3D organoid model or more miRNAs being packed into the EVs of the 3D culture system. With a differential gene expression analysis (DGE, filter criteria: base mean ≥ 100, log2FC ≥ 1 or log2FC ≤ 1, padj ≤ 0.01), were found that the sets of miRNAs are different. Indicating that the culture condition has a tremendous impact not only on the tumor cells but also on the “information” that is shared with the surrounding TME. Overlap analysis revealed differences in sets of miRNA for 2D and 3D cultures also dependent on the isolation method. We found for the IA method five miRNAs and for the PP method eleven differentially expressed miRNAs. Most miRNAs appeared similarly altered irrespective of the EV isolation method (IA or PP), however one miRNA, miR-23a-3p, appeared specifically in IA isolated EVs. This miRNA is reported to be frequently upregulated and involved in GBM invasion (47–49), proliferation and migration (49). This could indicate that 3D cultured tumor cells share a profile/phenotype with the GBM *in vivo* condition. In addition, miR-7-5p, which is associated with suppression of mTOR signaling in GBM, was also found downregulated in 3D cultured cells (50). This again could reflect a more aggressive nature of 3D cultured models. Furthermore, other miRNAs (miR-7-5p, miR-2682-5p, miR-323a-3p, miR-382-5p, miR-1260a, miR-370-3p, miR-92b-5p and miR-23a-3p) found regulated in 3D cultures were reported for GBM thus highlighting an alignment of 3D cultures with GBM *in vivo* conditions compared to corresponding 2D cultures (48, 50–59), with miR-7-5p, miR-2686-5p, and miR-323a-3p detected as EV-miRNAs from patient derived- GBM cells and serum (50, 59, 60). Notably, four of the twelve significantly regulated miRNAs (miR-323a-3p, miR-382-5p, miR-370-3p, miR-134-5p), are located within the DLK1-DIO3 domain, which has been identified as a cancer-associated genomic region on the human chromosome 14 (14q32) which harbors in total 53 miRNAs. Many of these miRNAs are differentially expressed in a variety of cancers (61). miR-370-3p has been frequently found to be decreased in glioma tissues, particularly in recurrent GBM (56), and was also found downregulated in 3D EVs in our analysis. miR-370-3p has been shown to inhibit GBM cell proliferation by targeting β-catenin and causing cell cycle arrest (62). Similarly, miR-382-5p was decreased in 3D models and was previously found to be downregulated in GBM compared to normal brain tissue (61). The data so far indicates that the DLK1-DIO3 domain harbors a large number of miRNAs, which are reported to increase GBM aggressive phenotype and are also altered in 3D cultures. However, more detailed analysis also with respect to potential changes induced in the TME are needed to better understand the interactions. In particular, *in vitro* functional analysis of these miRNAs or miRNA profiling of GBM patients would add valuable data to better understand their role in GBM but exceeds the scope of this study.

However, we performed an overrepresentation analysis to assess which signaling pathways are regulated by the differentially regulated miRNAs shuttled in 3D-EVs. Recently, Braun et al. showed that 3D GBM models based on the same tumor specimens exhibited significantly altered mRNA expression patterns compared to corresponding 2D models. Single-cell sequencing also revealed changes in immunoregulatory signaling pathways, such as IL-4 and IL-13 (11). Interestingly, our smallRNA-Seq supports this finding, as the miRNA cargo in 3D cultures are associated with the IL-4 and IL-13 cytokine signaling pathway. Moreover, this signaling pathway is also altered in our 3D conditioned media when comparing to 2D GBM cell culture supernatant. Produced by multiple components in the TME (63), IL-4 and IL-13 and their receptor systems are able to influence malignant behavior (64), via altering tumor proliferation, survival, and metastatic ability or suppressing tumor-directed immune surveillance mechanisms (64–66). A hallmark of tumor progression is the inflammatory response in the TME with an accumulation of macrophages (67). It is already known that IL-4 and IL-13 can also induce macrophages to polarize into an M2-like phenotype, which corresponds to the immunosuppressive phenotype (68). Gabrusiewicz et al. also demonstrated that exosomes from GBM stem cells reorganize the actin cytoskeleton after uptake into macrophages, resulting in a shift toward the immunosuppressive M2 phenotype (69). This highlights that 3D GBM cultures display several key phenotypes of *in vivo* GBM making this type of culture condition ideal for further investigations targeting immunosuppressive features of GBM or aspects of GBM progression. Such studies are not possible with 2D cultures, as these cells just do not possess similar phenotypes/profiles. Further pathway analysis revealed that the differentially regulated miRNAs are involved in the regulation of gene expression and signal transduction mediated by receptor tyrosine kinases and estrogen-mediated signals as well as their downstream elements such as MAPK and PIP3/AKT. These signals mainly play a role in altered gene expression leading to aberrant cell proliferation, apoptosis, survival, migration and invasion in GBM (70). The increased yield of miRNA targets by additional validation by NGS and microarray led to altered immunoregulatory signaling pathways, focusing this time on IL-12, which is also an important contributor to an effective anti-tumor immune response (71), and has been studied as a potential immunotherapeutic agent for GBM (72). Moreover, cell stress response regulated by TP53 is changed in 3D EVs from GBM models, with TP53 involved in GBM cellular processes such as cell cycle arrest, apoptosis, DNA repair, and cellular senescence (73). Since in 3D organoids, cells in the core of the cell aggregate may have reduced access to essential nutrients, hypoxia and nutrient deficiency might trigger stress responses (74). Evidence of this was also provided in a recent characterization of the used 3D models by Braun et al. describing core region with necrotic and damaged cells, similar to tumors from GBM patients (11). In addition to the necrotic areas of these complex models, it was observed that an outer layer with proliferating cells circumscribed the organoid. These are features also found in tumors of brain cancer patients, indicating that 3D models can mimic critical aspects of tumor growth/development. The extended time in culture needed for these models to form, reduces the influence originating from the use of Matrigel. However, of course more detailed studies are needed to better understand the impact Matrigel might have and if alternative approaches would lead to similar results.

Relatively few studies have been performed with MS-based proteomics of 2D and 3D cultured cells, and even only one study on GBM (75). These studies report significant differences in the proteomes of 2D and 3D cultured cells (75, 76). In our study, the proteomic analysis of GBM model supernatant also showed altered protein composition in 3D with completely different signaling pathways between upregulated and downregulated proteins in BTIC18 3D-conditioned media. Whereas the increased proteins in the media are mainly related to cell motility, immune regulation, and extracellular matrix interactions and organization, the less abundant proteins in 3D (increased proteins in 2D) involve signaling pathways that mainly affect the cytoskeleton, clearly indicating a greater complexity of the TME in the 3D organoid model. Comparing the protein composition of 2D EVs with 3D EVs from GBM models, we identified proteins that are differentially regulated in 3D and are associated with IL-12 signaling, a process that regulates various aspects of the immune response. IL-12 signaling was altered in the protein cargo of 3D-EVs, as demonstrated by the detection of miRNA profiles by smallRNA-Seq. previously also in this study. GBM tumors are reported to form immunosuppressive TME thus impeding immune response (77), altered IL-12 signaling again aligns 3D cultured cells in their specific phenotypes with those of GBM. Moreover, Rho-GTPase signaling pathways and cell-cell communication were altered in EVs from 3D organoids. Rho-GTPases play a critical role in regulating various cellular processes and have been identified as key regulators of GBM cell invasion and migration (78). Thus, RHO GTPase signaling in recipient cells may play a central role in regulating processes such as cytoskeletal dynamics, cell migration, adhesion, invasion, and cell-cell interactions – all functions associated with the TME.

In our data, cell line-specific comparison showed that the miRNA and protein expression patterns of EVs were quite different between 2D and 3D cultured cells but also specific for each cell line. This might be due to the small sample size of six 2D and 3D GBM cultures respectively and varying GBM tumor subtypes. Further reason could be the large heterogeneity of each GBM cell sample, which extends to the molecular level, including miRNA expression (79), and may also be reflected in their protein profile (80). Furthermore, the most downregulated miRNA (miR-4485-3p), found in 3D and similarly decreased after IA and PP EV isolation, hasn’t previously been linked to GBM, but miRNA mimic miR-4485-3p reduces proliferation of MDA-MB-231 breast cancer cells (81). Despite being the only miRNA significantly downregulated in 5 of 6 3D GBM cell lines, there is limited information and only few functional experiments on this miRNA available, especially in relation to cancer progression, with the exception of evidence for a mitochondrial origin of miR-4485-3p (82, 83). Regarding the size determination after IA isolation, one has to keep in mind that the measurement is affected by the magnetic beads (size 30 to 80 nm) attached to the EVs.

## Conclusions

Proceeding from the finding by Braun et al. that 3D tumor organoids have a pronounced effect on the mRNA profile of GBM models compared with conventional 2D culture conditions (11), we proved in this study that EVs derived from 3D models show a different miRNA and protein profile compared to conventional 2D cell cultures Remarkably, particular immunoregulatory pathway are targeted by EV delivered biomolecules. This suggests that aspects of the immunosuppressive phenotype of GBM tumor cells could be modeled by 3D culturing of GBM cells. The role of EVs in shaping of the TME further could be a promising target for new therapeutic strategies. Although conditions for successful therapy remain challenging due to the dramatic heterogeneity of malignant gliomas at genetic and immunological levels (84), the observed results highlight the potential of 3D models for the development and testing of immune cell-based therapeutic approaches. In addition, we discovered several upregulated EV- associated miRNAs, some of which had never been linked with GBM before. This demonstrates the utility of including EV-miRNAs in disease assessment of GBM. However, further sampling of EV-associated miRNAs in cerebrospinal fluid liquid biopsy would greatly improve biomarker research in GBM.

## Data availability statement

The datasets presented in this study can be found in online repositories. The names of the repository/repositories and accession number(s) can be found below: NGS datasets generated and/or analyzed during the current study are available in the ENA repository, accession number PRJEB70714. The mass spectrometric raw files as well as the MaxQuant output files have been deposited to the ProteomeXchange Consortium via the PRIDE partner repository and can be accessed using the identifier PXD047328.

## Ethics statement

The studies involving humans were approved by Ethics Committee of the University Regensburg (No° 18-207-101 and No° 21-2393_1-101) and University Hospital Regensburg (No° 19-1454-101). The studies were conducted in accordance with the local legislation and institutional requirements. The participants provided their written informed consent to participate in this study.

## Author contributions

MS: Data curation, Investigation, Project administration, Writing – original draft. FB: Data curation, Methodology, Writing – review & editing. DC: Data curation, Investigation, Software, Writing – original draft. CL: Resources, Supervision, Writing – review & editing. CM: Data curation, Software, Writing – original draft. CG: Data curation, Methodology, Writing – original draft. BK: Formal analysis, Software, Visualization, Writing – review & editing. MP: Resources, Writing – review & editing. PH: Methodology, Resources, Writing – review & editing. OS: Resources, Supervision, Writing – review & editing. MWP: Conceptualization, Resources, Supervision, Writing – review & editing. MJR: Conceptualization, Resources, Supervision, Writing – review & editing. MR: Conceptualization, Funding acquisition, Methodology, Project administration, Writing – review & editing.

## Supplementary material

SUPPLEMENTARY DATA SHEET 1 Consistent proteins of six time points from BTIC-18 media. List of consistent proteins from all six time points in 2D (77 proteins) and 3D (3 proteins) determined by proteomic analysis of the cell culture media of BTIC-18.

SUPPLEMENTARY DATA SHEET 2 Gating dot plots. dot plots of flow cytometry for surface and intracellular markers.

SUPPLEMENTARY DATA SHEET 3 Differentially regulated miRNAs from the pairwise comparison. List of all differentially regulated miRNAs from pairwise comparison based on the filter criteria: Base mean ≥ 100; log2FC ≥ 1 or log2FC ≤ 1; padj ≤ 0.1. The significant miRNAs from our overall comparison are highlighted in green.

SUPPLEMENTARY DATA SHEET 4 Twelve significantly regulated miRNAs and their genomic location. List of all significantly regulated miRNAs, their genomic location, sequence, and number of targets found in miRBase. A group of miRNAs (hsa-miR-370-3p, hsa-miR-323a-3p, hsa-miR-382-5p, hsa-miR-134-5p) located on chromosome 14 in the same genomic region (DLK1-DIO3 region) were highlighted in green.

SUPPLEMENTARY DATA SHEET 5 Reactome overrepresentation analysis of miRNA-targets interactions with strong evidence. Full list of significant terms of signaling pathways identified by Reactome overrepresentation analysis using miRNA-target interactions validated by qPCR, WesternBlot, and reporter assay.

SUPPLEMENTARY DATA SHEET 6 Reactome overrepresentation analysis of miRNA-targets interactions with supporting evidence by microarray and NGS. Full list of significant terms of signaling pathways identified by Reactome overrepresentation analysis using miRNA-target interactions validated by microarray and NGS assays.

SUPPLEMENTARY DATA SHEET 7 Reactome overrepresentation analysis of upregulated proteins in 3D BTIC-18 media. Full list of significant terms of the Reactome overrepresentation analysis of increased proteins in 3D media (n=6).

SUPPLEMENTARY DATA SHEET 8 Reactome overrepresentation analysis of downregulated proteins in 3D BTIC-18 media. Full list of significant terms of the Reactome overrepresentation analysis of decreased proteins in 3D media (n=6).

SUPPLEMENTARY DATA SHEET 9 Reactome overrepresentation analysis of significant proteins in 3D EVs. Full list of significant terms of the Reactome overrepresentation analysis of all significantly regulated proteins in 3D EVs isolated by IA (n=6).

SUPPLEMENTARY DATA SHEET 10 Common proteins of 2D and 3D EV proteomics. List of all common proteins from 2D and 3D EVs from proteomic analysis of EVs isolated by IA.
