# Supplementary material for: Extracellular vesicles secreted by 3D tumor organoids are enriched for immune regulatory signaling biomolecules compared to conventional 2D glioblastoma cell systems
Source: Front Immunol. 2024 Apr 25;15:1388769. doi: 10.3389/fimmu.2024.1388769 (PMC11079215; doi:10.3389/fimmu.2024.1388769)
Supplement: SUPPLEMENTARY DATA SHEET 2 — Gating dot plots. dot plots of flow cytometry for surface and intracellular markers. [file DataSheet_2.pdf]

PP EV surface marker staining

Cell line name in the manuscript	Cell line name in the gating dot plot
BTIC10	21
BTIC13	27
BTIC131	344
BTIC18	57
BTIC129	337
BTIC155	NP25

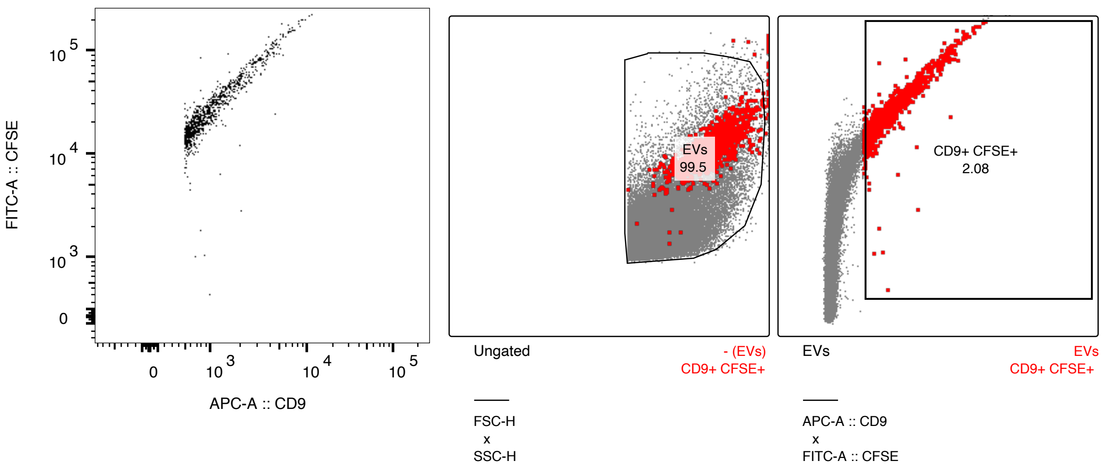

	Sample Name	Freq. of EVs
■	Specimen_001_21-2D (EV staining)_001.fcs	2.08

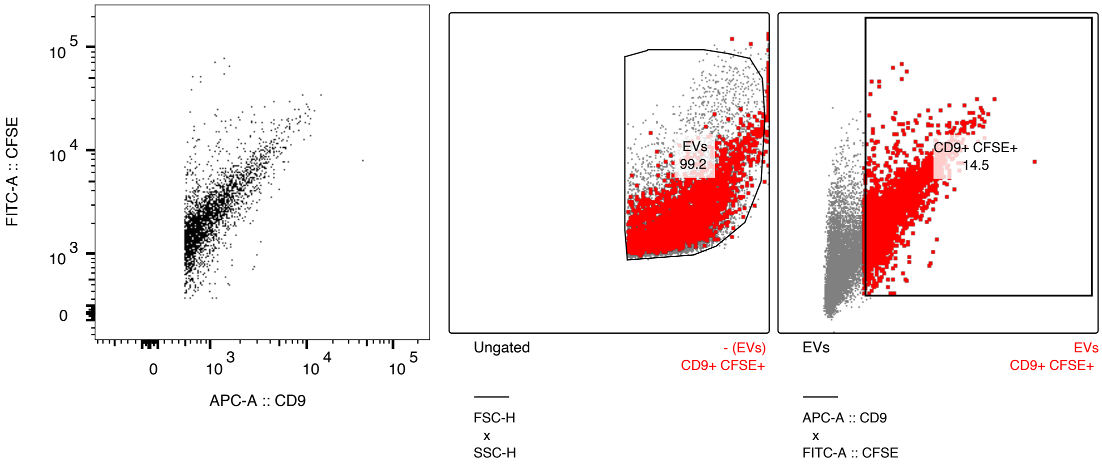

	Sample Name	Freq. of EVs
■	Specimen_001_337-2D (EV staining)_009.fcs	14.5

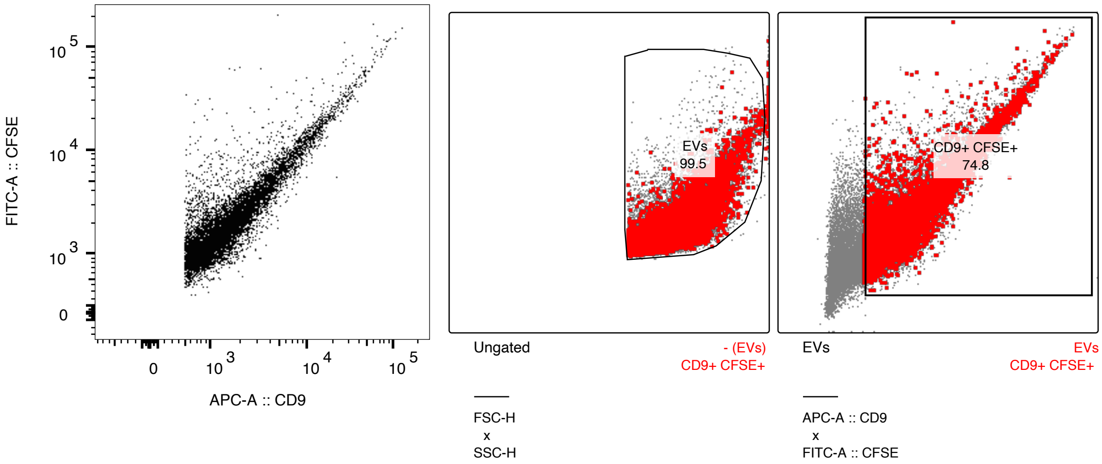

	Sample Name	Freq. of EVs
■	Specimen_001_57-2D (EV staining)_017.fcs	74.8

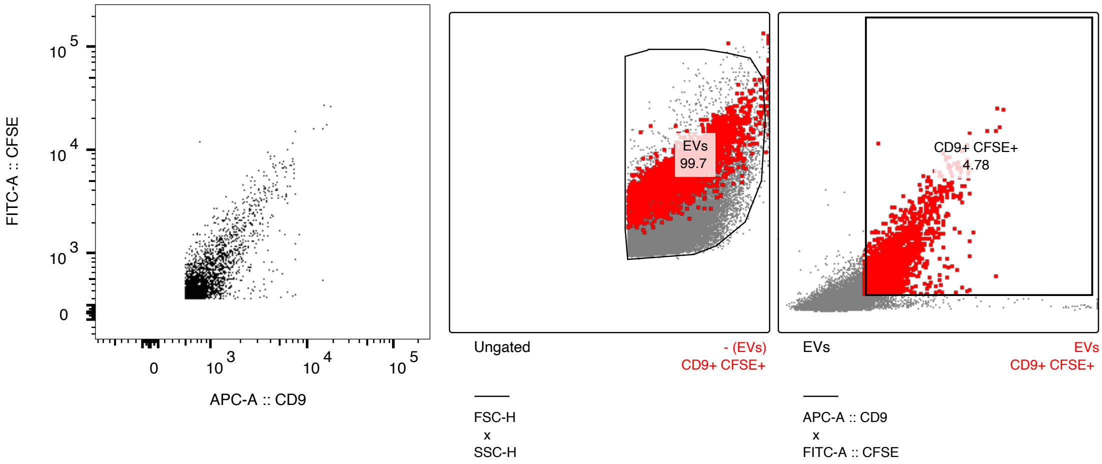

	Sample Name	Freq. of EVs
■	Specimen_001_21-2D (IgG staining)_002.fcs	4.78

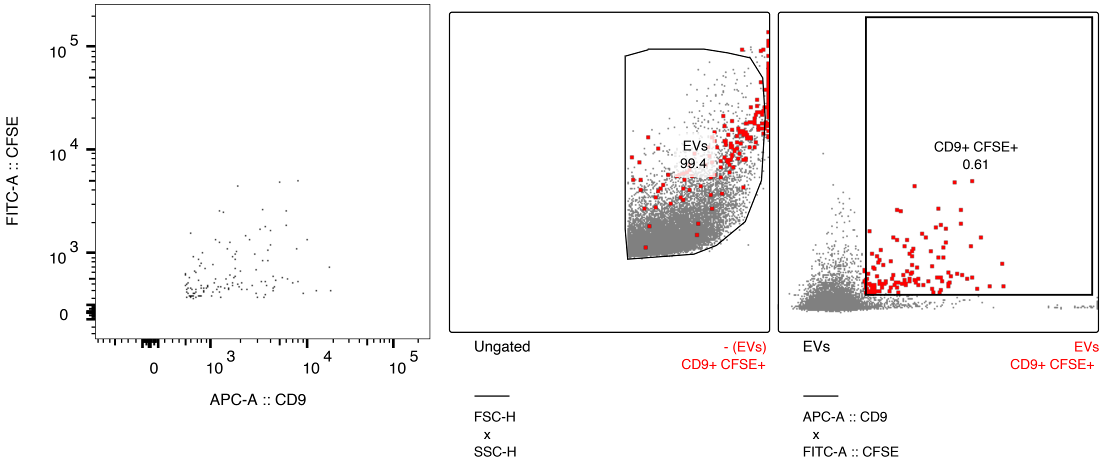

	Sample Name	Freq. of EVs
■	Specimen_001_337-2D (IGG staining)_010.fcs	0.61

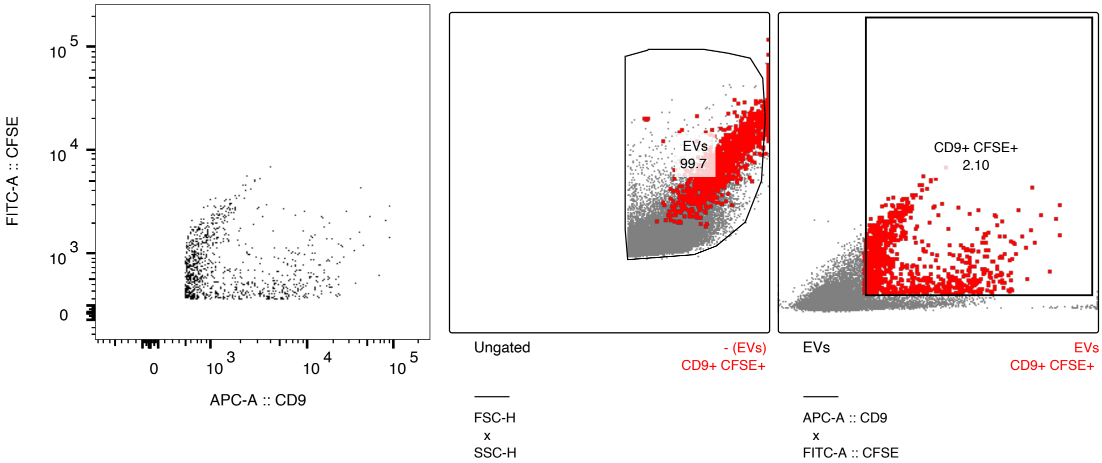

	Sample Name	Freq. of EVs
■	Specimen_001_57-2D (IGG staining)_018.fcs	2.10

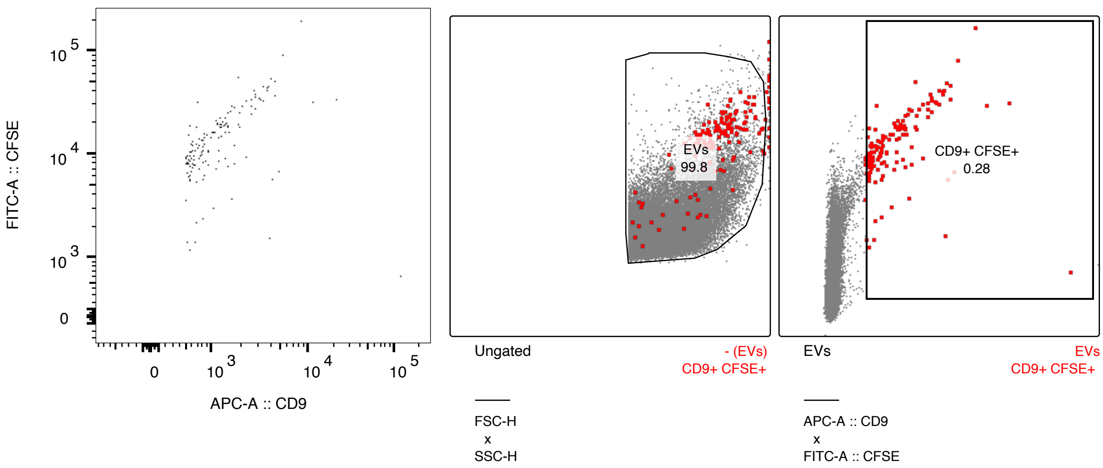

	Sample Name	Freq. of EVs
■	Specimen_001_21-3D (EV staining)_003.fcs	0.28

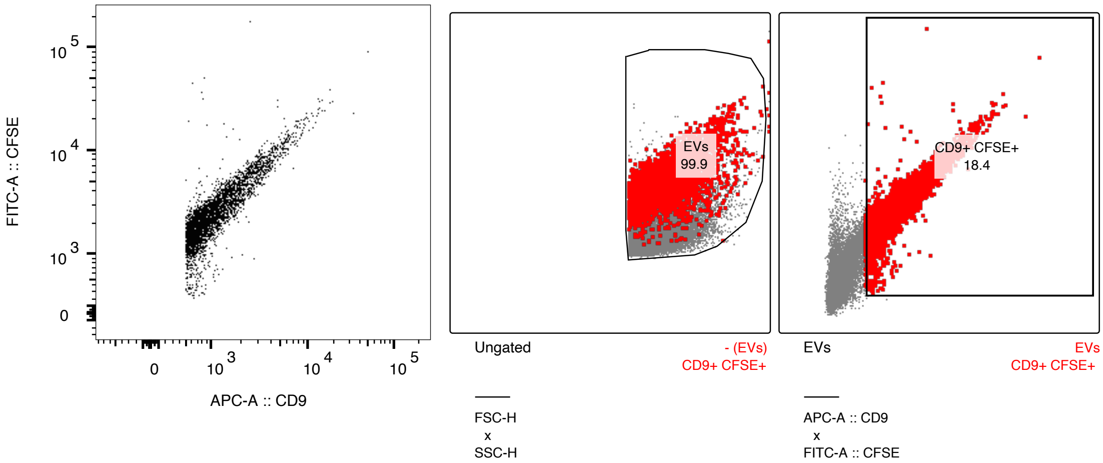

	Sample Name	Freq. of EVs
■	Specimen_001_337-3D (EV staining)_011.fcs	18.4

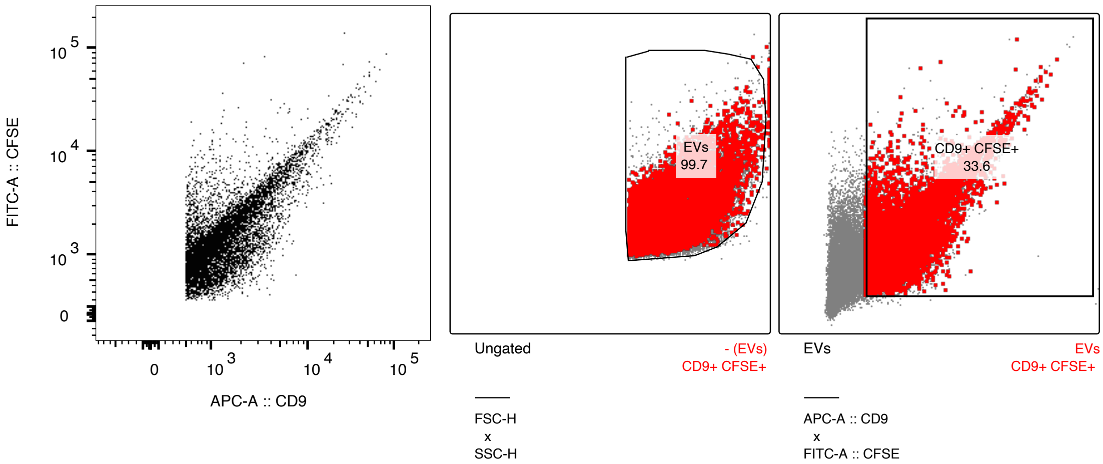

	Sample Name	Freq. of EVs
■	Specimen_001_57-3D (EV staining)_019.fcs	33.6

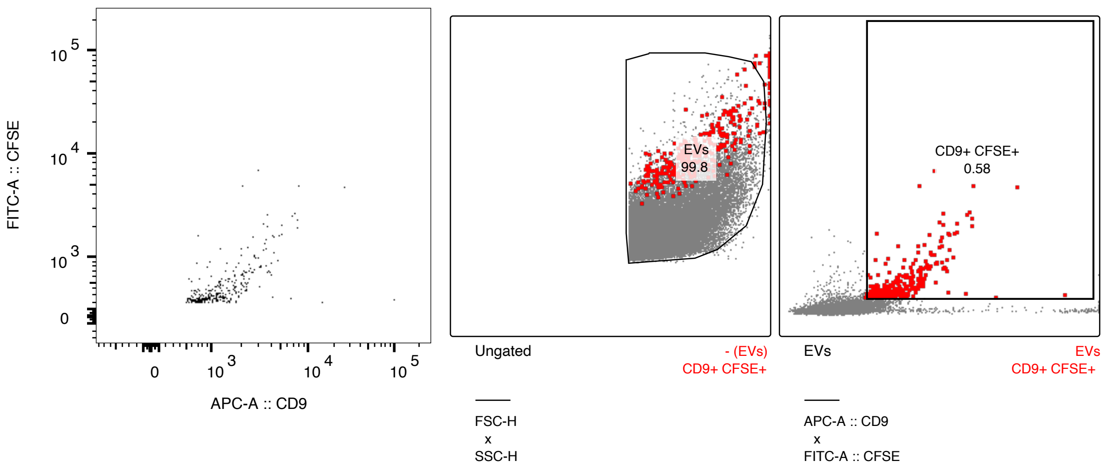

	Sample Name	Freq. of EVs
■	Specimen_001_21-3D (IgG staining)_004.fcs	0.58

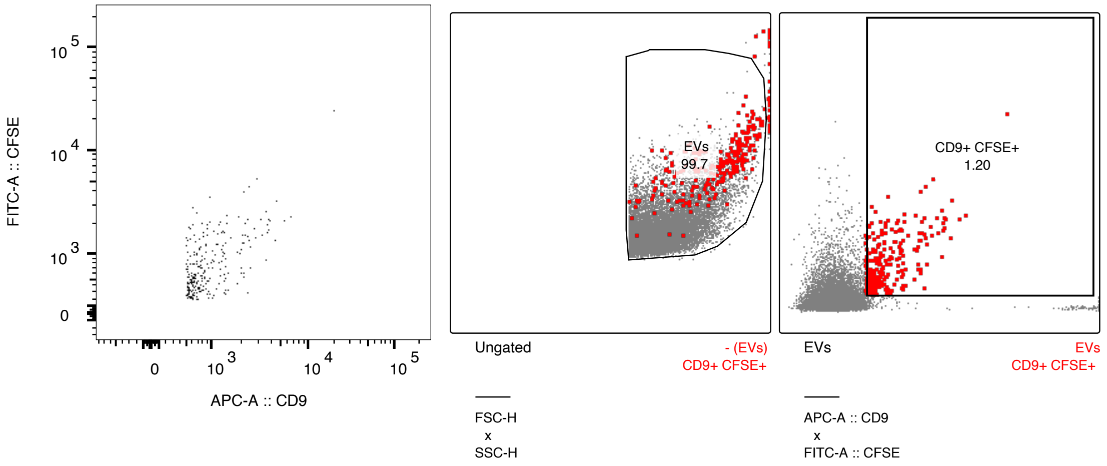

	Sample Name	Freq. of EVs
■	Specimen_001_337-3D (IGG staining)_012.fcs	1.20

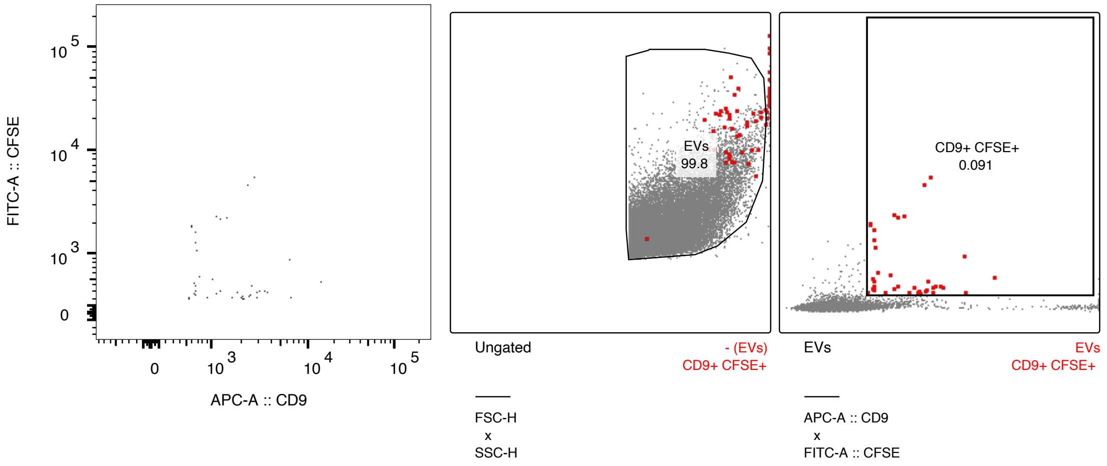

	Sample Name	Freq. of EVs
■	Specimen_001_57-3D (IGG staining)_020.fcs	0.091

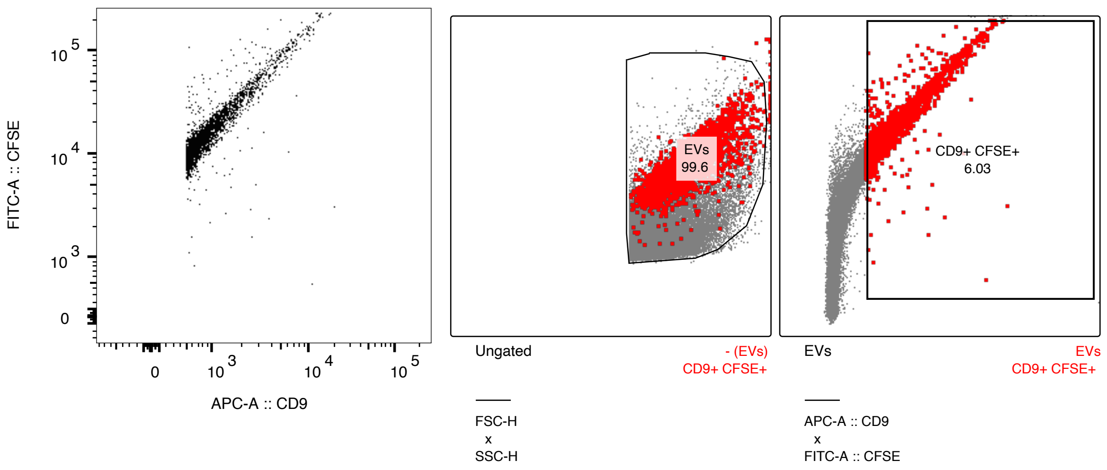

	Sample Name	Freq. of EVs
■	Specimen_001_27-2D (EV staining)_005.fcs	6.03

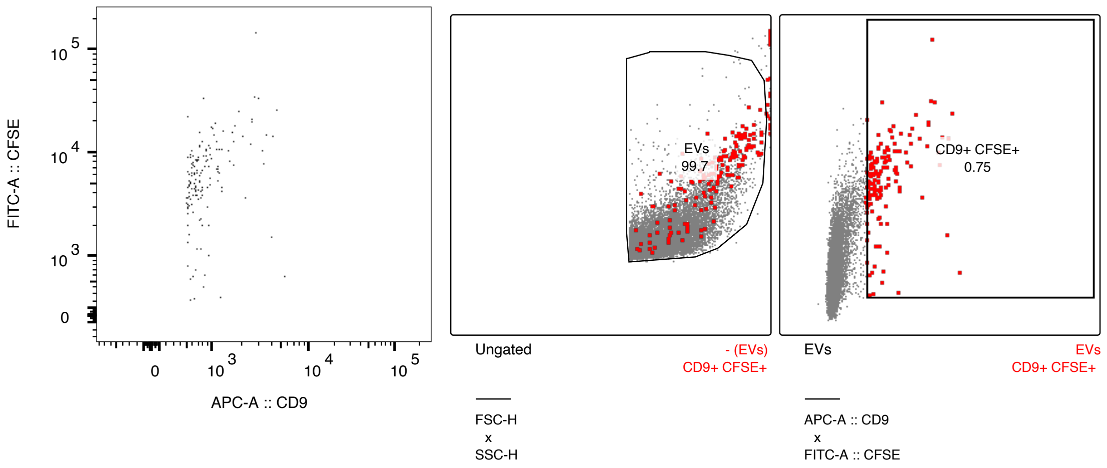

	Sample Name	Freq. of EVs
■	Specimen_001_344-2D (EV staining)_013.fcs	0.75

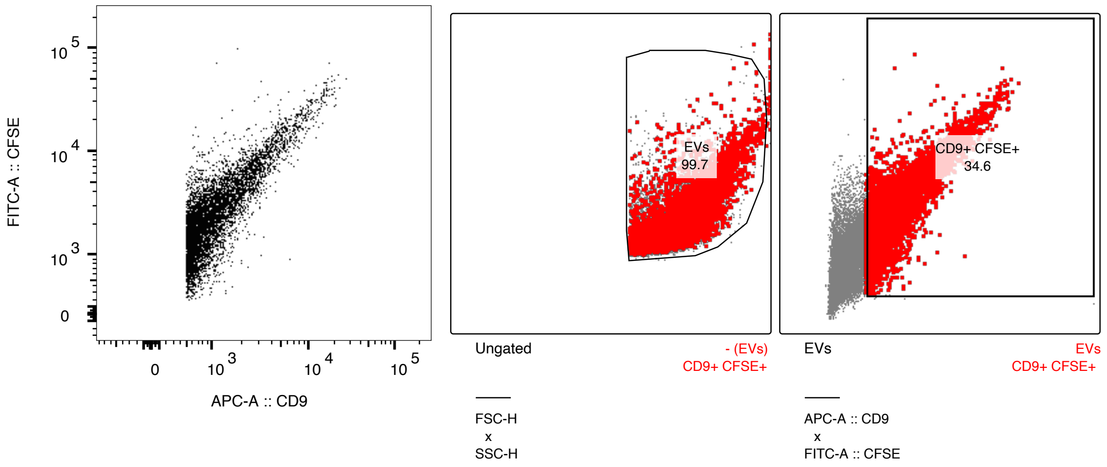

	Sample Name	Freq. of EVs
■	Specimen_001_NP25-2D (EV staining)_021.fcs	34.6

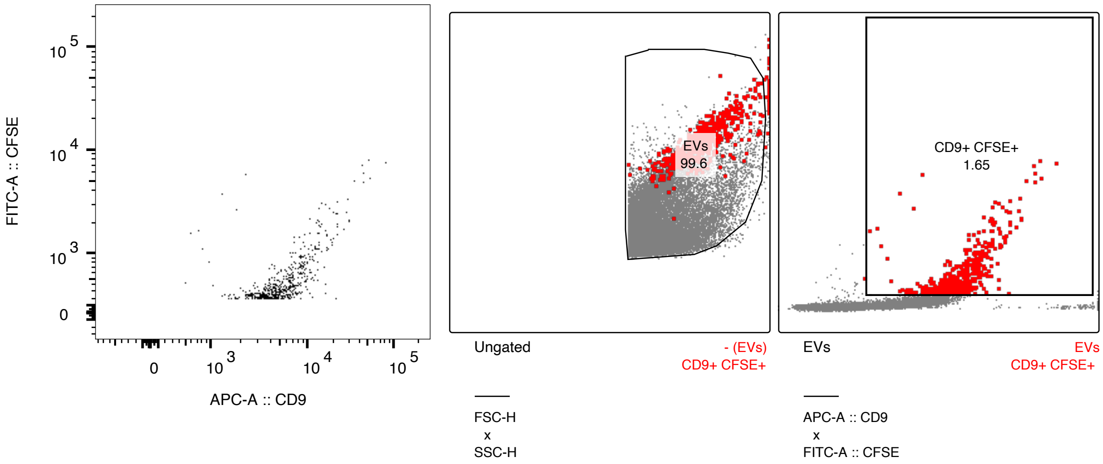

	Sample Name	Freq. of EVs
■	Specimen_001_27-2D (IgG staining)_006.fcs	1.65

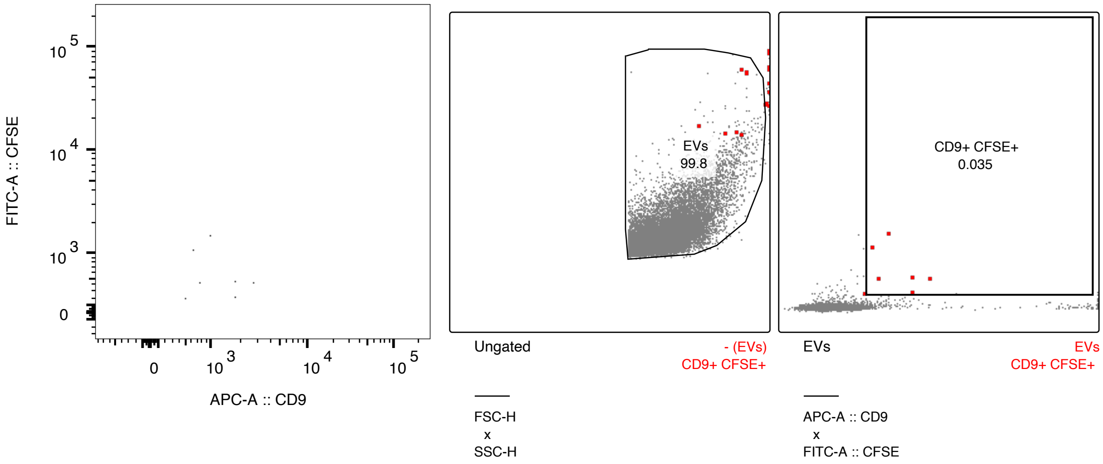

	Sample Name	Freq. of EVs
■	Specimen_001_344-2D (IGG staining)_014.fcs	0.035

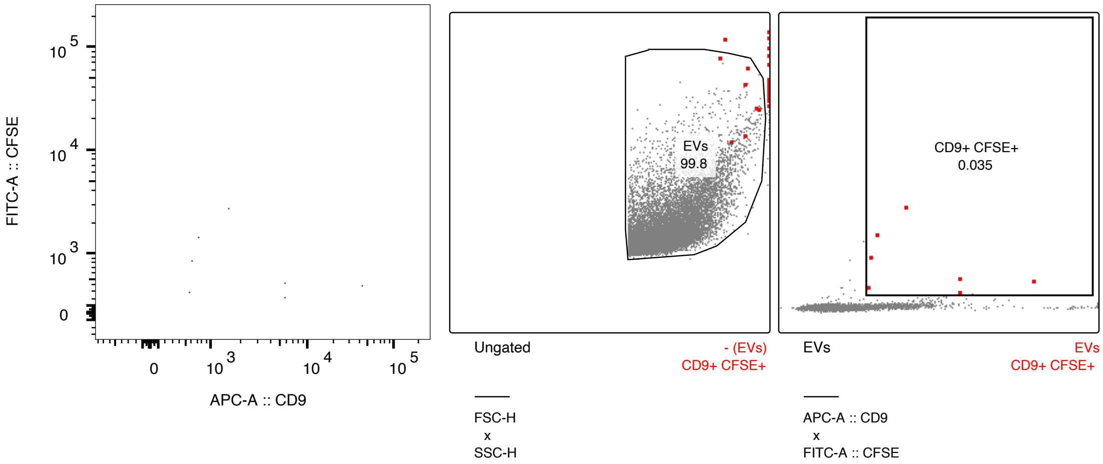

	Sample Name	Freq. of EVs
■	Specimen_001_NP25-2D (IGG staining)_022.fcs	0.035

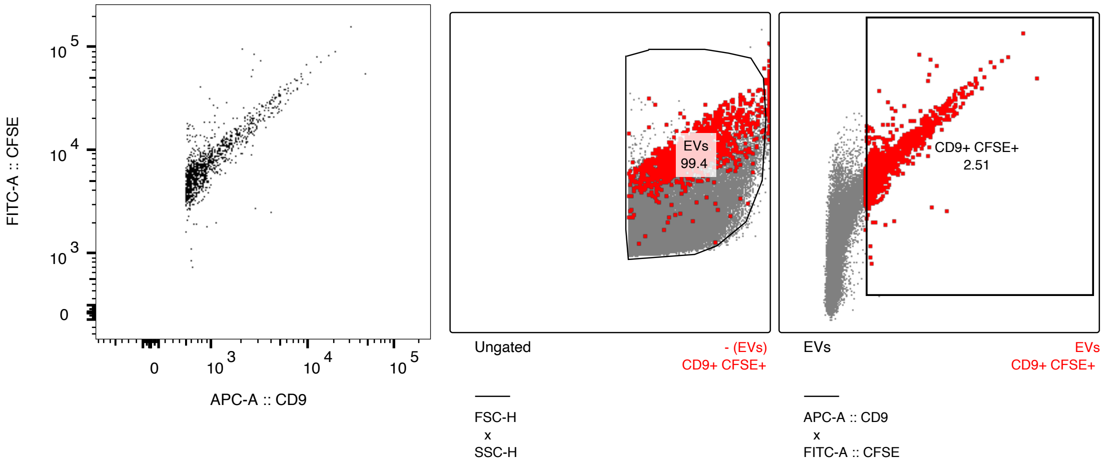

	Sample Name	Freq. of EVs
■	Specimen_001_27-3D (EV staining)_007.fcs	2.51

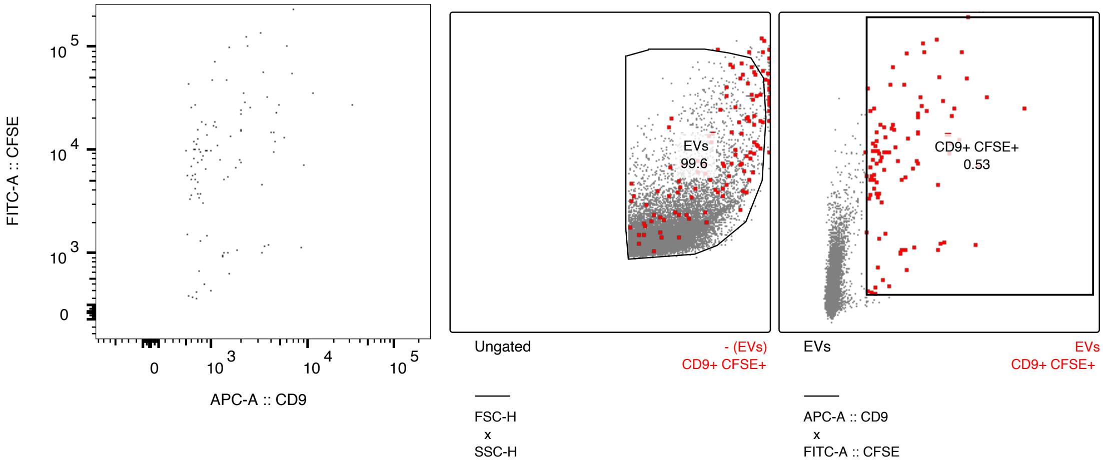

	Sample Name	Freq. of EVs
■	Specimen_001_344-3D (EV staining)_015.fcs	0.53

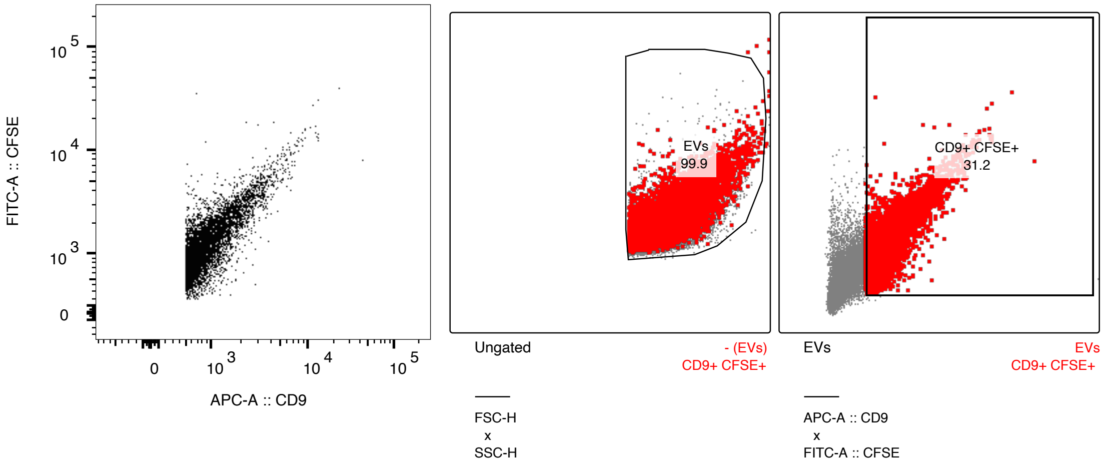

	Sample Name	Freq. of EVs
■	Specimen_001_NP25-3D (EV staining)_023.fcs	31.2

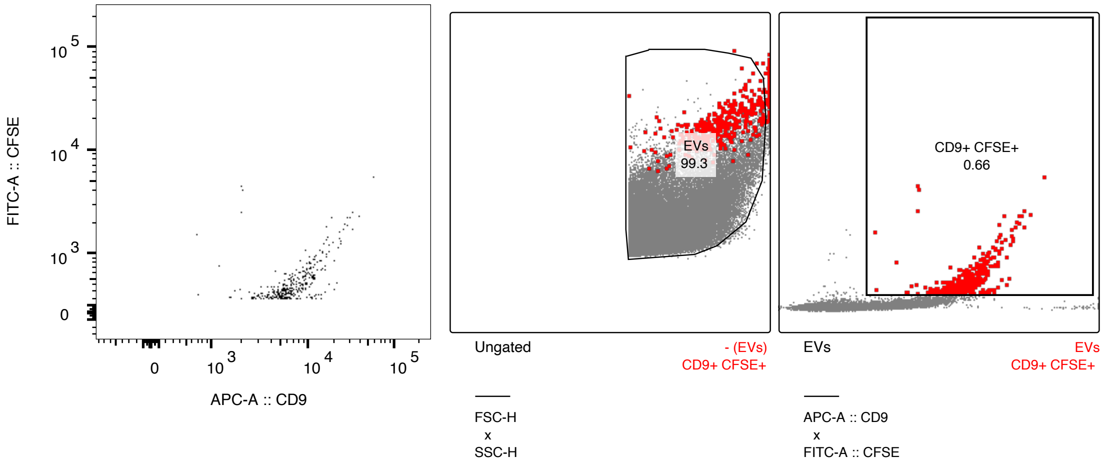

	Sample Name	Freq. of EVs
■	Specimen_001_27-3D (IgG staining)_008.fcs	0.66

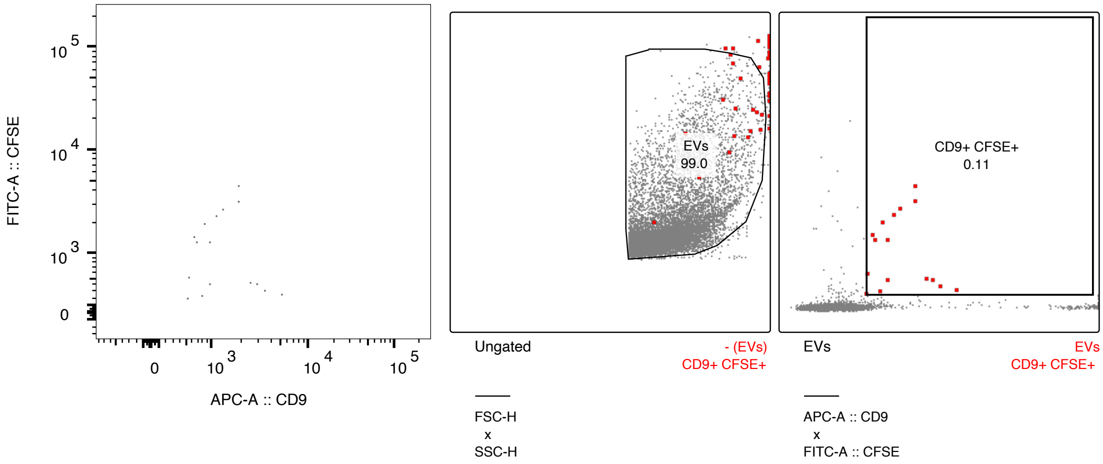

	Sample Name	Freq. of EVs
■	Specimen_001_344-3D (IGG staining)_016.fcs	0.11

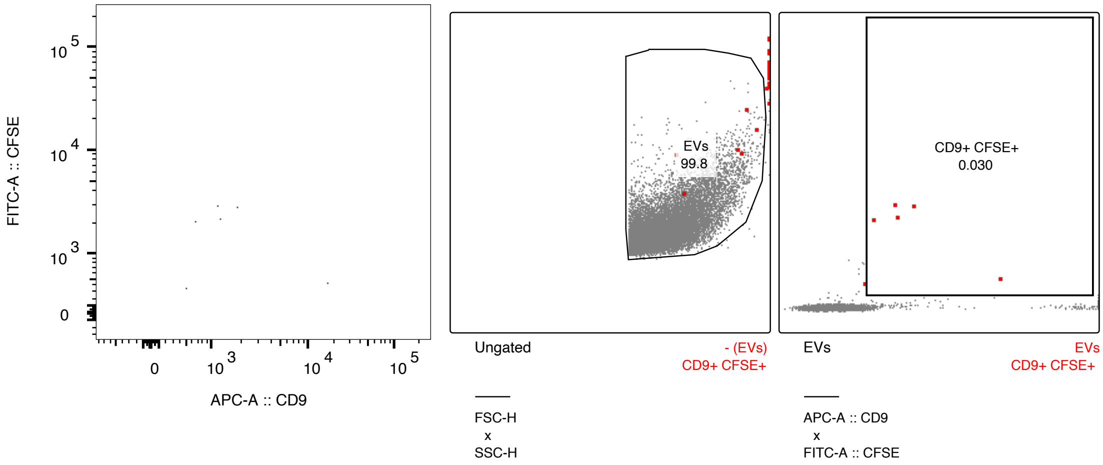

	Sample Name	Freq. of EVs
■	Specimen_001_NP25-3D (IGG staining)_024.fcs	0.030

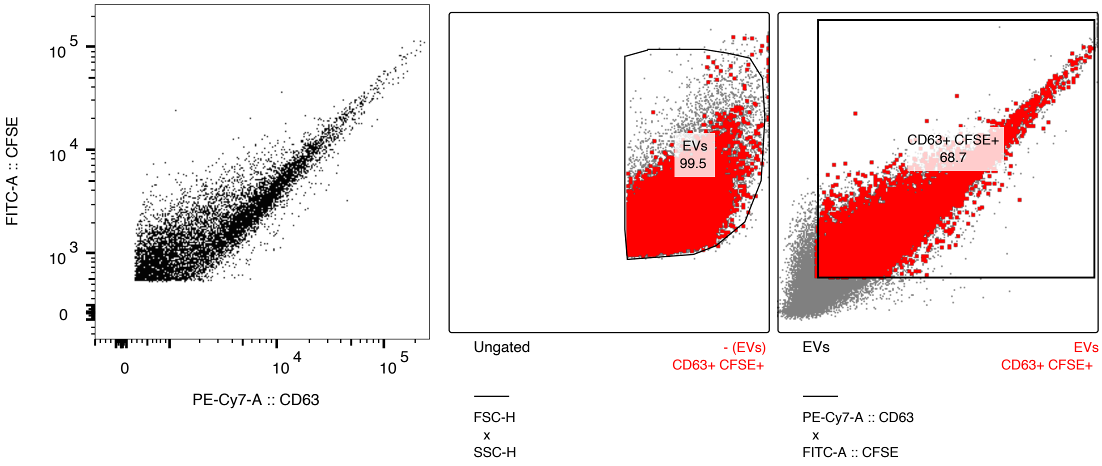

	Sample Name	Freq. of EVs
■	Specimen_001_21-2D (EV staining)_001.fcs	68.7

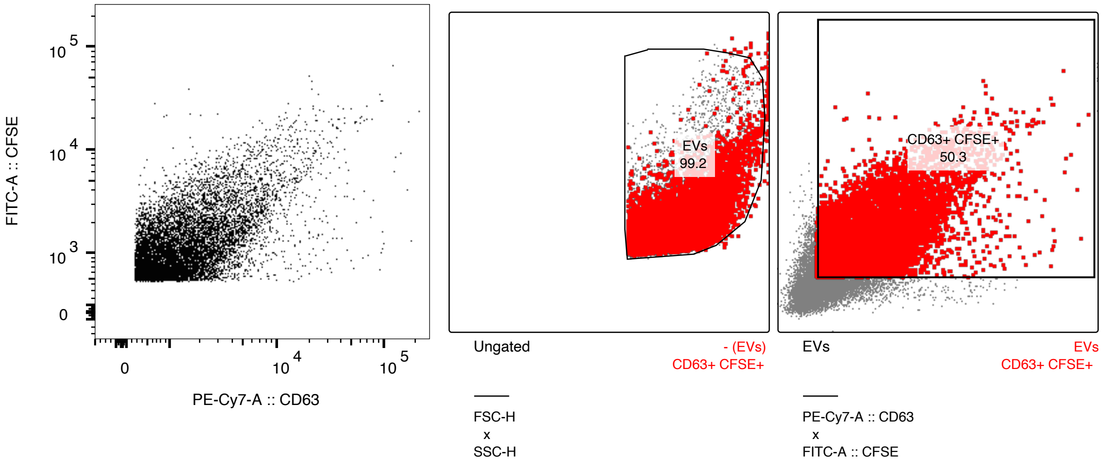

	Sample Name	Freq. of EVs
■	Specimen_001_337-2D (EV staining)_009.fcs	50.3

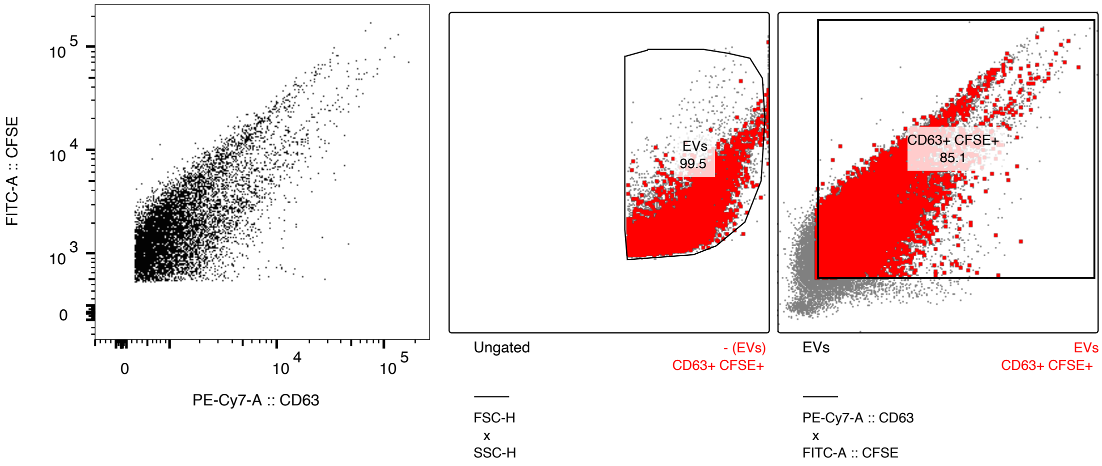

	Sample Name	Freq. of EVs
■	Specimen_001_57-2D (EV staining)_017.fcs	85.1

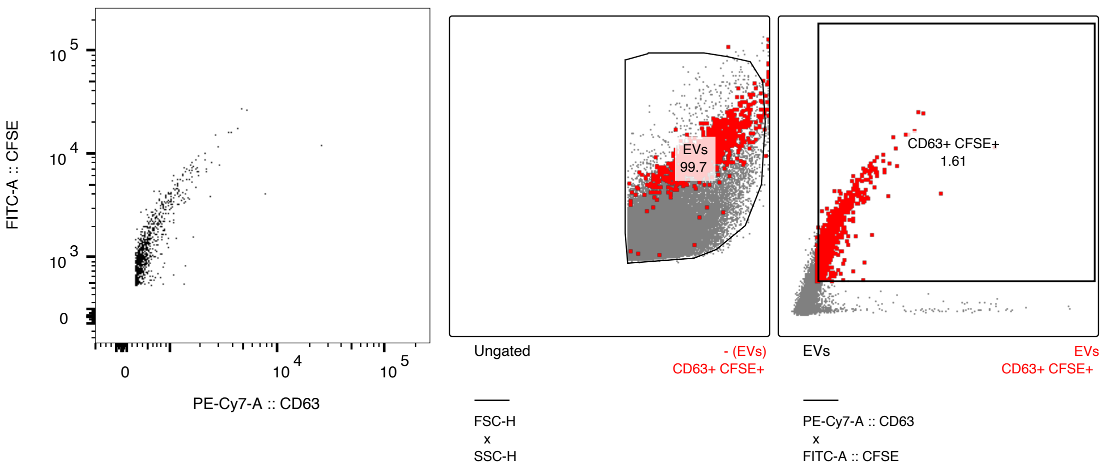

	Sample Name	Freq. of EVs
■	Specimen_001_21-2D (IgG staining)_002.fcs	1.61

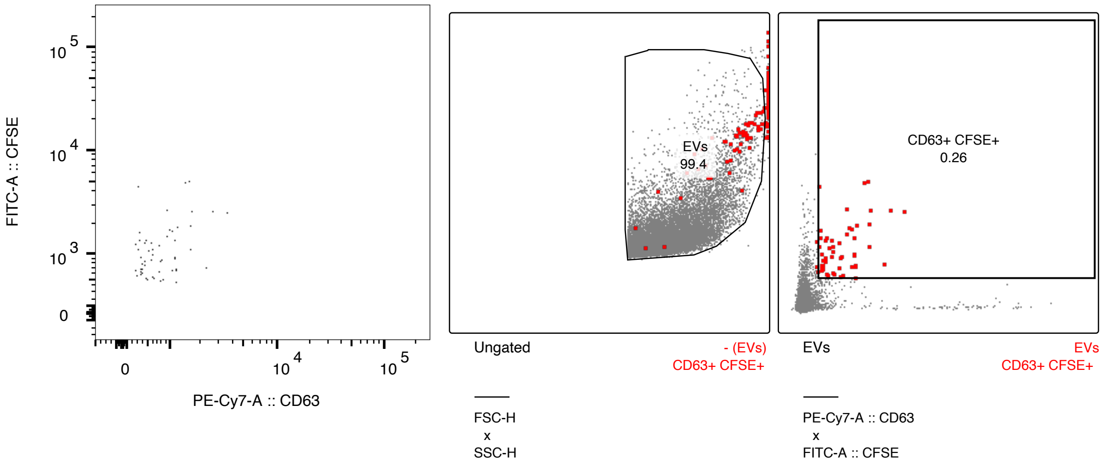

	Sample Name	Freq. of EVs
■	Specimen_001_337-2D (IGG staining)_010.fcs	0.26

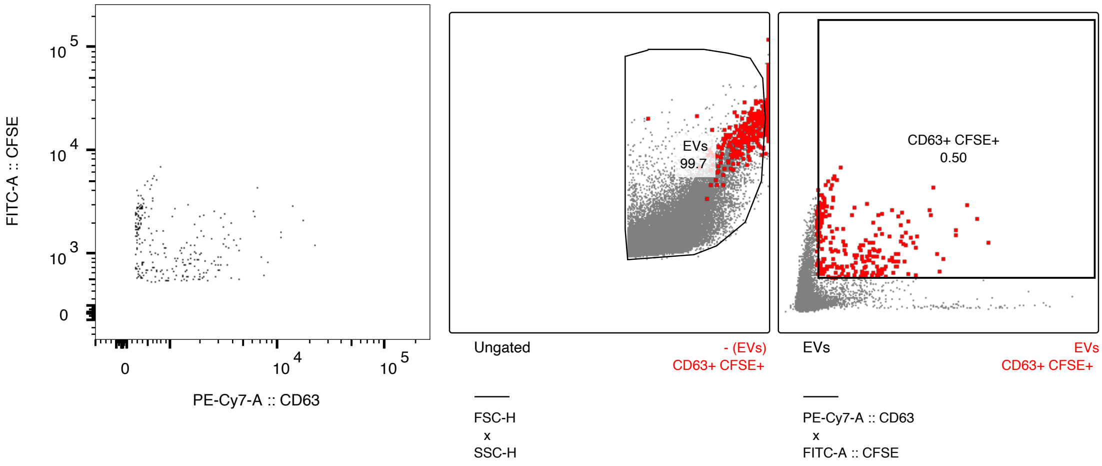

	Sample Name	Freq. of EVs
■	Specimen_001_57-2D (IGG staining)_018.fcs	0.50

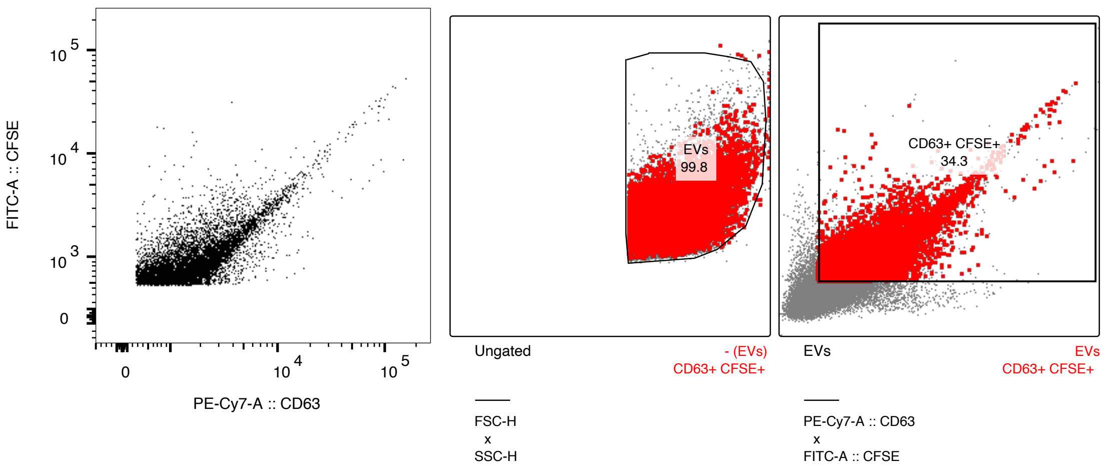

	Sample Name	Freq. of EVs
■	Specimen_001_21-3D (EV staining)_003.fcs	34.3

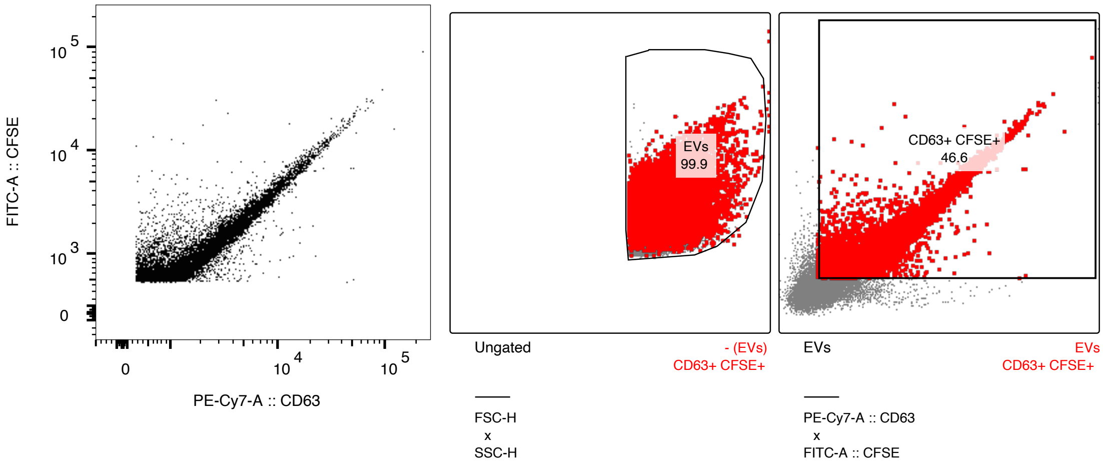

	Sample Name	Freq. of EVs
■	Specimen_001_337-3D (EV staining)_011.fcs	46.6

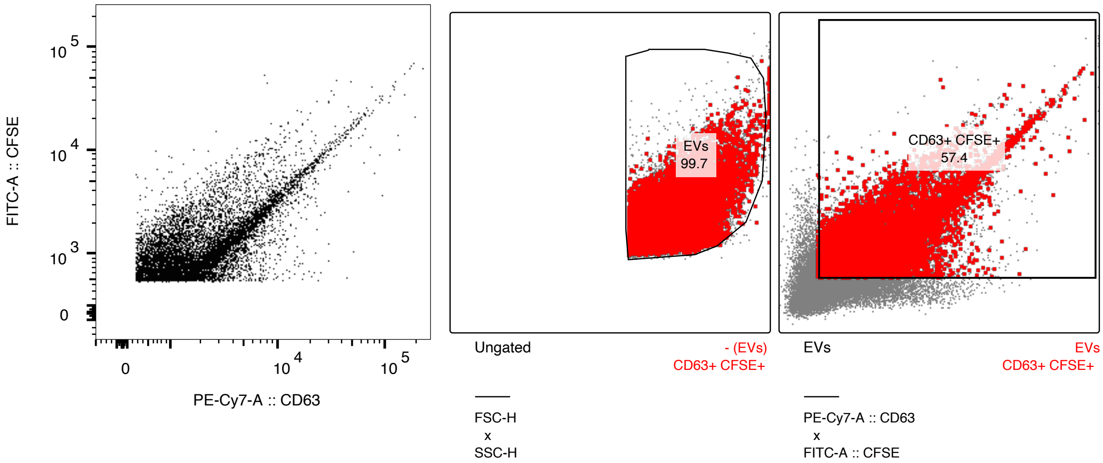

	Sample Name	Freq. of EVs
■	Specimen_001_57-3D (EV staining)_019.fcs	57.4

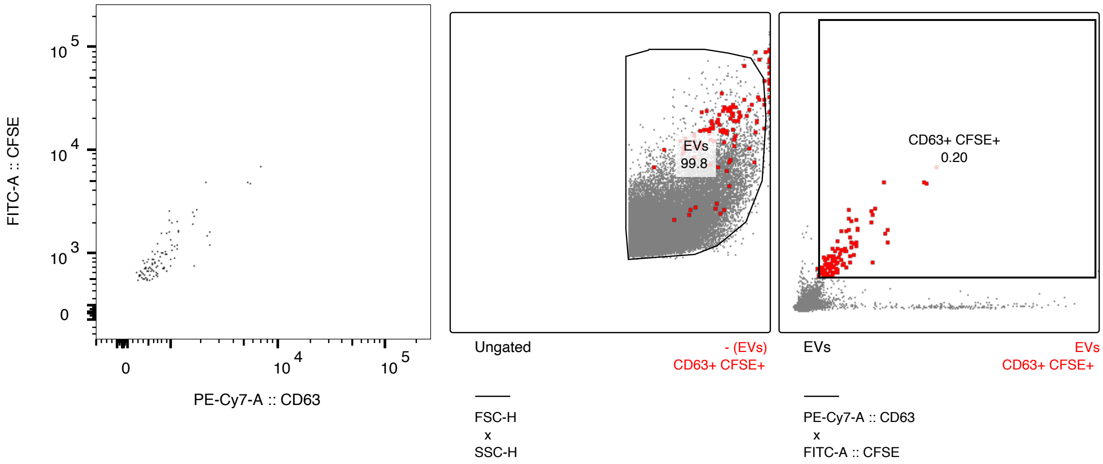

	Sample Name	Freq. of EVs
■	Specimen_001_21-3D (IgG staining)_004.fcs	0.20

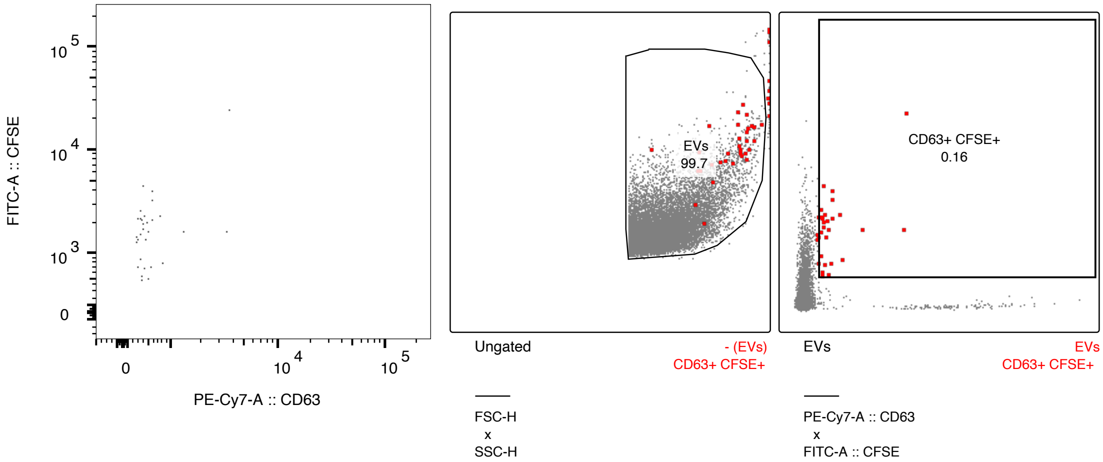

	Sample Name	Freq. of EVs
■	Specimen_001_337-3D (IGG staining)_012.fcs	0.16

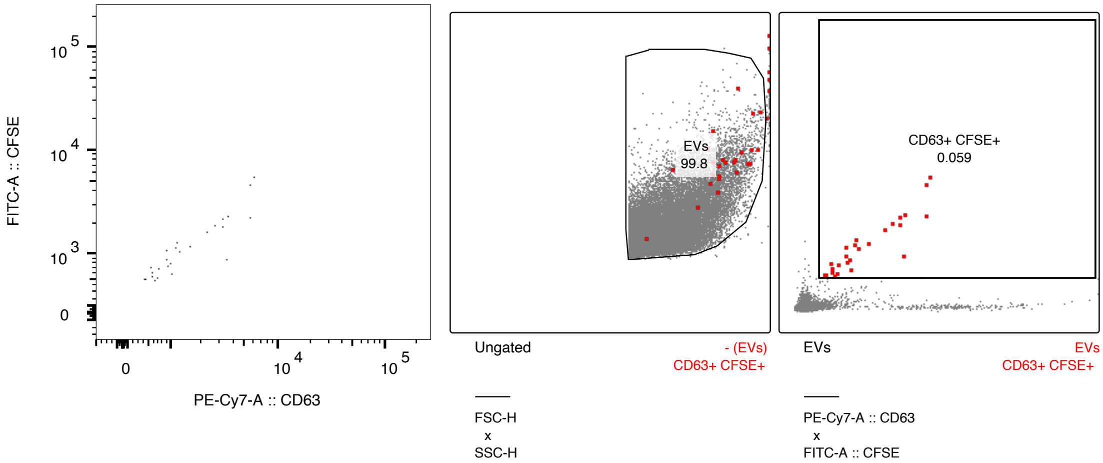

	Sample Name	Freq. of EVs
■	Specimen_001_57-3D (IGG staining)_020.fcs	0.059

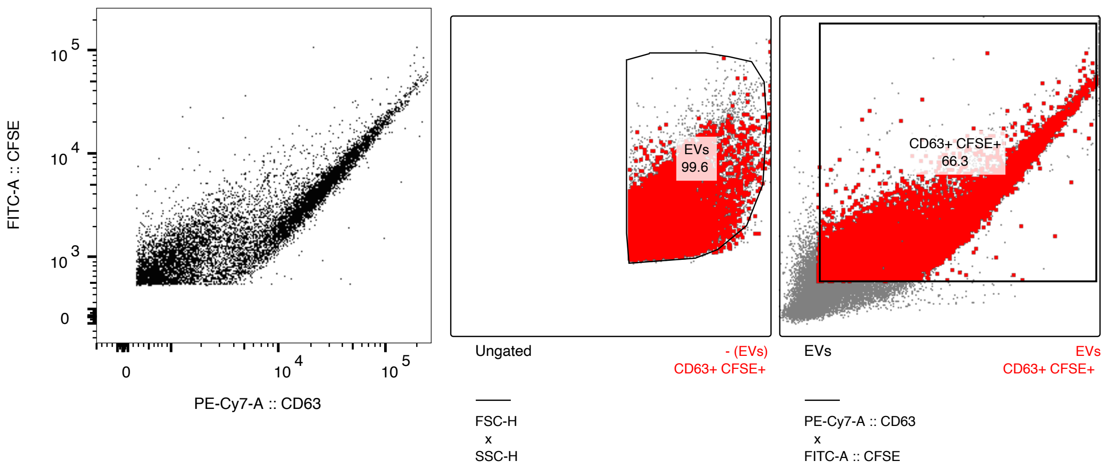

	Sample Name	Freq. of EVs
■	Specimen_001_27-2D (EV staining)_005.fcs	66.3

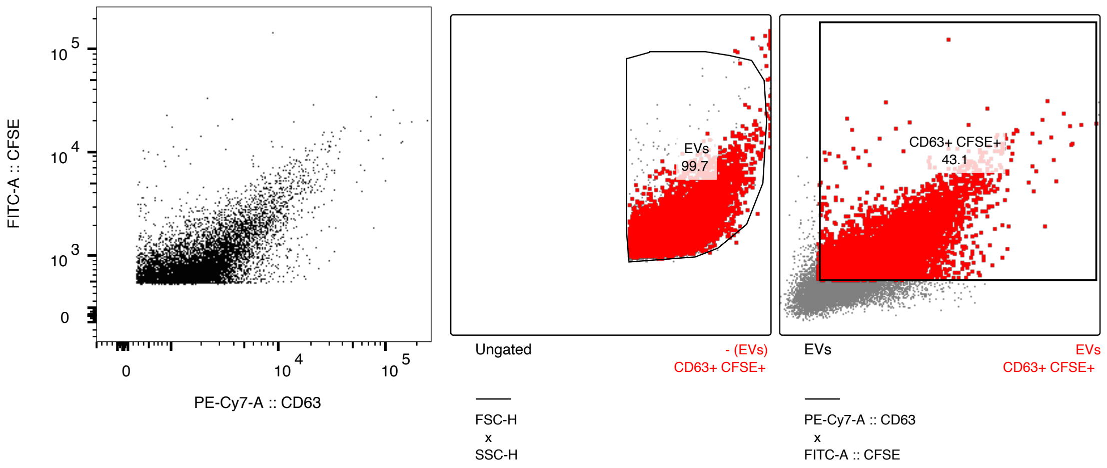

	Sample Name	Freq. of EVs
■	Specimen_001_344-2D (EV staining)_013.fcs	43.1

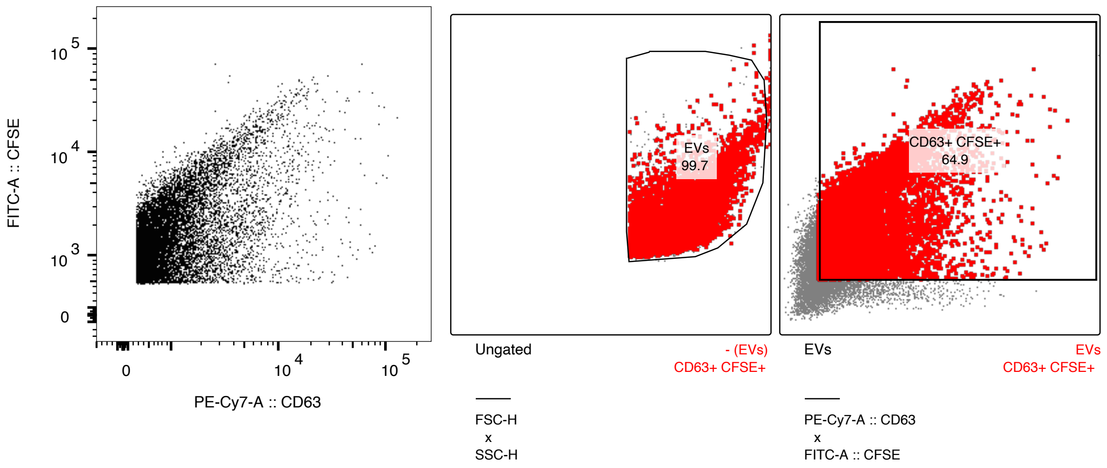

	Sample Name	Freq. of EVs
■	Specimen_001_NP25-2D (EV staining)_021.fcs	64.9

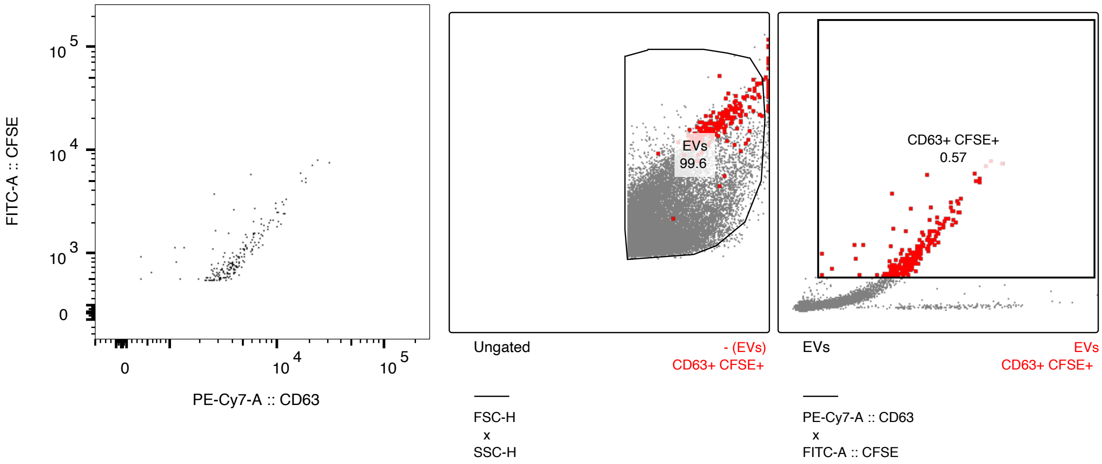

	Sample Name	Freq. of EVs
■	Specimen_001_27-2D (IgG staining)_006.fcs	0.57

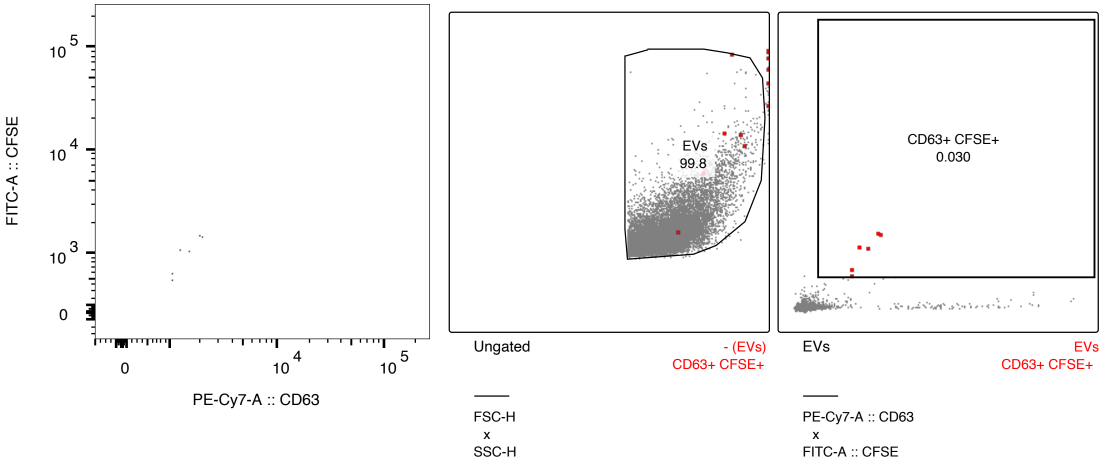

	Sample Name	Freq. of EVs
■	Specimen_001_344-2D (IGG staining)_014.fcs	0.030

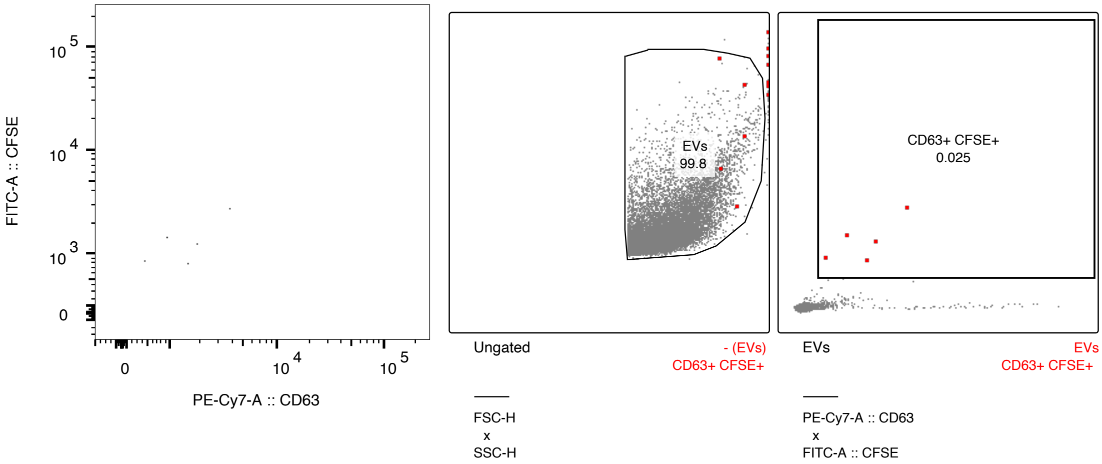

	Sample Name	Freq. of EVs
■	Specimen_001_NP25-2D (IGG staining)_022.fcs	0.025

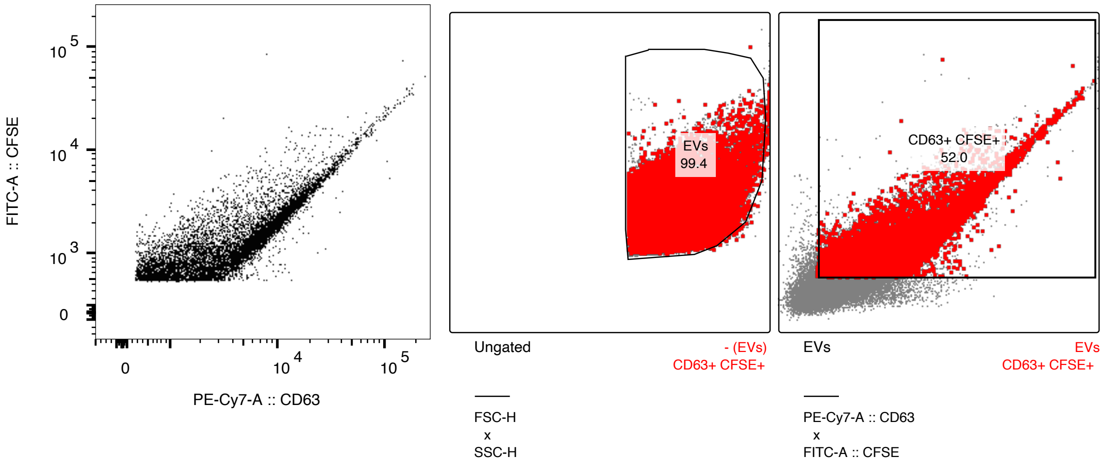

	Sample Name	Freq. of EVs
■	Specimen_001_27-3D (EV staining)_007.fcs	52.0

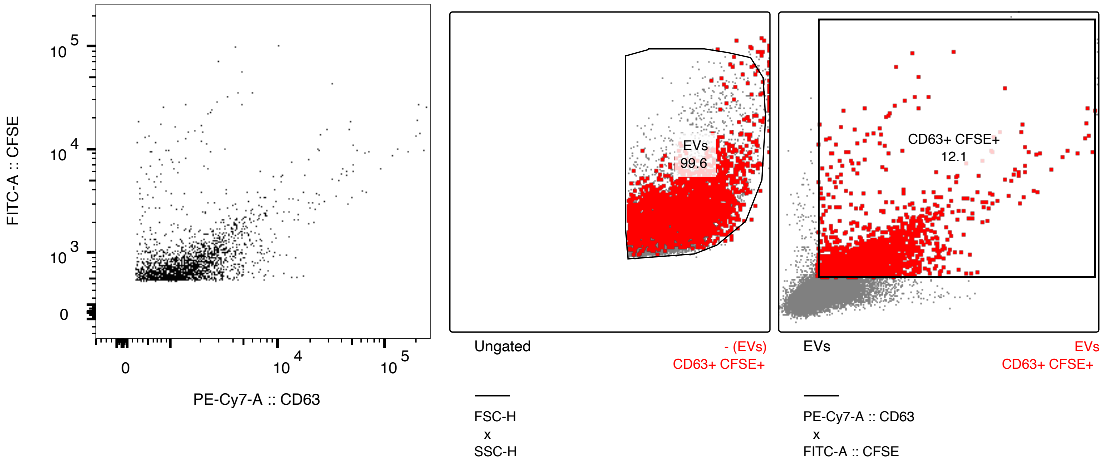

	Sample Name	Freq. of EVs
■	Specimen_001_344-3D (EV staining)_015.fcs	12.1

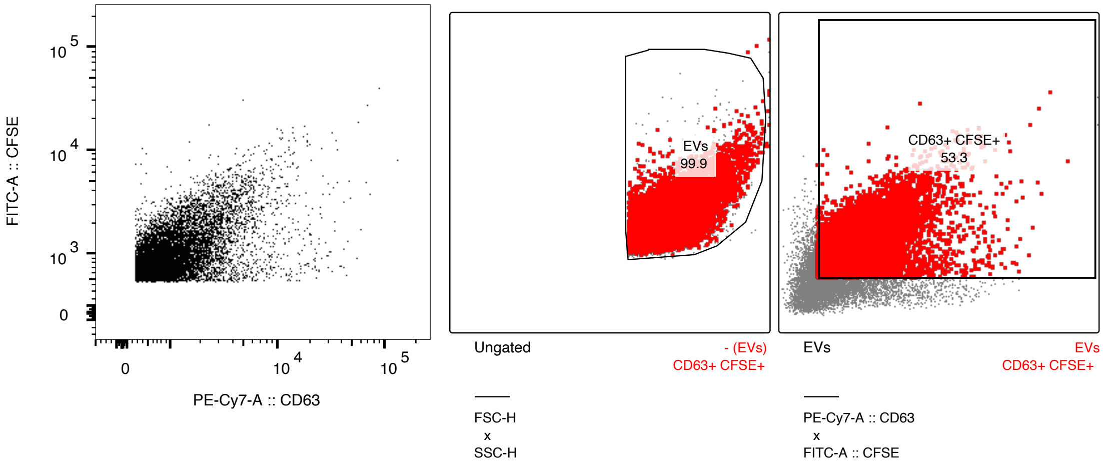

	Sample Name	Freq. of EVs
■	Specimen_001_NP25-3D (EV staining)_023.fcs	53.3

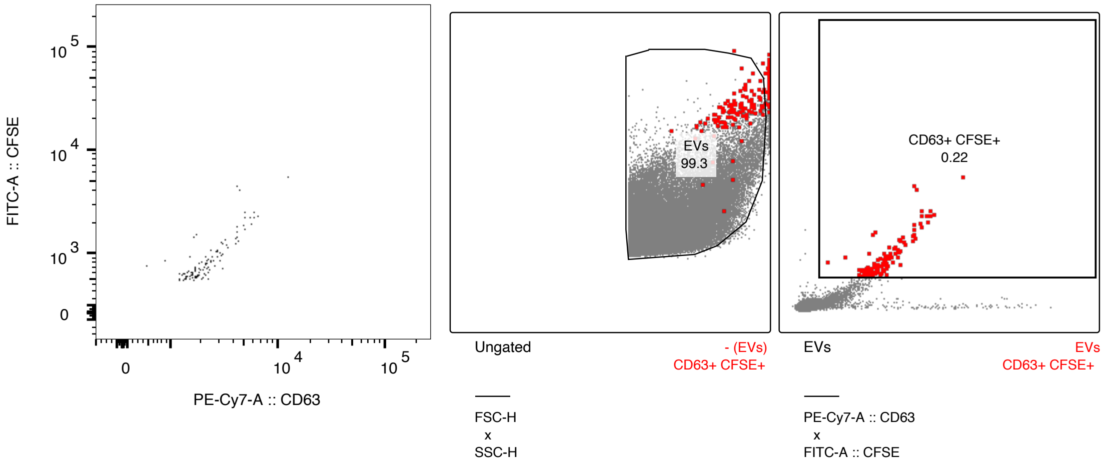

	Sample Name	Freq. of EVs
■	Specimen_001_27-3D (IgG staining)_008.fcs	0.22

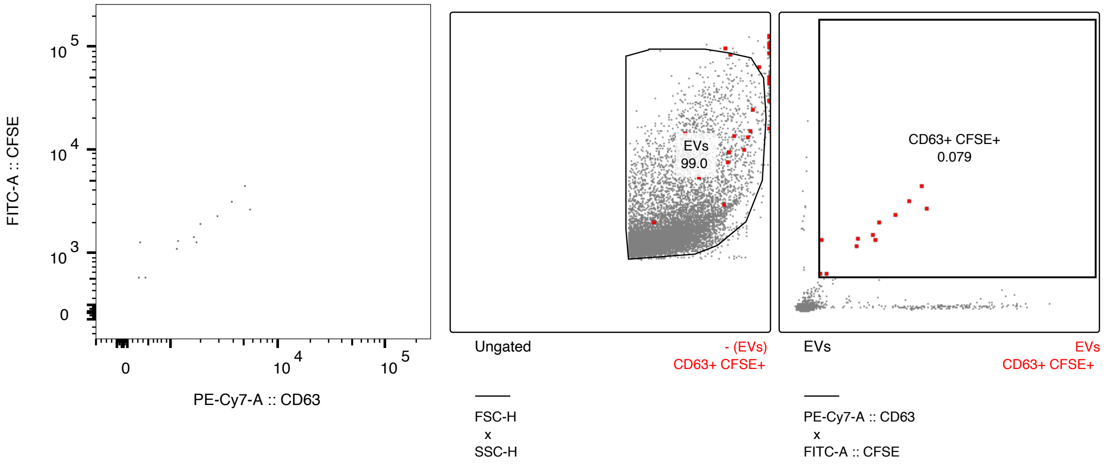

	Sample Name	Freq. of EVs
■	Specimen_001_344-3D (IGG staining)_016.fcs	0.079

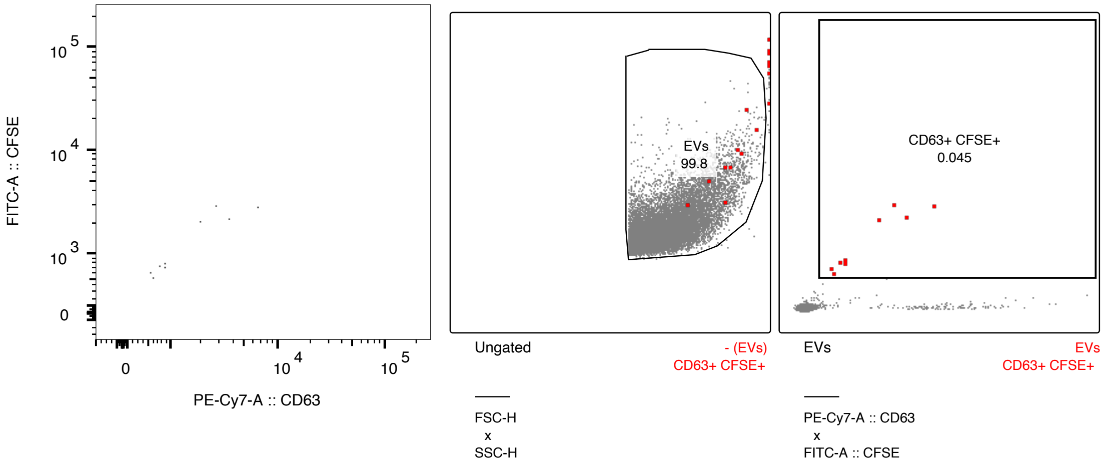

	Sample Name	Freq. of EVs
■	Specimen_001_NP25-3D (IGG staining)_024.fcs	0.045

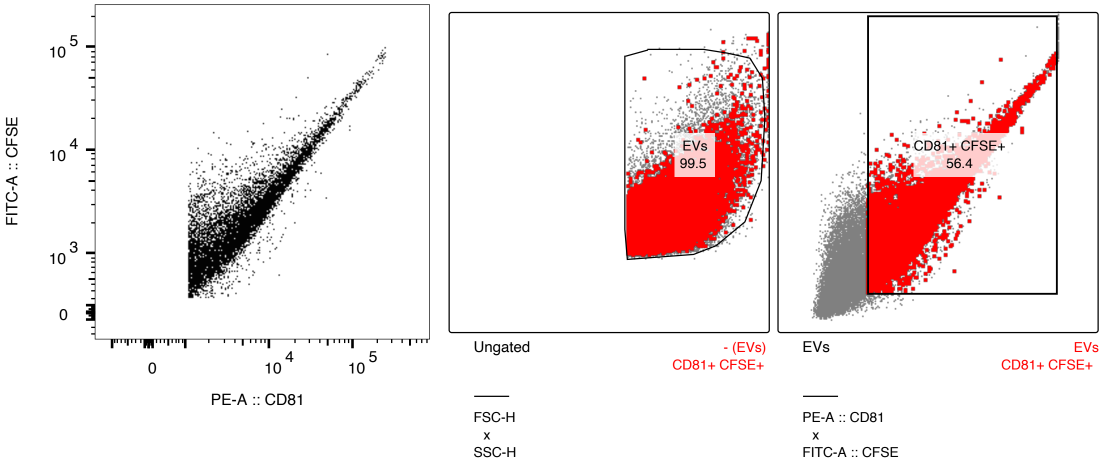

	Sample Name	Freq. of EVs
■	Specimen_001_21-2D (EV staining)_001.fcs	56.4

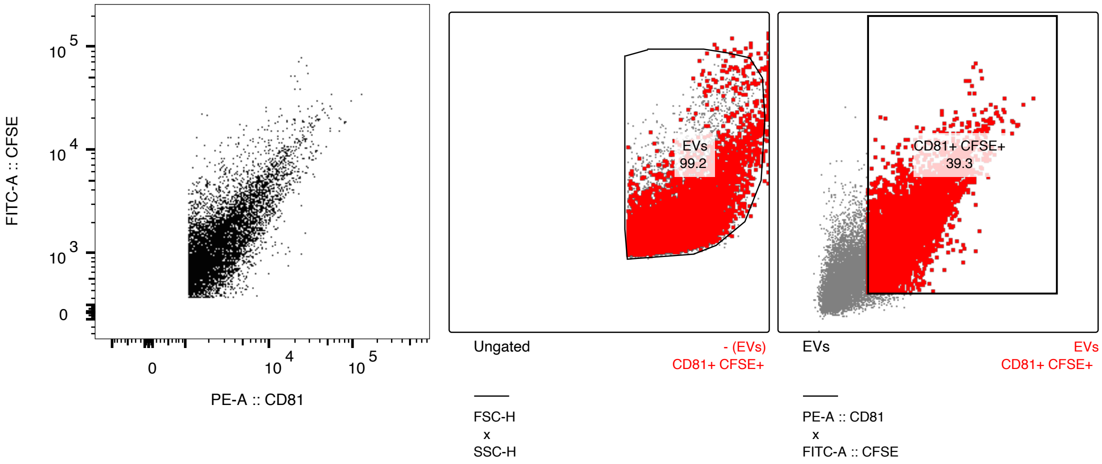

	Sample Name	Freq. of EVs
■	Specimen_001_337-2D (EV staining)_009.fcs	39.3

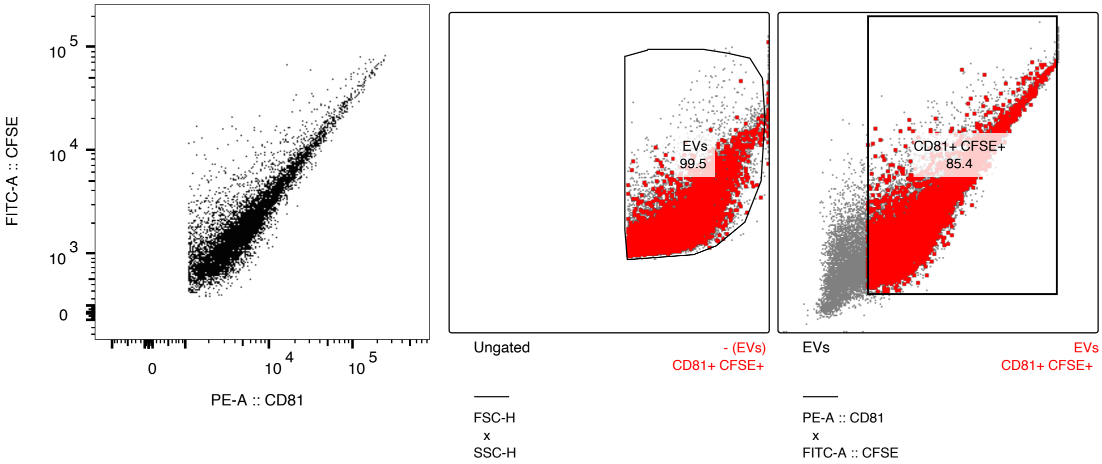

	Sample Name	Freq. of EVs
■	Specimen_001_57-2D (EV staining)_017.fcs	85.4

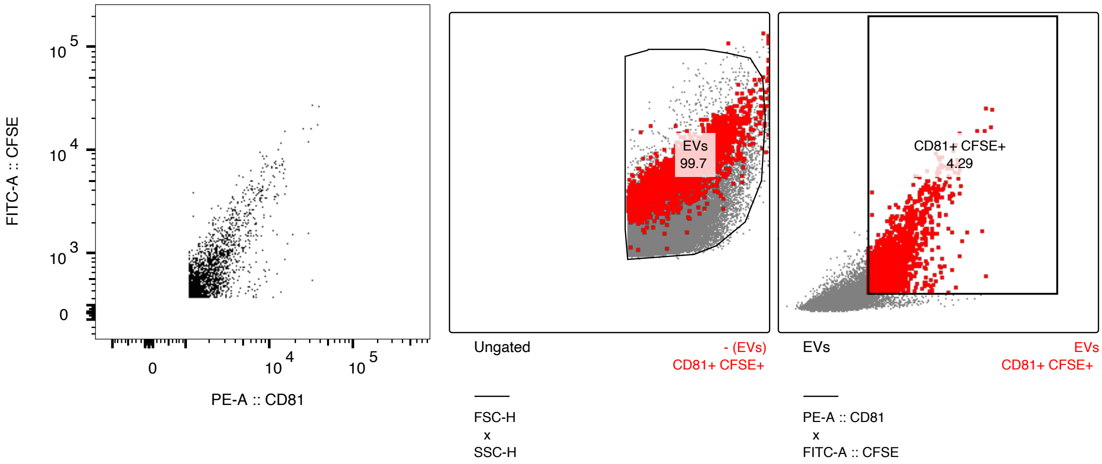

	Sample Name	Freq. of EVs
■	Specimen_001_21-2D (IgG staining)_002.fcs	4.29

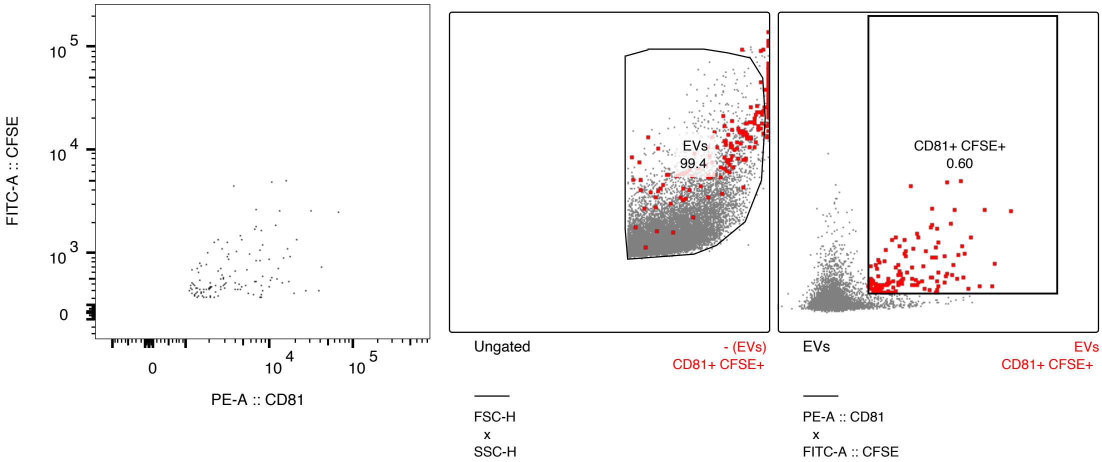

	Sample Name	Freq. of EVs
■	Specimen_001_337-2D (IGG staining)_010.fcs	0.60

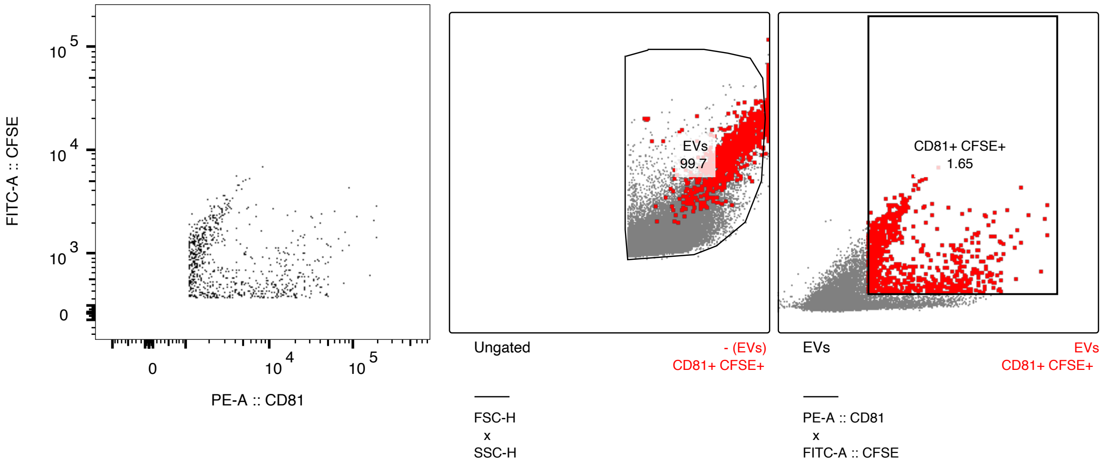

	Sample Name	Freq. of EVs
■	Specimen_001_57-2D (IGG staining)_018.fcs	1.65

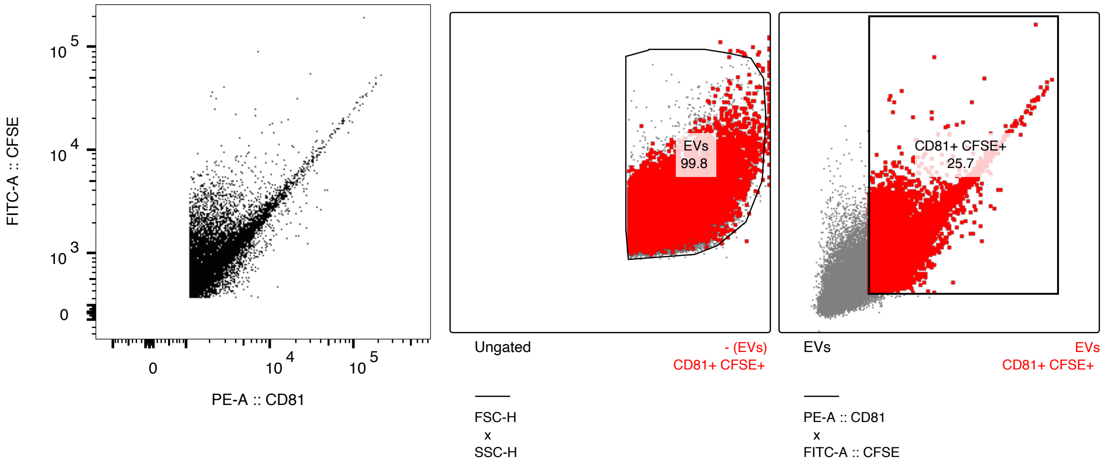

	Sample Name	Freq. of EVs
■	Specimen_001_21-3D (EV staining)_003.fcs	25.7

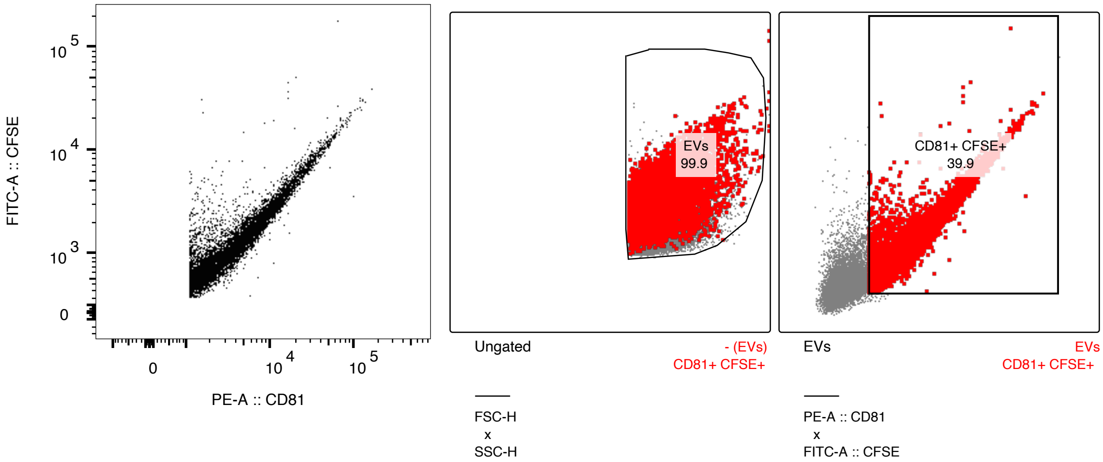

	Sample Name	Freq. of EVs
■	Specimen_001_337-3D (EV staining)_011.fcs	39.9

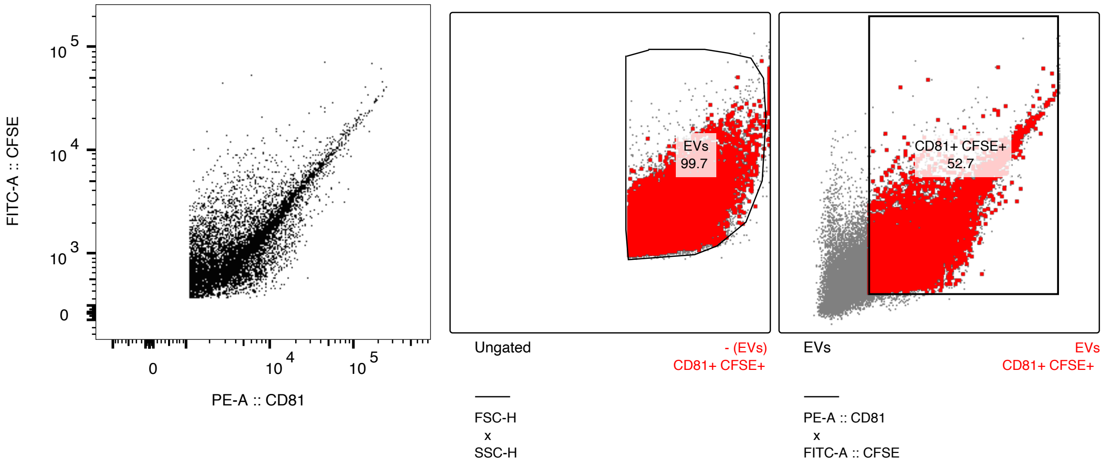

	Sample Name	Freq. of EVs
■	Specimen_001_57-3D (EV staining)_019.fcs	52.7

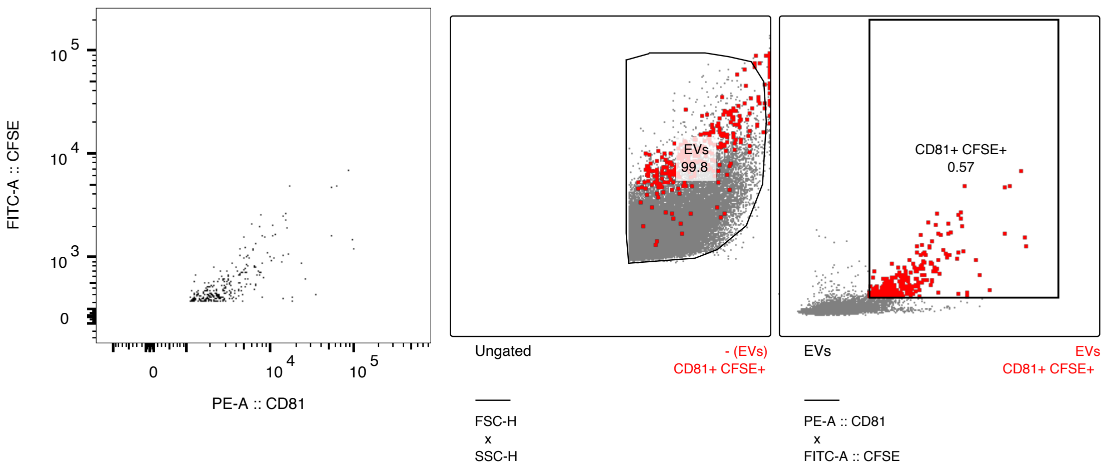

	Sample Name	Freq. of EVs
■	Specimen_001_21-3D (IgG staining)_004.fcs	0.57

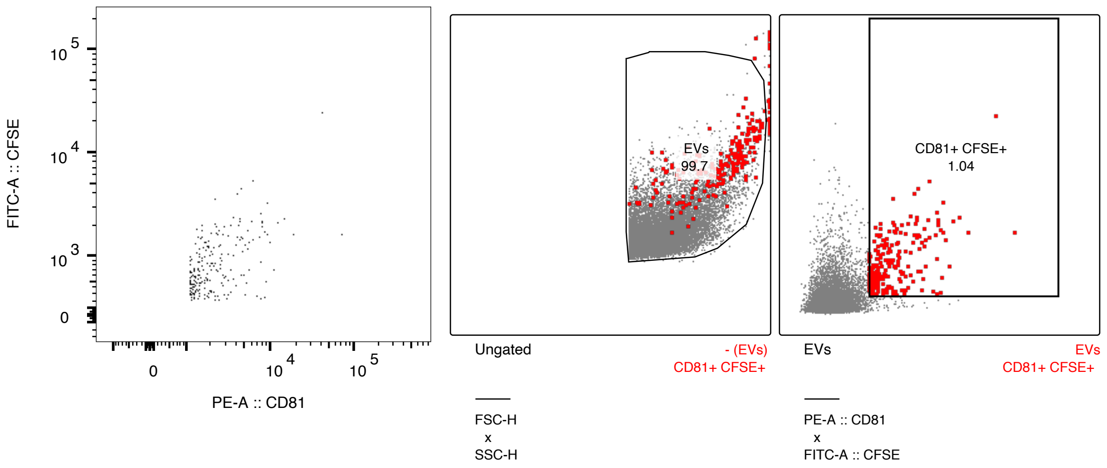

	Sample Name	Freq. of EVs
■	Specimen_001_337-3D (IGG staining)_012.fcs	1.04

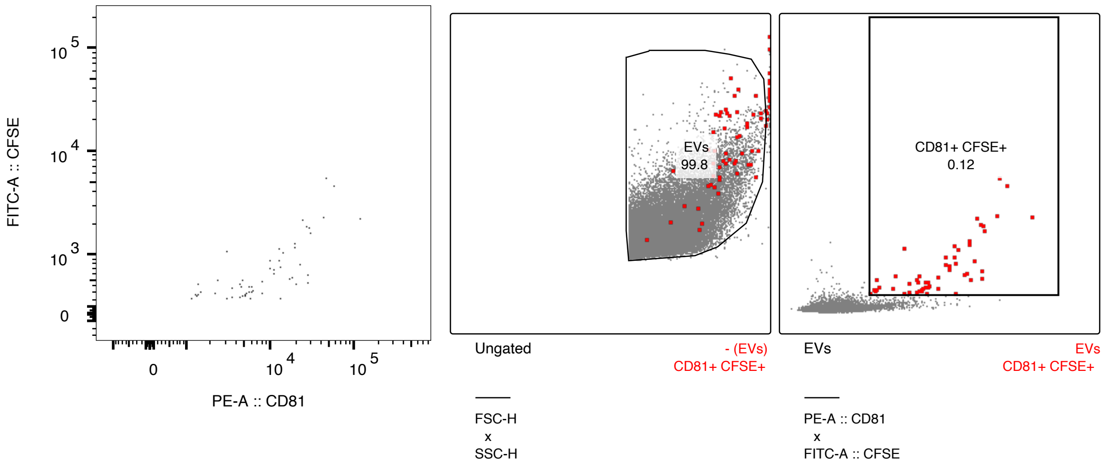

	Sample Name	Freq. of EVs
■	Specimen_001_57-3D (IGG staining)_020.fcs	0.12

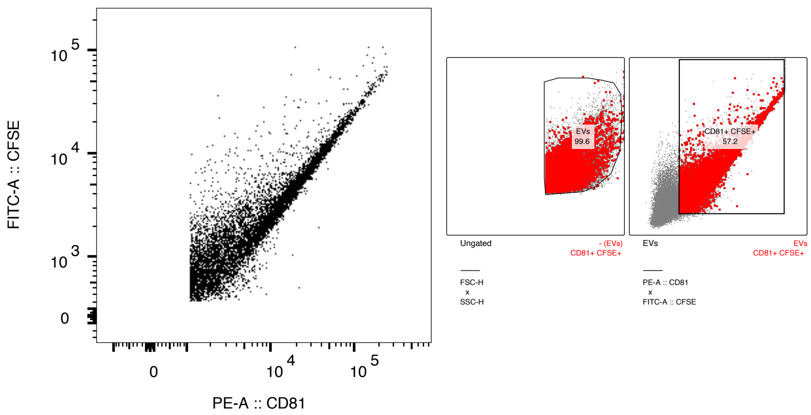

	Sample Name	Freq. of EVs
■	Specimen_001_27-2D (EV staining)_005.fcs	57.2

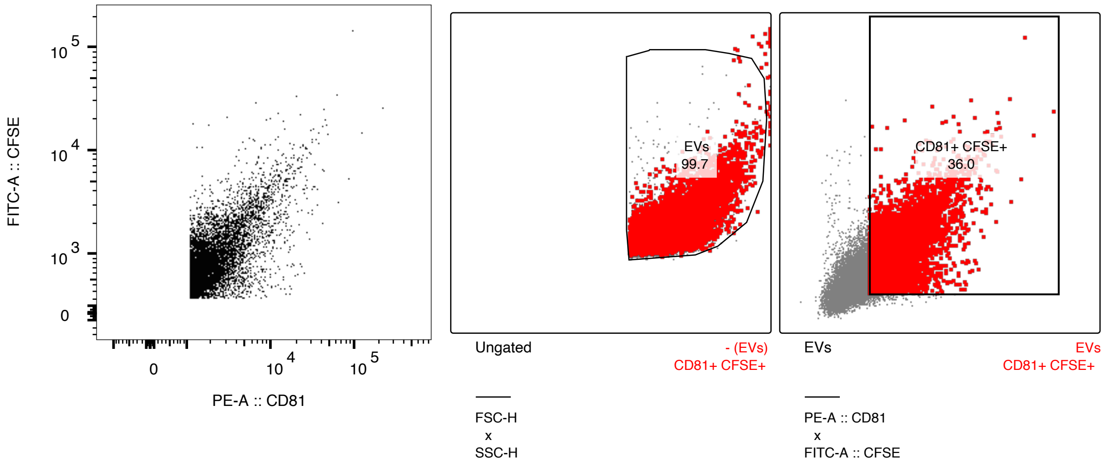

	Sample Name	Freq. of EVs
■	Specimen_001_344-2D (EV staining)_013.fcs	36.0

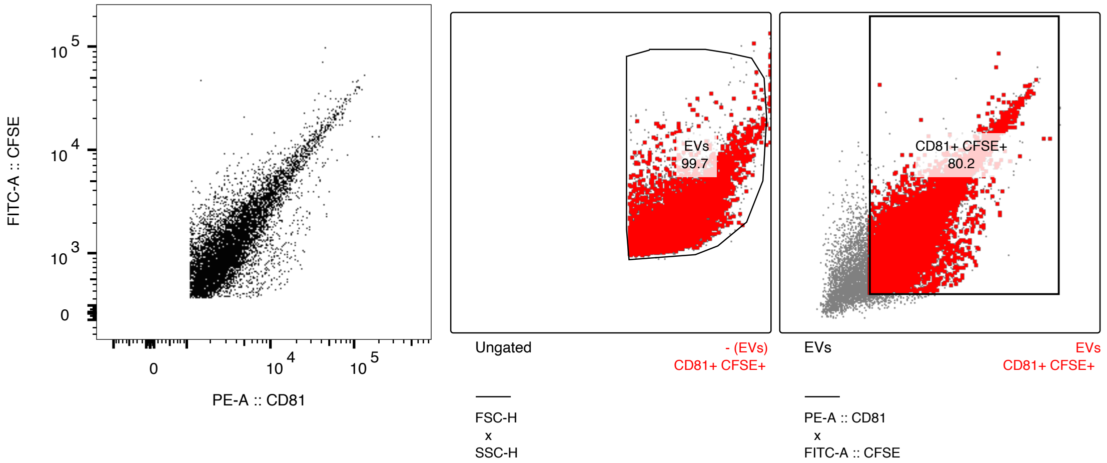

	Sample Name	Freq. of EVs
■	Specimen_001_NP25-2D (EV staining)_021.fcs	80.2

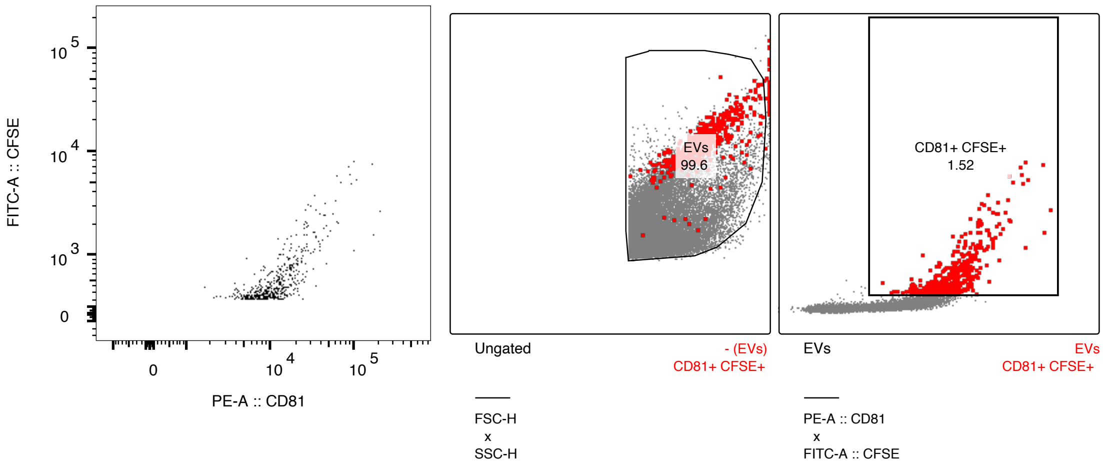

	Sample Name	Freq. of EVs
■	Specimen_001_27-2D (IgG staining)_006.fcs	1.52

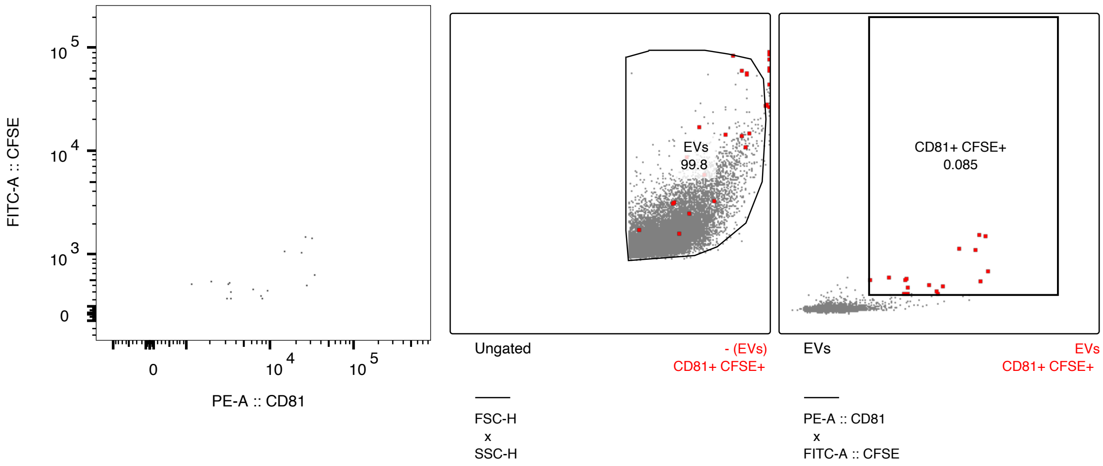

	Sample Name	Freq. of EVs
■	Specimen_001_344-2D (IGG staining)_014.fcs	0.085

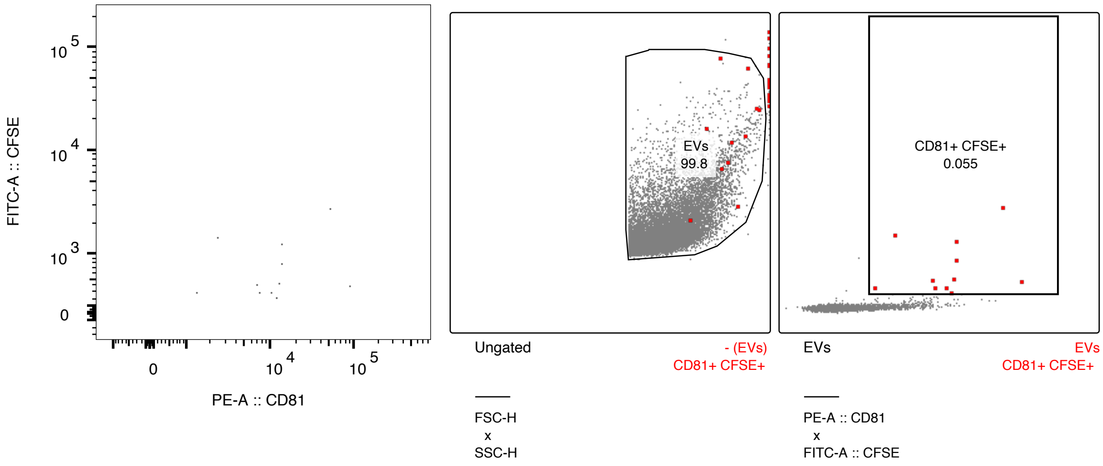

	Sample Name	Freq. of EVs
■	Specimen_001_NP25-2D (IGG staining)_022.fcs	0.055

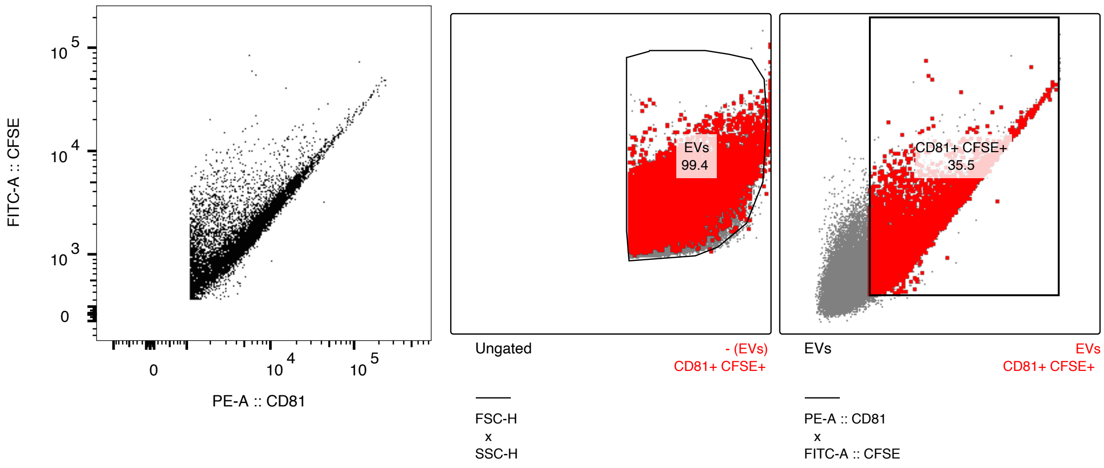

	Sample Name	Freq. of EVs
■	Specimen_001_27-3D (EV staining)_007.fcs	35.5

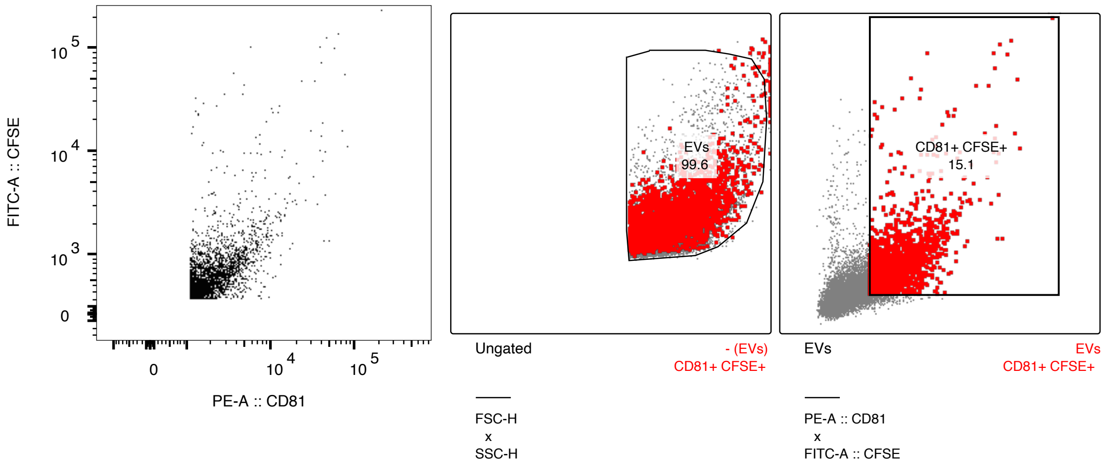

	Sample Name	Freq. of EVs
■	Specimen_001_344-3D (EV staining)_015.fcs	15.1

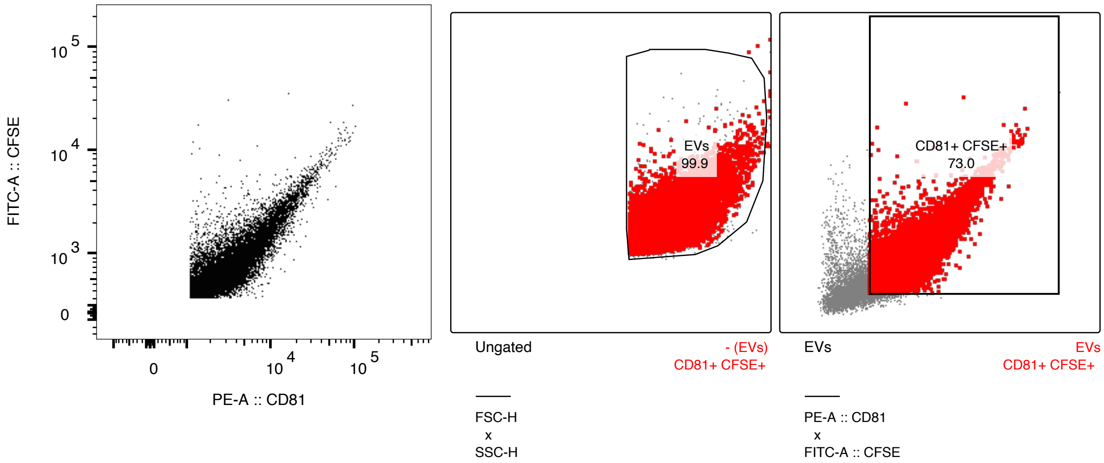

	Sample Name	Freq. of EVs
■	Specimen_001_NP25-3D (EV staining)_023.fcs	73.0

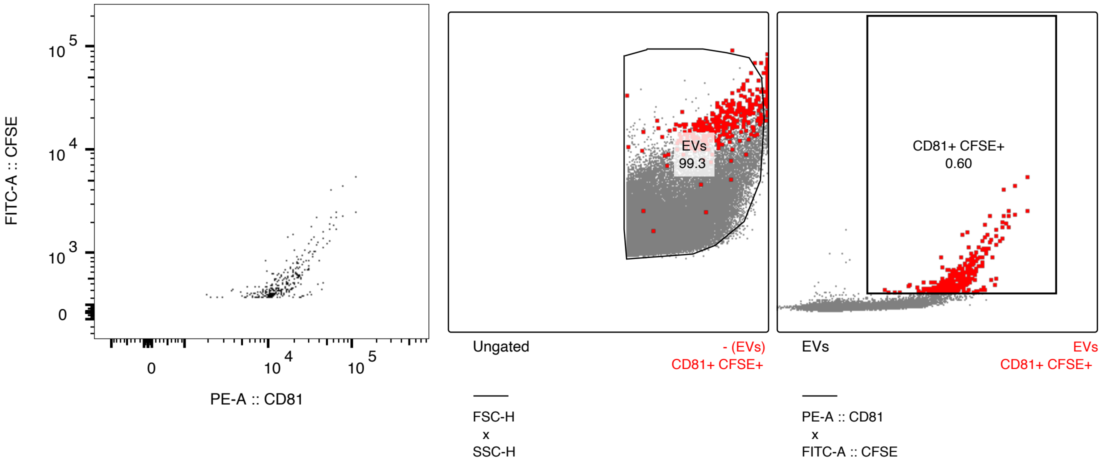

Sample Name	Freq. of EVs
Specimen_001_27-3D (IgG staining)_008.fcs	0.60

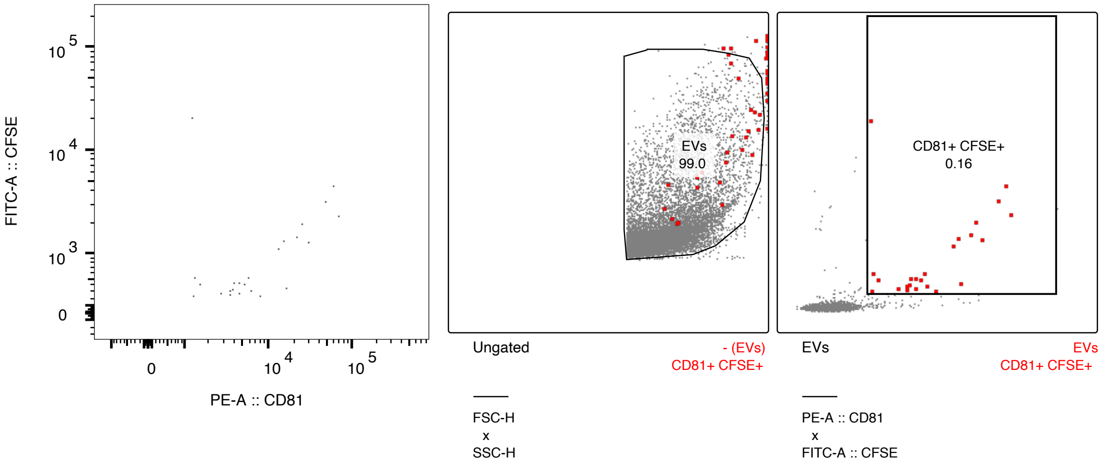

Sample Name	Freq. of EVs
Specimen_001_344-3D (IGG staining)_016.fcs	0.16

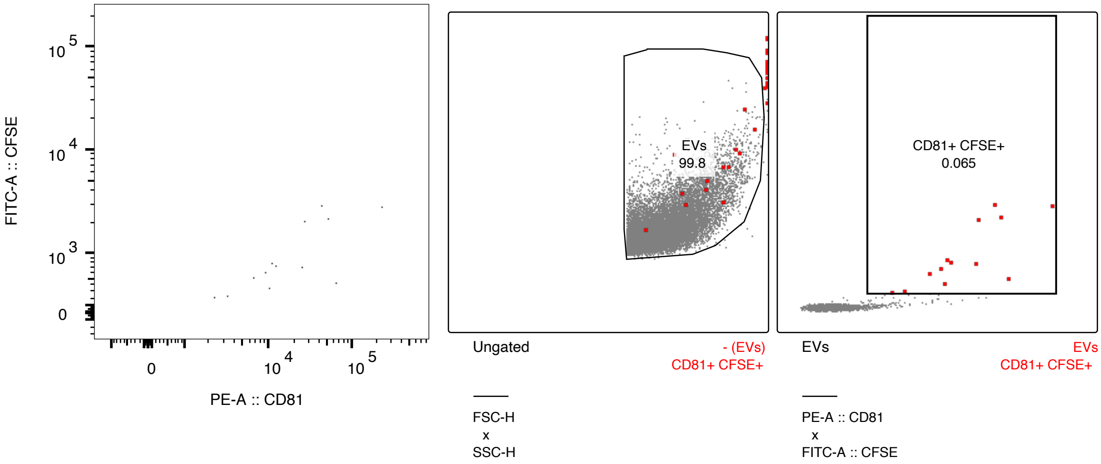

Sample Name	Freq. of EVs
Specimen_001_NP25-3D (IGG staining)_024.fcs	0.065

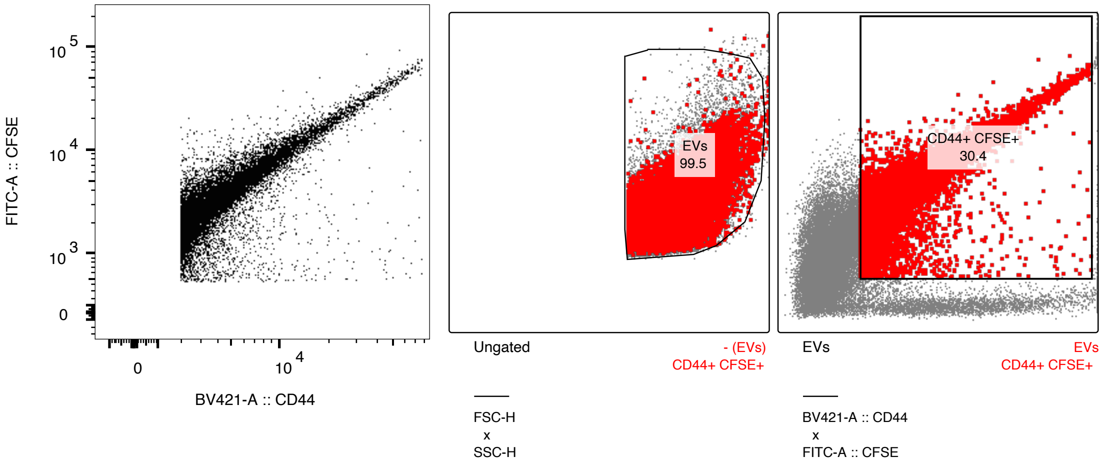

	Sample Name	Freq. of EVs
■	Specimen_001_21-2D (EV staining)_001.fcs	30.4

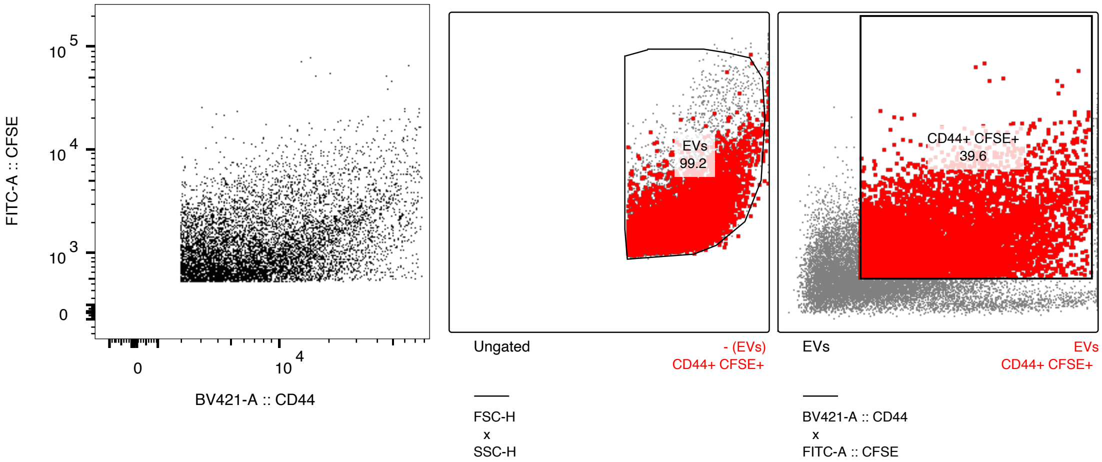

	Sample Name	Freq. of EVs
■	Specimen_001_337-2D (EV staining)_009.fcs	39.6

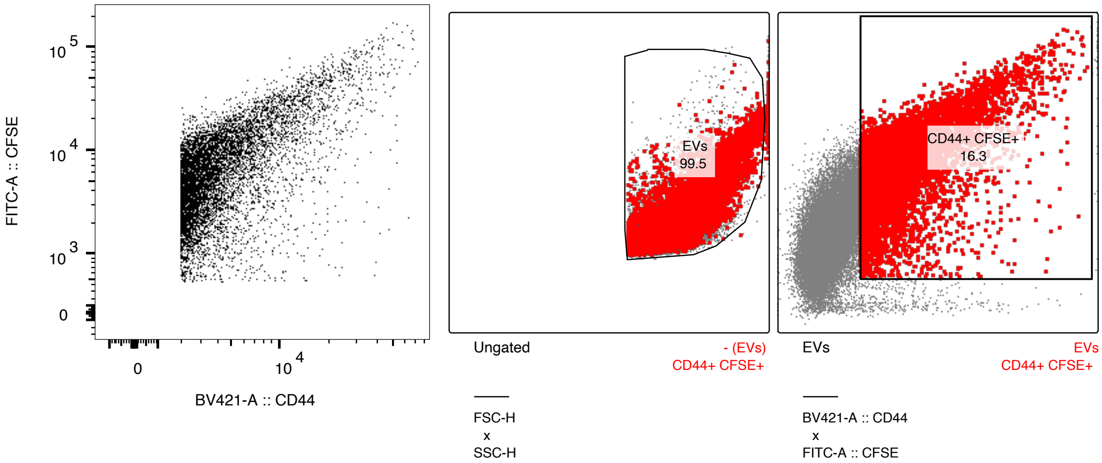

	Sample Name	Freq. of EVs
■	Specimen_001_57-2D (EV staining)_017.fcs	16.3

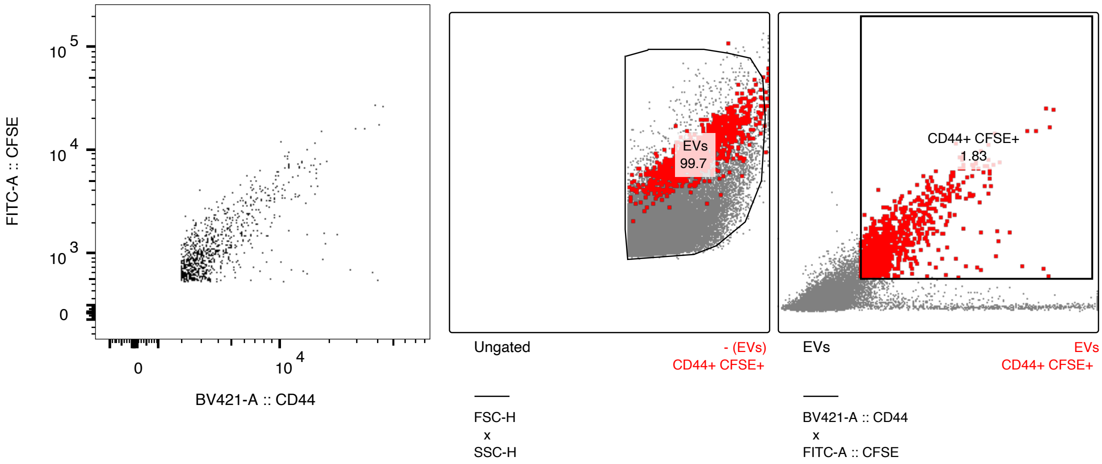

	Sample Name	Freq. of EVs
■	Specimen_001_21-2D (IgG staining)_002.fcs	1.83

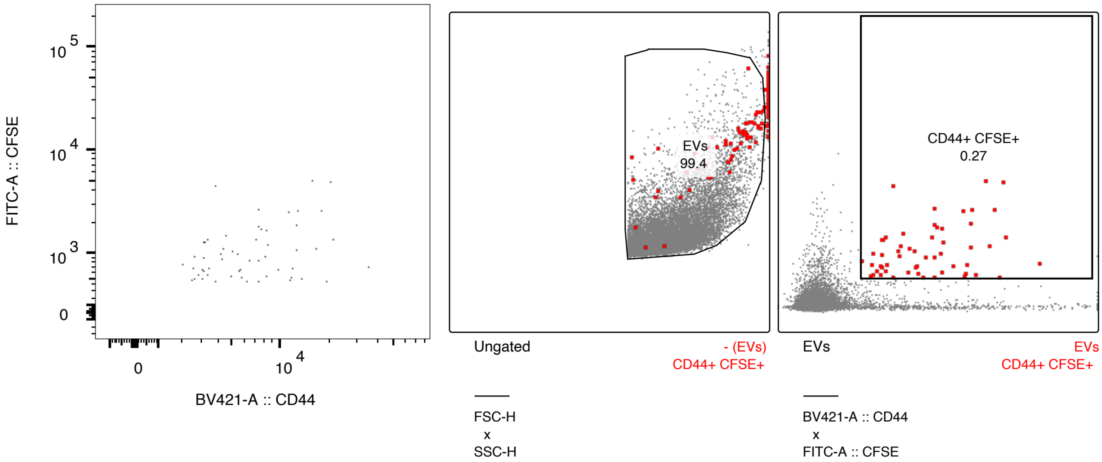

	Sample Name	Freq. of EVs
■	Specimen_001_337-2D (IGG staining)_010.fcs	0.27

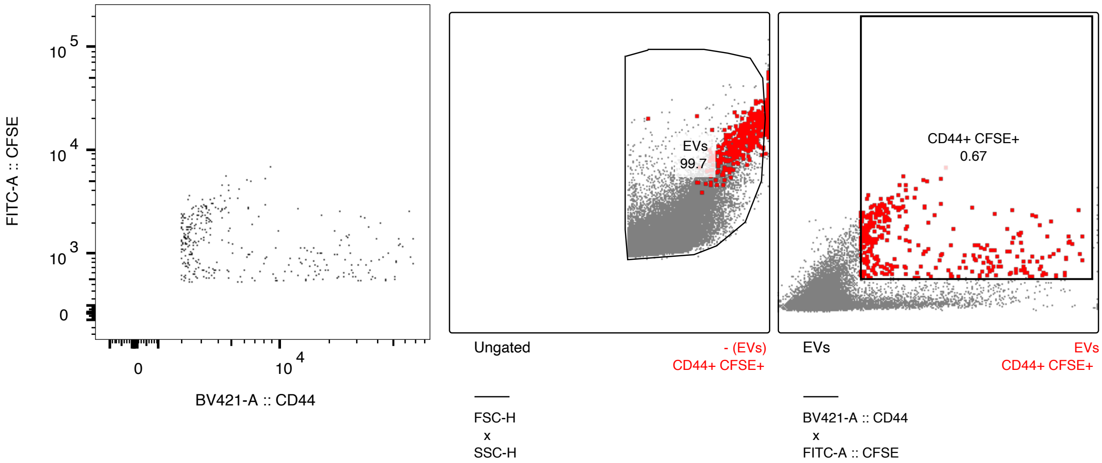

	Sample Name	Freq. of EVs
■	Specimen_001_57-2D (IGG staining)_018.fcs	0.67

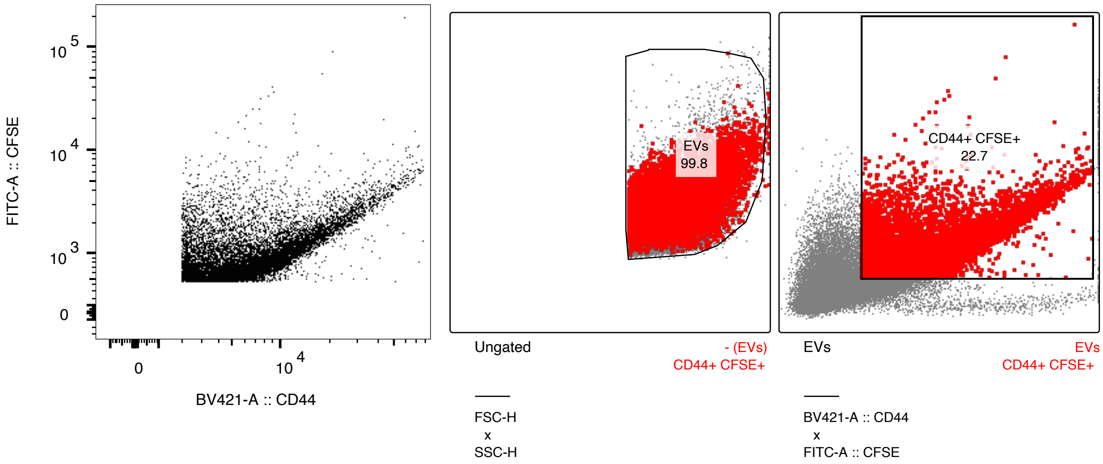

	Sample Name	Freq. of EVs
■	Specimen_001_21-3D (EV staining)_003.fcs	22.7

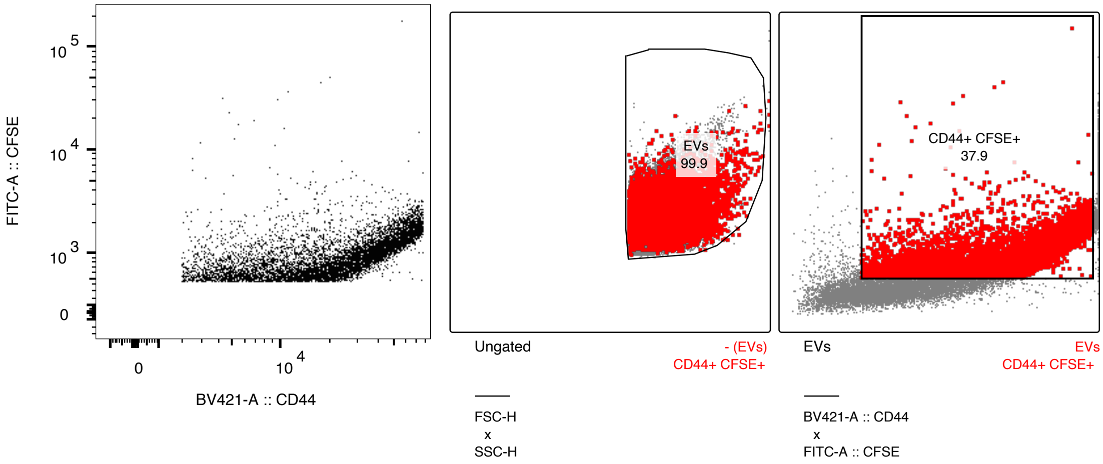

	Sample Name	Freq. of EVs
■	Specimen_001_337-3D (EV staining)_011.fcs	37.9

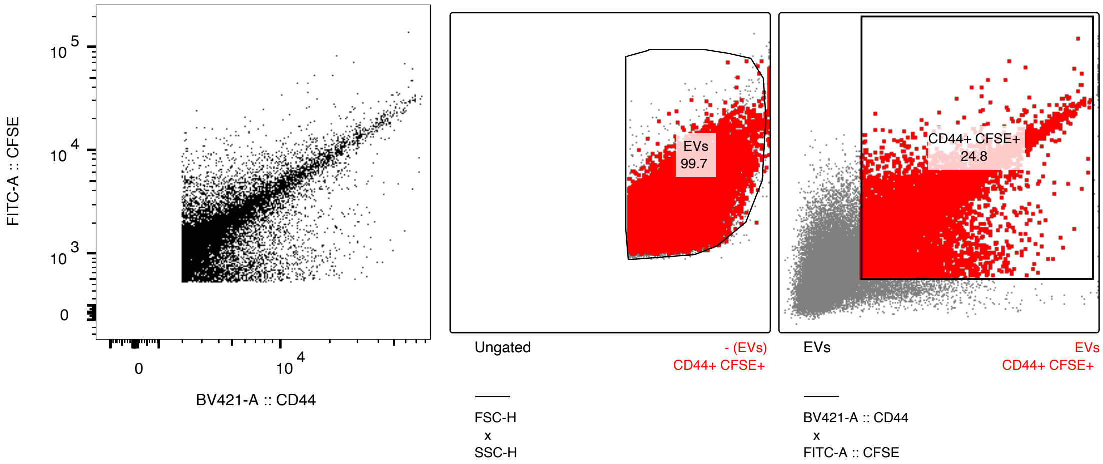

	Sample Name	Freq. of EVs
■	Specimen_001_57-3D (EV staining)_019.fcs	24.8

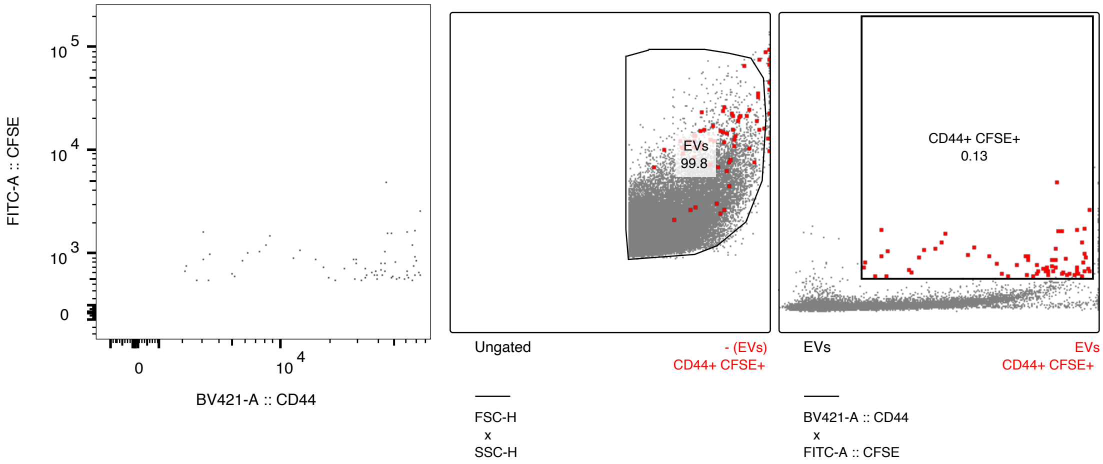

	Sample Name	Freq. of EVs
■	Specimen_001_21-3D (IgG staining)_004.fcs	0.13

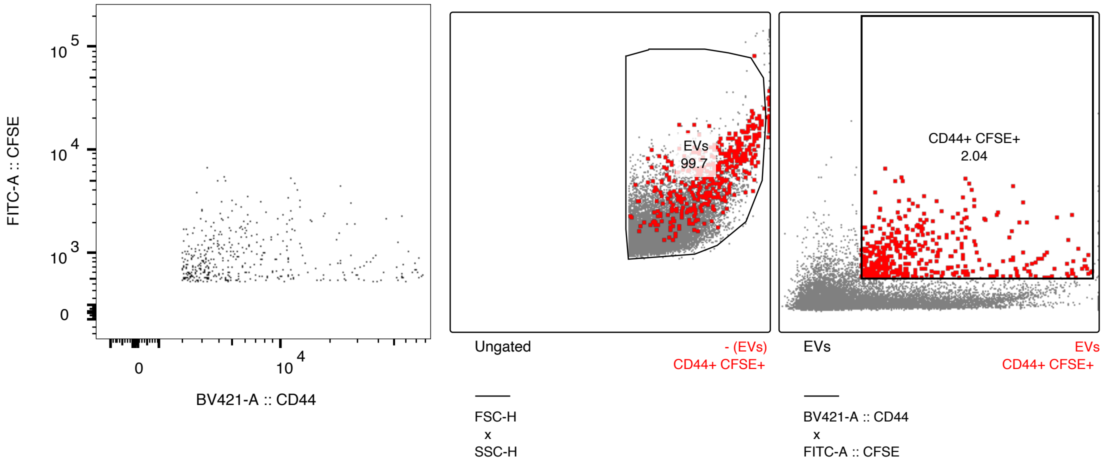

	Sample Name	Freq. of EVs
■	Specimen_001_337-3D (IGG staining)_012.fcs	2.04

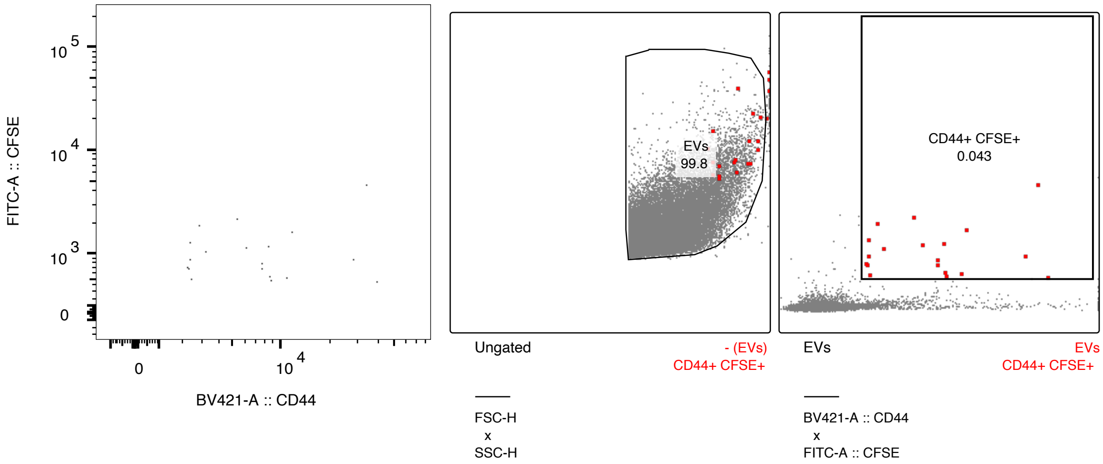

	Sample Name	Freq. of EVs
■	Specimen_001_57-3D (IGG staining)_020.fcs	0.043

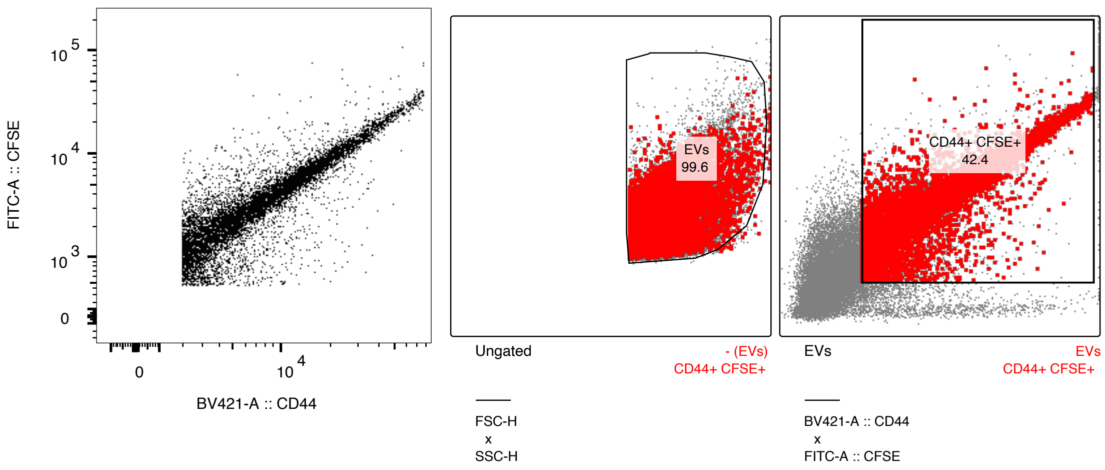

	Sample Name	Freq. of EVs
■	Specimen_001_27-2D (EV staining)_005.fcs	42.4

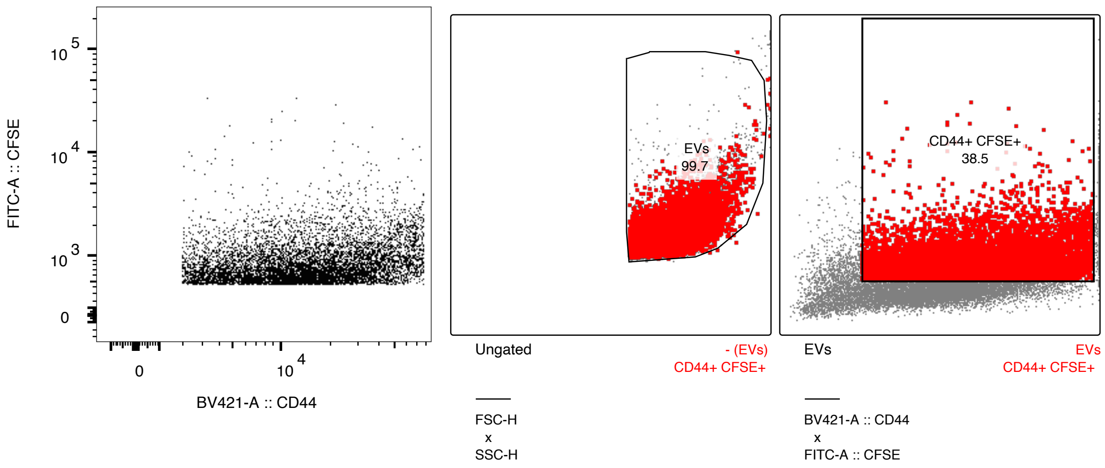

	Sample Name	Freq. of EVs
■	Specimen_001_344-2D (EV staining)_013.fcs	38.5

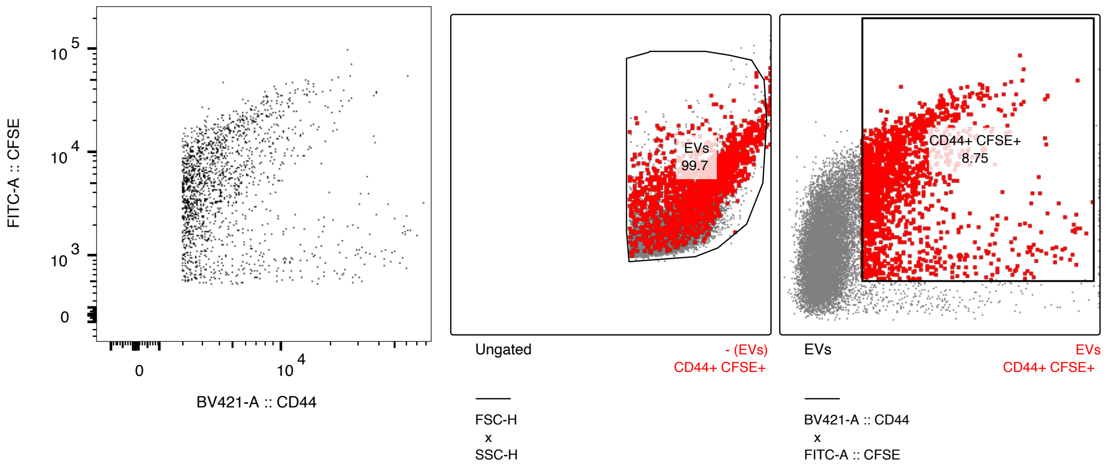

	Sample Name	Freq. of EVs
■	Specimen_001_NP25-2D (EV staining)_021.fcs	8.75

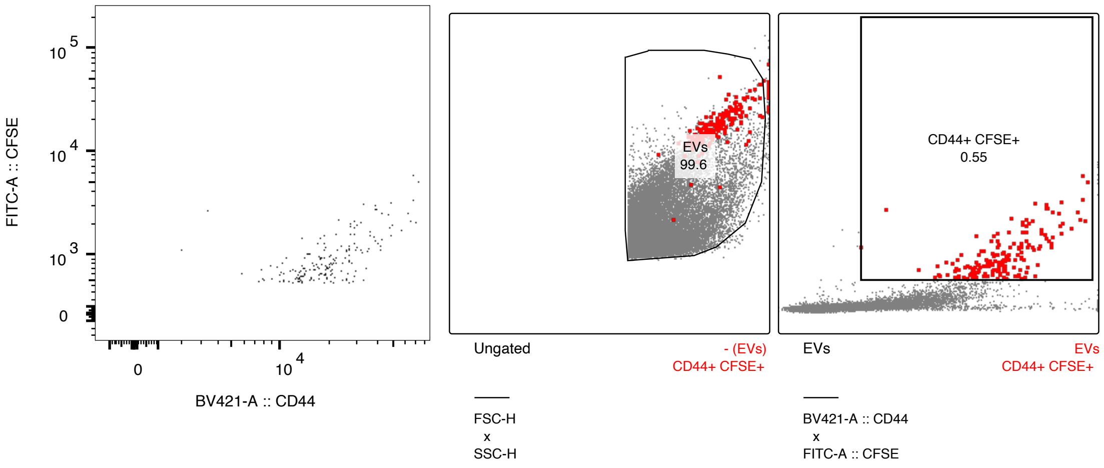

	Sample Name	Freq. of EVs
■	Specimen_001_27-2D (IgG staining)_006.fcs	0.55

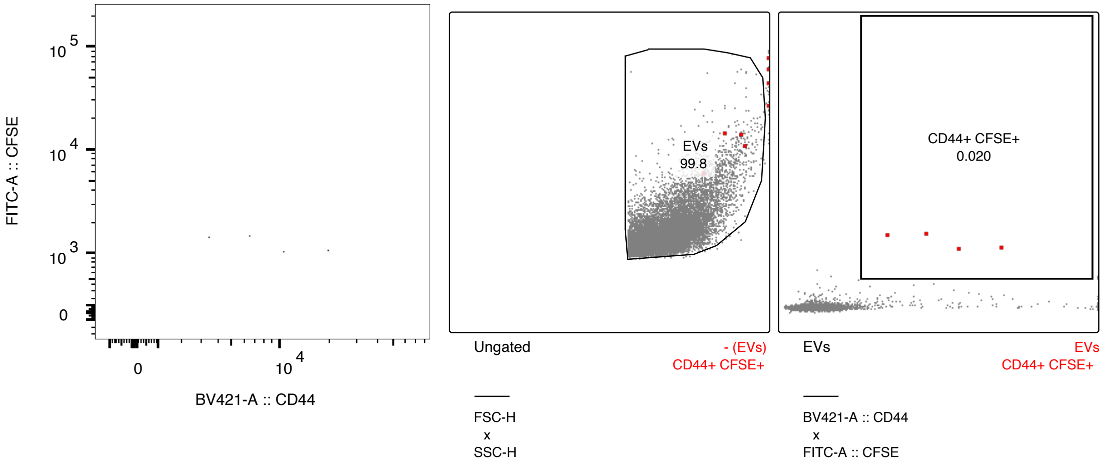

	Sample Name	Freq. of EVs
■	Specimen_001_344-2D (IGG staining)_014.fcs	0.020

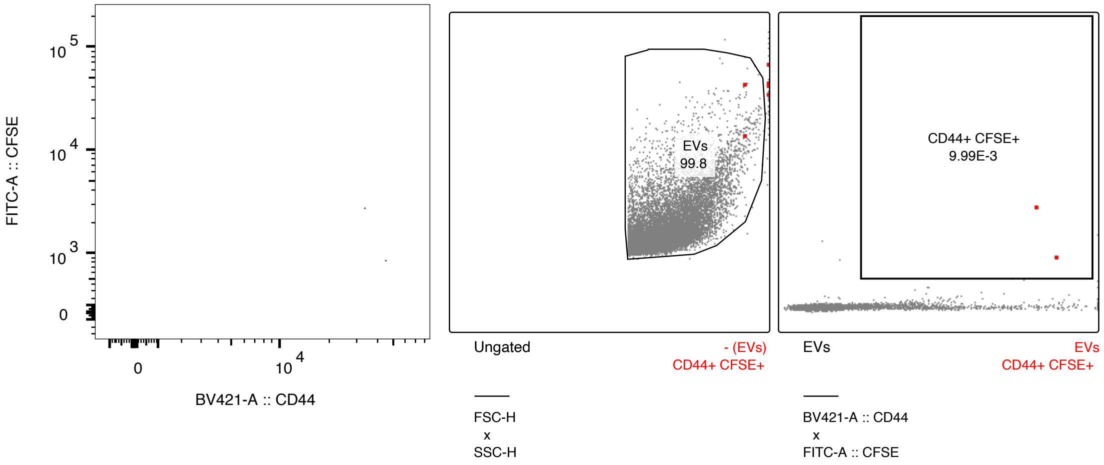

	Sample Name	Freq. of EVs
■	Specimen_001_NP25-2D (IGG staining)_022.fcs	9.99E-3

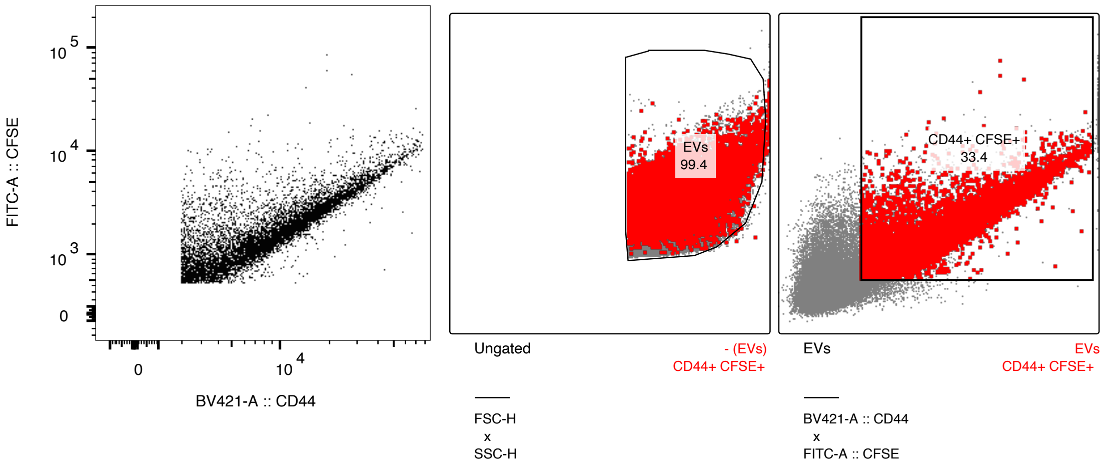

	Sample Name	Freq. of EVs
■	Specimen_001_27-3D (EV staining)_007.fcs	33.4

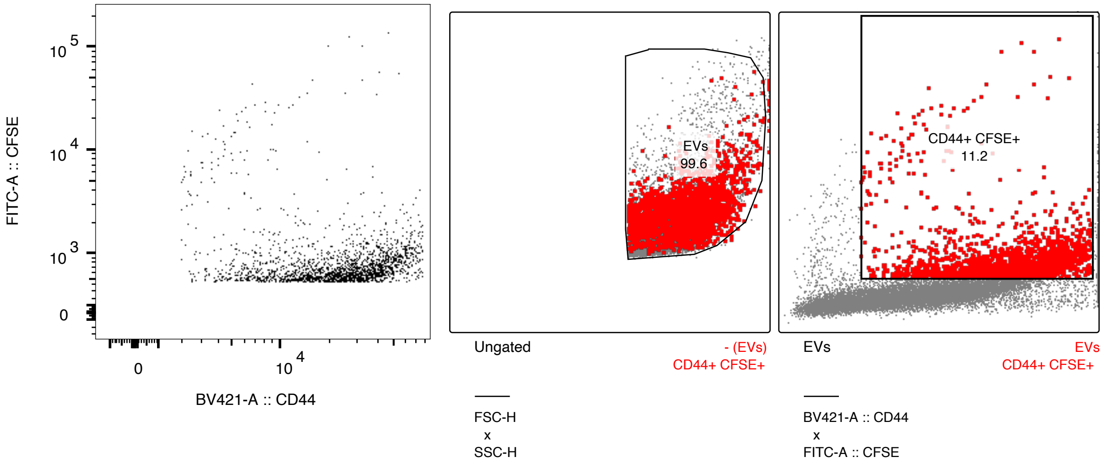

	Sample Name	Freq. of EVs
■	Specimen_001_344-3D (EV staining)_015.fcs	11.2

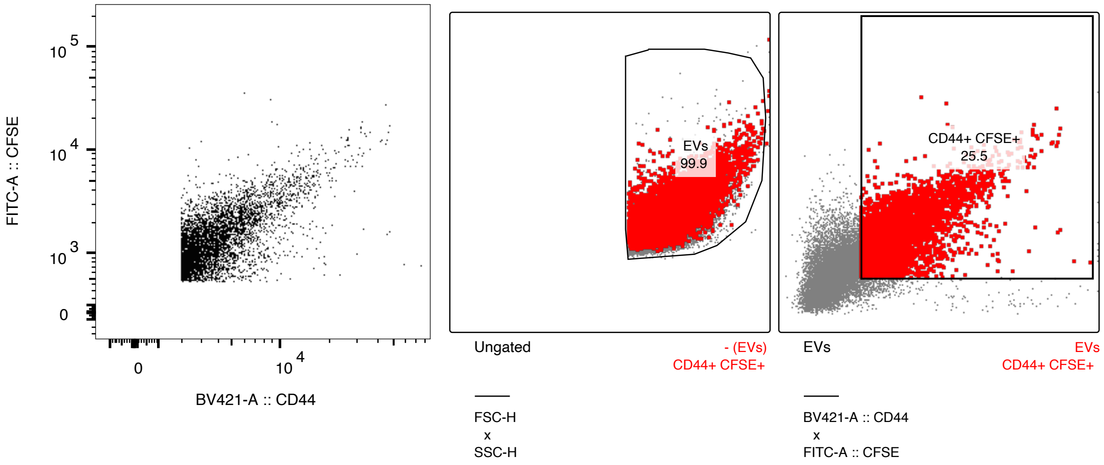

	Sample Name	Freq. of EVs
■	Specimen_001_NP25-3D (EV staining)_023.fcs	25.5

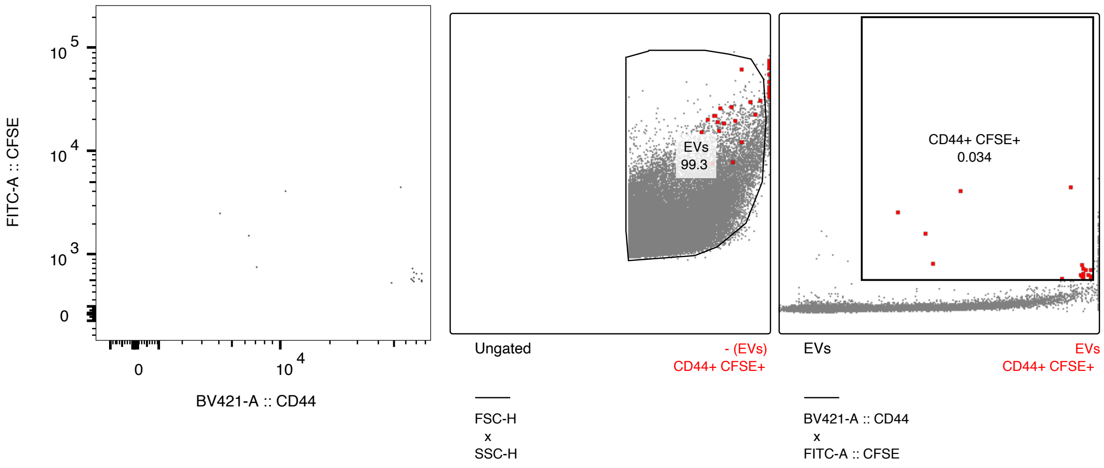

	Sample Name	Freq. of EVs
■	Specimen_001_27-3D (IgG staining)_008.fcs	0.034

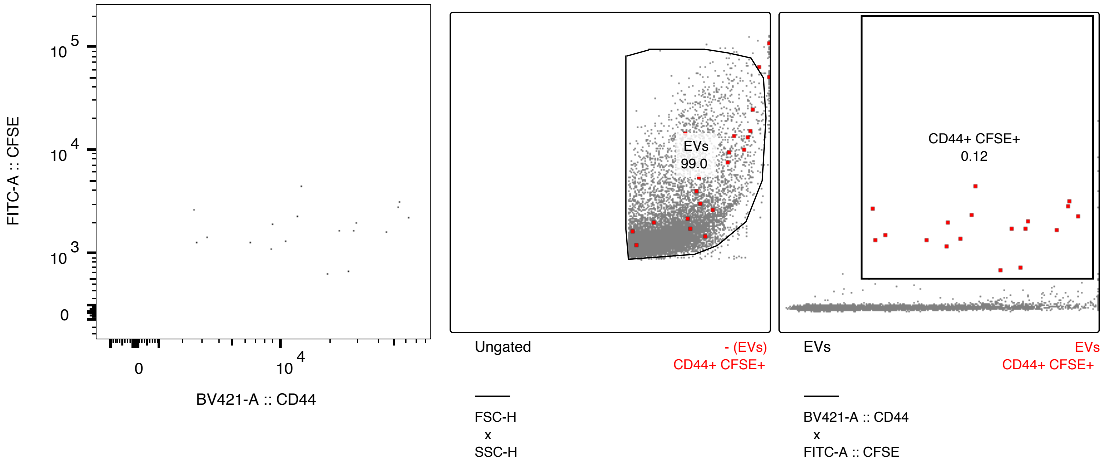

	Sample Name	Freq. of EVs
■	Specimen_001_344-3D (IGG staining)_016.fcs	0.12

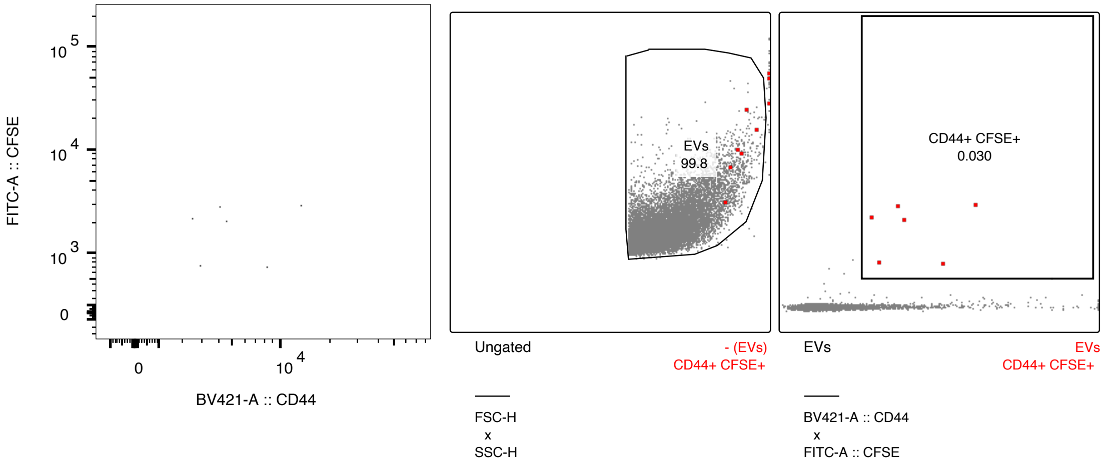

	Sample Name	Freq. of EVs
■	Specimen_001_NP25-3D (IGG staining)_024.fcs	0.030

IA EV surface marker staining

Cell line name in the manuscript	Cell line name in the gating dot plot
BTIC10	21
BTIC13	27
BTIC131	344
BTIC18	57
BTIC129	337
BTIC155	NP25

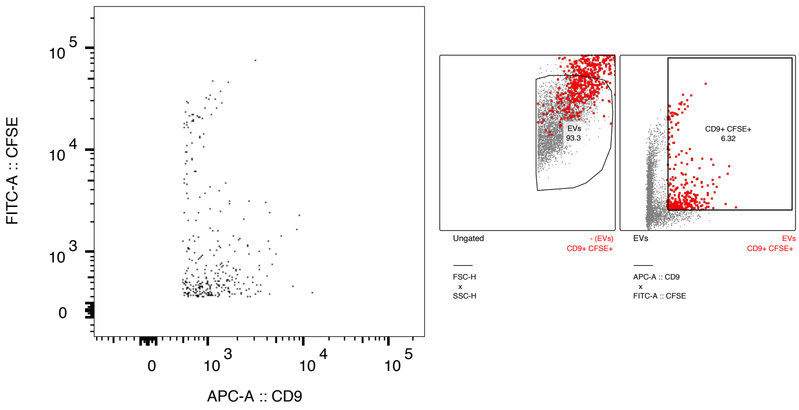

	Sample Name	Freq. of EVs
■	Specimen_001_21-2D (EV staining)_001.fcs	6.32

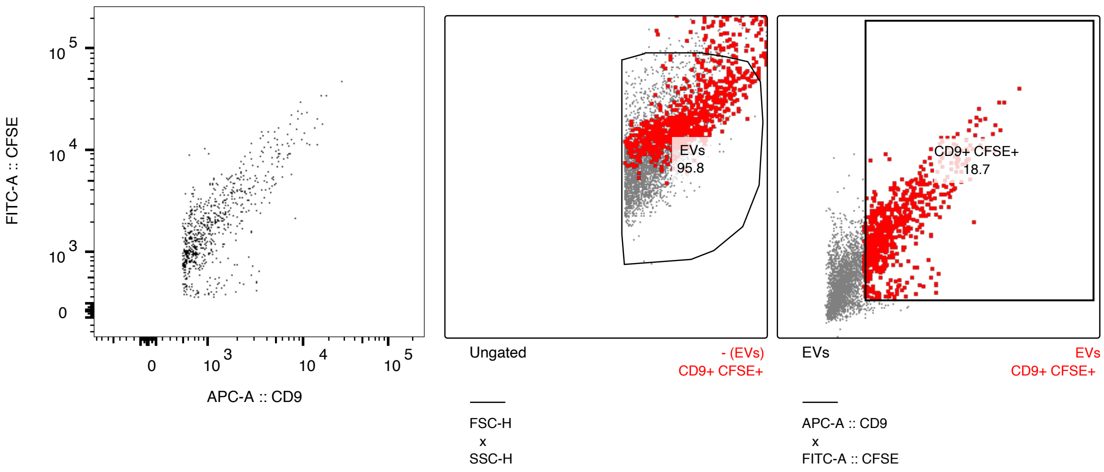

	Sample Name	Freq. of EVs
■	Specimen_001_337-2D (EV staining)_009.fcs	18.7

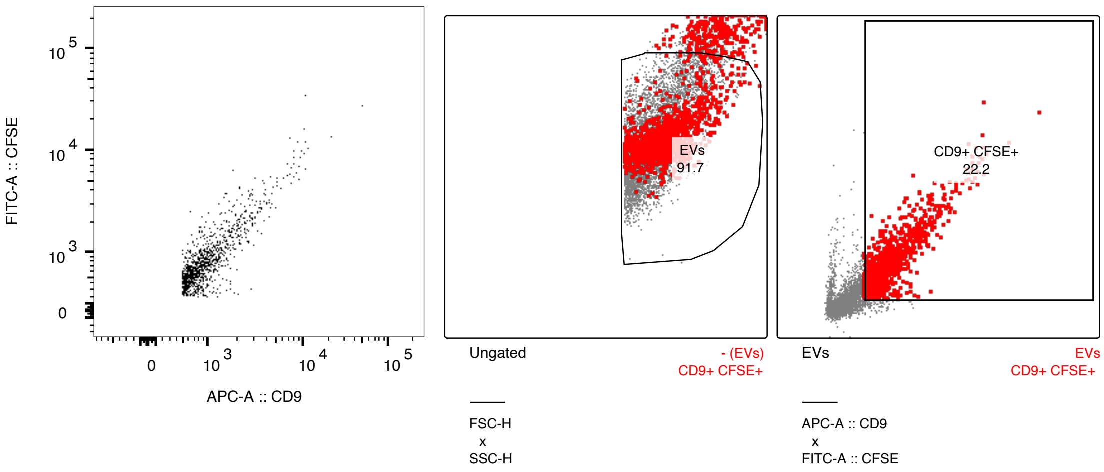

	Sample Name	Freq. of EVs
■	Specimen_001_57-2D (EV staining)_017.fcs	22.2

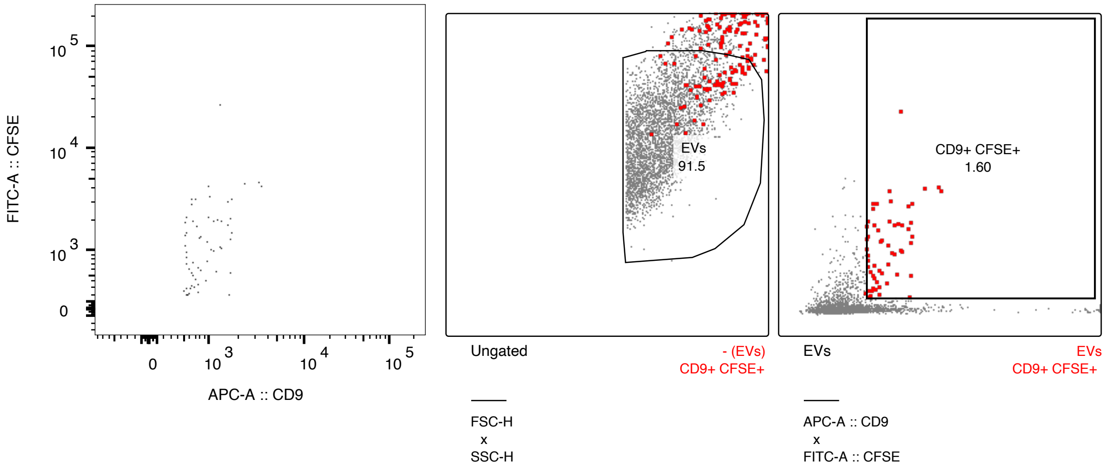

	Sample Name	Freq. of EVs
■	Specimen_001_21-2D (IgG staining)_002.fcs	1.60

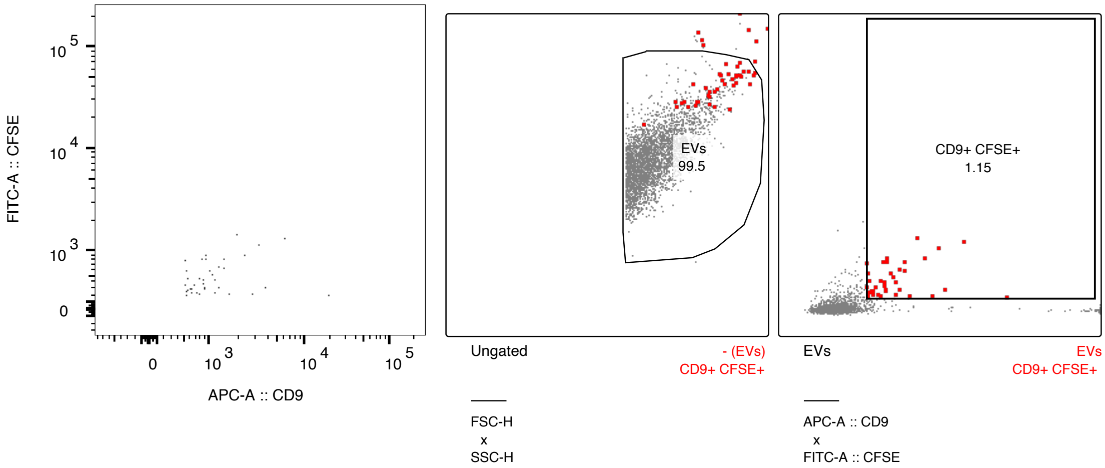

	Sample Name	Freq. of EVs
■	Specimen_001_337-2D (IGG staining)_010.fcs	1.15

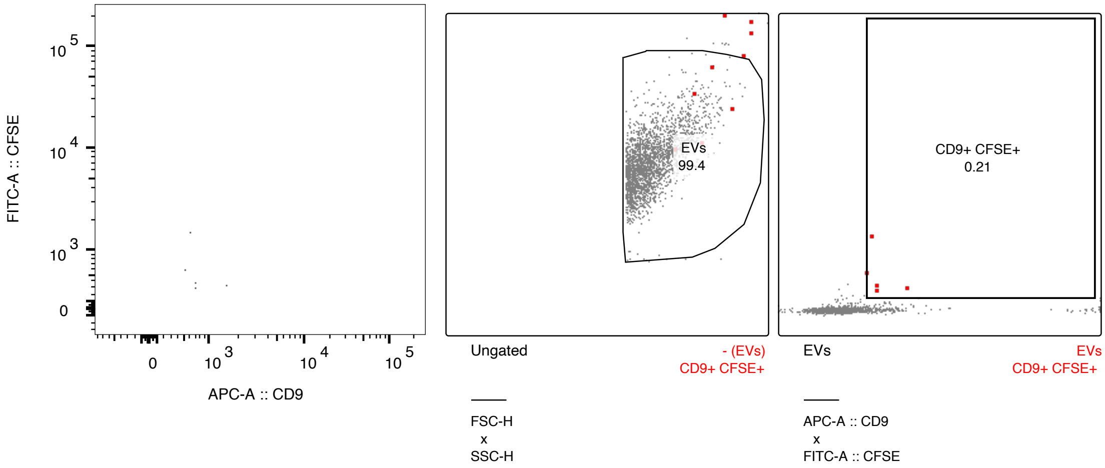

	Sample Name	Freq. of EVs
■	Specimen_001_57-2D (IGG staining)_018.fcs	0.21

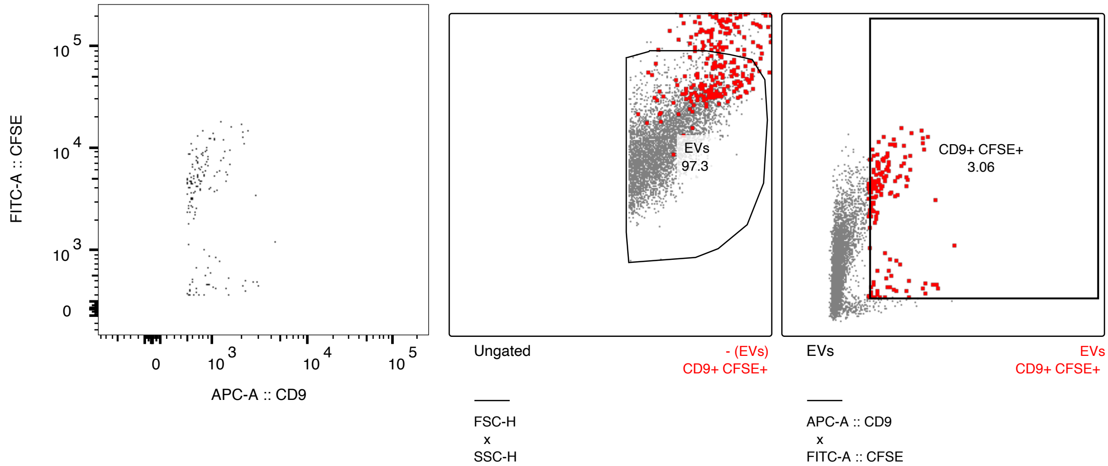

	Sample Name	Freq. of EVs
■	Specimen_001_21-3D (EV staining)_003.fcs	3.06

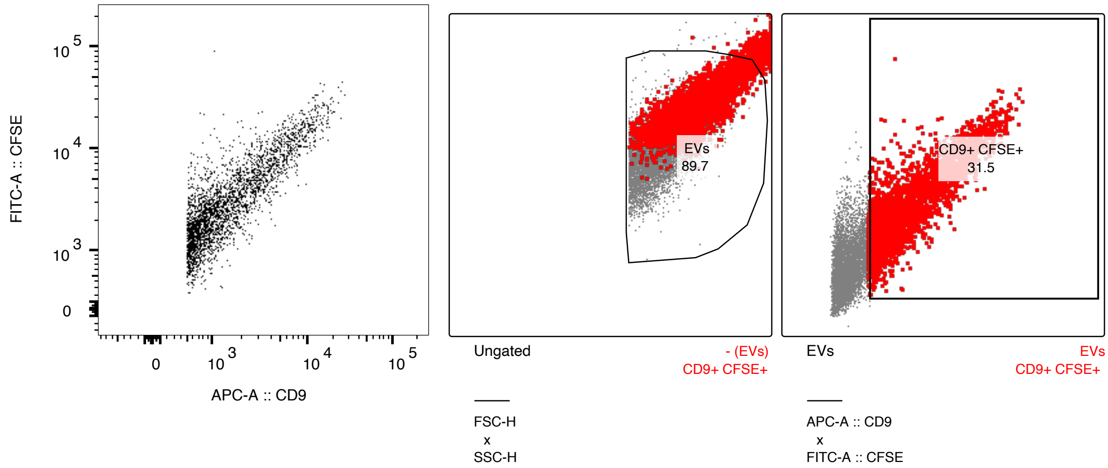

	Sample Name	Freq. of EVs
■	Specimen_001_337-3D (EV staining)_011.fcs	31.5

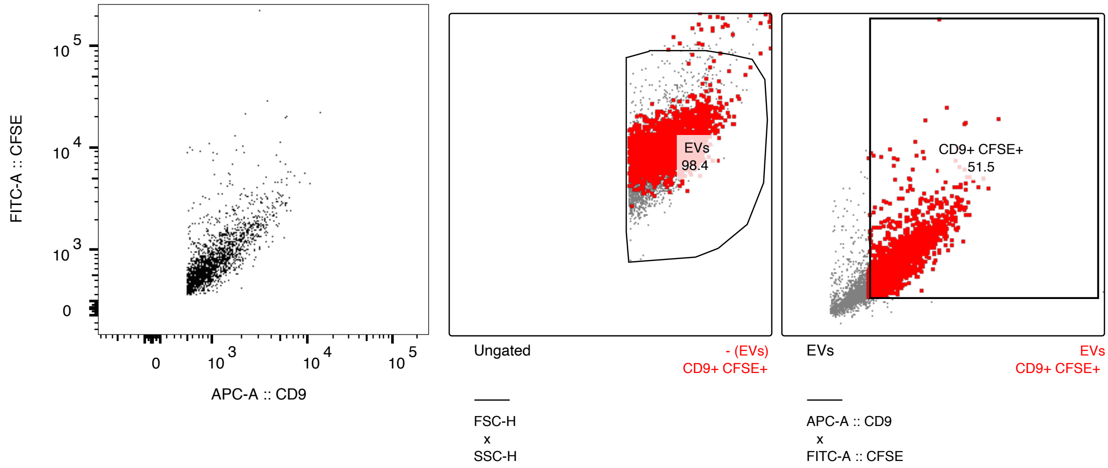

	Sample Name	Freq. of EVs
■	Specimen_001_57-3D (EV staining)_019.fcs	51.5

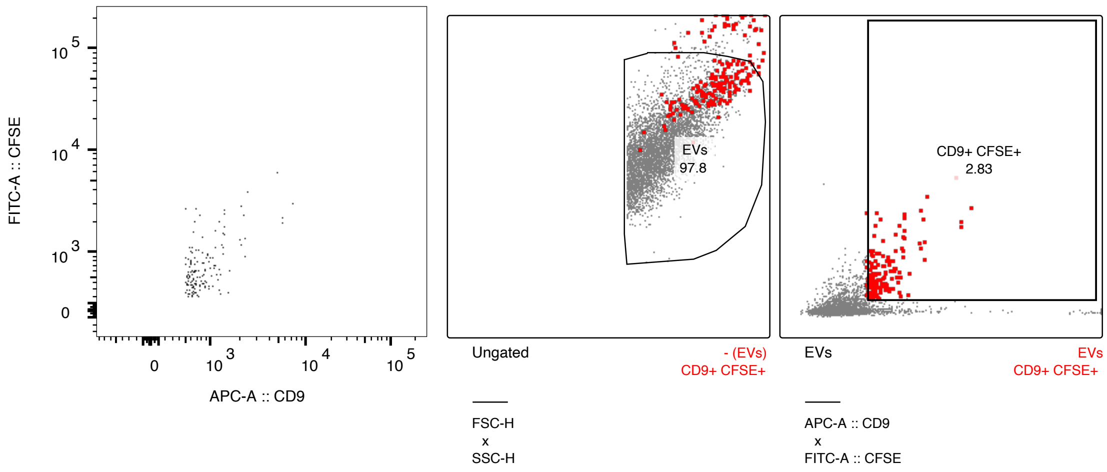

	Sample Name	Freq. of EVs
■	Specimen_001_21-3D (IgG staining)_004.fcs	2.83

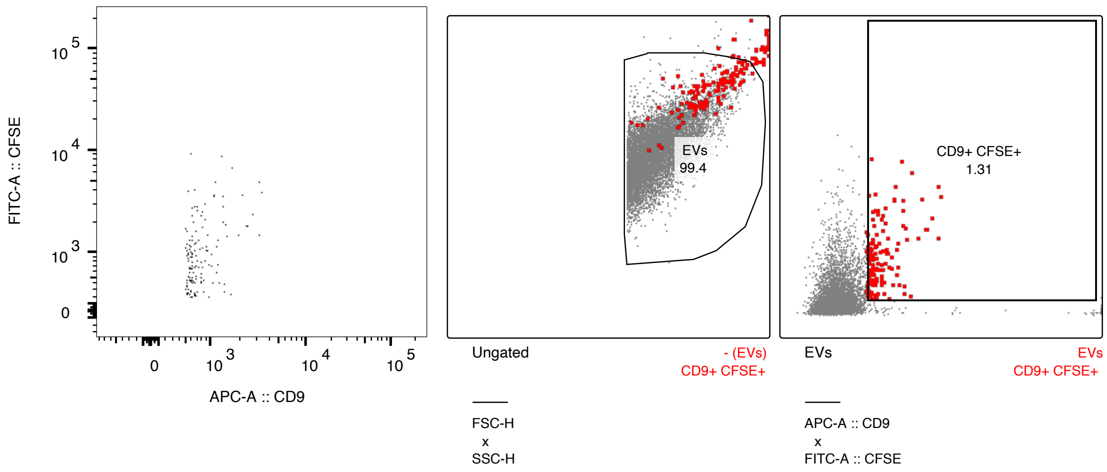

	Sample Name	Freq. of EVs
■	Specimen_001_337-3D (IGG staining)_012.fcs	1.31

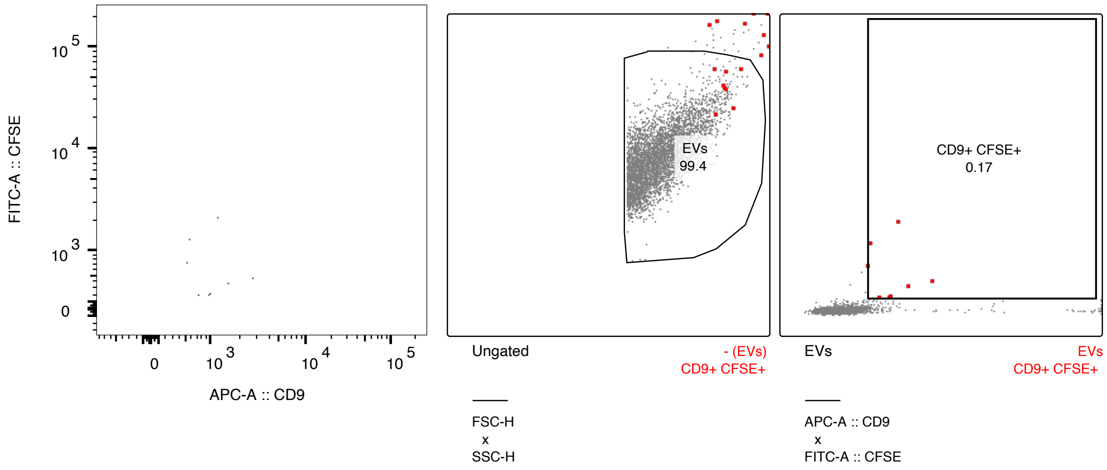

	Sample Name	Freq. of EVs
■	Specimen_001_57-3D (IGG staining)_020.fcs	0.17

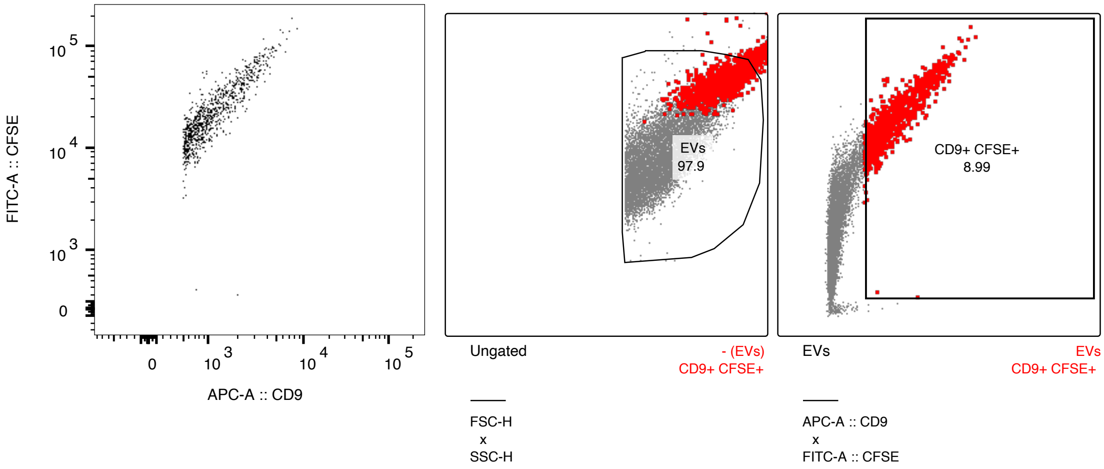

	Sample Name	Freq. of EVs
■	Specimen_001_27-2D (EV staining)_005.fcs	8.99

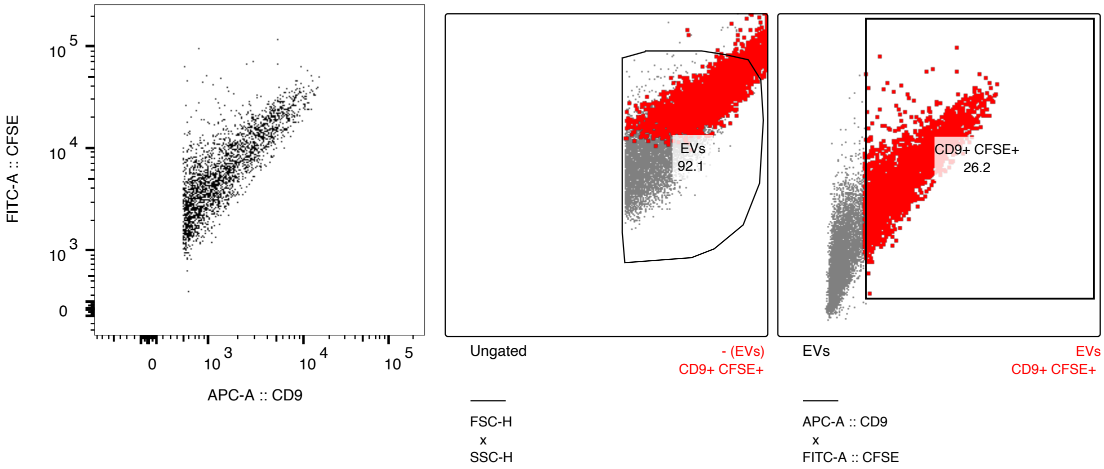

	Sample Name	Freq. of EVs
■	Specimen_001_344-2D (EV staining)_013.fcs	26.2

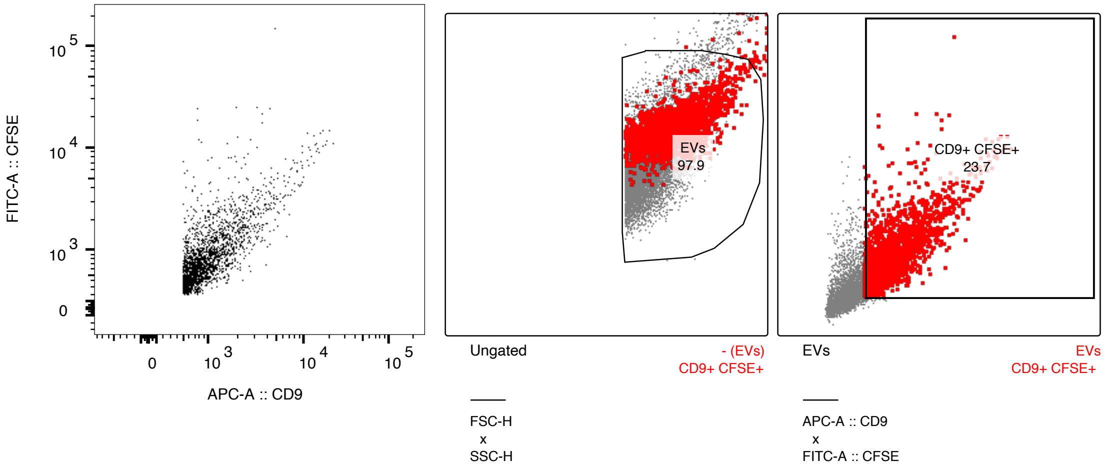

	Sample Name	Freq. of EVs
■	Specimen_001_NP25-2D (EV staining)_021.fcs	23.7

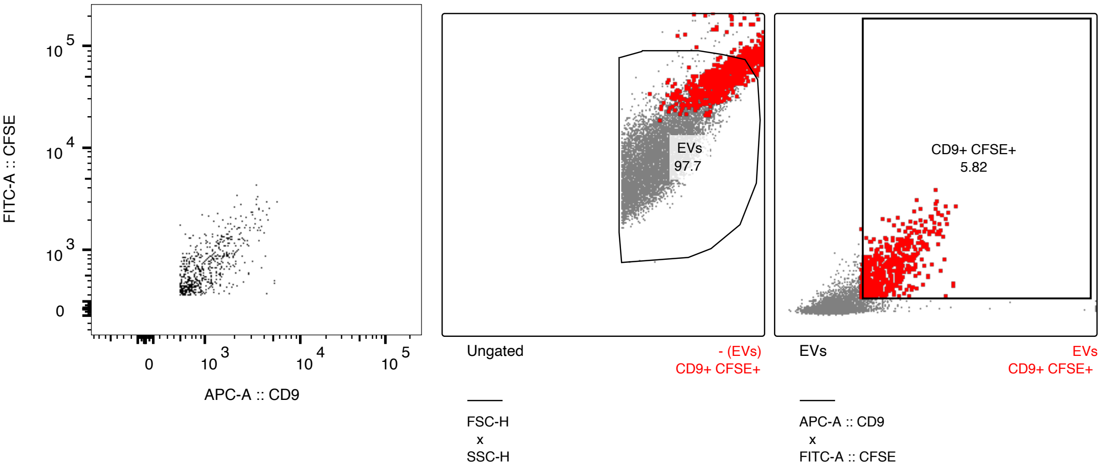

	Sample Name	Freq. of EVs
■	Specimen_001_27-2D (IgG staining)_006.fcs	5.82

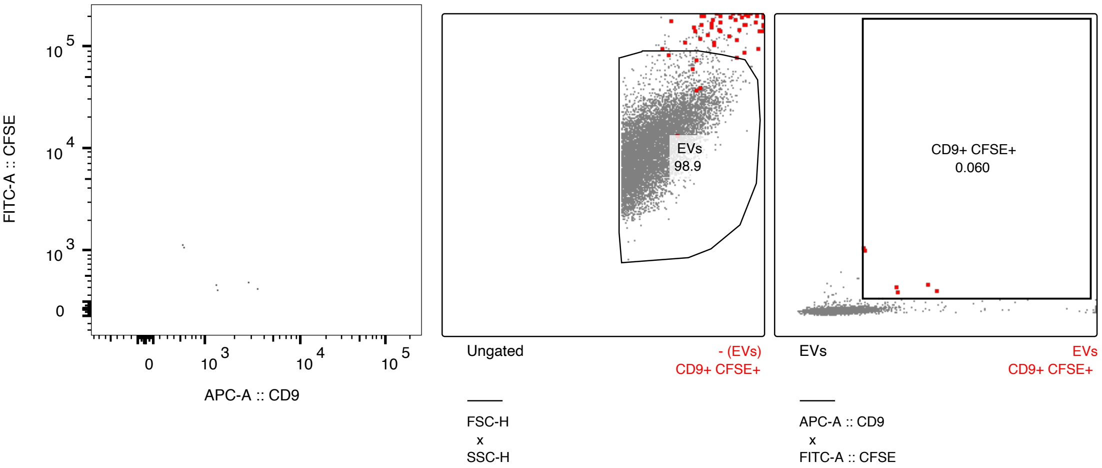

	Sample Name	Freq. of EVs
■	Specimen_001_344-2D (IGG staining)_014.fcs	0.060

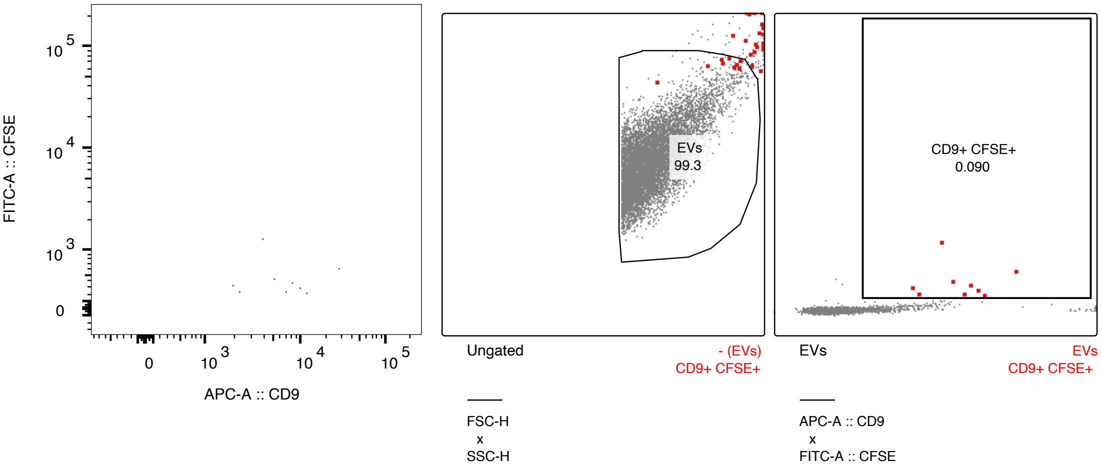

	Sample Name	Freq. of EVs
■	Specimen_001_NP25-2D (IGG staining)_022.fcs	0.090

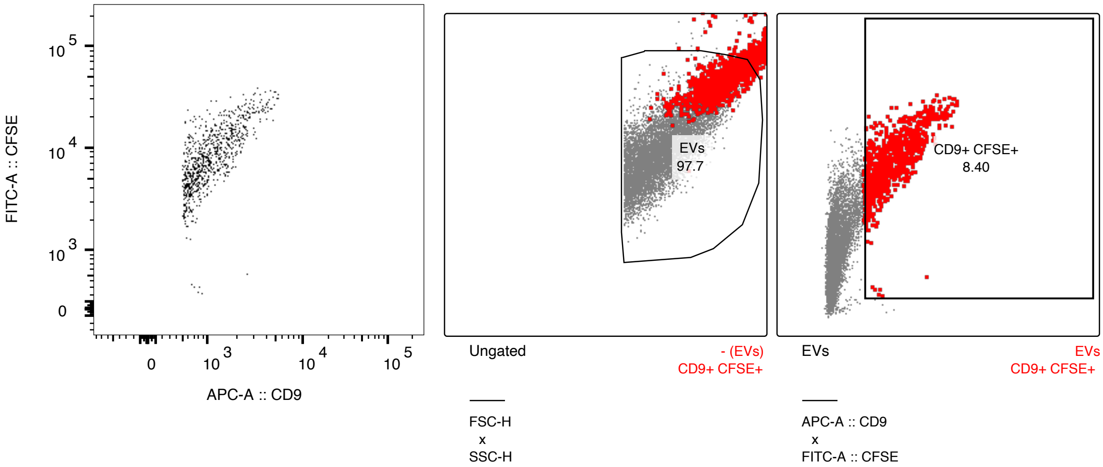

	Sample Name	Freq. of EVs
■	Specimen_001_27-3D (EV staining)_007.fcs	8.40

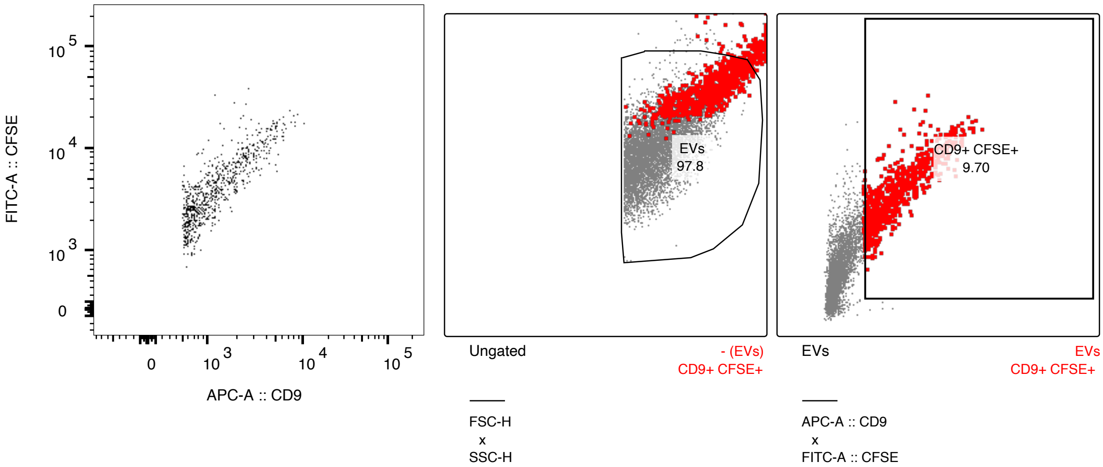

	Sample Name	Freq. of EVs
■	Specimen_001_344-3D (EV staining)_015.fcs	9.70

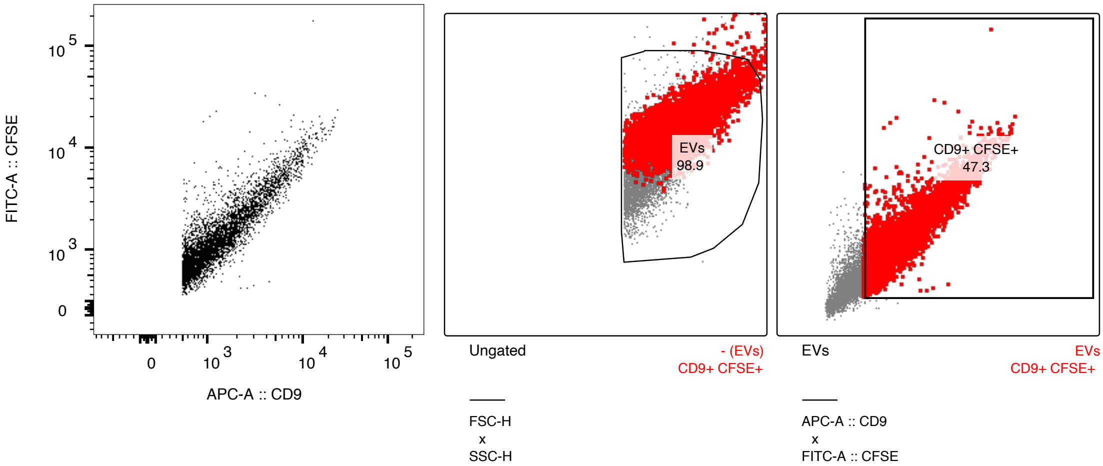

	Sample Name	Freq. of EVs
■	Specimen_001_NP25-3D (EV staining)_023.fcs	47.3

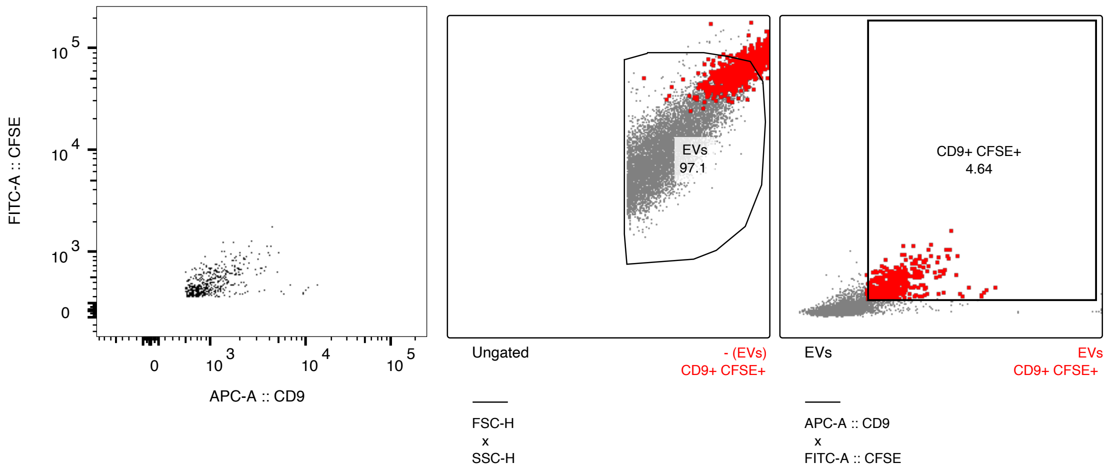

	Sample Name	Freq. of EVs
■	Specimen_001_27-3D (IgG staining)_008.fcs	4.64

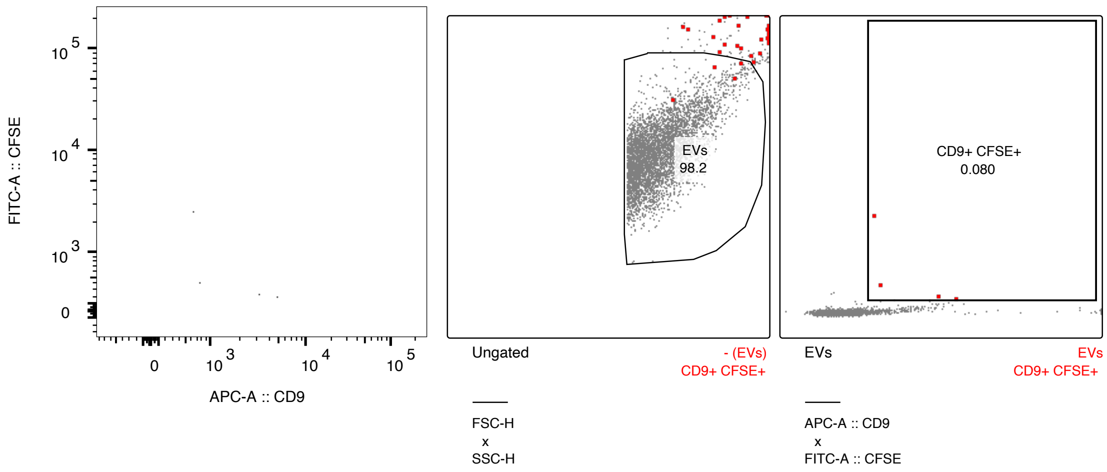

	Sample Name	Freq. of EVs
■	Specimen_001_344-3D (IGG staining)_016.fcs	0.080

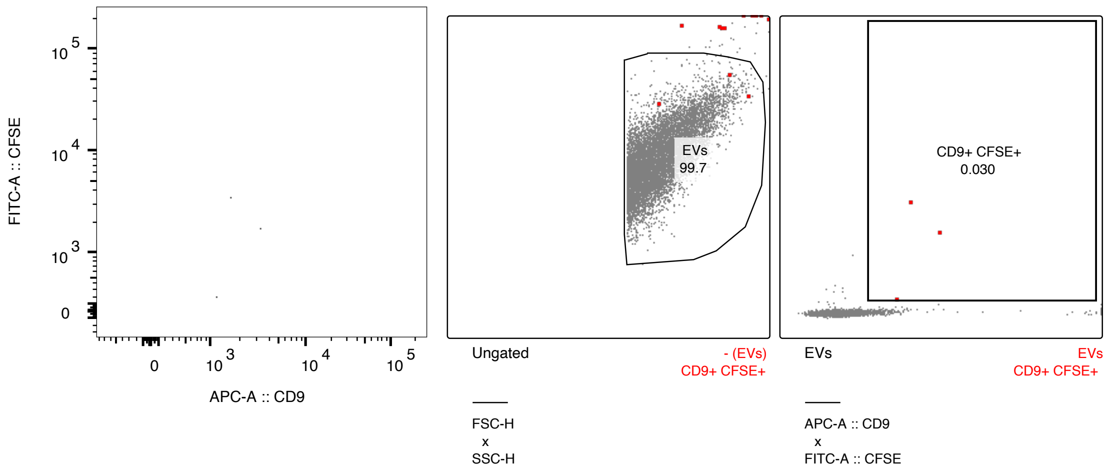

	Sample Name	Freq. of EVs
■	Specimen_001_NP25-3D (IGG staining)_024.fcs	0.030

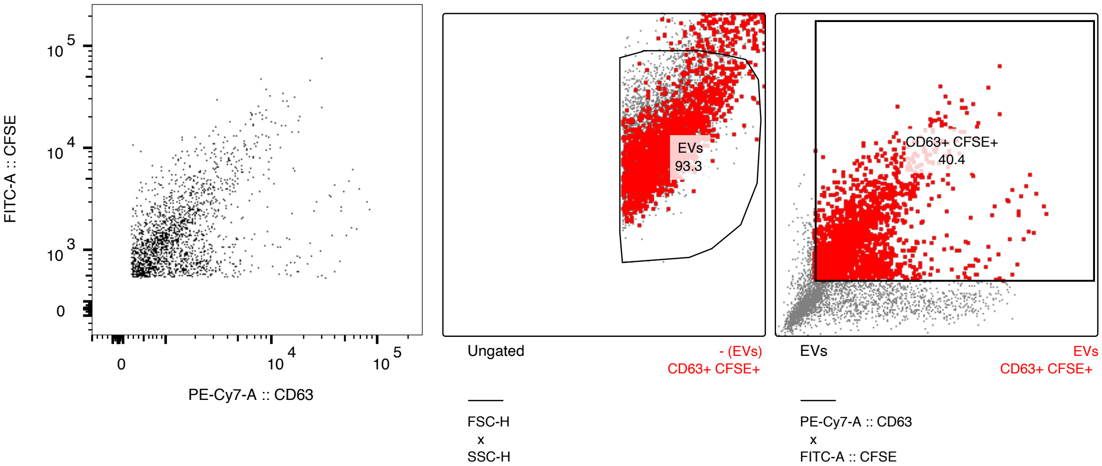

	Sample Name	Freq. of EVs
■	Specimen_001_21-2D (EV staining)_001.fcs	40.4

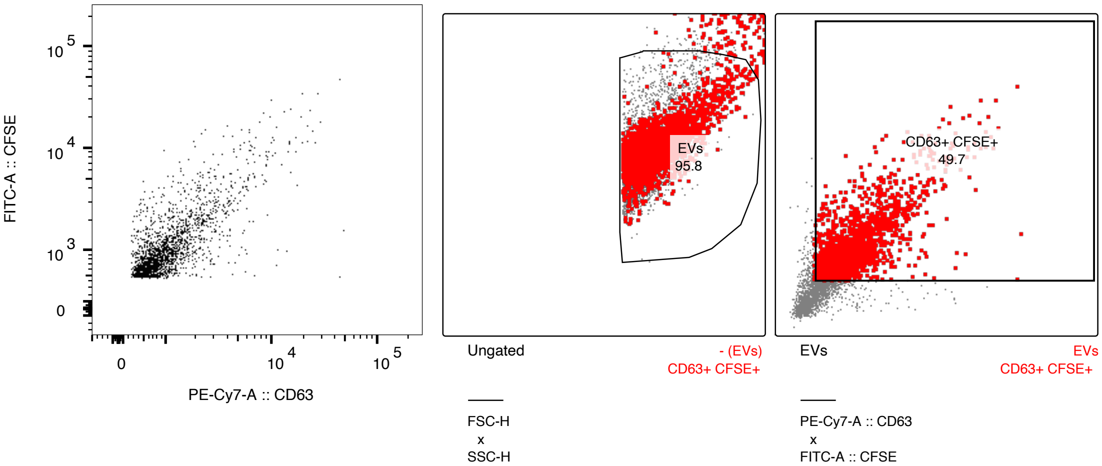

	Sample Name	Freq. of EVs
■	Specimen_001_337-2D (EV staining)_009.fcs	49.7

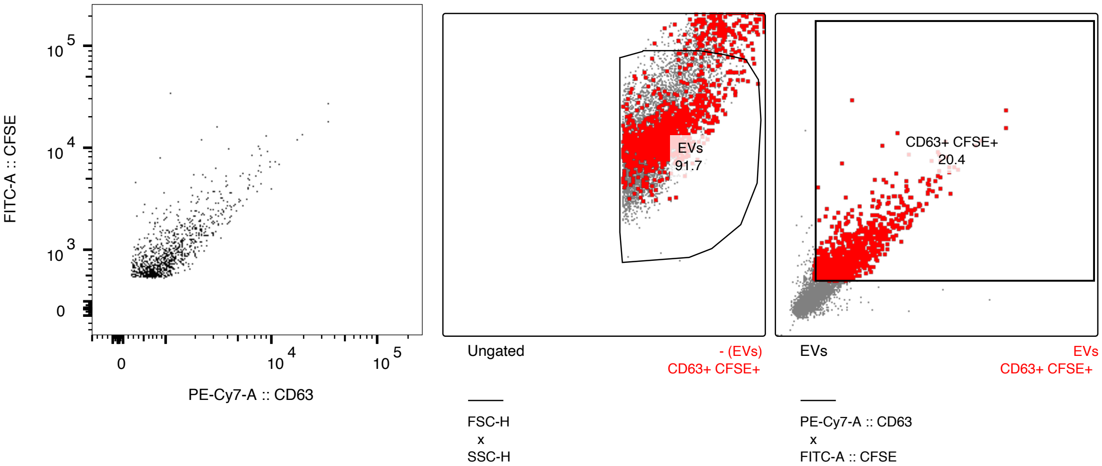

	Sample Name	Freq. of EVs
■	Specimen_001_57-2D (EV staining)_017.fcs	20.4

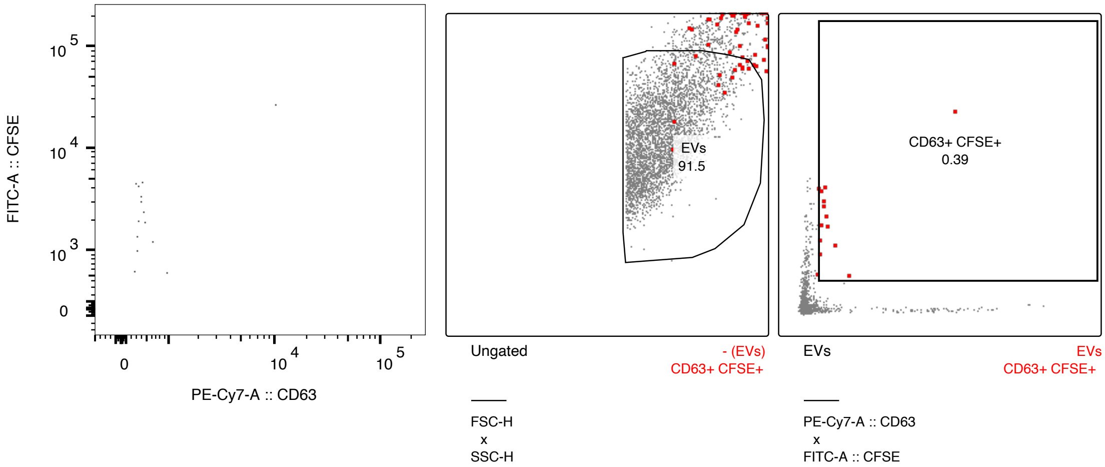

	Sample Name	Freq. of EVs
■	Specimen_001_21-2D (IgG staining)_002.fcs	0.39

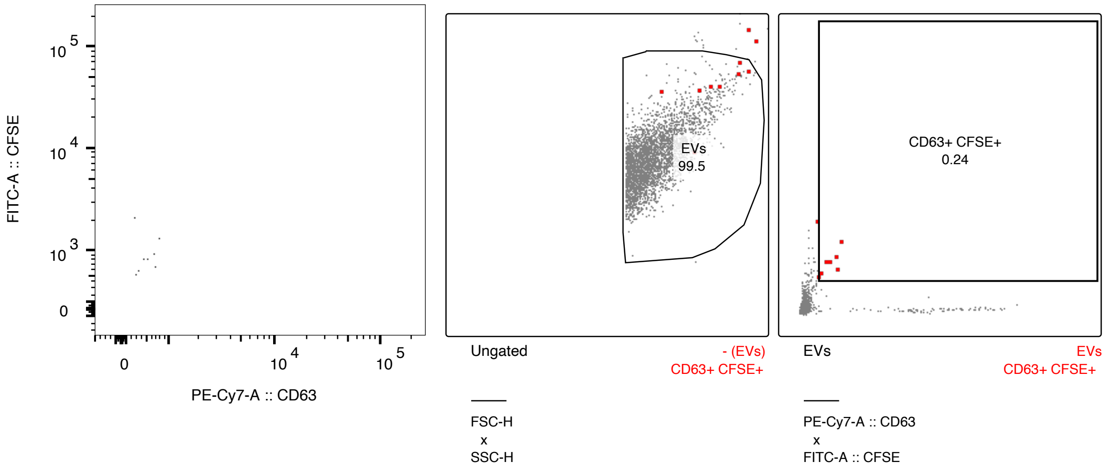

	Sample Name	Freq. of EVs
■	Specimen_001_337-2D (IGG staining)_010.fcs	0.24

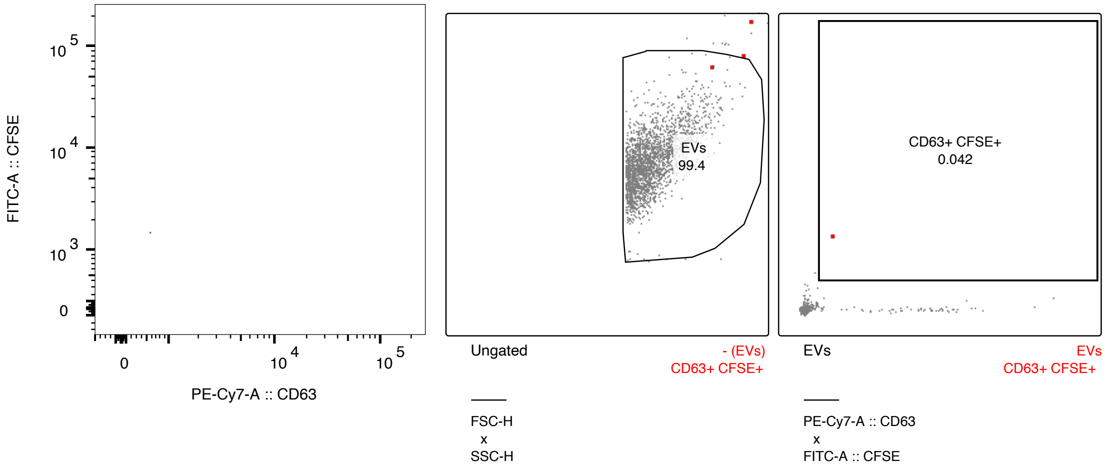

	Sample Name	Freq. of EVs
■	Specimen_001_57-2D (IGG staining)_018.fcs	0.042

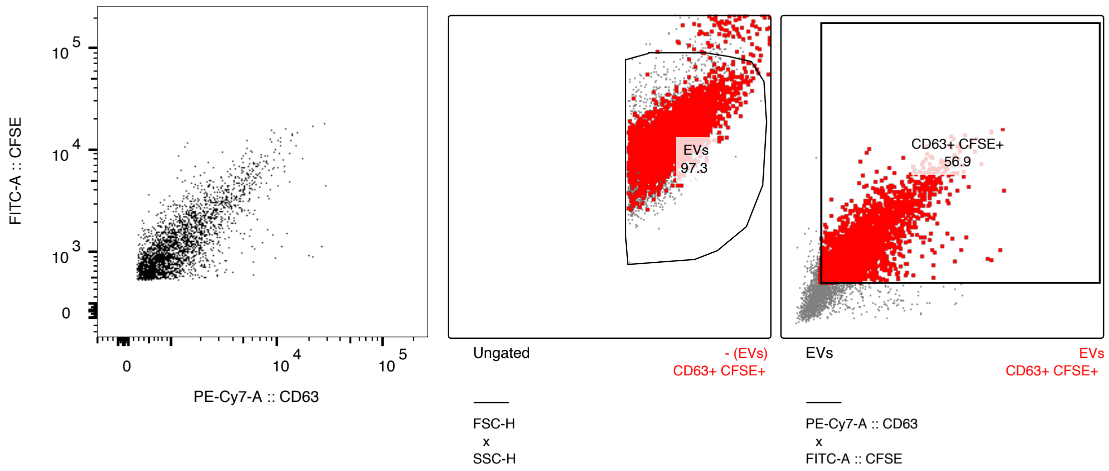

	Sample Name	Freq. of EVs
■	Specimen_001_21-3D (EV staining)_003.fcs	56.9

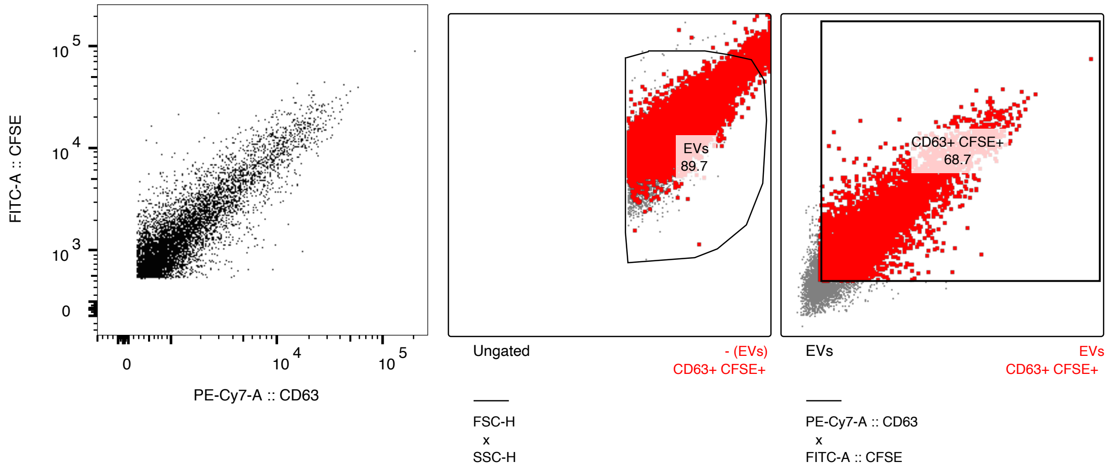

	Sample Name	Freq. of EVs
■	Specimen_001_337-3D (EV staining)_011.fcs	68.7

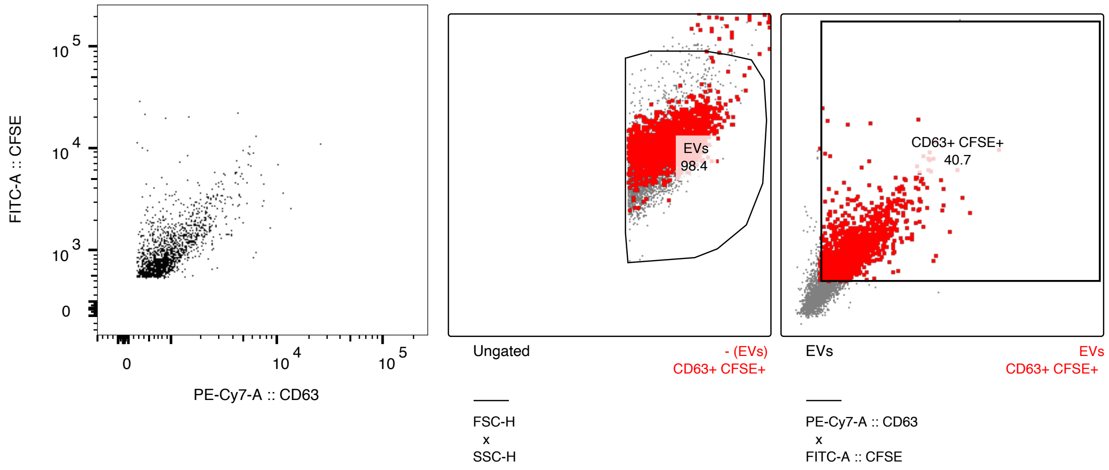

	Sample Name	Freq. of EVs
■	Specimen_001_57-3D (EV staining)_019.fcs	40.7

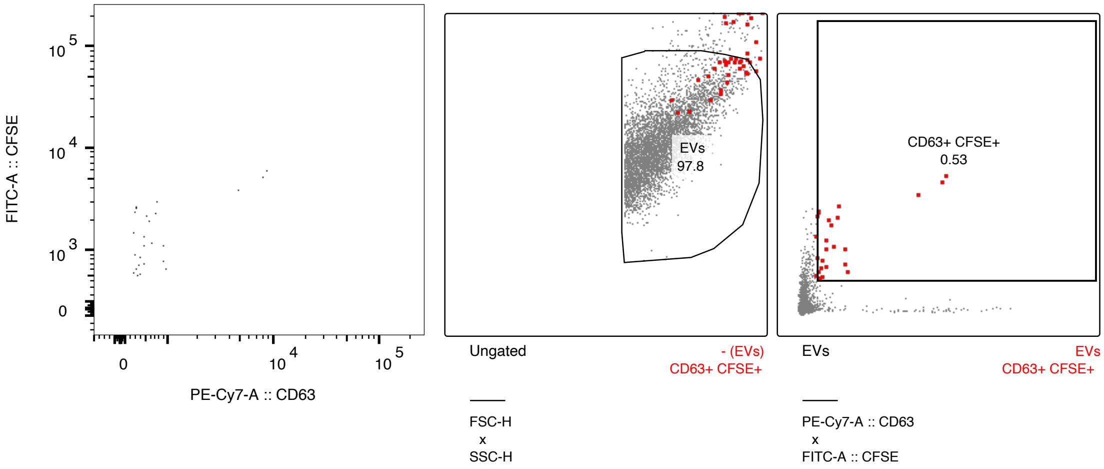

	Sample Name	Freq. of EVs
■	Specimen_001_21-3D (IgG staining)_004.fcs	0.53

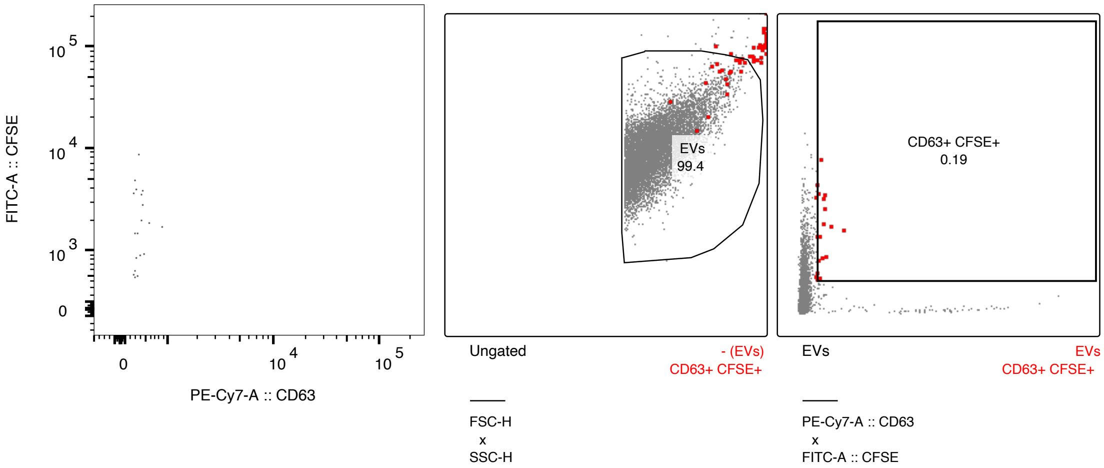

	Sample Name	Freq. of EVs
■	Specimen_001_337-3D (IGG staining)_012.fcs	0.19

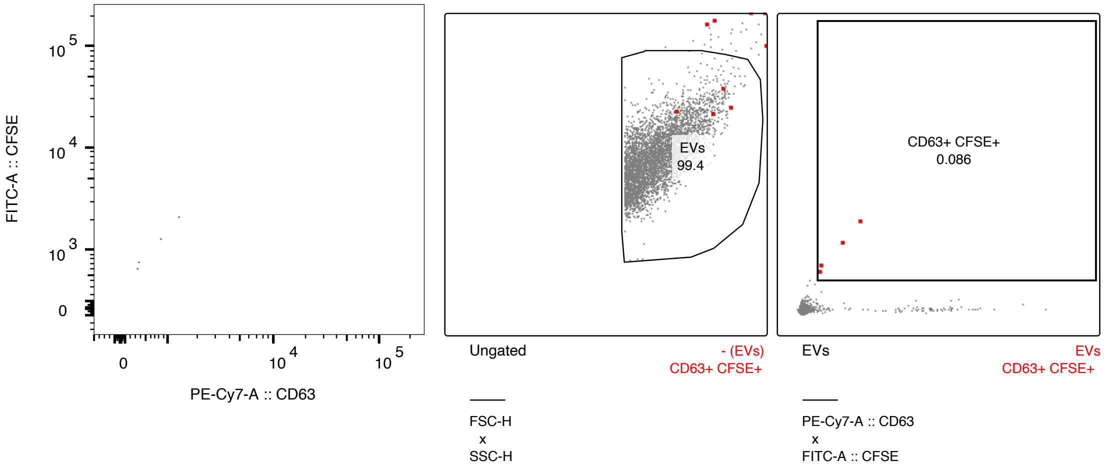

	Sample Name	Freq. of EVs
■	Specimen_001_57-3D (IGG staining)_020.fcs	0.086

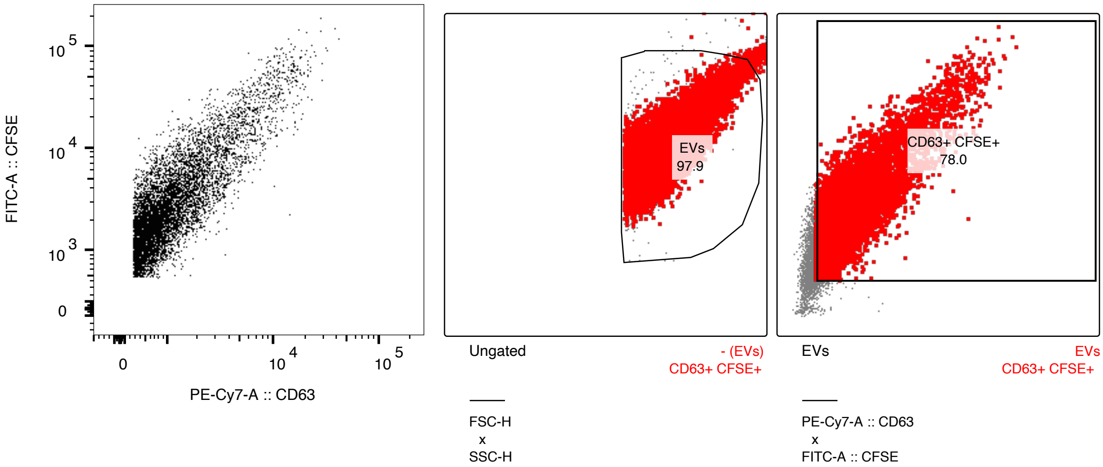

	Sample Name	Freq. of EVs
■	Specimen_001_27-2D (EV staining)_005.fcs	78.0

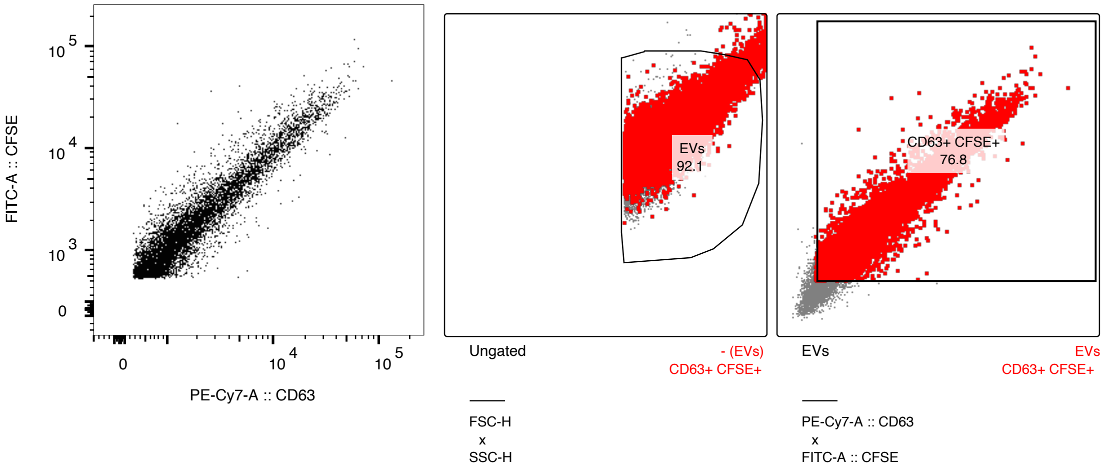

	Sample Name	Freq. of EVs
■	Specimen_001_344-2D (EV staining)_013.fcs	76.8

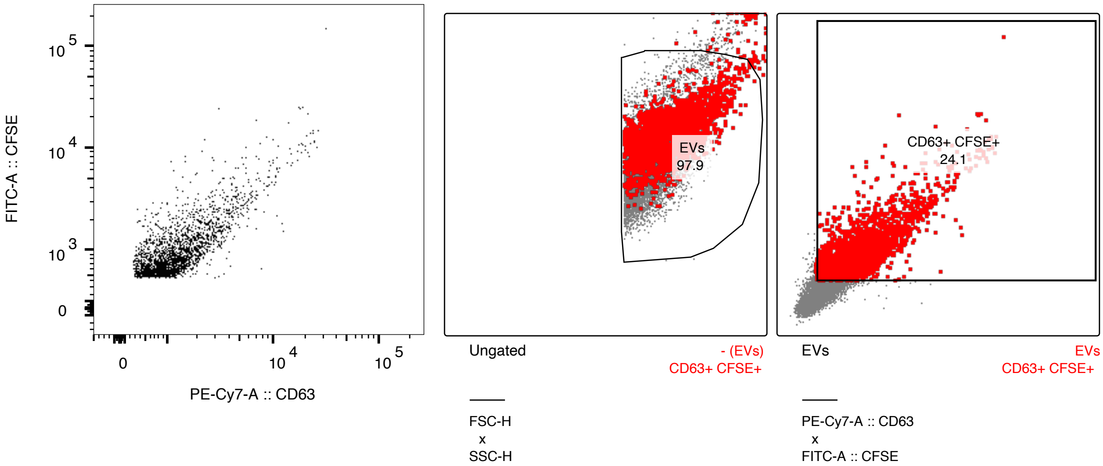

	Sample Name	Freq. of EVs
■	Specimen_001_NP25-2D (EV staining)_021.fcs	24.1

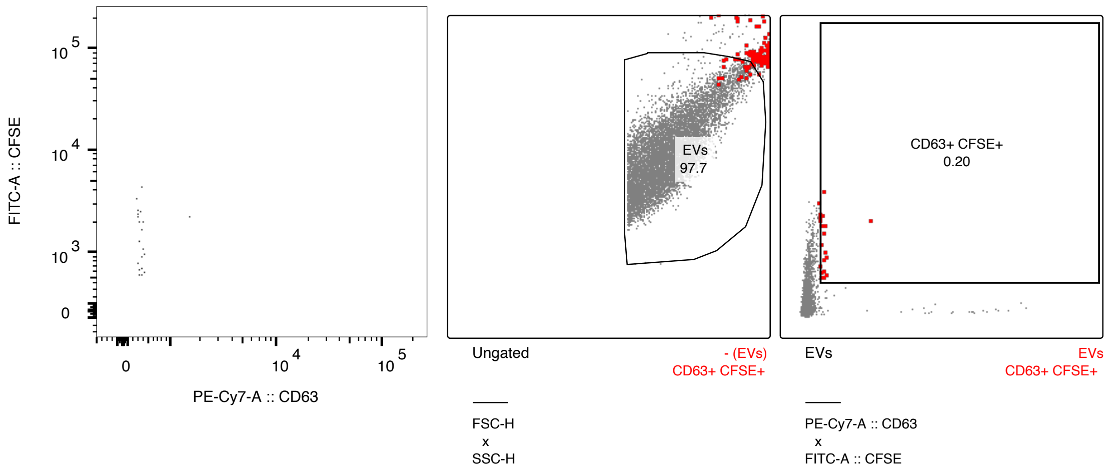

	Sample Name	Freq. of EVs
■	Specimen_001_27-2D (IgG staining)_006.fcs	0.20

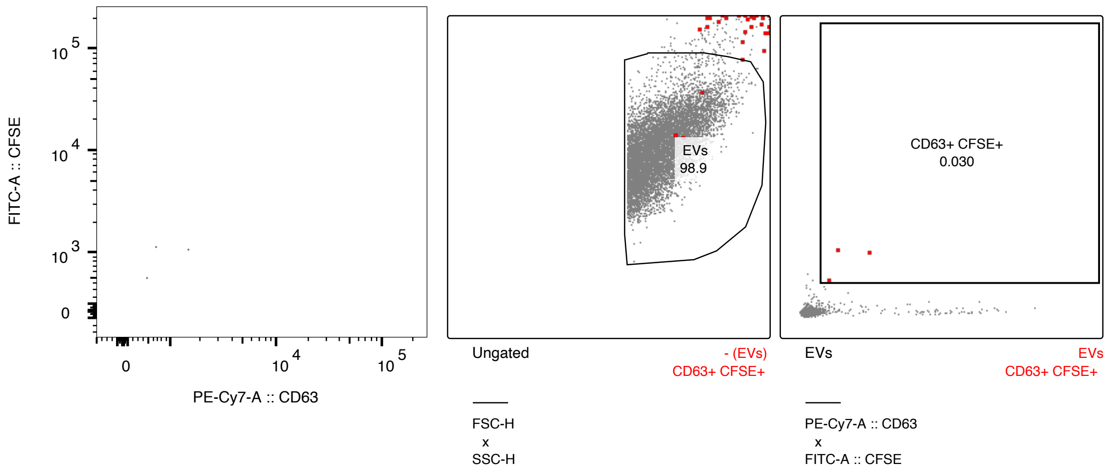

	Sample Name	Freq. of EVs
■	Specimen_001_344-2D (IGG staining)_014.fcs	0.030

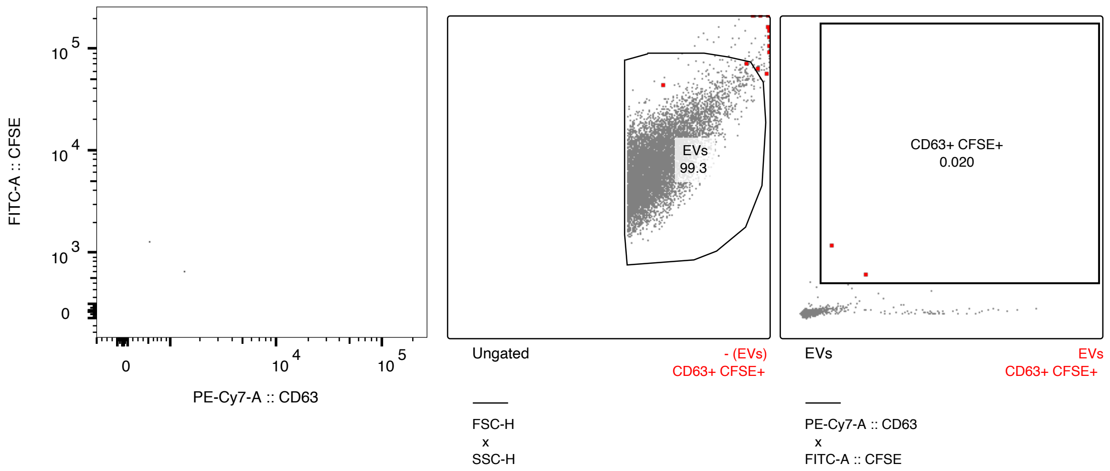

	Sample Name	Freq. of EVs
■	Specimen_001_NP25-2D (IGG staining)_022.fcs	0.020

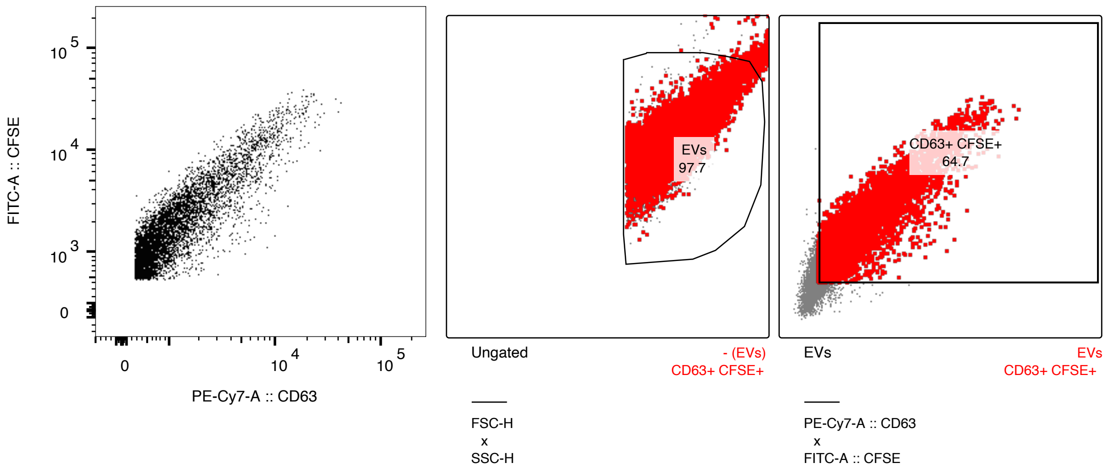

	Sample Name	Freq. of EVs
■	Specimen_001_27-3D (EV staining)_007.fcs	64.7

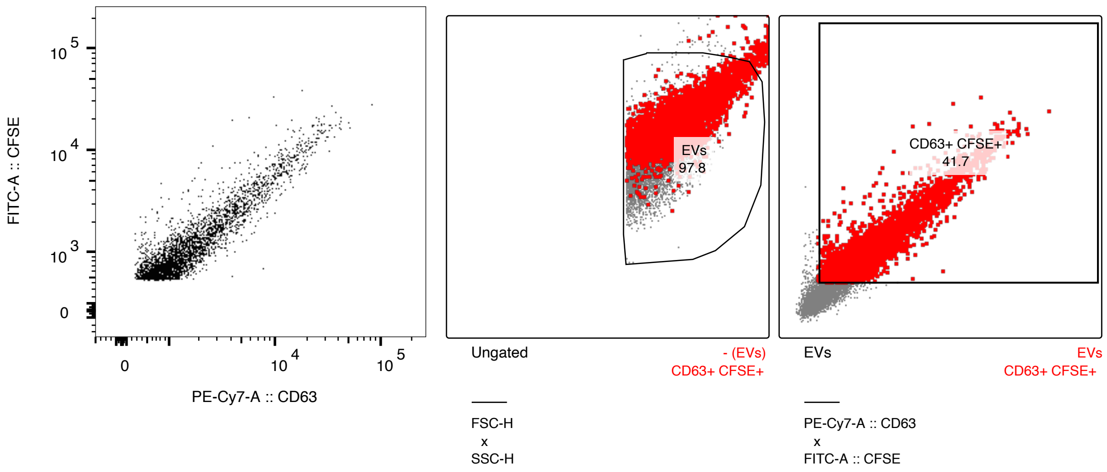

	Sample Name	Freq. of EVs
■	Specimen_001_344-3D (EV staining)_015.fcs	41.7

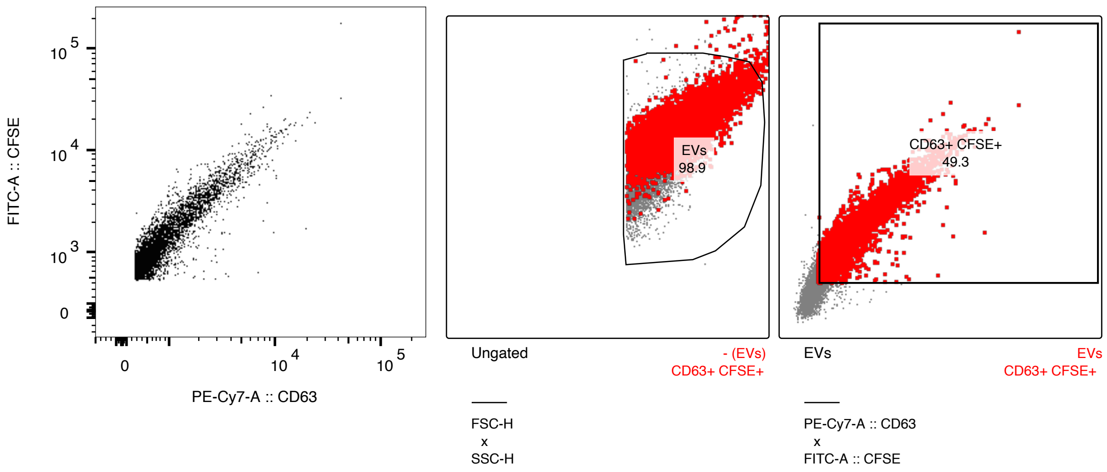

	Sample Name	Freq. of EVs
■	Specimen_001_NP25-3D (EV staining)_023.fcs	49.3

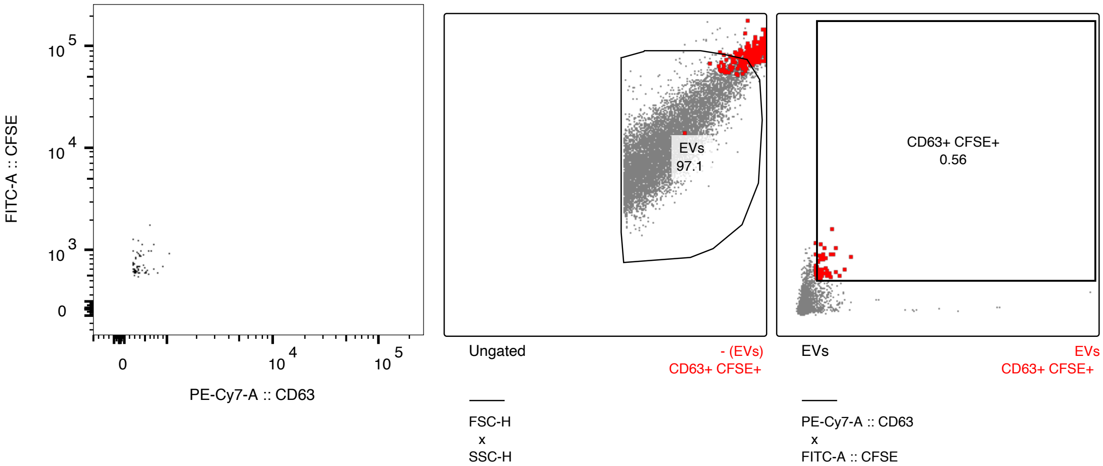

	Sample Name	Freq. of EVs
■	Specimen_001_27-3D (IgG staining)_008.fcs	0.56

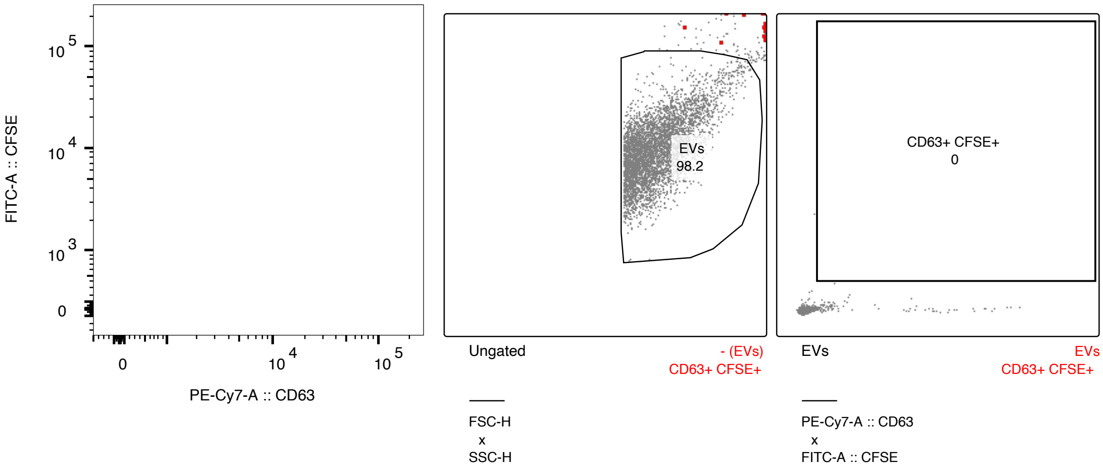

	Sample Name	Freq. of EVs
■	Specimen_001_344-3D (IGG staining)_016.fcs	0

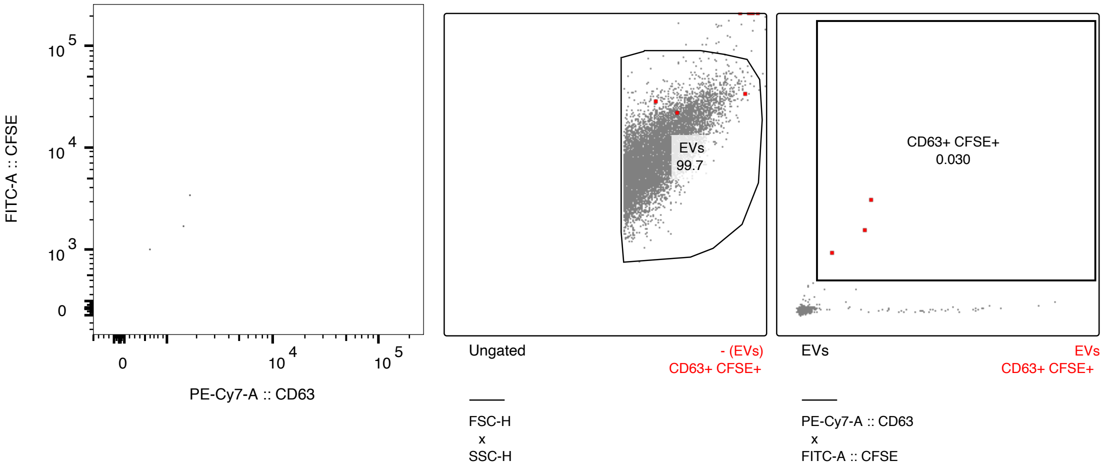

	Sample Name	Freq. of EVs
■	Specimen_001_NP25-3D (IGG staining)_024.fcs	0.030

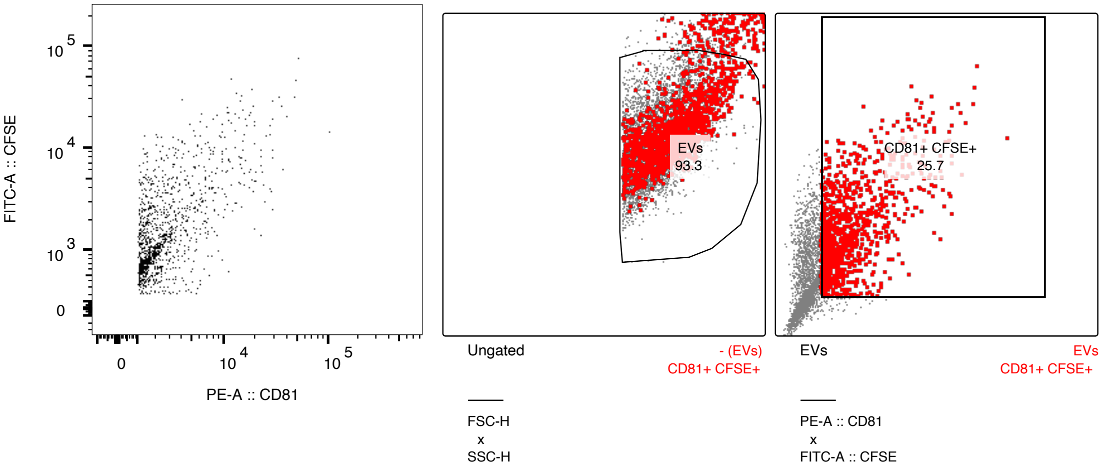

	Sample Name	Freq. of EVs
■	Specimen_001_21-2D (EV staining)_001.fcs	25.7

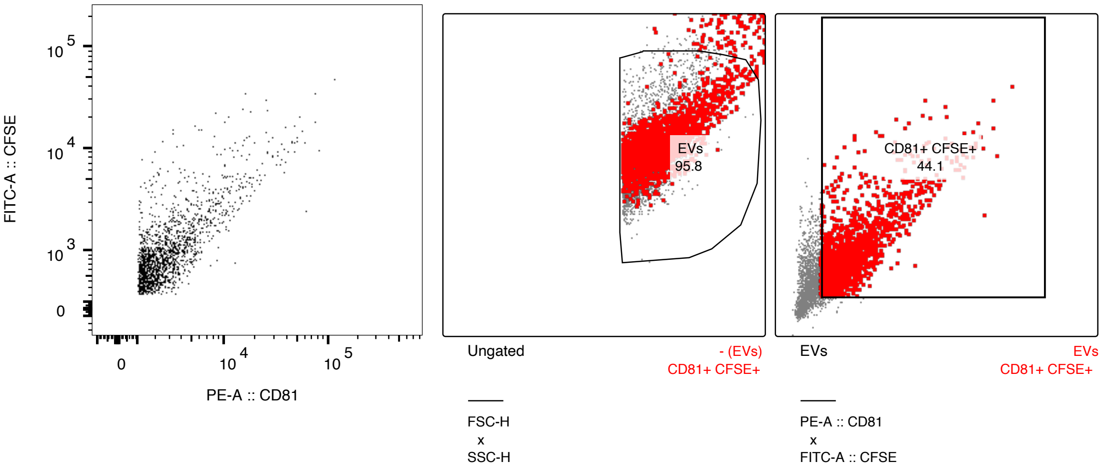

	Sample Name	Freq. of EVs
■	Specimen_001_337-2D (EV staining)_009.fcs	44.1

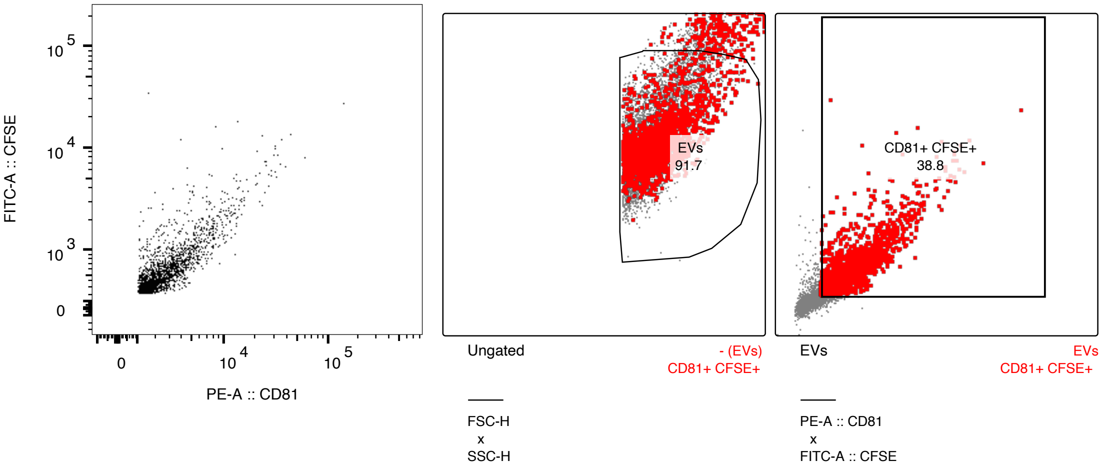

	Sample Name	Freq. of EVs
■	Specimen_001_57-2D (EV staining)_017.fcs	38.8

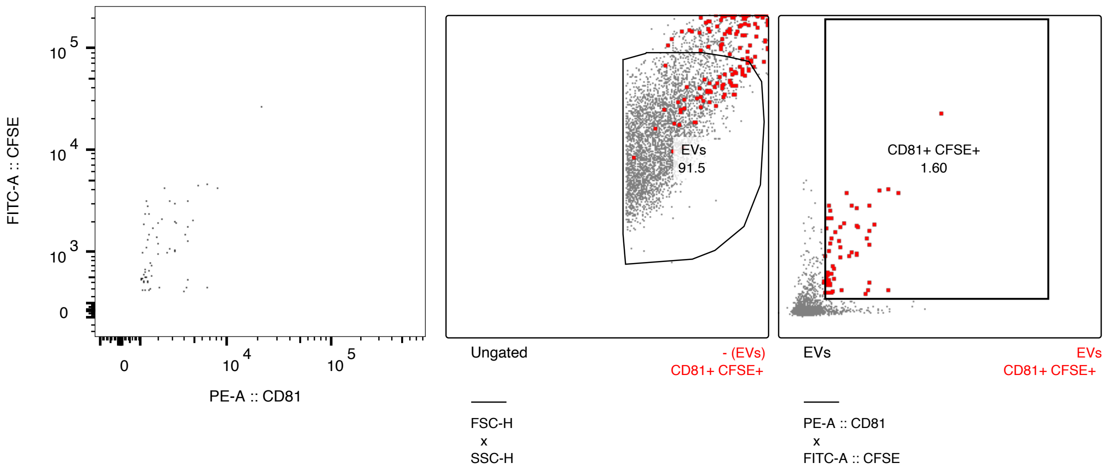

	Sample Name	Freq. of EVs
■	Specimen_001_21-2D (IgG staining)_002.fcs	1.60

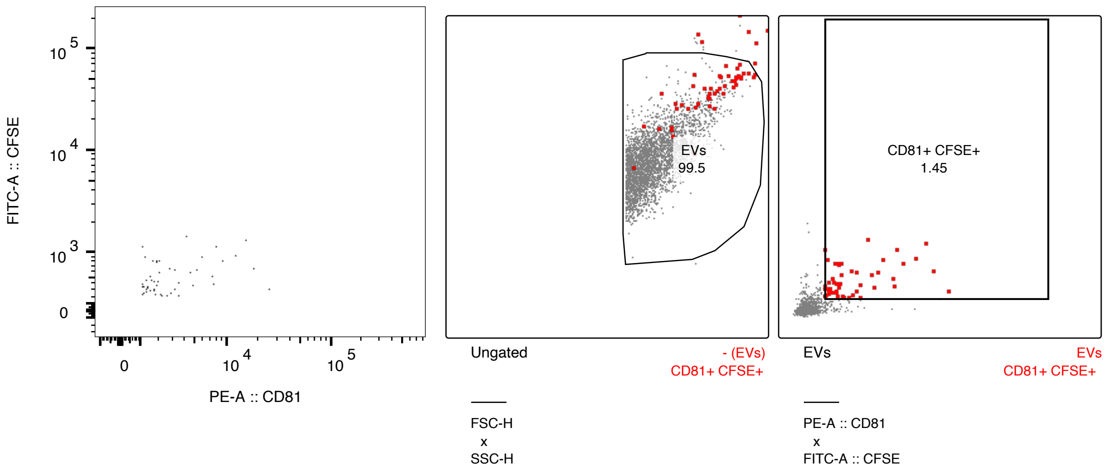

	Sample Name	Freq. of EVs
■	Specimen_001_337-2D (IGG staining)_010.fcs	1.45

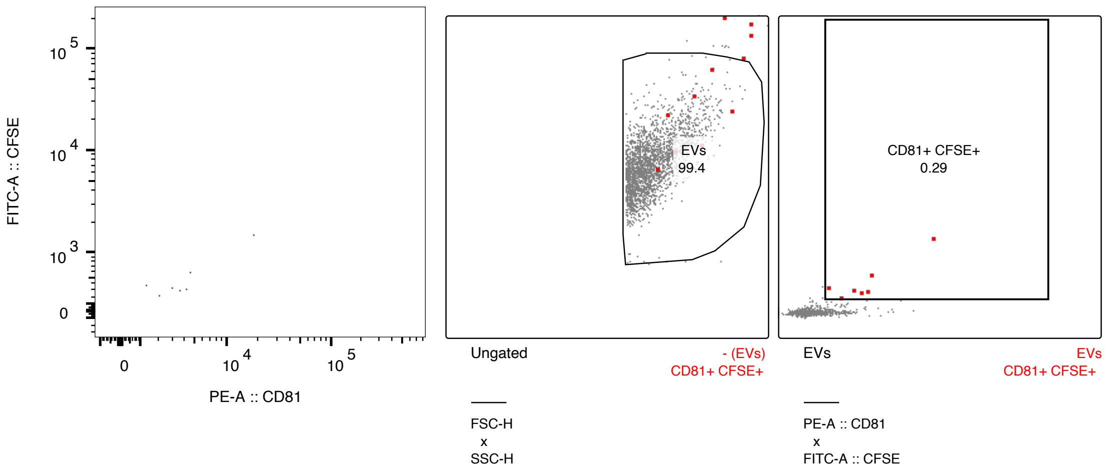

	Sample Name	Freq. of EVs
■	Specimen_001_57-2D (IGG staining)_018.fcs	0.29

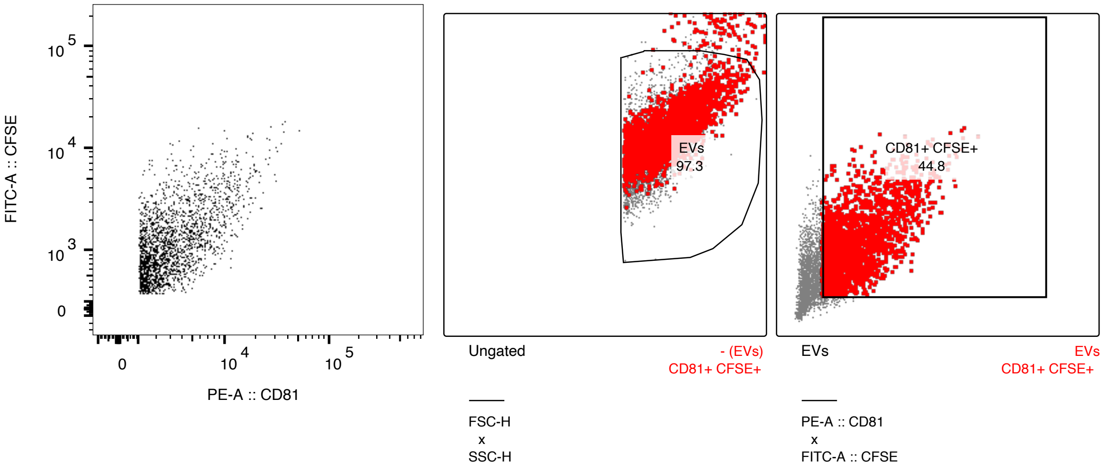

	Sample Name	Freq. of EVs
■	Specimen_001_21-3D (EV staining)_003.fcs	44.8

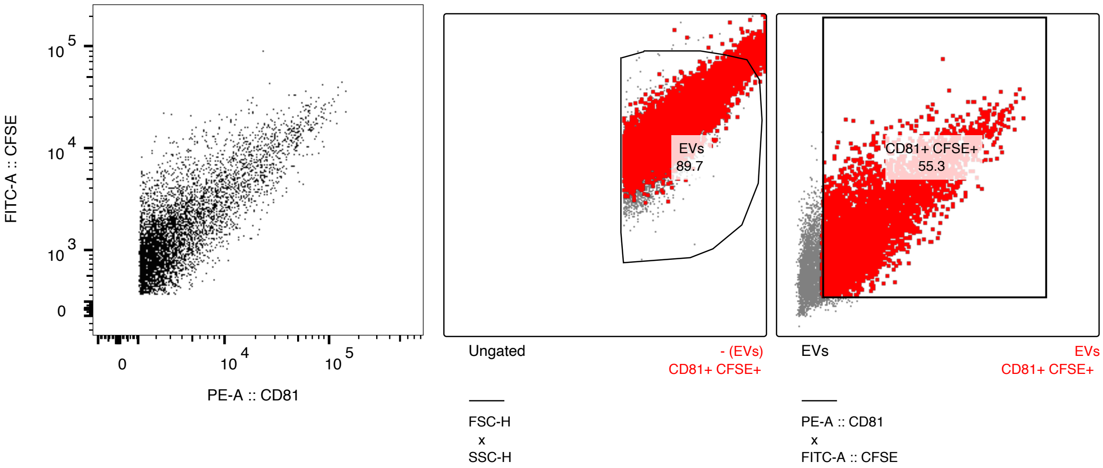

	Sample Name	Freq. of EVs
■	Specimen_001_337-3D (EV staining)_011.fcs	55.3

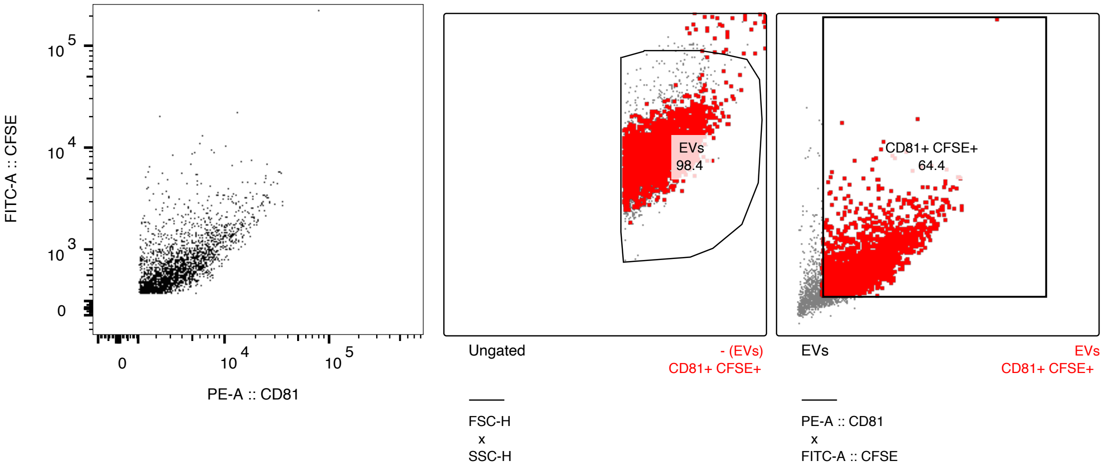

	Sample Name	Freq. of EVs
■	Specimen_001_57-3D (EV staining)_019.fcs	64.4

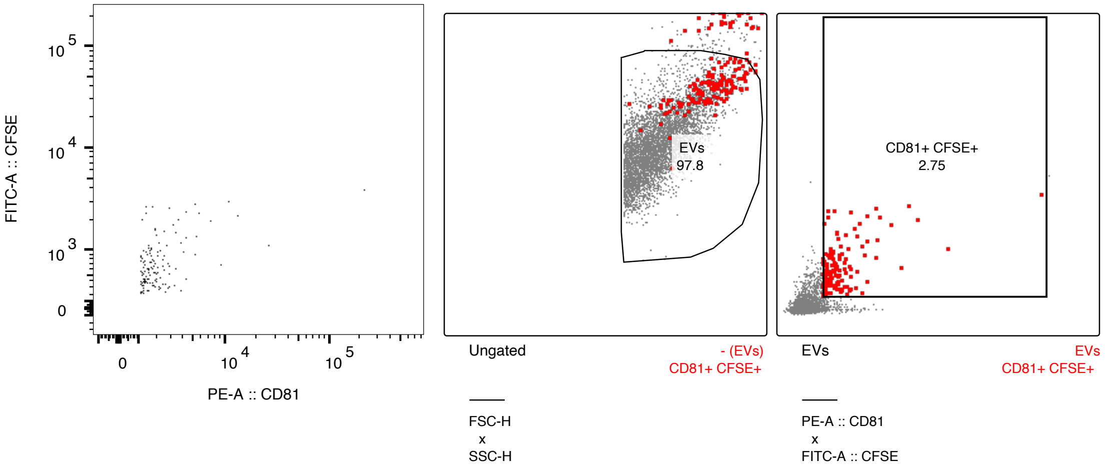

	Sample Name	Freq. of EVs
■	Specimen_001_21-3D (IgG staining)_004.fcs	2.75

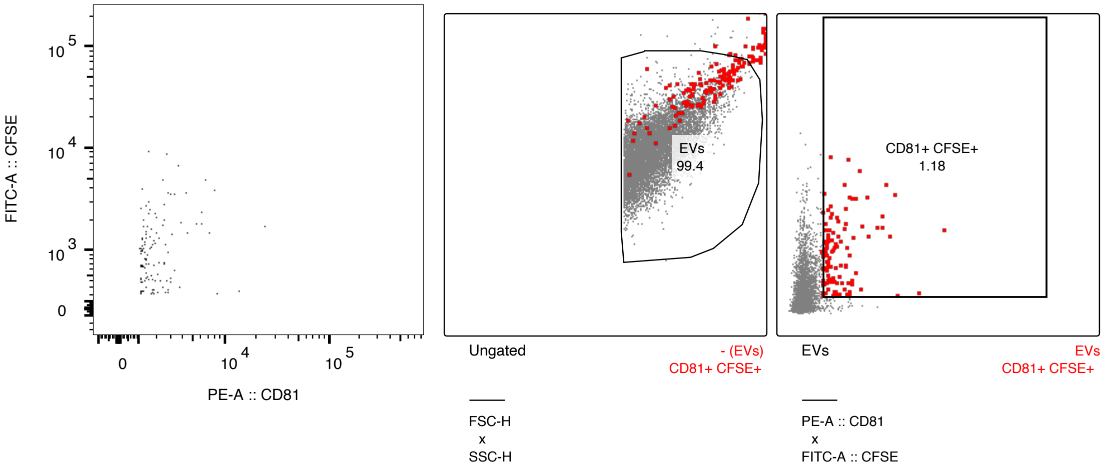

	Sample Name	Freq. of EVs
■	Specimen_001_337-3D (IGG staining)_012.fcs	1.18

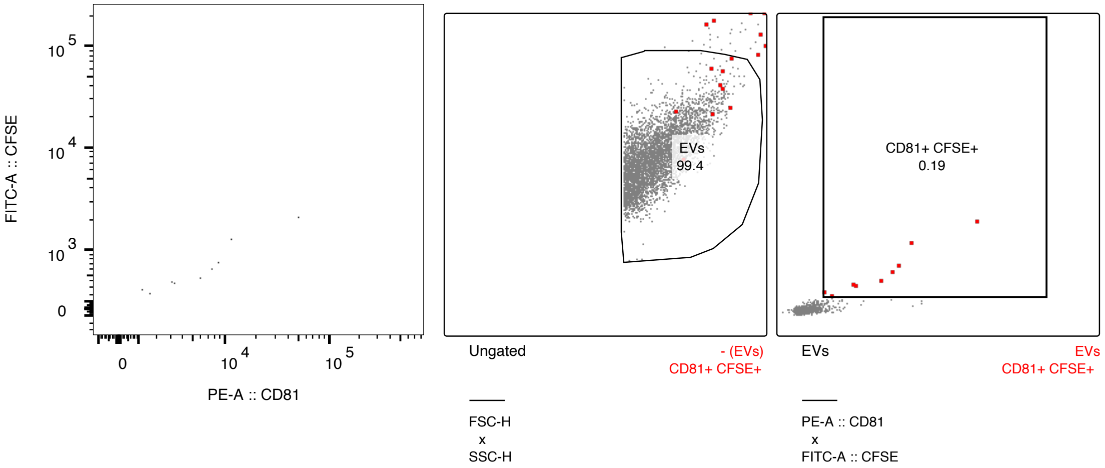

	Sample Name	Freq. of EVs
■	Specimen_001_57-3D (IGG staining)_020.fcs	0.19

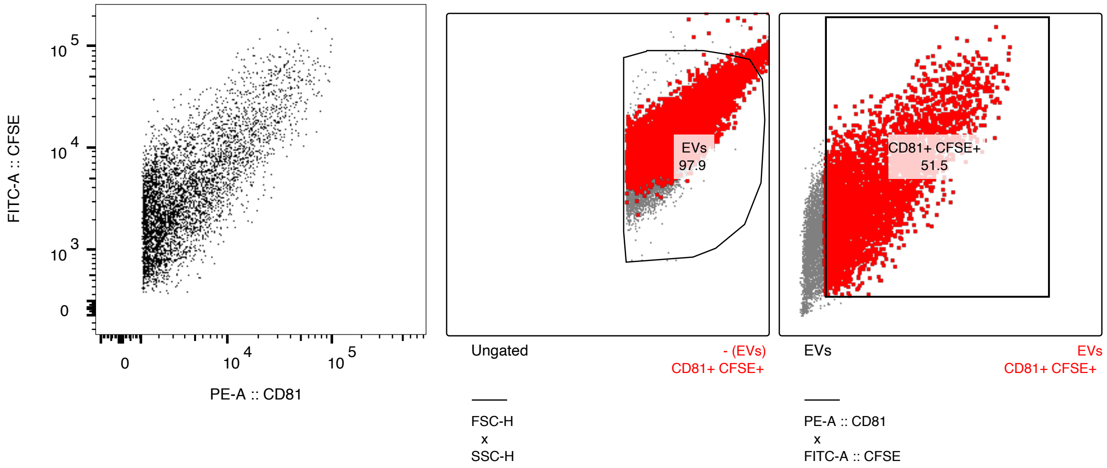

	Sample Name	Freq. of EVs
■	Specimen_001_27-2D (EV staining)_005.fcs	51.5

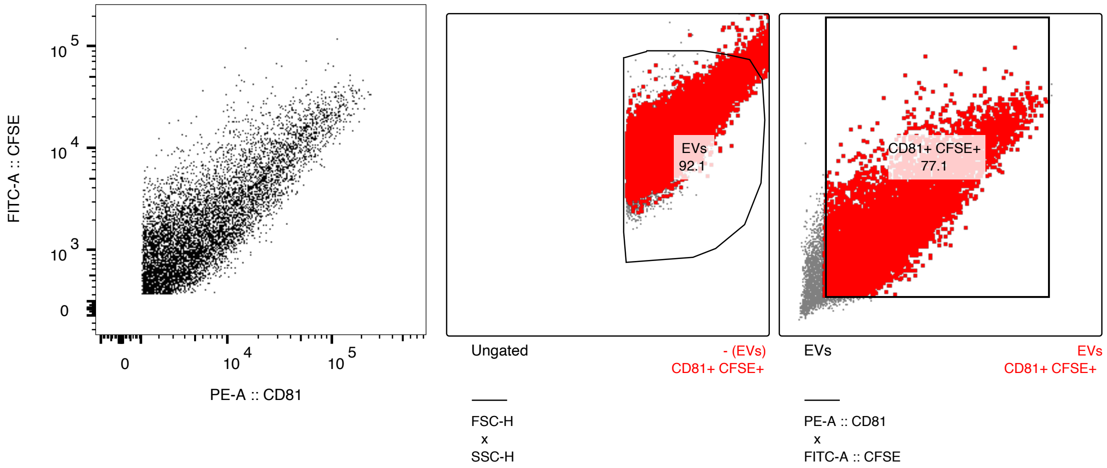

	Sample Name	Freq. of EVs
■	Specimen_001_344-2D (EV staining)_013.fcs	77.1

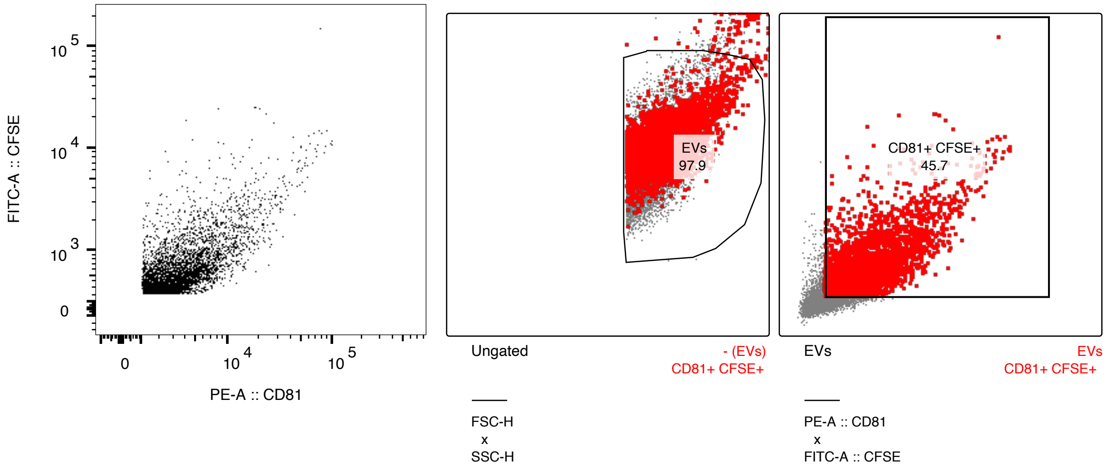

	Sample Name	Freq. of EVs
■	Specimen_001_NP25-2D (EV staining)_021.fcs	45.7

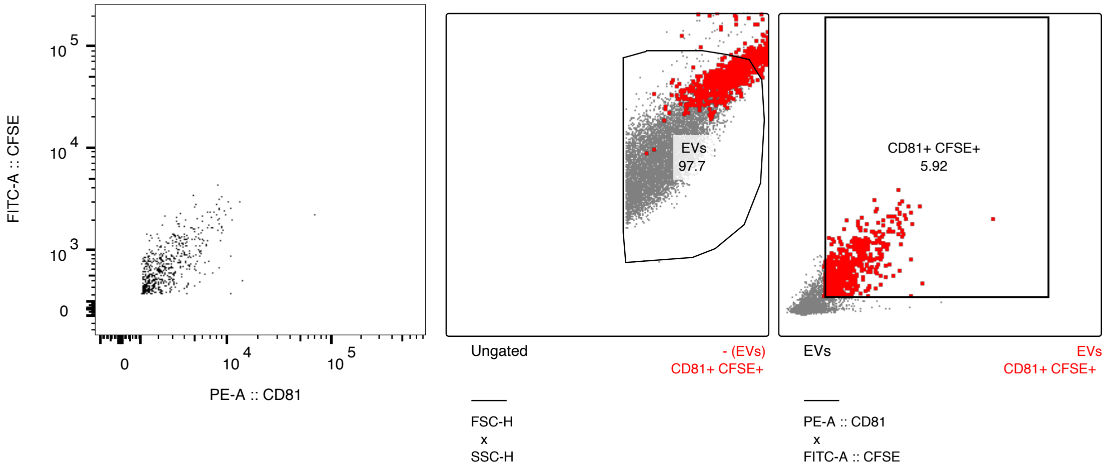

	Sample Name	Freq. of EVs
■	Specimen_001_27-2D (IgG staining)_006.fcs	5.92

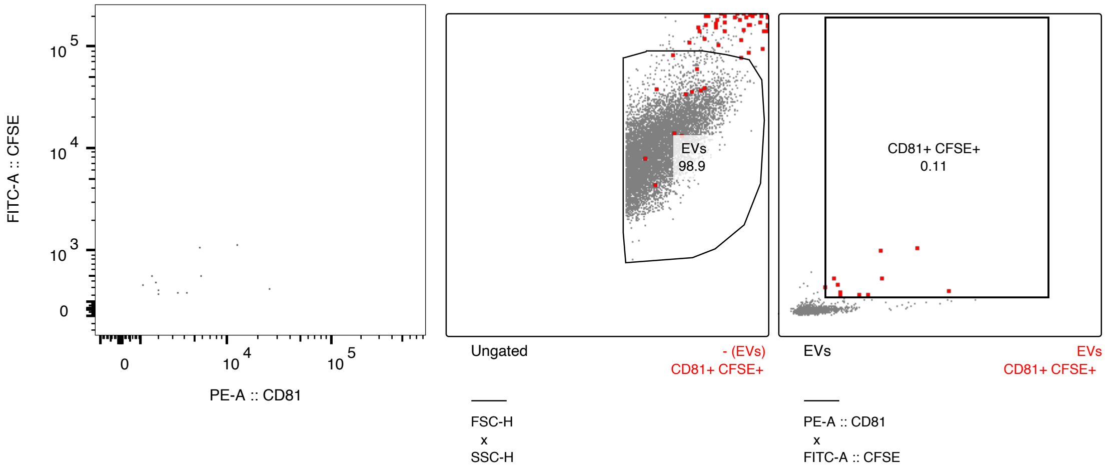

	Sample Name	Freq. of EVs
■	Specimen_001_344-2D (IGG staining)_014.fcs	0.11

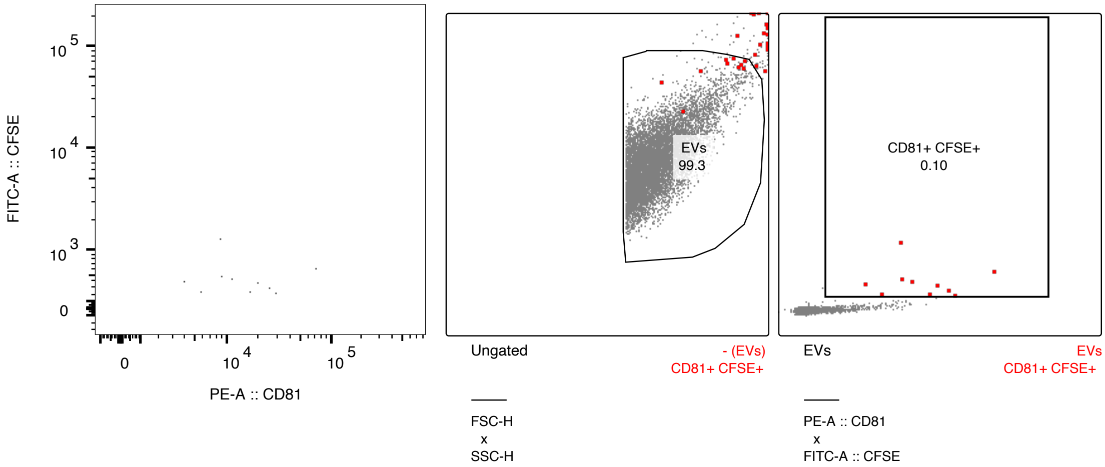

	Sample Name	Freq. of EVs
■	Specimen_001_NP25-2D (IGG staining)_022.fcs	0.10

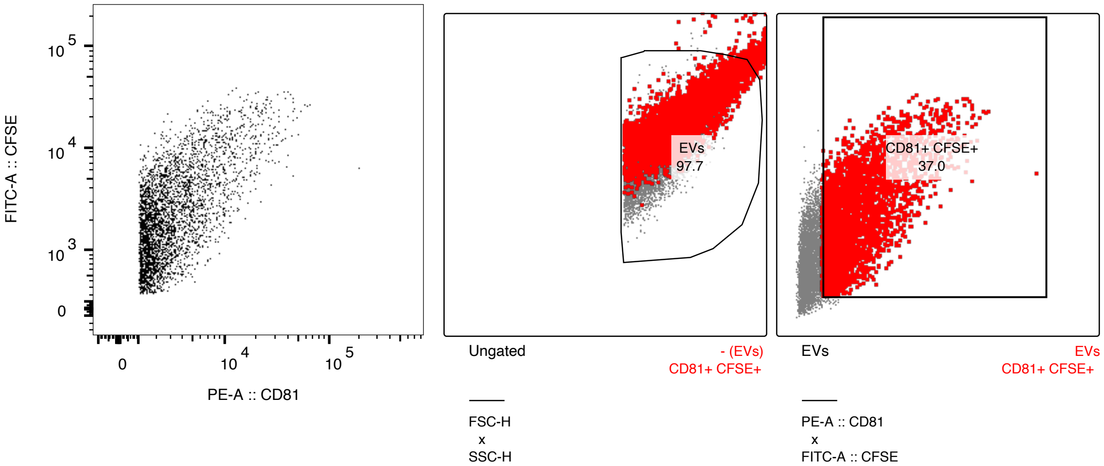

	Sample Name	Freq. of EVs
■	Specimen_001_27-3D (EV staining)_007.fcs	37.0

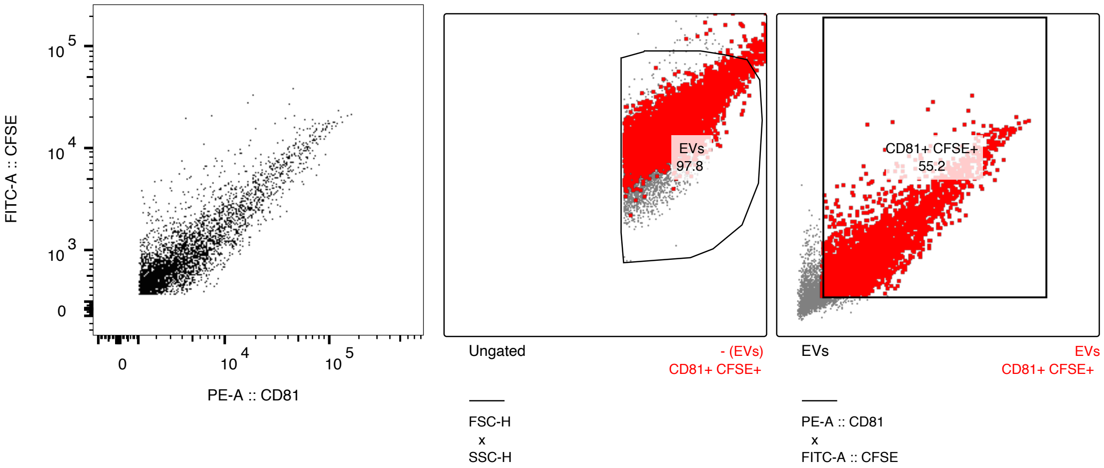

	Sample Name	Freq. of EVs
■	Specimen_001_344-3D (EV staining)_015.fcs	55.2

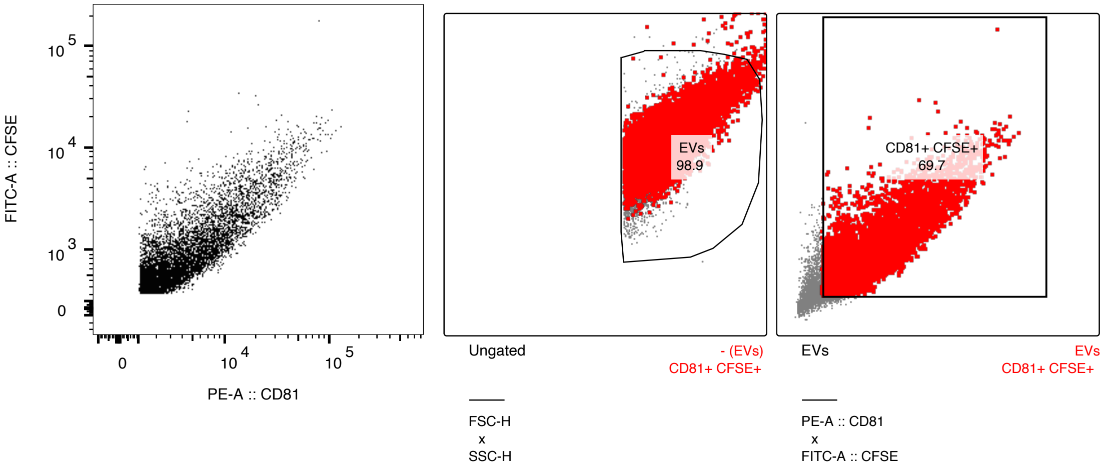

	Sample Name	Freq. of EVs
■	Specimen_001_NP25-3D (EV staining)_023.fcs	69.7

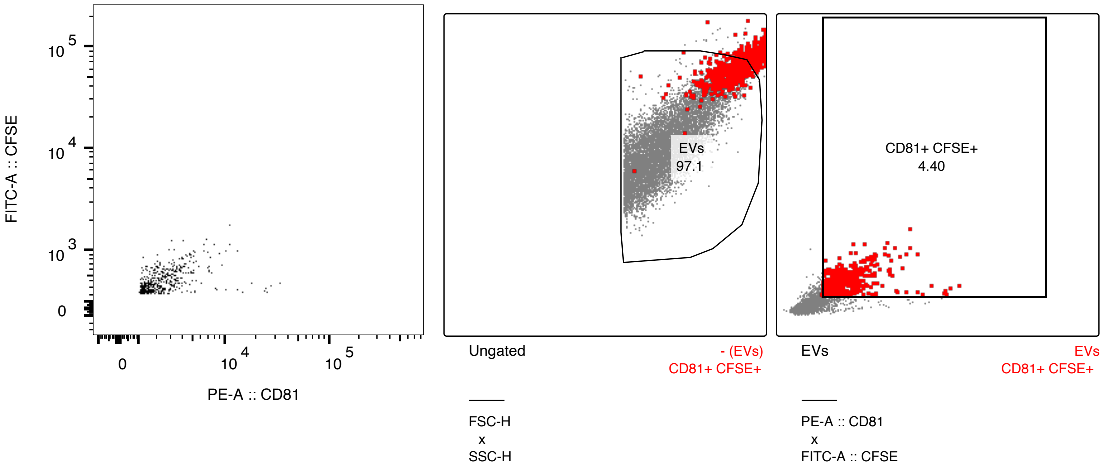

	Sample Name	Freq. of EVs
■	Specimen_001_27-3D (IgG staining)_008.fcs	4.40

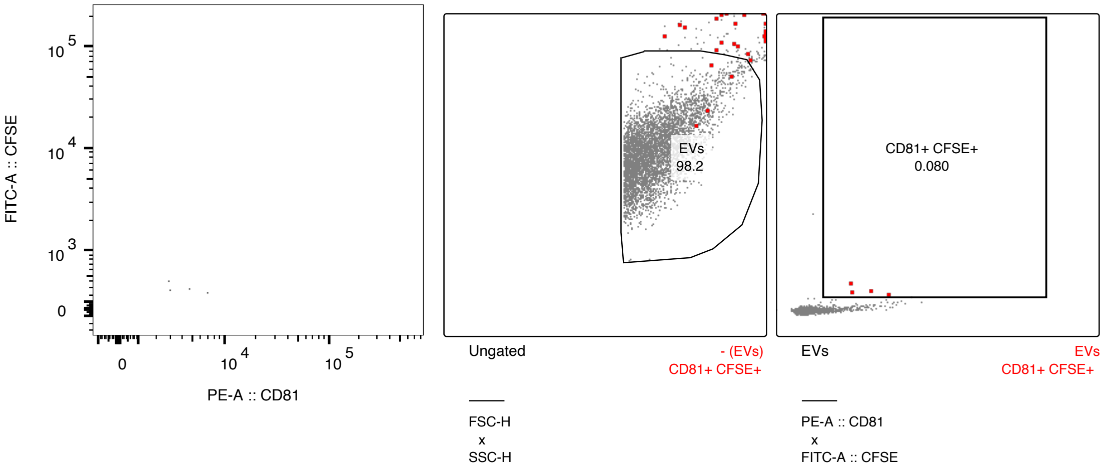

	Sample Name	Freq. of EVs
■	Specimen_001_344-3D (IGG staining)_016.fcs	0.080

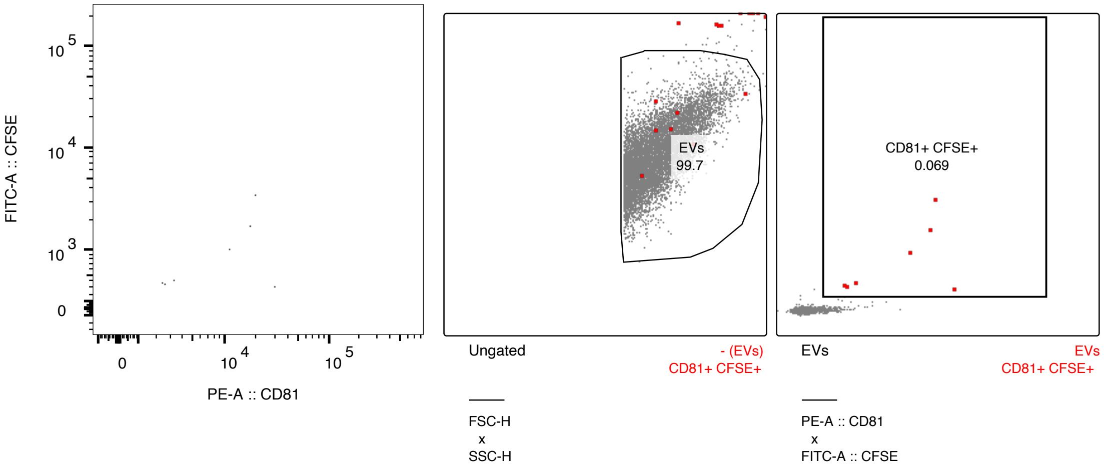

	Sample Name	Freq. of EVs
■	Specimen_001_NP25-3D (IGG staining)_024.fcs	0.069

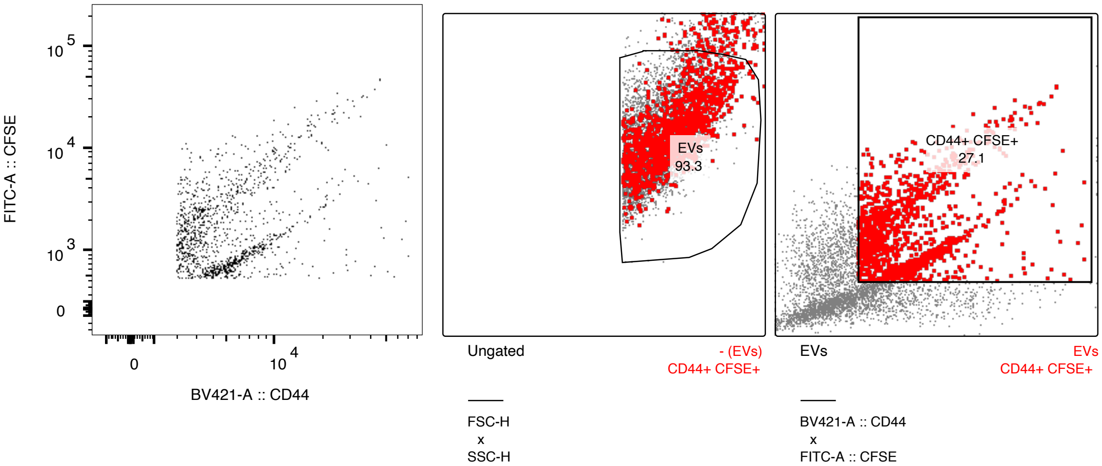

	Sample Name	Freq. of EVs
■	Specimen_001_21-2D (EV staining)_001.fcs	27.1

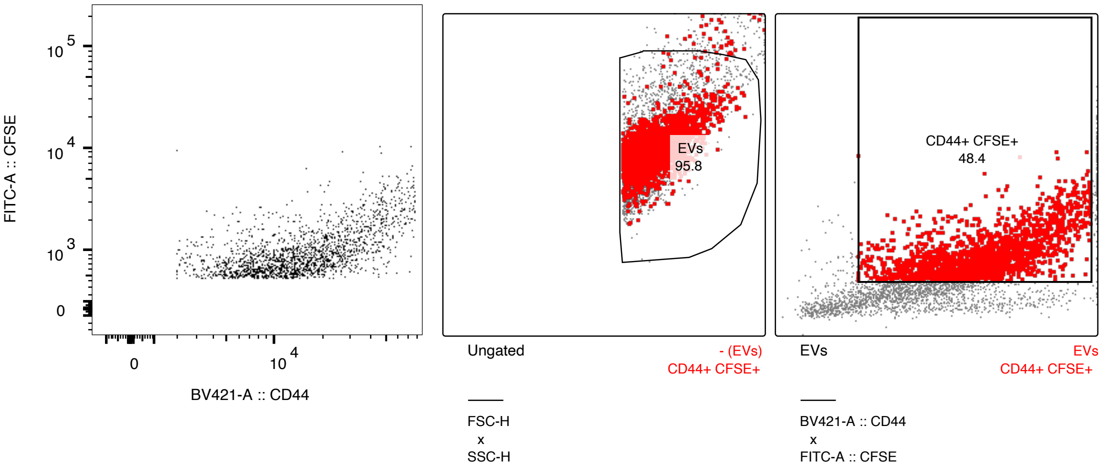

	Sample Name	Freq. of EVs
■	Specimen_001_337-2D (EV staining)_009.fcs	48.4

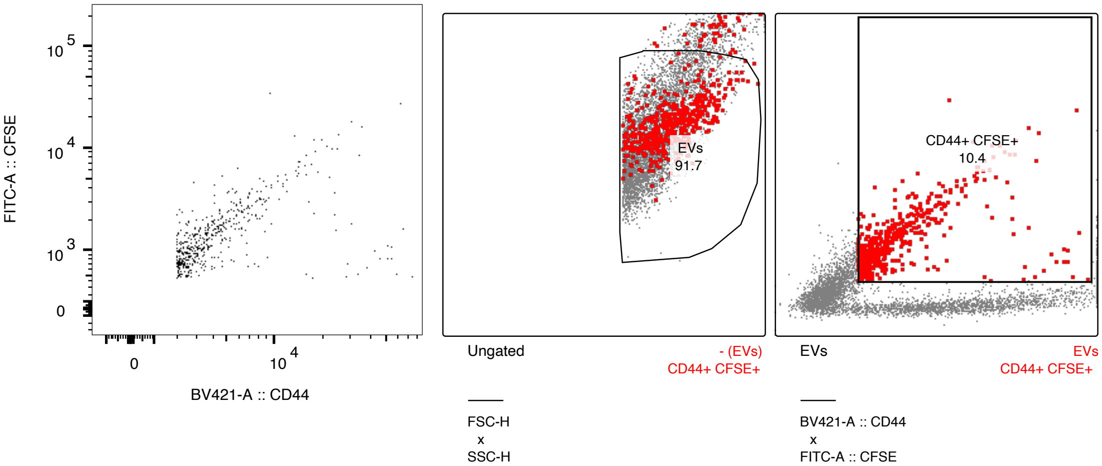

	Sample Name	Freq. of EVs
■	Specimen_001_57-2D (EV staining)_017.fcs	10.4

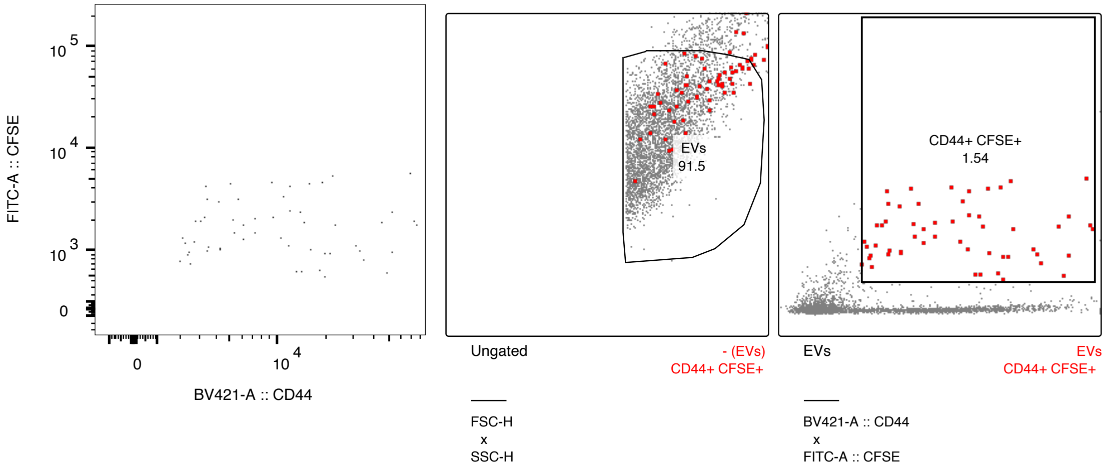

	Sample Name	Freq. of EVs
■	Specimen_001_21-2D (IgG staining)_002.fcs	1.54

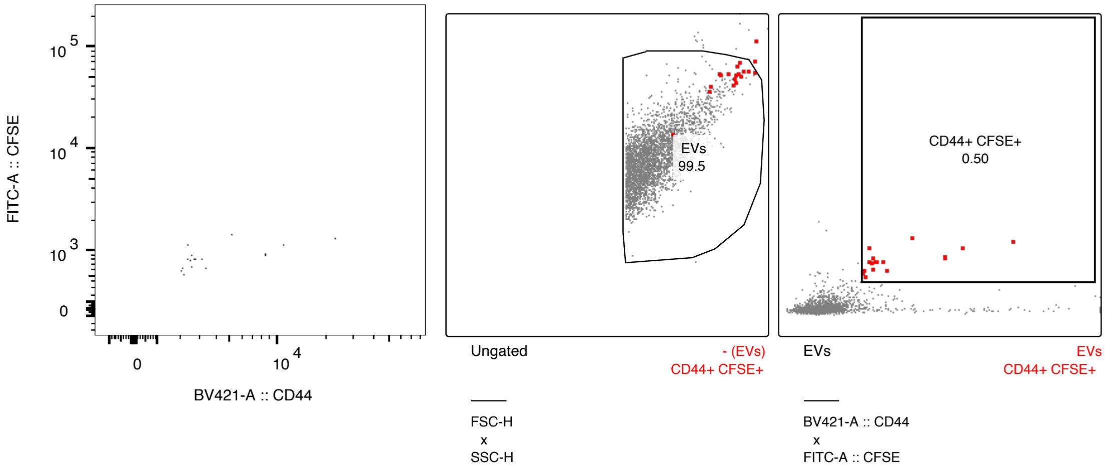

	Sample Name	Freq. of EVs
■	Specimen_001_337-2D (IGG staining)_010.fcs	0.50

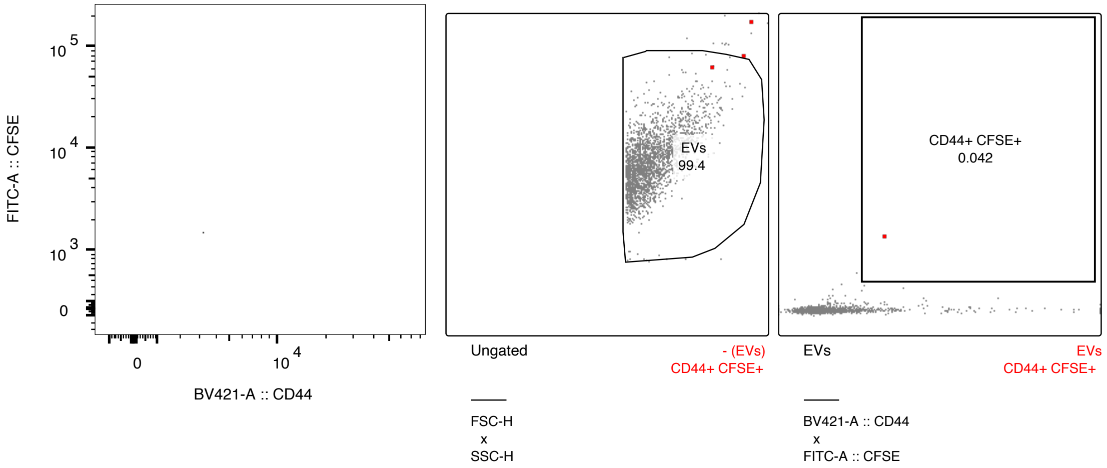

	Sample Name	Freq. of EVs
■	Specimen_001_57-2D (IGG staining)_018.fcs	0.042

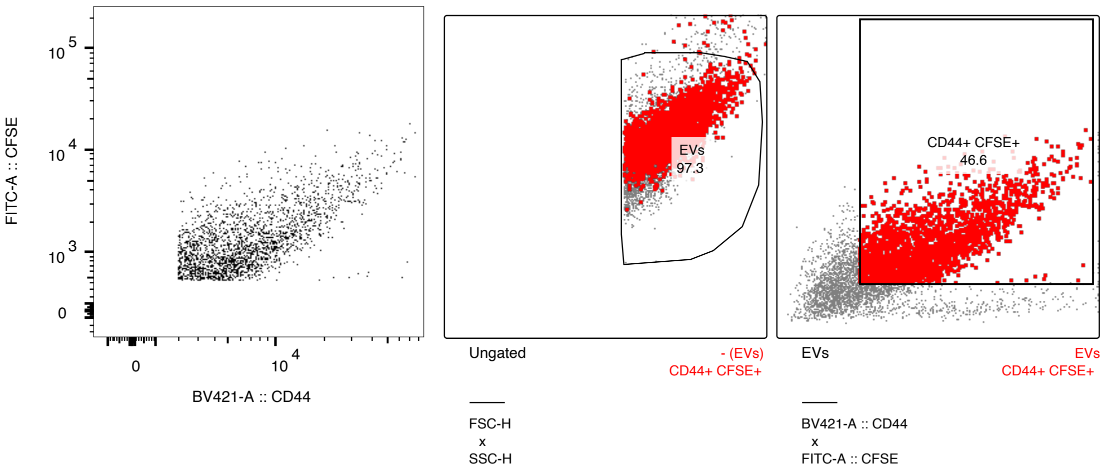

	Sample Name	Freq. of EVs
■	Specimen_001_21-3D (EV staining)_003.fcs	46.6

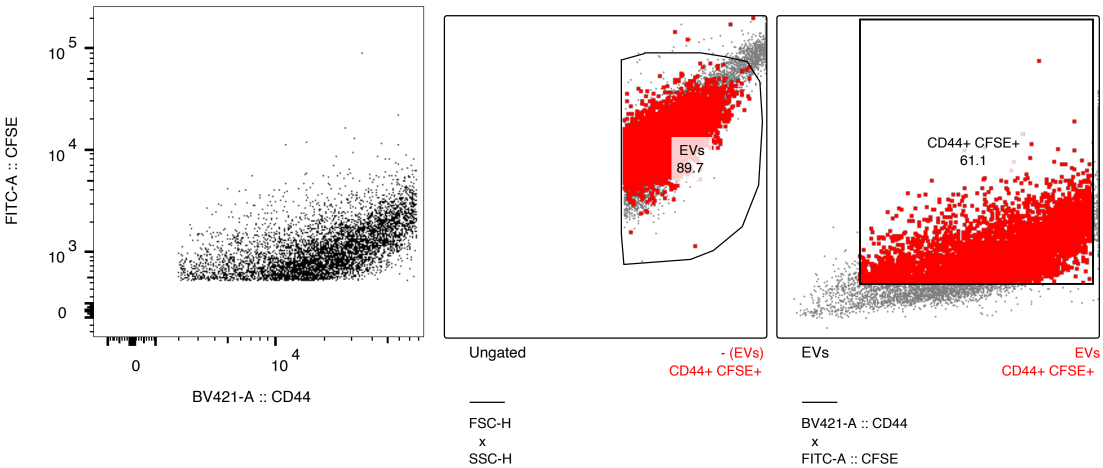

	Sample Name	Freq. of EVs
■	Specimen_001_337-3D (EV staining)_011.fcs	61.1

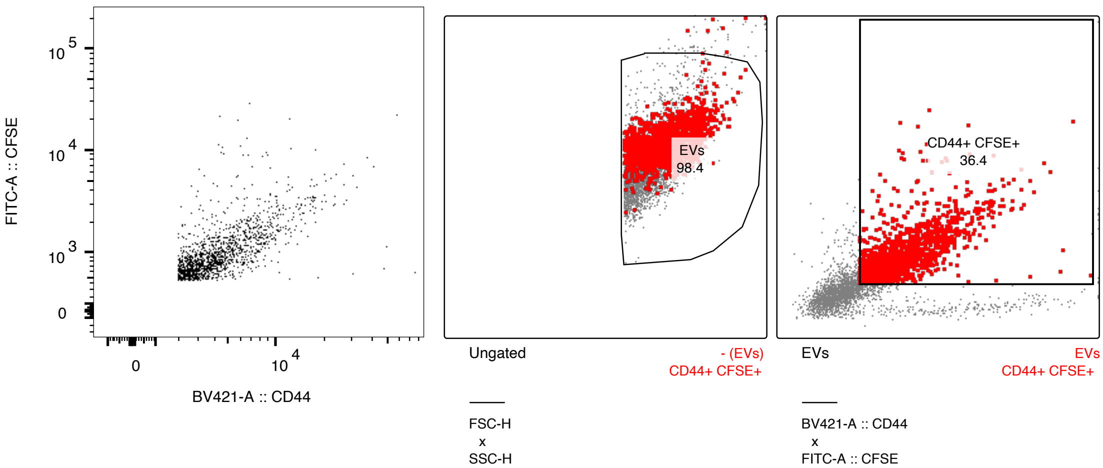

	Sample Name	Freq. of EVs
■	Specimen_001_57-3D (EV staining)_019.fcs	36.4

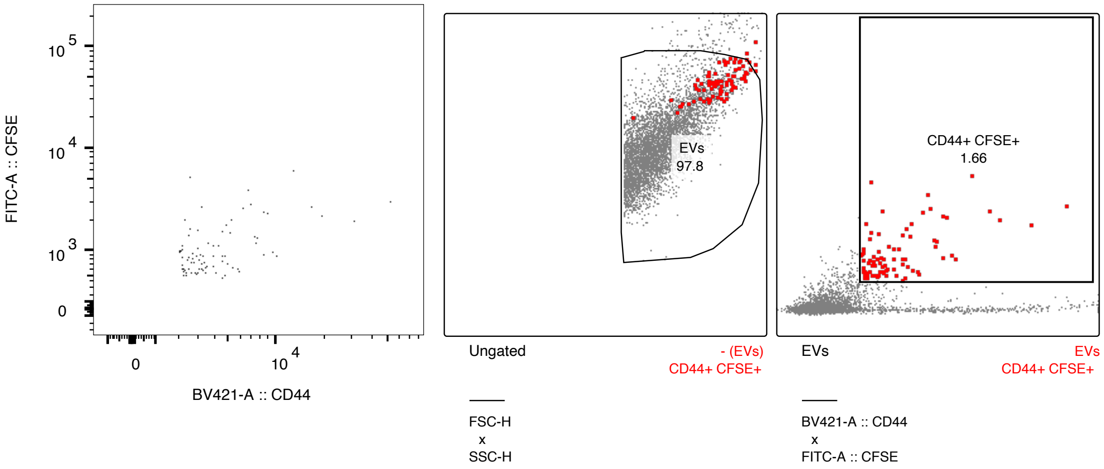

	Sample Name	Freq. of EVs
■	Specimen_001_21-3D (IgG staining)_004.fcs	1.66

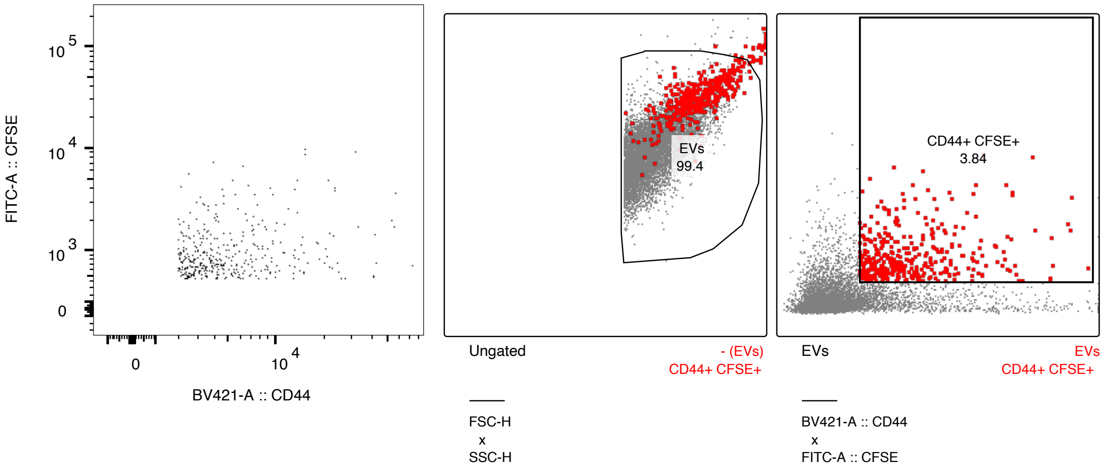

	Sample Name	Freq. of EVs
■	Specimen_001_337-3D (IGG staining)_012.fcs	3.84

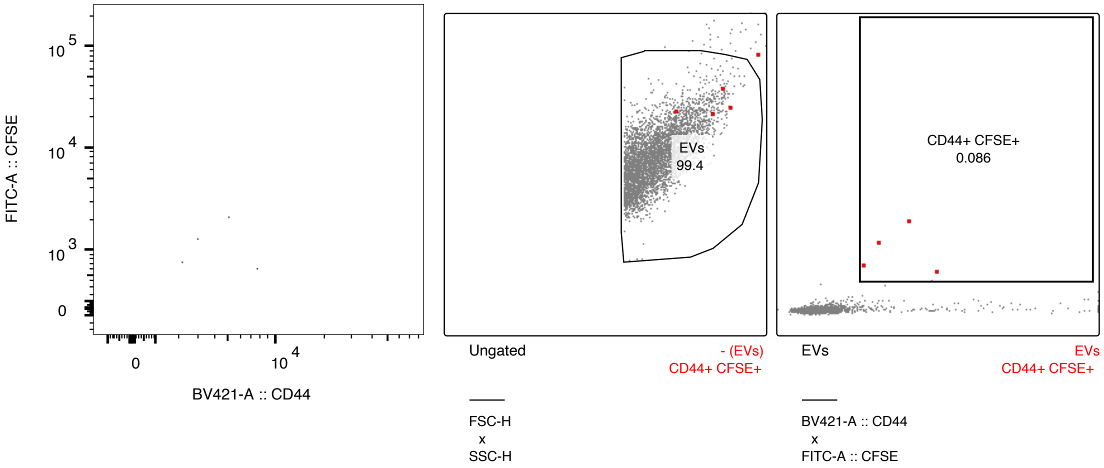

	Sample Name	Freq. of EVs
■	Specimen_001_57-3D (IGG staining)_020.fcs	0.086

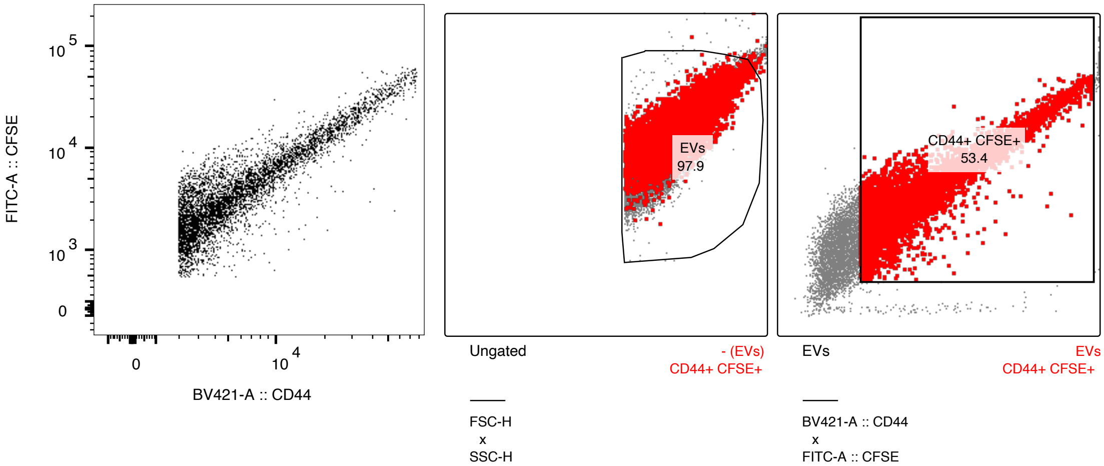

	Sample Name	Freq. of EVs
■	Specimen_001_27-2D (EV staining)_005.fcs	53.4

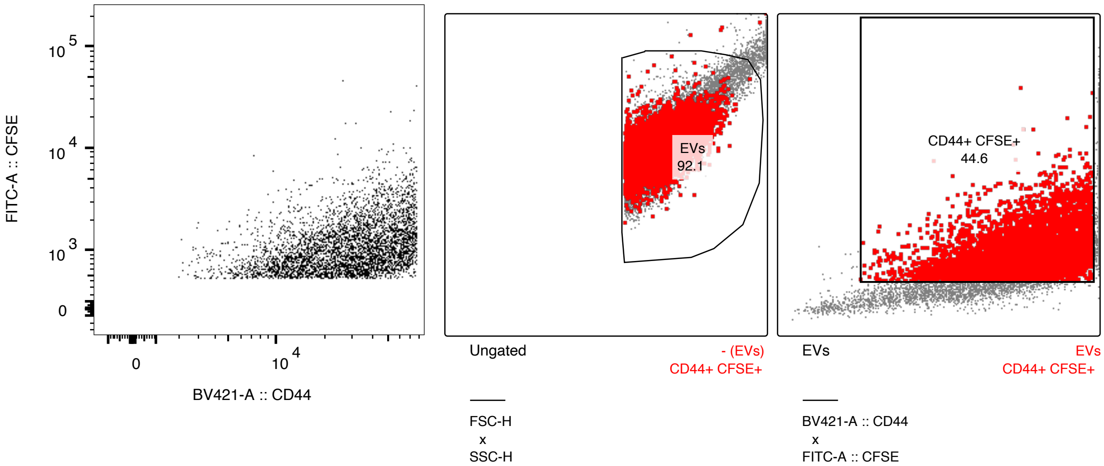

	Sample Name	Freq. of EVs
■	Specimen_001_344-2D (EV staining)_013.fcs	44.6

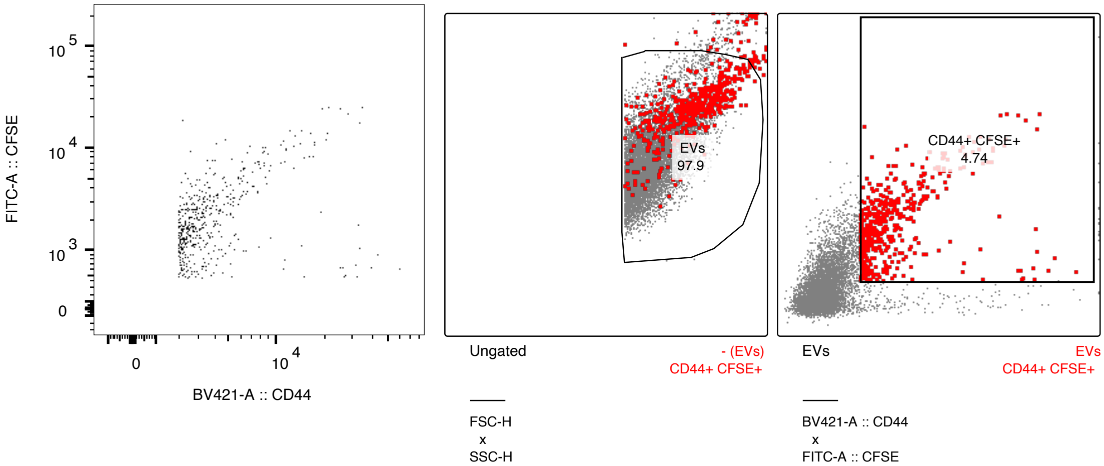

	Sample Name	Freq. of EVs
■	Specimen_001_NP25-2D (EV staining)_021.fcs	4.74

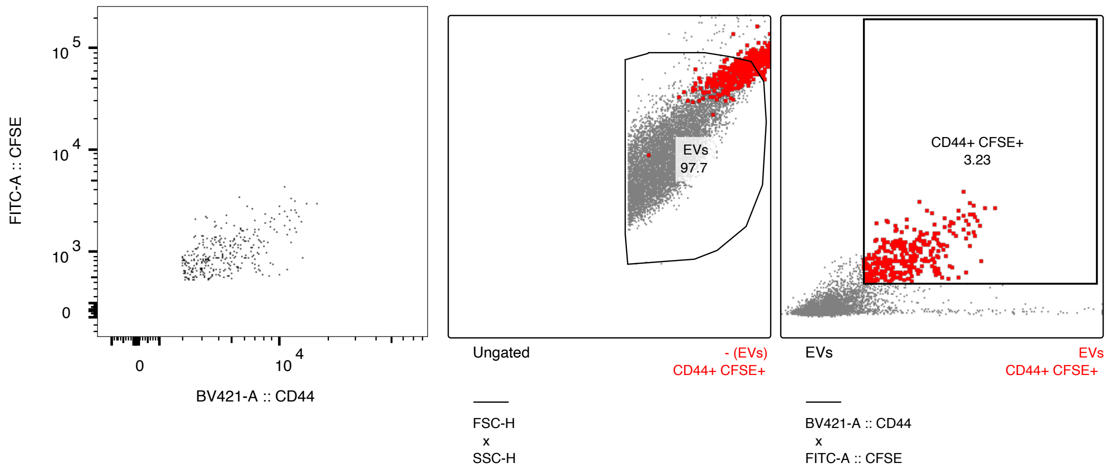

	Sample Name	Freq. of EVs
■	Specimen_001_27-2D (IgG staining)_006.fcs	3.23

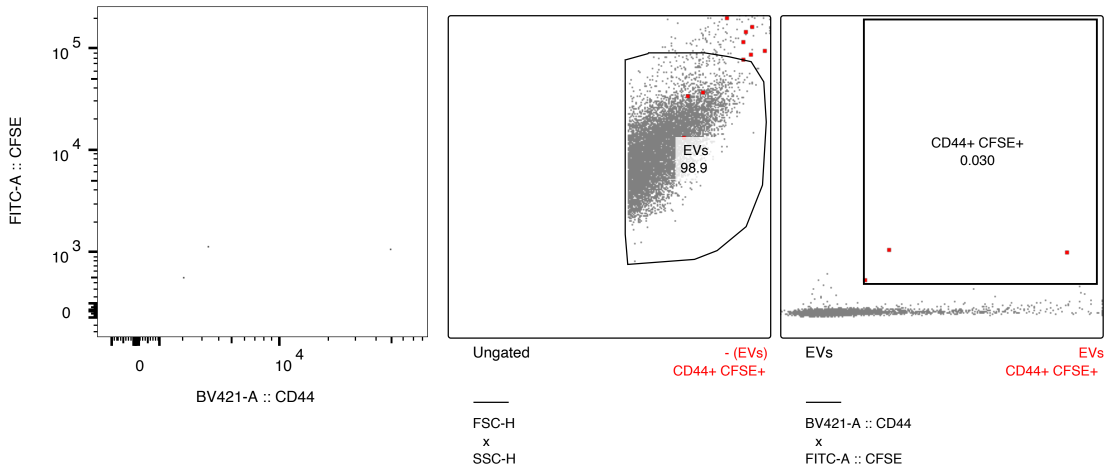

	Sample Name	Freq. of EVs
■	Specimen_001_344-2D (IGG staining)_014.fcs	0.030

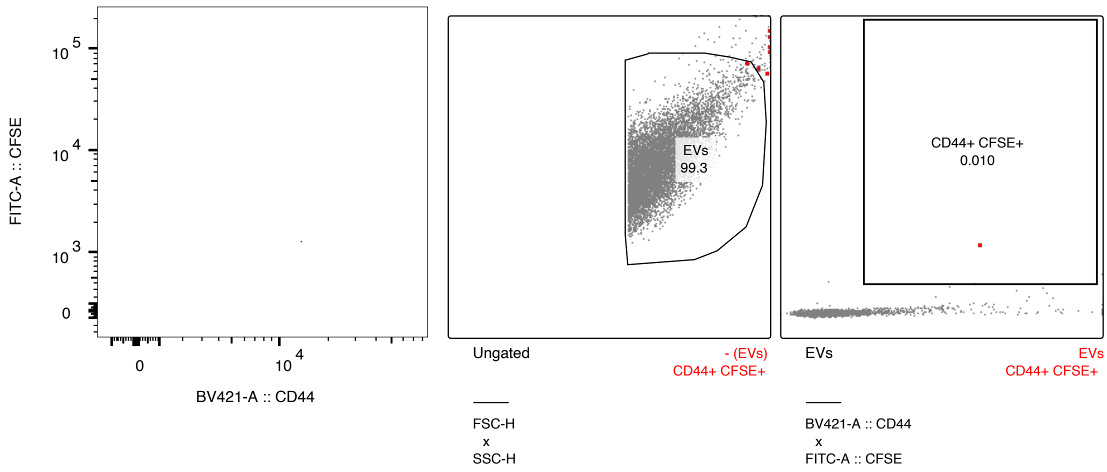

	Sample Name	Freq. of EVs
■	Specimen_001_NP25-2D (IGG staining)_022.fcs	0.010

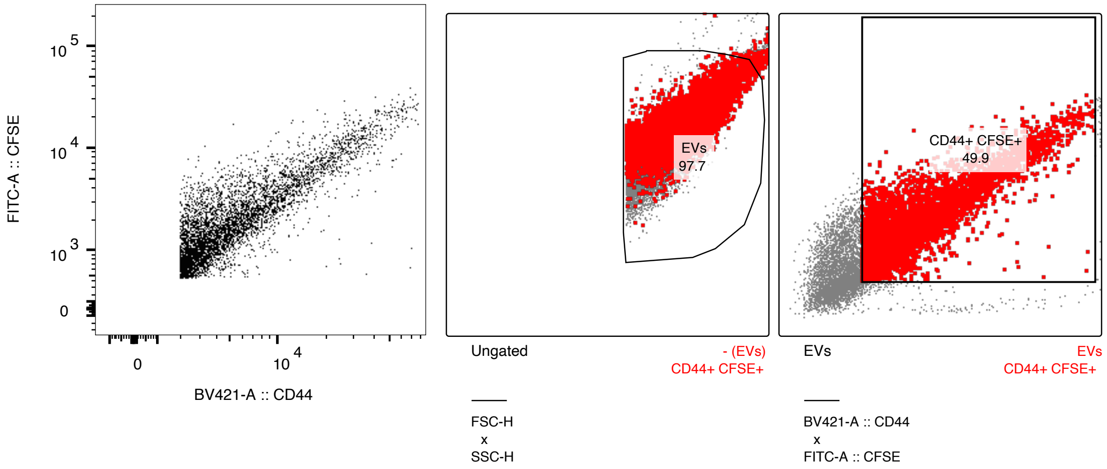

Sample Name	Freq. of EVs
Specimen_001_27-3D (EV staining)_007.fcs	49.9

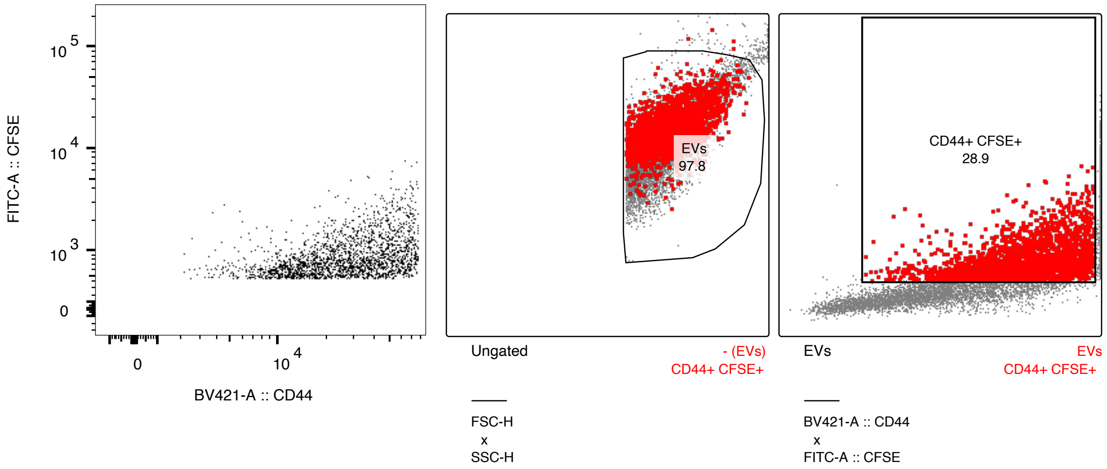

Sample Name	Freq. of EVs
Specimen_001_344-3D (EV staining)_015.fcs	28.9

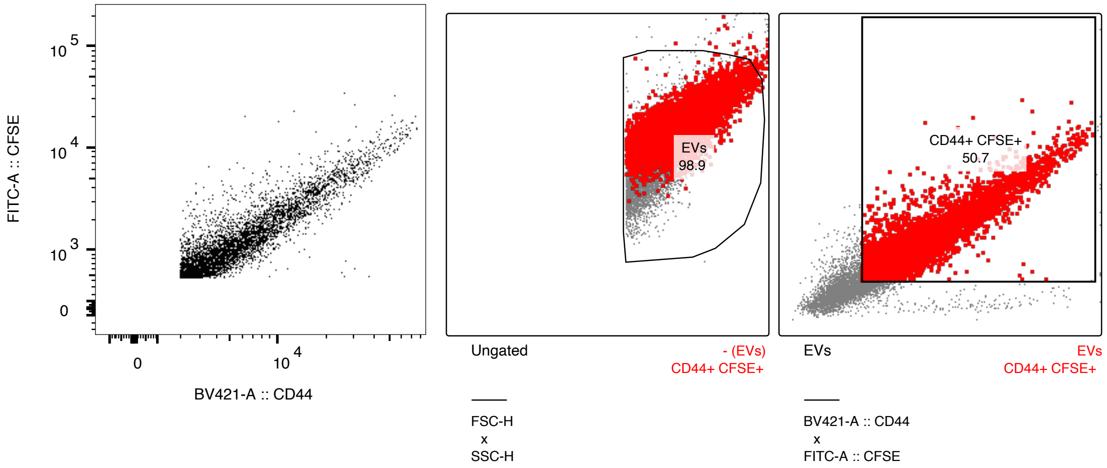

Sample Name	Freq. of EVs
Specimen_001_NP25-3D (EV staining)_023.fcs	50.7

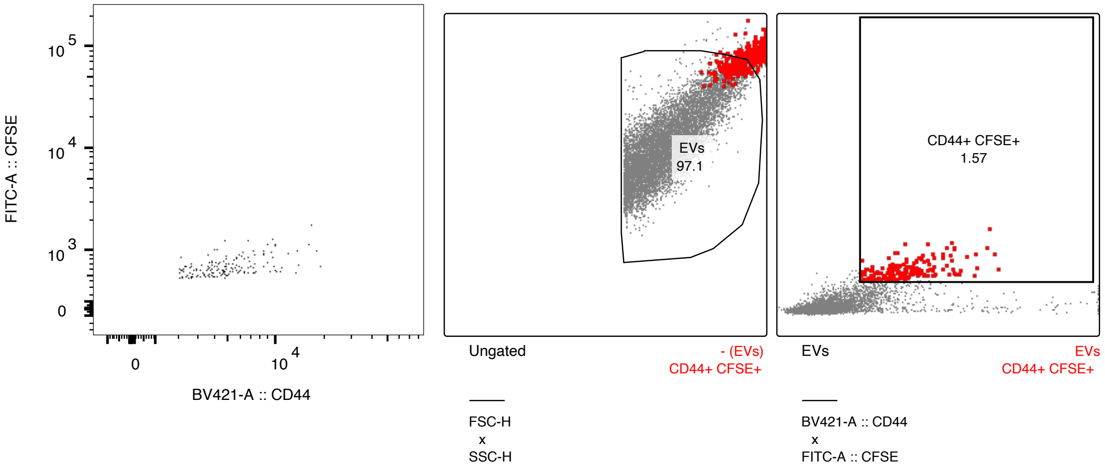

	Sample Name	Freq. of EVs
■	Specimen_001_27-3D (IgG staining)_008.fcs	1.57

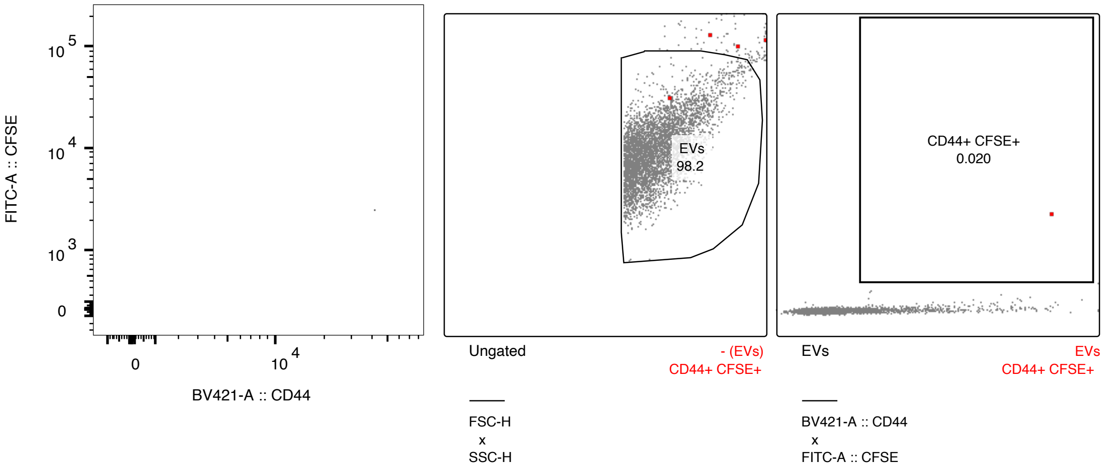

	Sample Name	Freq. of EVs
■	Specimen_001_344-3D (IGG staining)_016.fcs	0.020

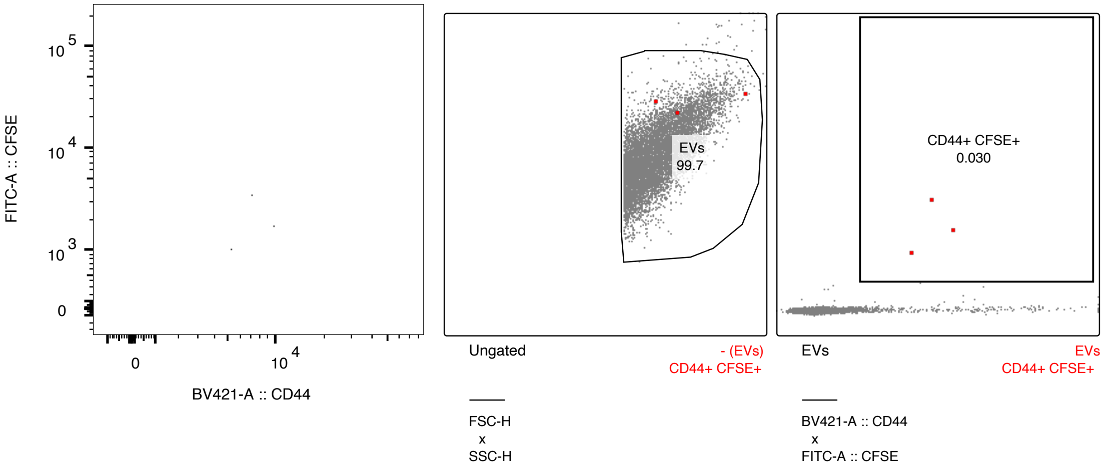

	Sample Name	Freq. of EVs
■	Specimen_001_NP25-3D (IGG staining)_024.fcs	0.030

PP: Bead-based intracellular marker staining

Cell line name in the manuscript	Cell line name in the gating dot plot
BTIC10	RAV21
BTIC13	RAV27
BTIC131	RAV344
BTIC18	RAV57
BTIC129	RAV337
BTIC155	NP025

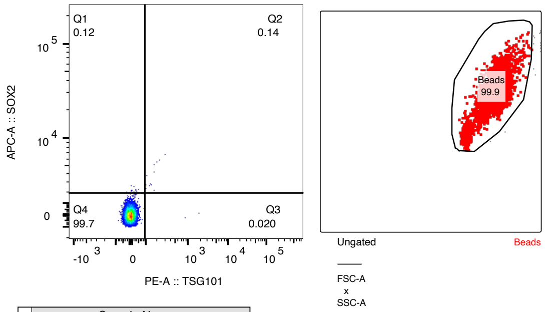

	Sample Name
■	Specimen_001_EF_IgG_025.fcs

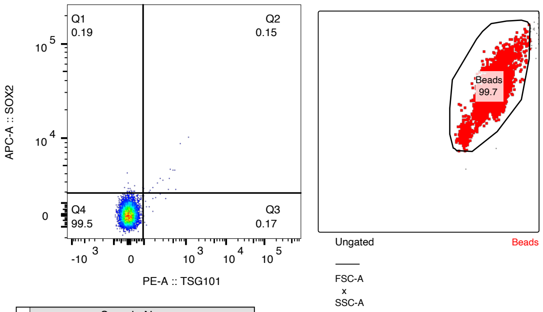

	Sample Name
■	Specimen_001_EF_intraEV_stain_026.fcs

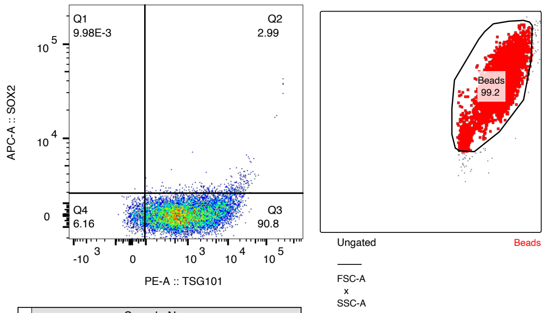

	Sample Name
■	Specimen_001_2D_RAV27_intraEV_stain_006.fcs

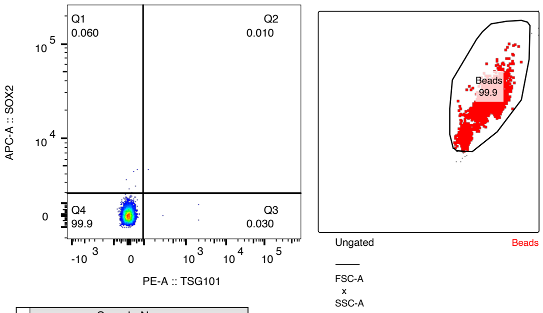

	Sample Name
■	Specimen_001_2D_RAV57_IgG_017.fcs

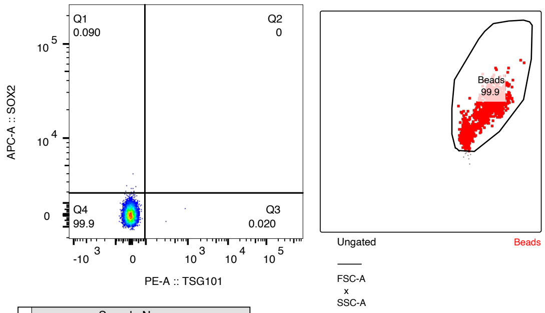

	Sample Name
■	Specimen_001_3D_NP025_IgG_023.fcs

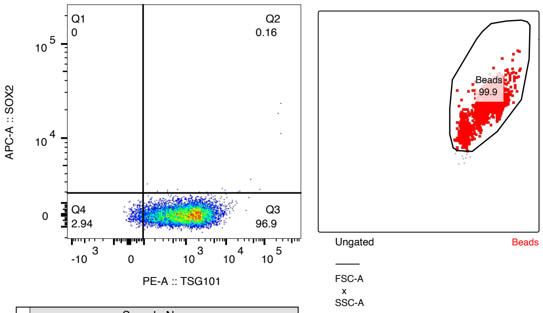

	Sample Name
■	Specimen_001_3D_NP025_intraEV_stain_024.fcs

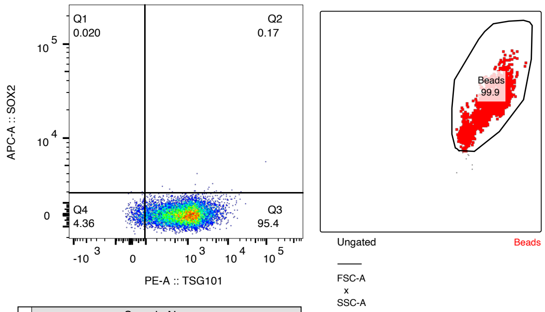

	Sample Name
■	Specimen_001_3D_RAV57_intraEV_stain_020.fcs

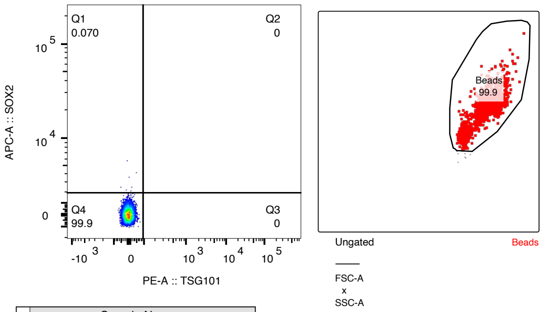

	Sample Name
■	Specimen_001_3D_RAV337_IgG_011.fcs

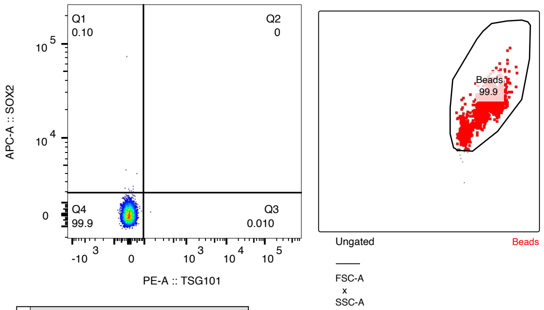

Sample Name
Specimen_001_2D_NP025_IgG_021.fcs

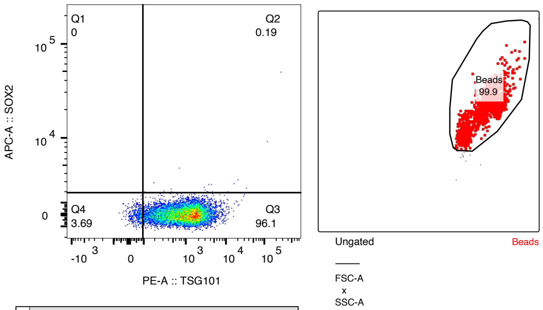

Sample Name
Specimen_001_2D_NP025_intraEV_stain_022.fcs

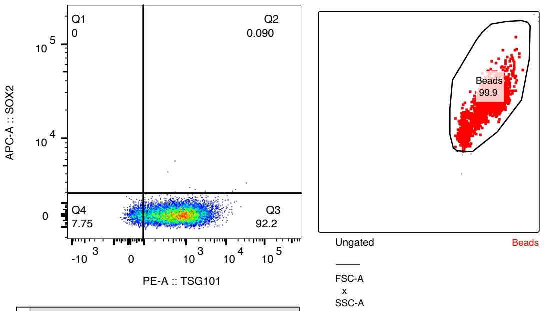

Sample Name
Specimen_001_2D_RAV57_intraEV_stain_018.fcs

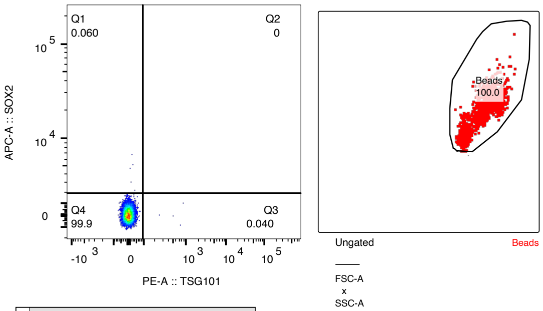

Sample Name
Specimen_001_2D_RAV337_IgG_009.fcs

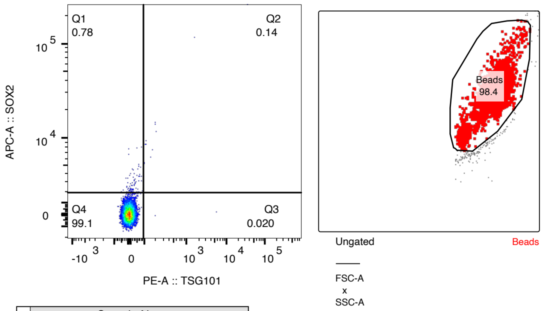

Sample Name
Specimen_001_3D_RAV21_IgG_003.fcs

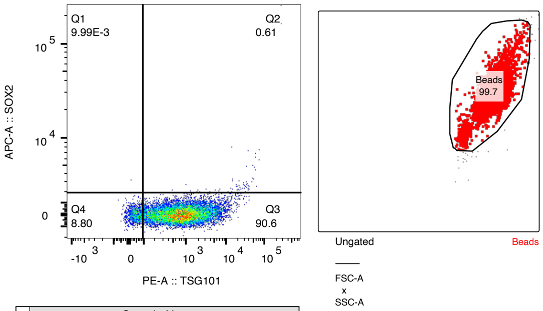

Sample Name
Specimen_001_3D_RAV21_intraEV_stain_004.fcs

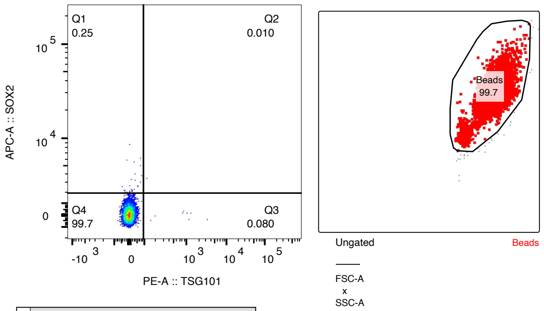

Sample Name
Specimen_001_3D_RAV344_IgG_015.fcs

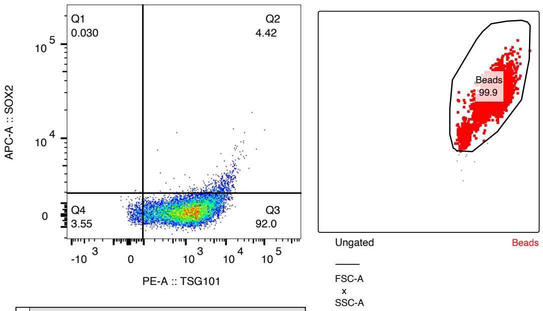

Sample Name
Specimen_001_3D_RAV344_intraEV_stain_016.fcs

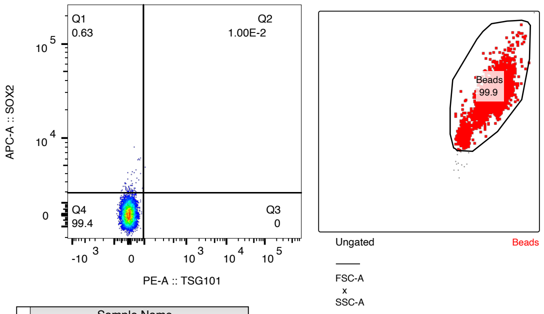

	Sample Name
■	Specimen_001_2D_RAV21_IgG_001.fcs

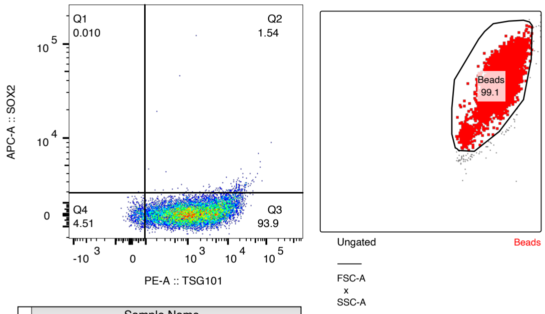

	Sample Name
■	Specimen_001_2D_RAV21_intraEV_stain_002.fcs

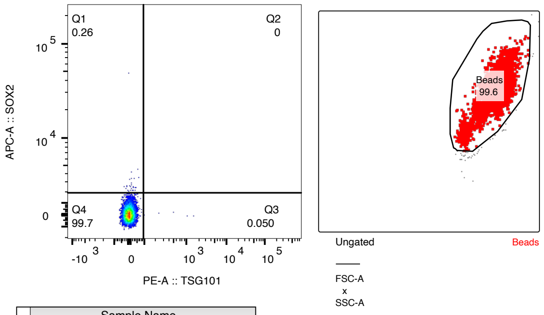

	Sample Name
■	Specimen_001_2D_RAV344_IgG_013.fcs

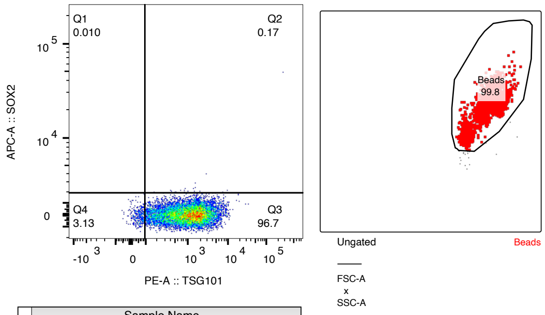

	Sample Name
■	Specimen_001_2D_RA337_intraEV_stain_010.fcs

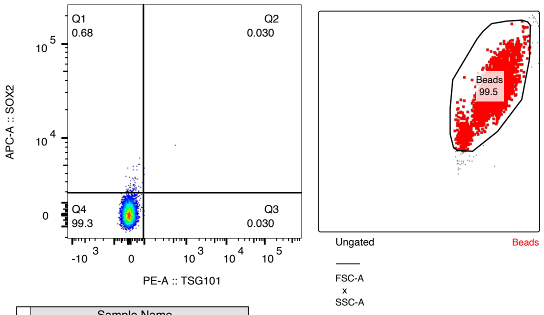

	Sample Name
■	Specimen_001_3D_RAV27_IgG_007.fcs

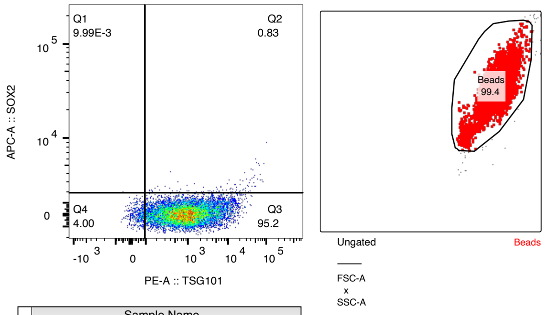

	Sample Name
■	Specimen_001_3D_RAV27_intraEV_stain_008.fcs

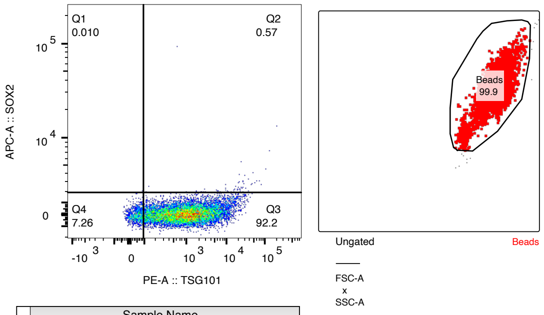

	Sample Name
■	Specimen_001_3D_RA337_intraEV_stain_012.fcs

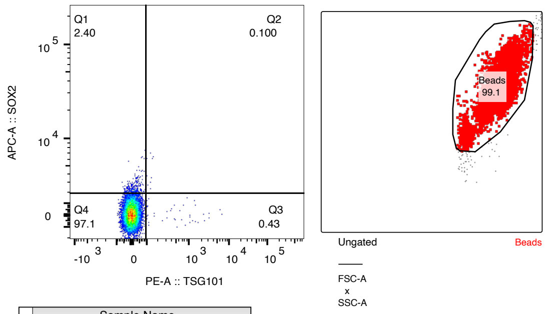

	Sample Name
■	Specimen_001_2D_RAV27_IgG_005.fcs

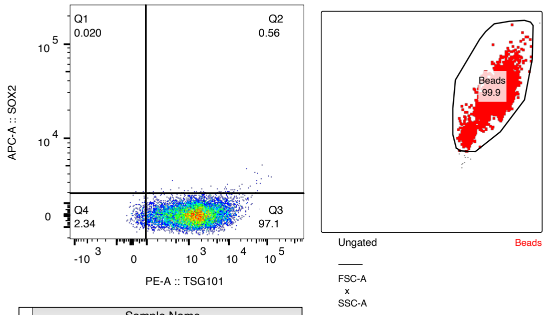

	Sample Name
■	Specimen_001_2D_RA344_intraEV_stain_014.fcs

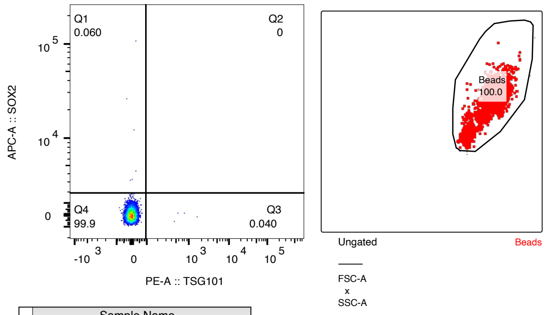

	Sample Name
■	Specimen_001_3D_RAV57_IgG_019.fcs

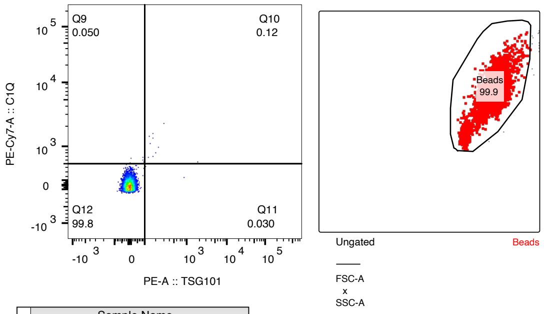

Sample Name
Specimen_001_EF_IgG_025.fcs

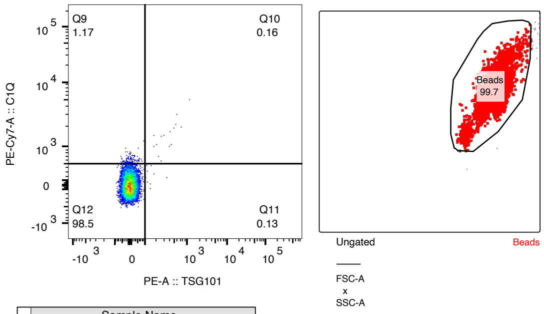

Sample Name
Specimen_001_EF_intraEV_stain_026.fcs

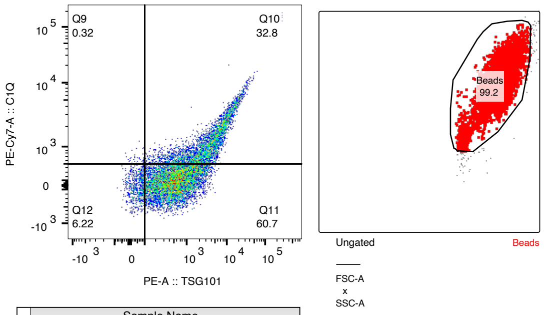

Sample Name
Specimen_001_2D_RAV27_intraEV_stain_006.fcs

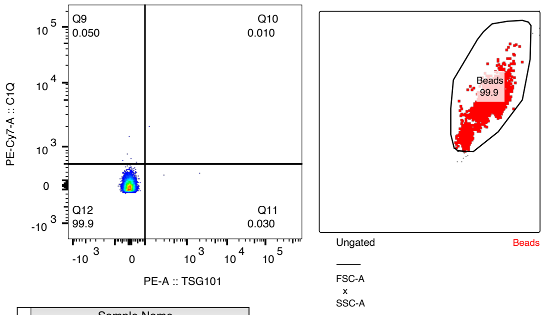

Sample Name
Specimen_001_2D_RAV57_IgG_017.fcs

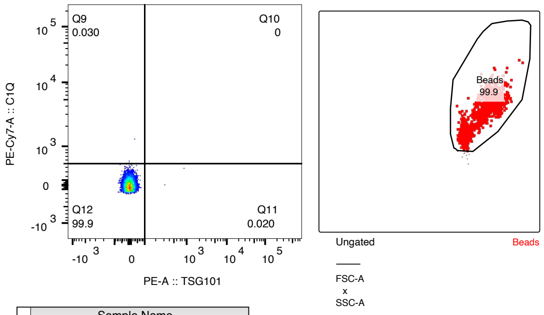

Sample Name
Specimen_001_3D_NP025_IgG_023.fcs

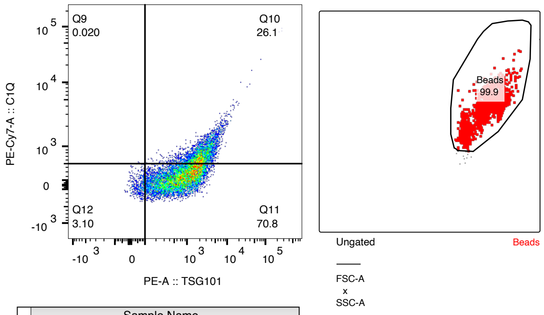

Sample Name
Specimen_001_3D_NP025_intraEV_stain_024.fcs

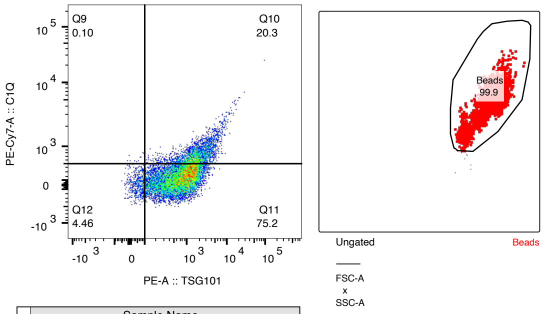

Sample Name
Specimen_001_3D_RAV57_intraEV_stain_020.fcs

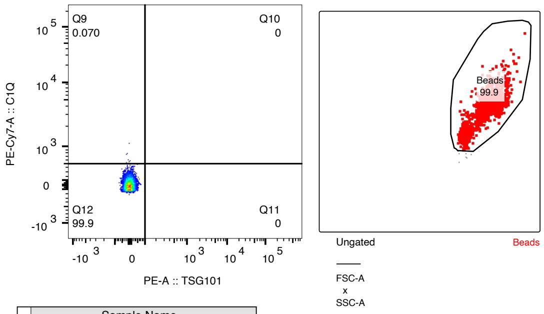

Sample Name
Specimen_001_3D_RAV337_IgG_011.fcs

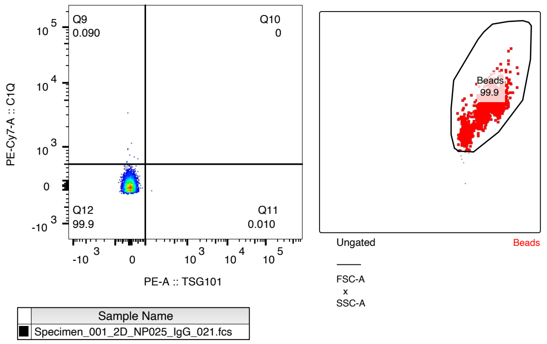
Q9
0.090
Q10
0
Q12
99.9
Q11
0.010
PE-Cy7-A :: C1Q
PE-A :: TSG101
Beads
99.9
Ungated
Beads
FSC-A
x
SSC-A
Sample Name
Specimen_001_2D_NP025_IgG_021.fcs

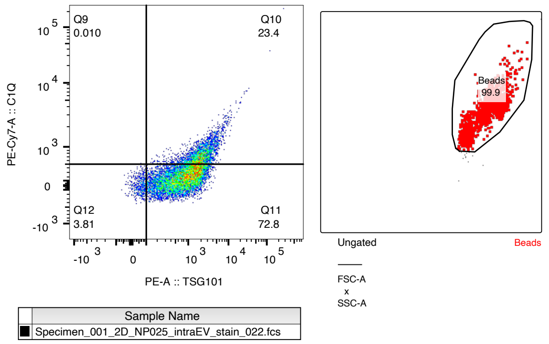
Q9
0.010
Q10
23.4
Q12
3.81
Q11
72.8
PE-Cy7-A :: C1Q
PE-A :: TSG101
Beads
99.9
Ungated
Beads
FSC-A
x
SSC-A
Sample Name
Specimen_001_2D_NP025_intraEV_stain_022.fcs

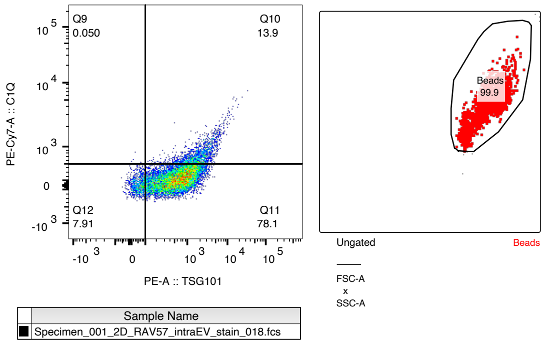
Q9
0.050
Q10
13.9
Q12
7.91
Q11
78.1
PE-Cy7-A :: C1Q
PE-A :: TSG101
Beads
99.9
Ungated
Beads
FSC-A
x
SSC-A
Sample Name
Specimen_001_2D_RAV57_intraEV_stain_018.fcs

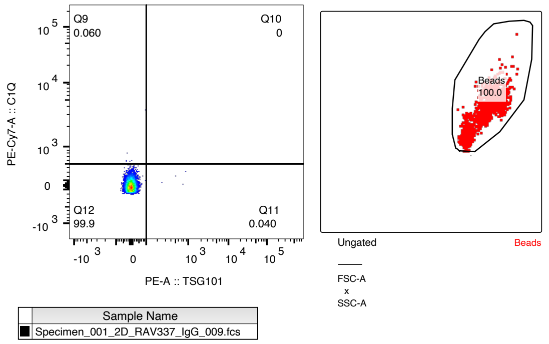
Q9
0.060
Q10
0
Q12
99.9
Q11
0.040
PE-Cy7-A :: C1Q
PE-A :: TSG101
Beads
100.0
Ungated
Beads
FSC-A
x
SSC-A
Sample Name
Specimen_001_2D_RAV337_IgG_009.fcs

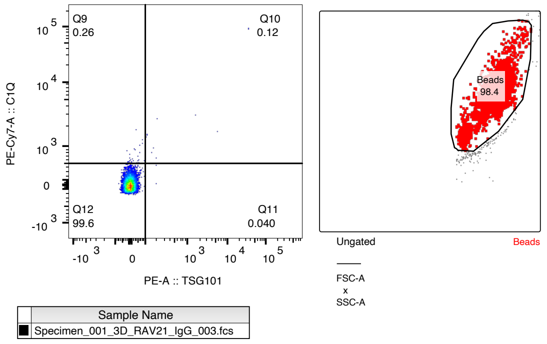
Q9
0.26
Q10
0.12
Q12
99.6
Q11
0.040
PE-Cy7-A :: C1Q
PE-A :: TSG101
Beads
98.4
Ungated
Beads
FSC-A
x
SSC-A
Sample Name
Specimen_001_3D_RAV21_IgG_003.fcs

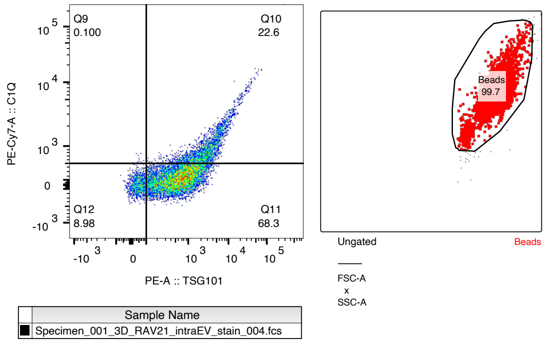
Q9
0.100
Q10
22.6
Q12
8.98
Q11
68.3
PE-Cy7-A :: C1Q
PE-A :: TSG101
Beads
99.7
Ungated
Beads
FSC-A
x
SSC-A
Sample Name
Specimen_001_3D_RAV21_intraEV_stain_004.fcs

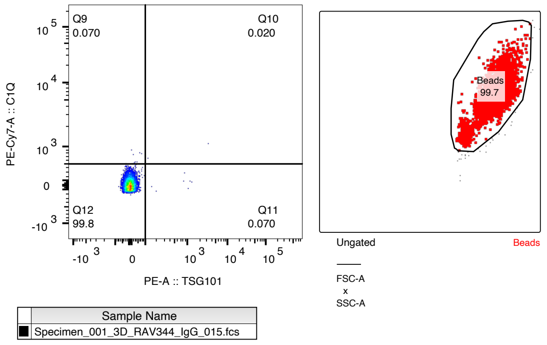
Q9
0.070
Q10
0.020
Q12
99.8
Q11
0.070
PE-Cy7-A :: C1Q
PE-A :: TSG101
Beads
99.7
Ungated
Beads
FSC-A
x
SSC-A
Sample Name
Specimen_001_3D_RAV344_IgG_015.fcs

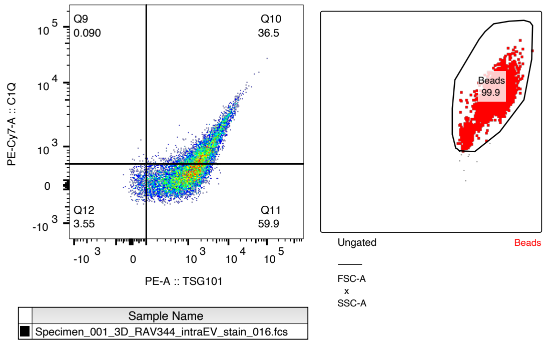
Q9
0.090
Q10
36.5
Q12
3.55
Q11
59.9
PE-Cy7-A :: C1Q
PE-A :: TSG101
Beads
99.9
Ungated
Beads
FSC-A
x
SSC-A
Sample Name
Specimen_001_3D_RAV344_intraEV_stain_016.fcs

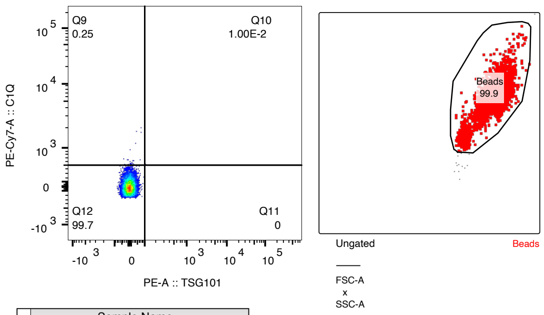

Sample Name
Specimen_001_2D_RAV21_IgG_001.fcs

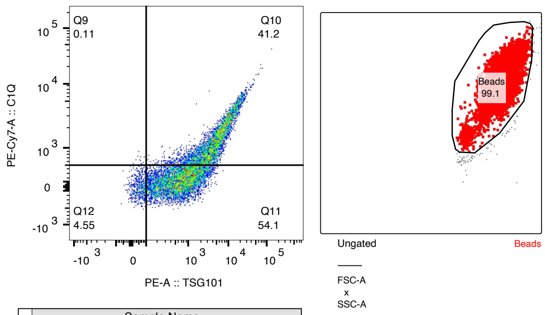

Sample Name
Specimen_001_2D_RAV21_intraEV_stain_002.fcs

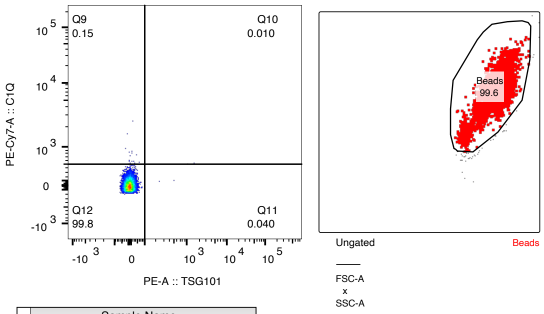

Sample Name
Specimen_001_2D_RAV344_IgG_013.fcs

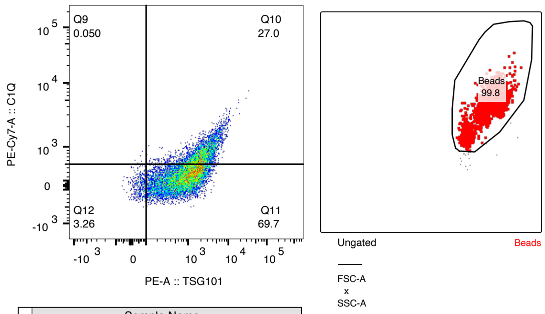

Sample Name
Specimen_001_2D_RA337_intraEV_stain_010.fcs

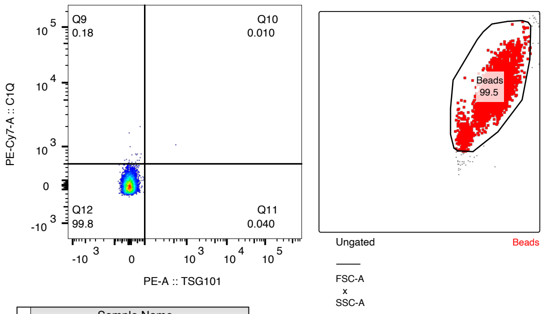

Sample Name
Specimen_001_3D_RAV27_IgG_007.fcs

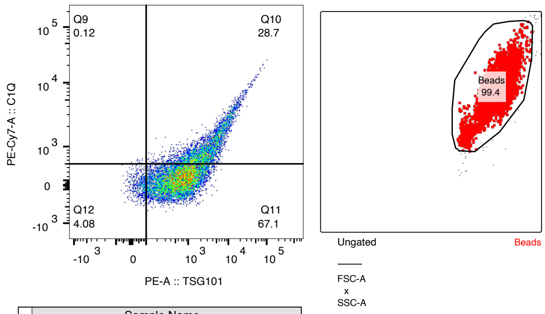

Sample Name
Specimen_001_3D_RAV27_intraEV_stain_008.fcs

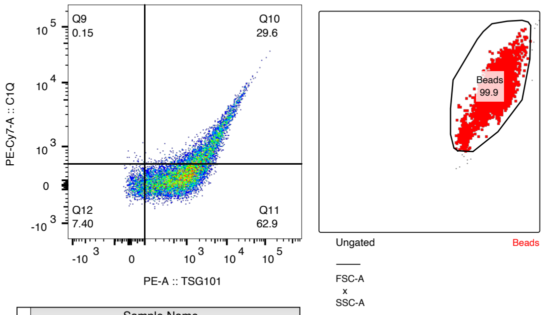

Sample Name
Specimen_001_3D_RA337_intraEV_stain_012.fcs

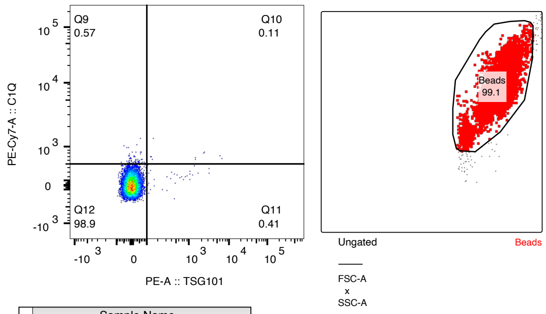

	Sample Name
■	Specimen_001_2D_RAV27_IgG_005.fcs

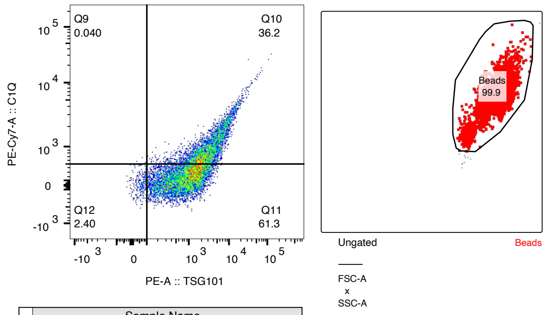

	Sample Name
■	Specimen_001_2D_RA344_intraEV_stain_014.fcs

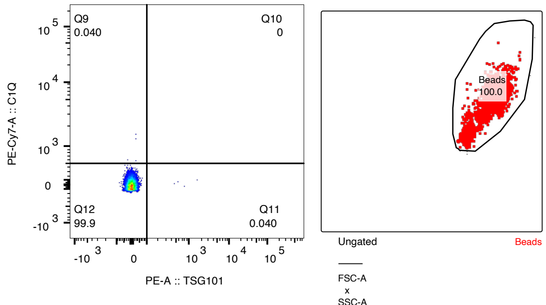

	Sample Name
■	Specimen_001_3D_RAV57_IgG_019.fcs

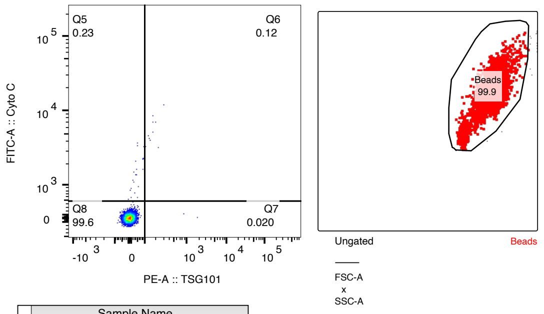

Sample Name
Specimen_001_EF_IgG_025.fcs

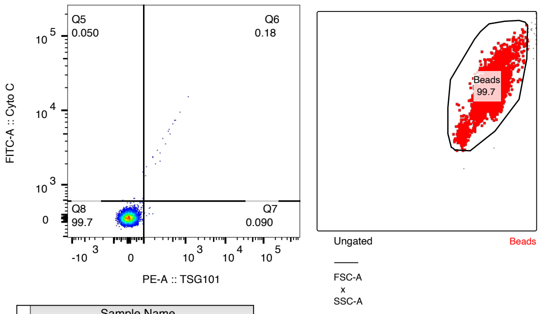

Sample Name
Specimen_001_EF_intraEV_stain_026.fcs

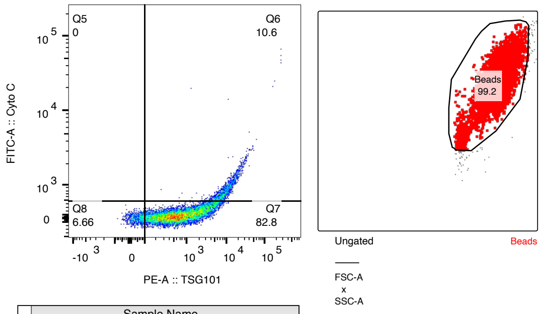

Sample Name
Specimen_001_2D_RAV27_intraEV_stain_006.fcs

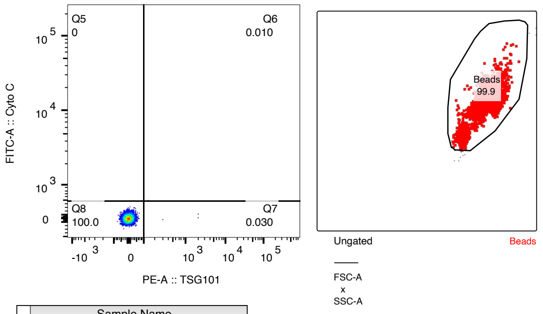

Sample Name
Specimen_001_2D_RAV57_IgG_017.fcs

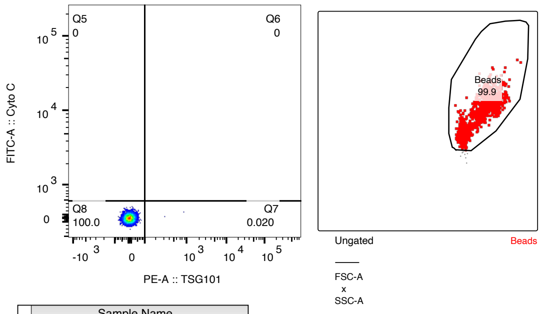

Sample Name
Specimen_001_3D_NP025_IgG_023.fcs

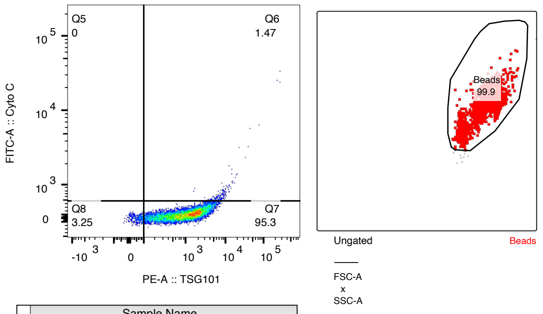

Sample Name
Specimen_001_3D_NP025_intraEV_stain_024.fcs

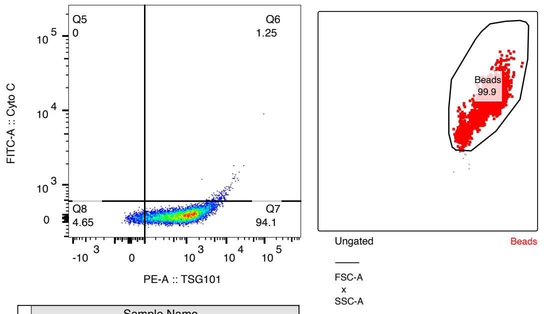

Sample Name
Specimen_001_3D_RAV57_intraEV_stain_020.fcs

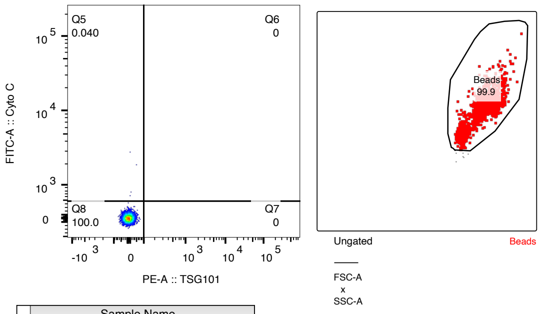

Sample Name
Specimen_001_3D_RAV337_IgG_011.fcs

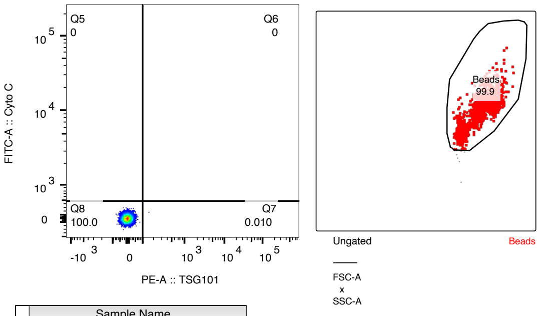

	Sample Name
■	Specimen_001_2D_NP025_IgG_021.fcs

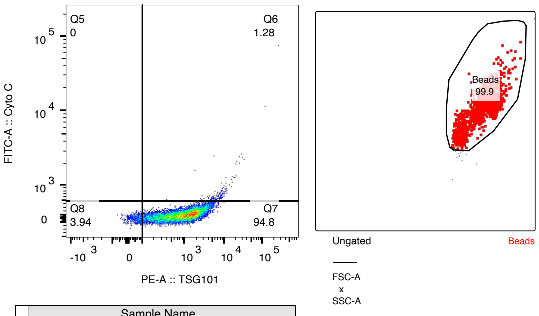

	Sample Name
■	Specimen_001_2D_NP025_intraEV_stain_022.fcs

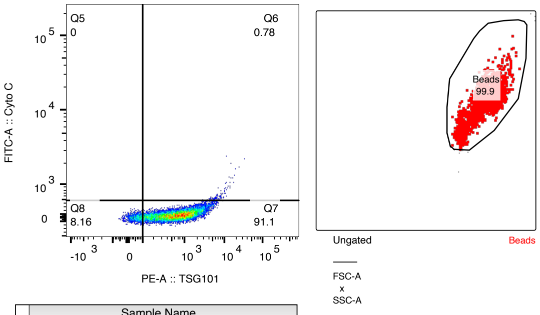

	Sample Name
■	Specimen_001_2D_RAV57_intraEV_stain_018.fcs

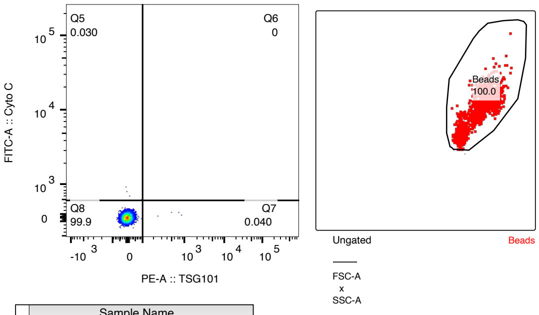

	Sample Name
■	Specimen_001_2D_RAV337_IgG_009.fcs

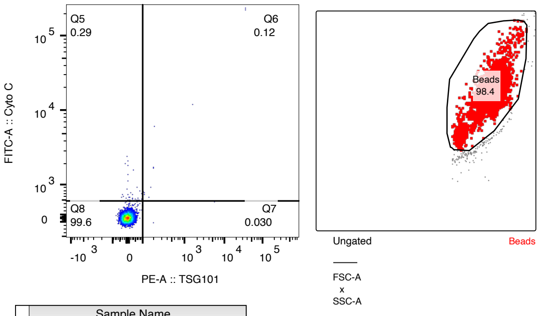

	Sample Name
■	Specimen_001_3D_RAV21_IgG_003.fcs

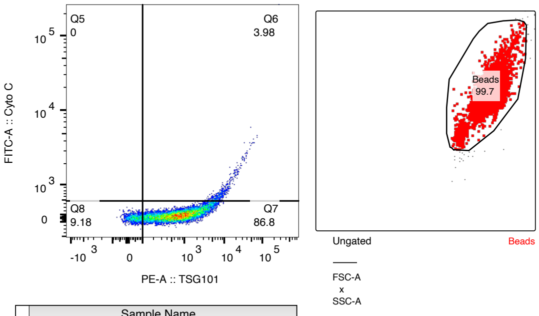

	Sample Name
■	Specimen_001_3D_RAV21_intraEV_stain_004.fcs

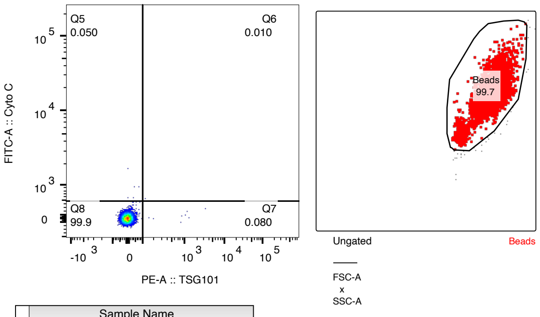

	Sample Name
■	Specimen_001_3D_RAV344_IgG_015.fcs

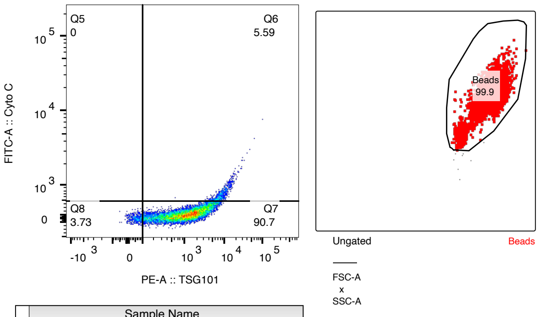

	Sample Name
■	Specimen_001_3D_RAV344_intraEV_stain_016.fcs

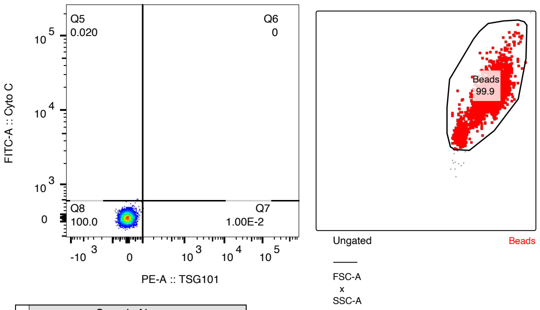

Sample Name
Specimen_001_2D_RAV21_IgG_001.fcs

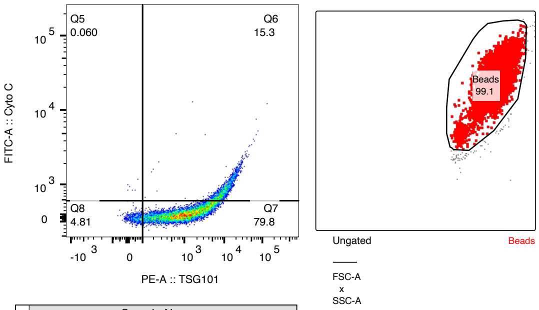

Sample Name
Specimen_001_2D_RAV21_intraEV_stain_002.fcs

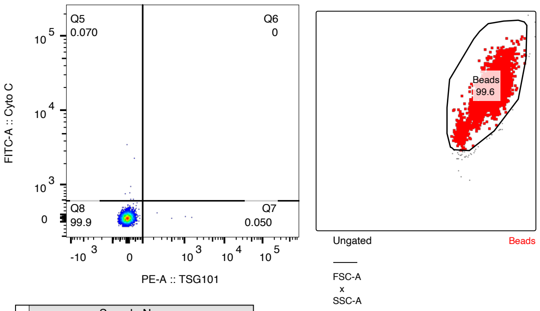

Sample Name
Specimen_001_2D_RAV344_IgG_013.fcs

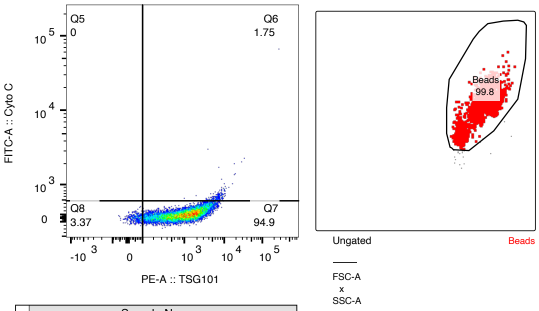

Sample Name
Specimen_001_2D_RA337_intraEV_stain_010.fcs

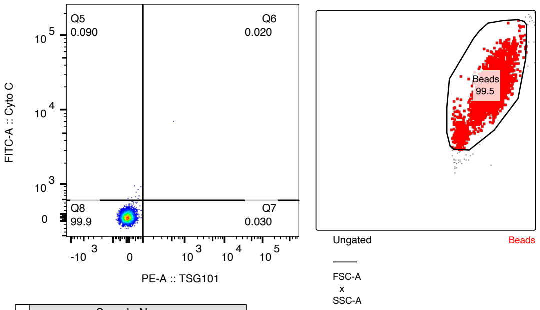

Sample Name
Specimen_001_3D_RAV27_IgG_007.fcs

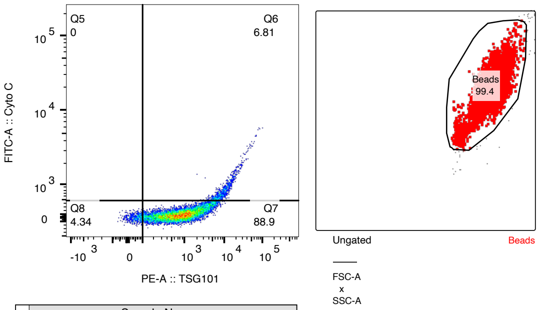

Sample Name
Specimen_001_3D_RAV27_intraEV_stain_008.fcs

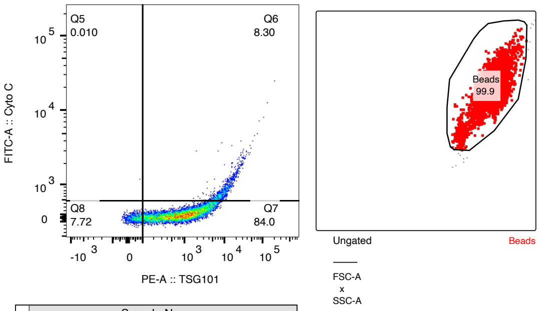

Sample Name
Specimen_001_3D_RA337_intraEV_stain_012.fcs

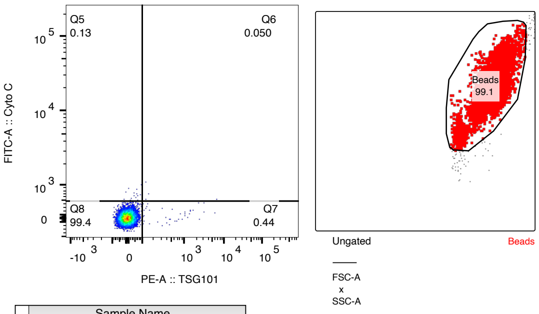

	Sample Name
■	Specimen_001_2D_RAV27_IgG_005.fcs

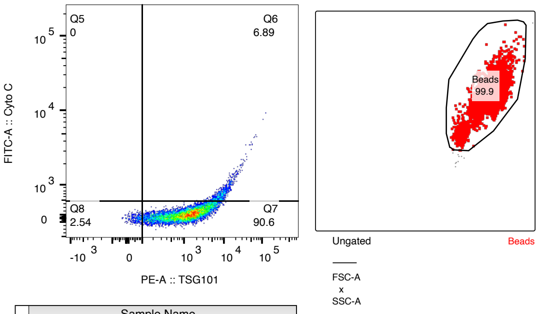

	Sample Name
■	Specimen_001_2D_RA344_intraEV_stain_014.fcs

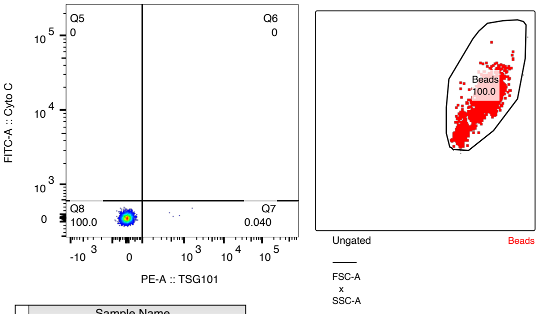

	Sample Name
■	Specimen_001_3D_RAV57_IgG_019.fcs
